# Cyclization of 1-aryl-4,4,4-trichlorobut-2-en-1-ones into 3-trichloromethylindan-1-ones in triflic acid

**DOI:** 10.3762/bjoc.19.105

**Published:** 2023-09-27

**Authors:** Vladislav A Sokolov, Andrei A Golushko, Irina A Boyarskaya, Aleksander V Vasilyev

**Affiliations:** 1 Department of Organic Chemistry, Institute of Chemistry, Saint Petersburg State University, Universitetskaya nab., 7/9, Saint Petersburg, 199034, Russia; 2 Department of Chemistry, Saint Petersburg State Forest Technical University, Institutsky per., 5, Saint Petersburg, 194021, Russia; 3 SAMS Research Group, University of Strasbourg, Institut Charles Sadron, CNRS, 67200 Strasbourg, France

**Keywords:** carbocations, enones, indanones, trichloromethyl group, triflic acid

## Abstract

Trichloromethyl-substituted enones (1-aryl-4,4,4-trichlorobut-2-en-1-ones, ArCOCH=CHCCl_3_, CCl_3_-enones) undergo intramolecular transformation into 3-trichloromethylindan-1-ones (CCl_3_-indanones) in Brønsted superacid CF_3_SO_3_H (triflic acid, TfOH) at 80 °C within 2–10 h in yields up to 92%. Protonation of the carbonyl oxygen of the starting CCl_3_-enones by TfOH affords the key reactive intermediates, the O-protonated forms ArC(=OH^+^)CH=CHCCl_3_, which are then cyclized into the target CCl_3_-indanones. These cations have been studied experimentally by means of NMR spectroscopy in TfOH and theoretically by DFT calculations. Under the same superacidic conditions in TfOH, CCl_3_-hydroxy ketones (1-aryl-4,4,4-trichloro-3-hydroxybutan-1-ones; ArCOCH_2_CH(OH)CCl_3_) undergo dehydration to the corresponding CCl_3_-enones, which are further cyclized into CCl_3_-indanones. The yields of CCl_3_-indanones starting from CCl_3_-hydroxy ketones are up to 86% in TfOH at 80 °C within 3–18 h.

## Introduction

Superelectrophilic activation of organic compounds under the action of strong Brønsted and Lewis acids is an effective method for the synthesis of various carbocycles and heterocycles, and polyfunctional compounds (see books [1–2] and reviews [3–10]). Protonation (or coordination) of basic centers (carbons of unsaturated bonds and heteroatoms) of organic molecules in Brønsted (or Lewis) acids gives rise to not only monocations, but also to highly reactive dicationic (and even higher charged) species. Thus, different conjugated enones afford O,C-diprotonated forms under superelectrophilic activation conditions. These dications can participate in electrophilic aromatic substitution reactions with arenes ([11] and references therein).

**Scheme 1:**
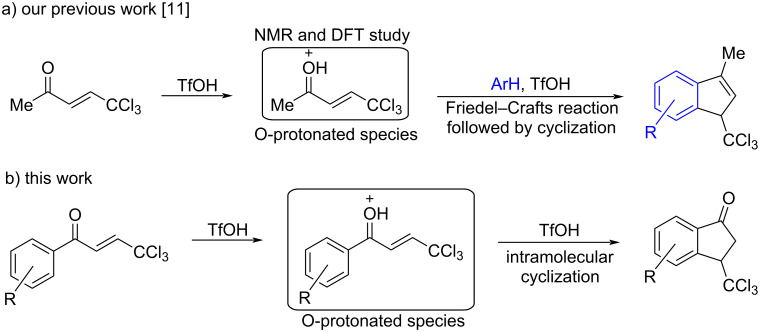
Generation of O-protonated and O,C-diprotonated species from substituted conjugated enones under superelectrophilic activation and their subsequent transformations.

Recently, we have shown that the reaction of (*E*)-5,5,5-trichloropent-3-en-2-one [Cl_3_CCH=CHC(=O)Me] with arenes in Brønsted superacid TfOH (triflic acid, CF_3_SO_3_H) furnishes 3-methyl-1-trichloromethylindenes (Scheme 1a) [11]. Based on NMR analysis in TfOH and theoretical DFT calculations, it has been found that the reaction proceeds through an intermediate formation of the O-protonated form of the starting compound [Cl_3_CCH=CHC(=OH^+^)Me]. The presence of two strong electron-withdrawing substituents, the trichloromethyl group (CCl_3_) and a protonated carbonyl (C(OH^+^)Me), at the carbon–carbon double bond makes this O-protonated species electrophilic enough to react with arenes (Scheme 1a). The second protonation of the C=C bond is hampered due to a strong acceptor character of the substituents, contrary to other more donating enones.

As a continuation of the research on the electrophilic activation of electron-poor alkenes bearing two electron-withdrawing substituents at the C=C bond, we initiated this study on transformations of 1-aryl-4,4,4-trichlorobut-2-en-1-ones under superelectrophilic activation conditions (Scheme 1b). The main goals of this work were the investigation of the protonation of CCl_3_-enones (1-aryl-4,4,4-trichlorobut-2-en-1-ones) by NMR spectroscopy and DFT calculations, and to study their intramolecular cyclization in triflic acid into the synthetically and medicinally relevant (see recent reviews [12–18]) indan-1-ones.

## Results and Discussion

**Scheme 2:**
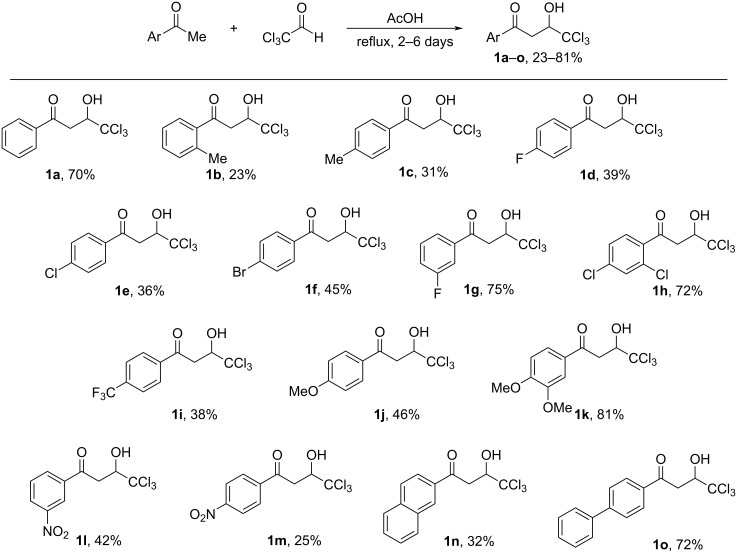
Synthesis of 1-aryl-4,4,4-trichloro-3-hydroxybutan-1-ones **1a**–**o** by condensation of acetophenones with chloral in refluxing acetic acid.

**Scheme 3:**
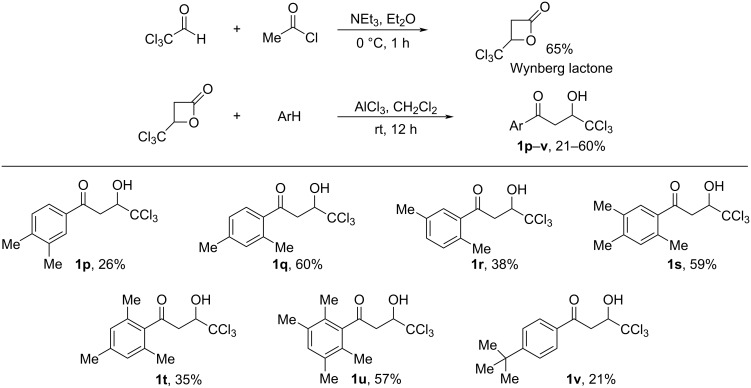
Synthesis of 1-aryl-4,4,4-trichloro-3-hydroxybutan-1-ones **1p**–**v** by acylation of electron-donating arenes with Wynberg lactone.

The synthesis of 1-aryl-4,4,4-trichloro-3-hydroxybutan-1-ones (CCl_3_-hydroxy ketones) **1a–o** was carried out by condensation of acetophenones with chloral under reflux in acetic acid using the known literature procedure [19] (Scheme 2). Based on another literature approach [20], compounds **1p–v** were obtained by acylation of electron-donating arenes with Wynberg lactone [21] (Scheme 3). Additionally, exact structures of compounds **1g**,**h**,**s**,**t**,**v** were confirmed by X-ray analysis (see Supporting Information File 1).

**Scheme 4:**
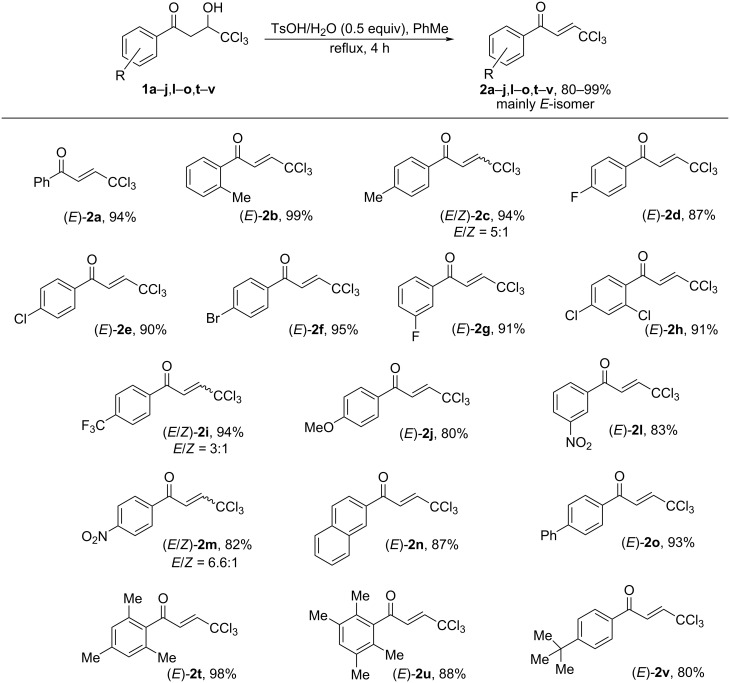
Synthesis of 1-aryl-4,4,4-trichlorobut-2-en-1-ones **2** by dehydration of hydroxy ketones **1**.

The hydroxy ketones **1** were used as precursors for the synthesis of 1-aryl-4,4,4-trichlorobut-2-en-1-ones (CCl_3_-enones) **2** by dehydration of compounds **1** with *p*-toluenesulfonic acid monohydrate at reflux in toluene [19] (Scheme 4). By this route, mainly *E*-isomers of compounds **2** were formed except for compounds **2c**,**i**,**m** which were obtained as mixtures of *E,Z-*isomers (see Experimental section). However, under the reaction conditions in the presence of TsOH, the hydroxy ketones **1k**,**p–s** bearing strong electron-donating substituents in the aromatic ring gave oligomeric materials. Presumably, in these cases, after dehydration and formation of the corresponding enone **2**, the latter underwent subsequent cationic oligomerization.

**Scheme 5:**
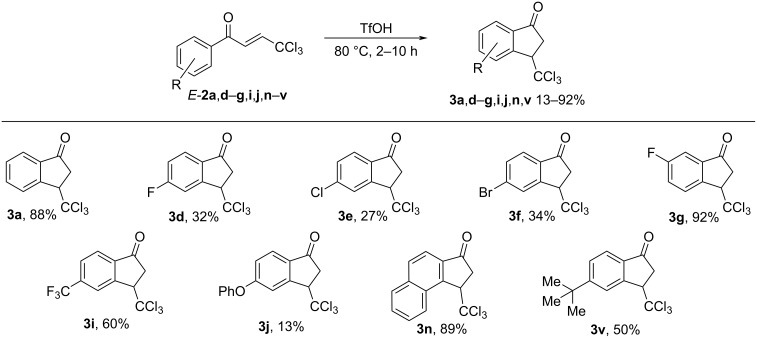
Cyclization of 1-aryl-4,4,4-trichlorobut-2-en-1-ones **2** into 3-trichloromethylindan-1-ones **3** in TfOH.

**Scheme 6:**
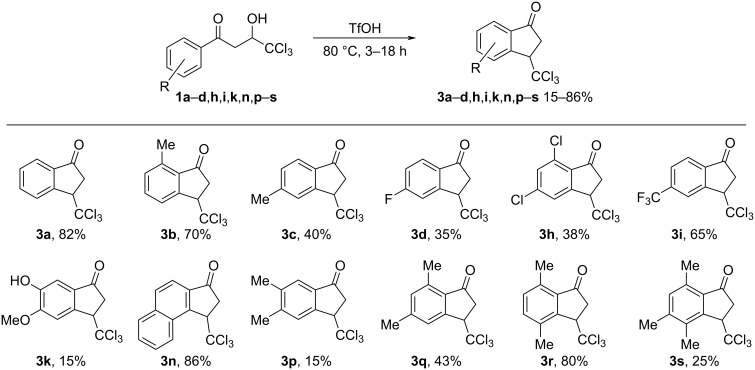
Cyclization of 1-aryl-4,4,4-trichloro-3-hydroxybutan-1-ones **1** into 3-trichloromethylindan-1-ones **3** in TfOH.

Next we studied the intramolecular cyclization of compounds **1** and **2** in TfOH. It was found that enones **2** were transformed into the corresponding 3-trichloromethylindan-1-ones **3** upon heating in neat TfOH at 80 °C for 2–10 h (Scheme 5). Under the same reaction conditions, hydroxy ketones **1** were cyclized into indanones **3** as well (Scheme 6). The structure of compound **3a** was confirmed by X-ray analysis (see Supporting Information File 1). Both, hydroxy ketone **1** and the corresponding enone **2**, can be converted into the same indanone **3** in comparable yields; see pairs of reactions for **1a** and **2a** (indanone **3a**), **1d** and **2d** (indanone **3d**), **1i** and **3i** (indanone **3i**), and **1n** and **3n** (indanone **3n**) in Scheme 5, Scheme 6 and the Experimental section).

There are some features of this cyclization. The electron-poor enones **2l**,**m** are unreactive and do not give rise to the corresponding indanones due to the low nucleophilicity of the nitro-substituted aromatic ring. On the other hand, the electron-rich enones **2o**,**t**,**u**, bearing electron-donating substituents in the aromatic rings, afford complex mixtures of oligomeric materials. The cyclization of hydroxy ketone **1k** into indanone **3k** is accompanied by demethylation of one of the methoxy groups (Scheme 6). The positions of hydroxy and methoxy groups in the aryl ring of compound **3k** were determined by NOESY correlations between protons in this structure (see Supporting Information File 1). Surprisingly, enone **2j** transformed into the phenoxy-substituted indanone **3j** in a low yield of 13% (Scheme 5). The formation of the latter represents an interesting rearrangement with the intermolecular transfer of a phenyl group in the starting methoxy-substituted enone **2j** under the rather harsh reaction conditions (TfOH at 80 °C).

To study the cyclization further, reactions of some hydroxy ketones **1** were run for shorter time (1–4.5 h) in TfOH at 80 °C, i.e., under conditions of incomplete conversion of the starting compounds. It was found that, apart from the target indanones **3**, substantial amounts of the corresponding enones **2** were detected (Table 1). This means that, in the superacid TfOH, hydroxy ketones **1** may initially undergo dehydration to enones **2**, which are subsequently cyclized into indanones **3**. From a synthetic point of view, the use of hydroxy ketones **1** as starting compounds for the cyclization without additional preparation and isolation of the corresponding enones **2** is more economical as it reduces the number of steps in the synthesis.

**Table 1:** Transformations of hydroxy ketones **1a**,**c**,**f**,**i** into the corresponding enones **2** and indanones **3** in TfOH at 80 °C for shorter reaction time under the conditions of incomplete conversion of the starting compounds.^a^

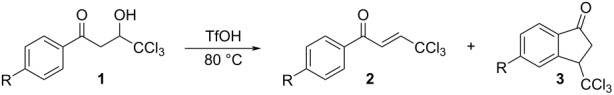				

Entry	Starting hydroxy ketone **1**	Reaction time, h	Ratio of compounds hydroxy ketone **1**/enone **2**/indanone **3**	Substituent, R in **1**, **2**, and **3**

1	**1a**	1	**1a**/**2a**/**3a** 1:2.6:5	H
2	**1c**	3	**1c**/**2c**/**3c** 5:1.8:1	Me
3	**1f**	3	**1f**/**2f**/**3f** 0:2:1	Br
4	**1i**	4.5	**1i**/**2i**/**3i** 0:1:5	CF_3_

^a^Ratio of compounds **1**, **2**, and **3** was determined by NMR.

Other Brønsted and Lewis acids were also tested for this cyclization. Thus, enones **2a** and **2e** were not transformed into the corresponding indanones **3** in neat sulfuric acid (H_2_SO_4_) at room temperature for 3 days. That is in accord with literature data [19], where H_2_SO_4_ was used for dehydration of hydroxy ketones **1** into enones **2**, and no cyclization into indanones was detected in this acid. However, in the current study, we carried out the reaction of electron-donating naphthyl-substituted enone **2n** in H_2_SO_4_ at room temperature, which resulted in the quantitative formation of indanone **3n** after 3 days. The reactions of enones **2a**,**e**,**n** with an excess (5 equiv) of AlBr_3_ or AlCl_3_ in CH_2_Cl_2_ solution at room temperature for 3 days afforded complex mixtures of compounds.

Then, the protonation of compounds **1** and **2** in TfOH was investigated by means of NMR spectroscopy. According to the ^1^H, ^13^C, and ^19^F NMR data, hydroxy ketones **1** and enones **2** afford stable O-protonated forms **A** and **B**, respectively, in TfOH at room temperature (Table 2 and spectra in Supporting Information File 1).

**Table 2:** ^1^H, ^13^C, and ^19^F NMR data of compounds **1**, and **2** in CDCl_3_ and their O-protonated forms **A**, and **B**, respectively, in TfOH.

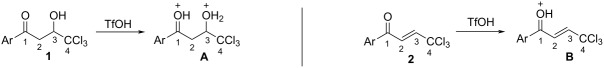							

compounds **1**, **2** and cations **A**, **B**, respectively	solvent	^1^H NMR, δ, ppm, *J*, Hz		^13^C NMR, δ, ppm			
		H^2^	H^3^	C^1^	C^2^	C^3^	C^4^

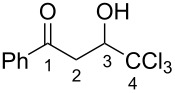 **1a**	CDCl_3_	3.68 dd, *J* = 17.4, 2.0; 3.52 dd, *J* = 17.4, 8.9 *AB system*	4.91 ddd, *J* = 8.9, 4.4, 2.0	197.1	40.7	79.0	102.5
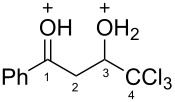 **Aa**	TfOH^a^	4.54 dd, *J* = 18.7, 4.1; 4.15 dd, *J* = 18.7, 7.6 *AB system*	5.21 dd, *J* = 7.6, 4.1	218.2	34.2	81.0	98.9
	Δδ^b^	0.86 0.63	0.30	21.1	−6.5	2.0	−3.6
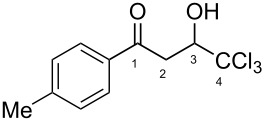 **1c**	CDCl_3_	3.66 dd, *J* = 17.3, 2.0; 3.48 dd, *J* = 17.3, 9.0 *AB system*	4.87 dd, *J* = 8.9	196.8	40.5	79.1	102.5
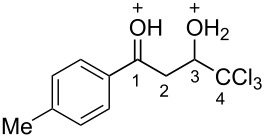 **Ac**	TfOH^a^	4.46 dd, *J* = 18.3, 3.9; 4.07 dd, *J* = 18.3, 7.8 *AB system*	5.15 dd, *J* = 7.8, 3.9	214.5	33.9	80.9	99.0
	Δδ^b^	0.80 0.59	0.28	17.7	−6.6	1.8	−3.5
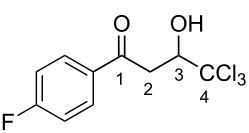 **1d**	CDCl_3_	3.62 dd, *J* = 17.3, 2.2; 3.50 dd, *J* = 17.3, 8.8 *AB system*	4.89 dd, *J* = 8.8, 2.2	195.4	40.7	79.0	102.6
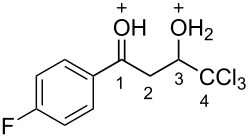 **Ad**	TfOH^a^	4.49 dd, *J* = 18.6, 4.0; 4.11 dd, *J* = 18.6, 7.6 *AB system*	5.20 dd, *J* = 7.6, 4.0	215.3	34.1	80.9	98.8
^19^F NMR, δ, ppm −103.6 → −77.9 Δδ^a^ = 25.7	Δδ^b^	0.81 0.61	0.31	19.9	−6.6	1.9	−3.8
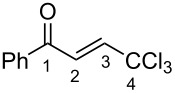 (*E*)-**2a**	CDCl_3_	7.27 d, *J* = 17.6	7.42 d, *J* = 17.6	189.0	124.3	145.6	93.1
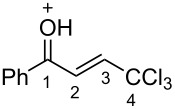 **Ba**	TfOH^a^	7.86 d, *J* = 12.0	7.90 d, *J* = 12.0	204.9	120.7	159.0	90.0
	Δδ^b^	0.61	0.46	15.9	−3.6	13.4	−3.1
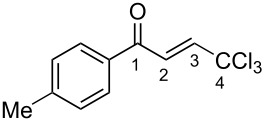 (*E*)-**2c**	CDCl_3_	7.28 d, *J* = 14.6	7.43 d, *J* = 14.6	188.3	124.2	145.2	93.1
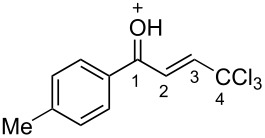 **Bc**	TfOH^a^	7.82 d, *J* = 12.0	7.84 d, *J* = 12.0	201.0	120.4	163.3	90.2
	Δδ^b^	0.54	0.41	12.7	−3.8	8.1	−2.9
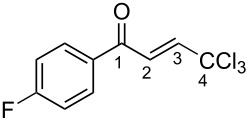 (*E*)-**2d**	CDCl_3_	7.26 d, *J* = 14.5	7.38 d, *J* = 14.5	187.3	123.9	146.8	93.0
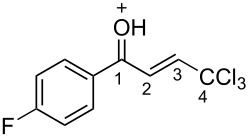 **Bd**	TfOH	7.80 d, *J* = 16.0	7.84 d, *J* = 16.0	202.0	120.4	158.1	90.2
^19^F NMR, δ, ppm −103.2 → −77.9 Δδ^a^ = 25.3	Δδ^a^	0.54	0.46	14.7	−3.5	11.3	−2.8
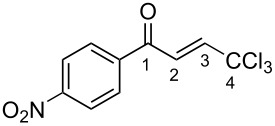 **2m**	CDCl_3_	7.35 d, *J* = 14.6	7.42 d, *J* = 14.6	187.5	124.1	146.9	92.4
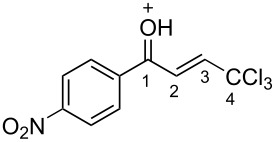 **Bm**	TfOH^a^	7.85 d, *J* = 14.9	7.96 d, *J* = 14.9	208.8	121.6	162.9	90.2
	Δδ^b^	0.50	0.54	21.3	−2.5	6.0	−2.2

^а^CH_2_Cl_2_ was used as internal standard; ^b^∆δ = δ_acid_ − δ_CDCl3_.

In species **A** both oxygens should be protonated by the superacid. The differences in chemical shifts (∆δ = δ_acid_ − δ_CDCl3_) for the corresponding atoms H^2^ and H^3^ in cations **Aa**,**c**,**d** and their neutral precursors **1a**,**c**,**d** were 0.59–0.86 and 0.28–0.31 ppm (Table 2). These downfield shifts of the signals point to an inductively induced positive charge on these hydrogens due to the protonation of the oxygens of the carbonyl and hydroxy groups. According to the ^13^C NMR spectra, the largest downfield shift was observed for the carbonyl carbon С^1^, with ∆δ = 17.7–21.1 ppm, showing a substantial degree of protonation of the carbonyl group in TfOH.

The tendencies are the same for the protonation of enones **2a**,**c**,**d**,**m** leading to cations **Ba**,**c**,**d**,**m** (Table 2). Thus, in the ^1^H NMR spectra, downfield shifts of vinyl protons H^2^ and H^3^ upon protonation were 0.50–0.61 and 0.41–0.54 ppm, respectively. In the ^13^C NMR spectra, ∆δ values for carbons С^1^ and С^3^ were 12.7–21.3 and 6.0–13.4 ppm, respectively. The NMR data revealed that the positive charge in the O-protonated forms **B** is substantially delocalized from the carbonyl group to vinyl carbon C^3^.

For fluorophenyl-substituted compounds and cations **1d** and **Ad**, **2d** and **Bd**, also a large downfield shift of the corresponding fluorine signals is observed in the ^19^F NMR spectra (∆δ = 25.3–25.7 ppm), which shows a delocalization of the positive charge from the carbonyl group to the aromatic ring.

It should be mentioned that cation **Bm** was generated by two ways in TfOH: either directly by protonation of enone **2m** or from hydroxy ketone **1m**. The latter was found to undergo fast dehydration into enone **2m** in TfOH at room temperature.

Then, we carried out DFT calculations of cations **Aa–Da** derived from protonation of compounds **1a** and **2a**. Thermodynamics of their formation, as Gibbs energies Δ*G*_298_ of the corresponding reactions, energies of HOMO/LUMO, electrophilicity indices ω [22–23], charge distribution, and contribution of atomic orbital into LUMO in species **Aa–Da** were estimated (Table 3).

**Table 3:** Selected calculated (DFT) electronic characteristics of the protonated forms **Aa**, **Ba**, **Ca**, and **Da** generated from hydroxy ketone **1a** and enone **2a**, and values of Gibbs energies of reactions (Δ*G*, kJ/mol).

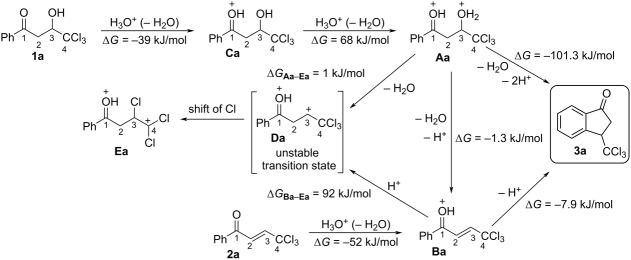								

Entry	Species	*E*_HOMO_, eV	*E*_LUMO_, eV	ω,^a^ eV	q(C^1^),^b^ e	q(C^3^),^b^ e	k(C^1^)_LUMO_,^с^ %	k(C^3^)_LUMO_,^с^ %

1	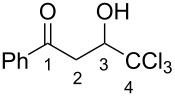 **1a**	−7.34	−2.09	2.1	0.60	0.08	15.6	18.2
2	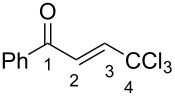 **2a**	−7.37	−2.86	2.9	0.54	−0.18	10.5	12.7
3	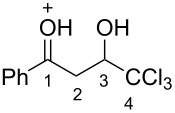 **Ca**	−7.93	−3.67	3.9	0.66	0.07	25.0	3.1
4	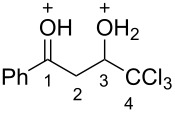 **Aa**	−8.05	−3.97	4.4	0.66	0.07	28.9	12.7
5	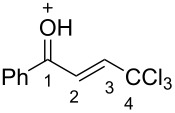 **Ba**	−7.93	−4.12	4.8	0.60	-0.13	14.9	12.1
6	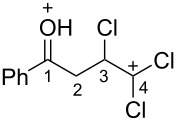 **Ea**	−8.10	−5.50	8.9	0.65	−0.28	3.3	14.4

^a^Global electrophilicity index ω = (*E*_HOMO_ + *E*_LUMO_)^2^/8(*E*_LUMO_−*E*_HOMO_); ^b^natural charges; ^c^contribution of atomic orbital to the molecular orbital.

The formation of O-monoprotonated species **Ba** and **Ca** from enone **2a** and hydroxy ketone **1a** is favorable, with negative Δ*G*_298_ values for protonation of −52 and −39 kJ/mol, respectively (see overview scheme of Table 3). The second protonation of the C=C bond of species **Ba** is very unfavorable. Moreover, the corresponding O,C-diprotonated form **Da** was found to be extremely unstable and spontaneously rearranges into species **Ea** via a shift of a chlorine atom. Therefore, the DFT calculations and NMR data for enones **2** in TfOH (Table 2) indicate that the formation of dications **D** does not take place. Species **B** should be the key reactive intermediates that undergo cyclization into indanones **3** with a negative Gibbs energy of −7 kJ/mol for the reaction **Ba**→**3a**.

According to the calculations, the subsequent complete protonation of the hydroxy group in species **Ca** leading to dication **Aa** is thermodynamically unfavorable (Δ*G*_298_ = 68 kJ/mol) as well (see overview scheme of Table 3). NMR experiments allowed us to observe the formation of O,O-diprotonated species in the superacid TfOH (Table 2). The dication **Aa** should not be transformed to species **Da** analogously to cation **Ba**. The cyclization of dication **Aa** into indanone **3a** is very favorable (Δ*G*_298_ = −101.3 kJ/mol). However, the formation of both enones **2** and indanones **3** from hydroxy ketones **1** is observed in TfOH (Table 1). We assume that, at first, hydroxy ketones **1** undergo dehydration into enones **2**, which then cyclize into the target indanones **3**. Despite thermodynamic gain in energy, the cyclization of dications **Aa** in compounds **3** may have a high activation barrier.

The calculations showed that the largest part of the positive charge in the key reactive species **Ba** is localized on the carbonyl carbon C^1^ (0.60 e). This carbon atom gives 14.9% contribution to the LUMO. Contrary to that, the carbon C^3^ bears no positive charge (−0.13 e) and contributes 12.1% to the LUMO. The intramolecular cyclization of cation **Ba** takes place between the atom C^3^ and the *ortho*-carbons of the phenyl ring. Thus, electrophilic properties of atom C^3^ should be mainly explained by orbital factors, rather than charge ones.

**Scheme 7:**
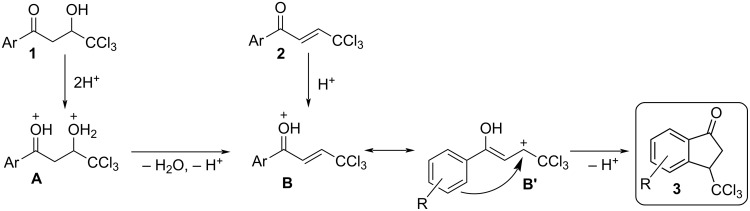
Plausible mechanisms for the cyclization of compounds **1** and **2** into indanones **3** in TfOH.

Summarizing the data obtained during the synthesis of indanones **3** (Scheme 5, Scheme 6 and Table 1), NMR, and DFT studies on intermediate cations (Table 2 and Table 3), one may propose plausible reaction mechanisms for the cyclization of compounds **1** and **2** into indanones **3** in TfOH (Scheme 7). Protonation of the carbonyl oxygen of enone **2** gives rise to cation **B** which is followed by cyclization into indanone **3** through mesomeric form **B'**. The hydroxy ketone **1** is protonated at the oxygen atoms leading to cation **A**, followed by dehydration resulting in cation **B**, which is cyclized into indanone **3**. The transformation of species **B** into indanone **3** can be also considered as a variant of Nazarov cyclization. It should be noted that such 3-trichloromethylindanones **3** have been obtained for the first time in this study, which structurally resemble the known 3-dichloromethylindanones [24] and 3-trifluoromethylindanones [25–27].

## Conclusion

A method for the synthesis of CCl_3_-indanones (3-trichloromethylindan-1-ones), as a novel class of indanones, has been developed and involves an intramolecular cyclization of both, CCl_3_-enones (1-aryl-4,4,4-trichlorobut-2-en-1-ones) and CCl_3_-hydroxy ketones (1-aryl-4,4,4-trichloro-3-hydroxybutan-1-ones) in Brønsted superacid TfOH at an elevated temperature of 80 °C within 2–18 h. In both cases, the reaction proceeds through an intermediate formation of the O-protonated carbonyl form of the CCl_3_-enones, that are finally cyclized into the target CCl_3_-indanones.

## Supporting Information

File 1 Experimental, characterization data and copies of spectra.
