# Supplementary material for: Cyclization of 1-aryl-4,4,4-trichlorobut-2-en-1-ones into 3-trichloromethylindan-1-ones in triflic acid
Source: Beilstein J Org Chem. 2023 Sep 27;19:1460–70. doi: 10.3762/bjoc.19.105 (PMC10548255; doi:10.3762/bjoc.19.105)
Supplement: File 1 — Experimental, characterization data and copies of spectra. [file Beilstein_J_Org_Chem-19-1460-s001.pdf]

BEILSTEIN JOURNAL OF ORGANIC CHEMISTRY

Supporting Information

for

Cyclization of 1-aryl-4,4,4-trichlorobut-2-en-1-ones into 3-trichloromethylindan-1-ones in triflic acid

Vladislav A. Sokolov, Andrei A. Golushko, Irina A. Boyarskaya
and Aleksander V. Vasilyev

Beilstein J. Org. Chem. **2023,** *19,* 1460–1470. doi:10.3762/bjoc.19.105

Experimental, characterization data and copies of spectra

License and Terms: This is a supporting information file under the terms of the Creative Commons Attribution License (https://creativecommons.org/licenses/by/4.0). Please note that the reuse, redistribution and reproduction in particular requires that the author(s) and source are credited and that individual graphics may be subject to special legal provisions.
The license is subject to the *Beilstein Journal of Organic Chemistry* terms and conditions: (https://www.beilstein-journals.org/bjoc/terms)

Table of contents

1. Experimental part……………………………………………………………………..S2

2.References………………………………………………………………………………S17

3. ^{1}H, $^{13}C\{^{1}H\}$, $^{19}F\{^{1}H\}$, NOESY ^{1}H -^{1}H, NOESY ^{1}H-^{19}F NMR spectra of compounds **1–3**...S18

4. Copies of ^{1}H, $^{13}C\{^{1}H\}$, $^{19}F\{^{1}H\}$ NMR spectra of cations **Aa, Ac, Ad, Ba, Bc, Bd, Bm**…...S80

5. X-ray data of compounds **1g,h,s,t,v**, **3a** …………………………………………………S88

6. DFT-calculations data for compounds **1a**, **2a**, **3a** and cations **Aa, Ba, Ca, Ea**……….........S97

1. Experimental part

General information. The NMR spectra of solutions of compounds in $CDCl_3$ and in the acid CF_3SO_3H were recorded on Bruker 400 spectrometer at 25 °C at 400, 101, and 376 MHz for ^1H and ^{13}C, and ^{19}F NMR spectra, respectively. The residual proton solvent peak of $CDCl_3$ (δ 7.26 ppm) for ^1H NMR spectra, and the carbon signals of $CDCl_3$ (δ 77.0 ppm) for ^{13}C NMR spectra were used as references. ^{19}F NMR spectra were indirectly referred to the signal of $CFCl_3$ (δ 0.0 ppm). NMR spectra in the superacid TfOH were referenced to the signal of CH_2Cl_2 added as internal standard: δ 5.30 ppm for ^1H NMR spectra, and δ 53.52 ppm for ^{13}C NMR spectra. HRMS were carried out using Bruker maXis HRMS-ESI-QTOF and Varian 902-MS MALDI mass spectrometers. Preparative TLC was performed on silica gel (Merck Co., 5–40 μm) with hexane/ethyl acetate mixture elution.

Single-crystal X-ray analyses of **1g**,**h**,**s**,**t**,**v**, and **3a** were performed using a single-crystal diffractometer SuperNova, Single source at offset/far, HyPix3000. The crystals were kept at 100(5) K during data collection. Using Olex2 [1], the structures were solved with the ShelXT [2] structure solution program using Intrinsic Phasing and refined with the ShelXL [3] refinement package using least squares minimization. Supporting crystallographic data for this paper have been deposited at the Cambridge Crystallographic Data Centre (CCDC2237593 for **1g**, 2237594 for **1h**, 2237595 for **1s**, 2237596 for **1t**, 2237597 for **1v**, and 2237598 for **3a**) and can be obtained free of charge via www.ccdc.cam.ac.uk/data_request/cif.

DFT calculations. All computations were carried out at the DFT/HF hybrid level of theory using hybrid exchange functional B3LYP by using GAUSSIAN 2009 program packages [4]. The geometries optimization were performed using the 6-311+G(2d,2p) basis set (standard 6-311G basis set added with polarization (d,p) and diffuse functions). Optimizations were performed on all degrees of freedom and solvent phase optimized structures were verified as true minima with no imaginary frequencies. The Hessian matrix was calculated analytically for the optimized structures in order to prove the location of correct minima and to estimate the thermodynamic parameters. Solvent-phase calculations used the polarizable continuum model (PCM, solvent = water).

General procedure for the synthesis of 1-aryl-4,4,4-trichloro-3-hydroxybutane-1-ones 1a–o by condensation of acetophenones with chloral according to the literature method [5].

A solution of the acetophenone of choice (5.0 mmol) and chloral (1.11 g, 7.5 mmol) in 12 mL of glacial acetic acid was heated to reflux for 2 or 6 days with TLC monitoring of the reaction progress. Then, the reaction mixture was poured into water (50 mL). The reaction product was extracted with CH_2Cl_2 (2 × 20 mL); the organic phases were combined, washed

once with saturated aqueous solution of $NaHCO_3$ (20 mL), with water (2 × 20 mL), and dried with Na_2SO_4; the solvent was removed in vacuum. The obtained crude compounds **1a–o** were crystallized from cyclohexane, CCl_4, or ethyl acetate.

Procedure for the synthesis of Wynberg lactone (4-trichloromethyloxetan-2-one) according to the literature method [6]. A solution of NEt_3 (9.6 g, 95 mmol) in 40 mL of Et_2O was slowly added to the solution of chloral (7 g, 47.5 mmol) and AcCl (7.5 g, 95 mmol) in 75 mL of Et_2O with stirring at 0 °C. After 1 h, the reaction mixture was warmed to room temperature and diluted with saturated aqueous solution of NH_4Cl (70 mL). The organic phase was separated, washed with brine (2 × 40 mL), and dried with Na_2SO_4; the solvent was removed in vacuum. Yield: 5.9 g (65%). Yellow solid, mp 37-39°C (lit.: mp 51-52°C [31]). ^1H NMR ($CDCl_3$, 400 MHz): δ 5.02 dd (1H, J = 5.7, 3.6 Hz), 3.75 dd (1H, J = 11.4, 5.7 Hz), 3.61 dd (1H, J = 11.4, 3.6 Hz). ^{13}C NMR ($CDCl_3$, 101 MHz): δ 163.9, 96.7, 76.1, 42.5.

General procedure for the synthesis of 1-aryl-4,4,4-trichloro-3-hydroxybutane-1-ones 1p–v by acylation of arenes with Wynberg lactone according to the literature method [6]. $AlCl_3$ (528 mg, 4.0 mmol) was gradually added to the solution of Wynberg lactone (200 mg, 1.0 mmol,) and the arene (1.0 mmol) in 5 mL of CH_2Cl_2 at room temperature with stirring. The reaction mass was stirred at room temperature for 12 h. Then, the reaction mixture was poured into water (30 mL) and additionally 20 mL of saturated aqueous solution of NH_4Cl were added. The reaction product was extracted with CH_2Cl_2 (2 × 20 mL); the organic phases were combined, washed with brine (2 × 20 mL), and dried with Na_2SO_4. The solvent was removed in vacuum. The obtained crude compounds **1p–v** were crystallized from cyclohexane.

General procedure for the synthesis of 1-aryl-4,4,4-trichlorobut-3-en-1-ones 2 from hydroxy ketones 1 according to the literature method [7]. A solution of hydroxy ketone **1** (0.355 mmol) and *p*-toluenesulfonic acid monohydrate (34 mg, 0.177 mmol) in 12 mL of toluene was refluxed with a Dean–Stark trap (to remove water) for 4 h. Then, toluene was removed in vacuum and the residue was dissolved in CH_2Cl_2 (20 mL). The obtained solution was washed with saturated aqueous solution of $NaHCO_3$ (2 × 20 mL), water (2 × 20 mL), and dried with Na_2SO_4. The solvent was removed in vacuum and the reaction product was purified by preparative TLC using hexane/ethyl acetate mixtures as eluent.

General procedure for the synthesis of 3-trichloromethylindan-1-ones 3 by cyclization of hydroxy ketones 1 or enones 2 in TfOH. A solution of compound **1** or **2** (0.26 mmol) in 1 mL of TfOH was stirred at 80 °C for 2–18 h with TLC monitoring of the reaction progress. Then, the reaction mixture was cooled to room temperature and quenched with water (30 mL). The reaction product was extracted with CH_2Cl_2 (2 × 20 mL). The combined organic phases were consecutively washed with water (2 × 20 mL) and saturated aqueous solution of $NaHCO_3$

(20 mL), and dried with Na_2SO_4. The solvent was removed in vacuum and the reaction product was purified by preparative TLC using hexane/ethyl acetate mixtures as eluent.

Procedure for the synthesis of indanone 3n by cyclization of enone 2n in H_2SO_4. A solution of compound **2n** (50 mg, 0.16 mmol) in 1 mL of H_2SO_4 was stirred at room temperature for 3 days with TLC monitoring of the reaction progress. Then, the reaction mixture was cooled to room temperature and quenched with water (30 mL). The reaction product was extracted with CH_2Cl_2 (2 × 20 mL). The combined organic phase was consecutively washed with water (2 × 20 mL) and saturated aqueous solution of $NaHCO_3$ (20 mL), then dried with Na_2SO_4. The solvent was removed in vacuum that gave quantitatively the reaction product **3n**.

4,4,4-Trichloro-3-hydroxy-1-phenylbutan-1-one (1a) [7]. It was obtained from acetophenone (1.0 g, 8.3 mmol) and anhydrous chloral (1.84 g, 12.5 mmol) in 2 days. Yield: 1.56 g (70%). White plates, mp 66-67 °C (lit.: mp 65-66 °C [7]). ^{1}H NMR ($CDCl_3$, 400 MHz): δ 8.03 d (2H, J = 7.4 Hz), 7.65 t (1H, 7.4 Hz), 7.53 t (2H, J = 7.4 Hz), 4.91 ddd (1H, J = 8.9, 4.4, 2.0 Hz), 3.73 d (1H, J = 4.4 Hz), 3.68 dd (1H, J = 17.4, 2.0 Hz), 3.52 dd (1H, J = 17.4, 8.9 Hz). ^{13}C NMR ($CDCl_3$, 101 MHz): δ 197.1, 136.3,133.9,128.8,128.3,102.5, 79.0, 40.7.

4,4,4-Trichloro-3-hydroxy-1-(2-methylphenyl)butan-1-one (1b). It was obtained from 2-methylacetophenone (500 mg, 3.7 mmol) and anhydrous chloral (824 mg, 5.6 mmol) in 2 days. Yield: 295 mg (23%). White prisms, mp 65-67 °C (cyclohexane). ^{1}H NMR ($CDCl_3$, 400 MHz): δ 7.74 d (1H, J = 7.3 Hz), 7.45 t (1H, J = 7.3 Hz), 7.29-7.35 m (2H), 4.85 d (1H, J = 8.7 Hz), 3.71 d (1H, J = 17.1 Hz), 3.61 dd (1H, J = 17.1, 2.1 Hz), 3.43 dd (1H, J = 17.1, 9.0 Hz), 2.57 s (3H). ^{13}C NMR ($CDCl_3$, 101 MHz): δ 200.5, 138.9, 136.9, 132.3, 132.2, 128.9, 125.9, 102.6, 79.3, 43.2, 21.5. HRMS (ESI) m/z: $[M+Na]^+$ Calcd for $C_{11}H_{11}Cl_3O_2Na$ 302.9717; Found 302.9720.

4,4,4-Trichloro-3-hydroxy-1-(4-methylphenyl)butan-1-one (1c) [7]**.** It was obtained from 4-methylacetophenone (500 mg, 3.7 mmol) and anhydrous chloral (824 mg, 5.6 mmol) in 2 days. Yield: 412 mg (31%). Pale yellow prisms, mp 91-93 °C (cyclohexane) (lit.: mp 101-102 °C [7]). ^{1}H NMR ($CDCl_3$, 400 MHz): δ 7.92 d (2H, J = 8.2 Hz), 7.32 d (2H, J = 8.2 Hz), 4.87 d (1H, J = 8.9 Hz), 3.77 s (1H), 3.66 dd (1H, J = 17.3, 2.0 Hz), 3.48 dd (1H, J = 17.3, 9.0 Hz), 2.46 s (3H). ^{13}C NMR ($CDCl_3$, 101 MHz): δ 196.8, 144.9, 133.9, 129.5, 128.4, 102.5, 79.1, 40.5, 21.7.

4,4,4-Trichloro-1-(4-fluorophenyl)-3-hydroxybutan-1-one (1d) [7]. It was obtained from 4-fluoroacetophenone (1.0 g, 7.3 mmol) and anhydrous chloral (1.6 g, 10.9 mmol) in 6 days. Yield: 811 mg (39%). Pale yellow prisms, mp 93-95 °C (cyclohexane) (lit.: mp 94-95 °C [7]). ^{1}H NMR ($CDCl_3$, 400 MHz): δ 8.05 dd (2H, J = 8.6, 5.4 Hz), 7.19 t (2H, J = 8.6 Hz), 4.89 dd (1H, J = 8.8, 2.2 Hz), 3.86 s (1H), 3.62 dd (1H, J = 17.3, 2.2 Hz), 3.50 dd (1H, J = 17.3, 8.8

Hz). ^{13}C NMR ($CDCl_3$, 101 MHz): δ 195.4, 166.2 d (*J* = 256.3 Hz), 132.8 d (*J* = 3.0 Hz), 131.0 d (*J* = 9.5 Hz), 116.0 d (*J* = 22.0 Hz), 102.6, 79.0, 40.7. ^{19}F NMR ($CDCl_3$, 376 MHz): δ -103.6 s (F).

4,4,4-Trichloro-1-(4-chlorophenyl)-3-hydroxybutan-1-one (1e) [7]. It was obtained from 4-chloroacetophenone (1.0 g, 6.5 mmol) and anhydrous chloral (1.4 g, 9.7 mmol) in 2 days. Yield: 703 mg (36%). Pale yellow needles, mp 108-110 °C (cyclohexane) (lit.: mp 118 °C [7]). ^{1}H NMR ($CDCl_3$, 400 MHz): δ 7.96 d (2H, J = 8.3 Hz), 7.49 d (2H, J = 8.3 Hz), 4.88 dd (1H, J = 8.8, 2.1 Hz), 3.77 d (1H, J = 4.6), 3.61 dd (1H, J = 17.2, 2.1 Hz), 3.49 dd (1H, J = 17.2, 8.8 Hz). ^{13}C NMR ($CDCl_3$, 101 MHz): δ 195.7, 140.5, 134.7, 129.7, 129.2, 102.5, 79.0, 40.8.

1-(4-Bromophenyl)-4,4,4-trichloro-3-hydroxybutan-1-one (1f) [7]. It was obtained from 4-bromoacetophenone (1.0 g, 5.0 mmol) and anhydrous chloral (1.11 g, 7.5 mmol) in 6 days. Yield: 783 mg (45%). Pale brown needles, mp 125-127 °C (cyclohexane) (lit.: mp 127 °C [7]). ^{1}H NMR ($CDCl_3$, 400 MHz): δ 7.87 d (2H, J = 8.6 Hz), 7.66 d (2H, J = 8.6 Hz), 4.88 ddd (1H, J = 8.8, 4.4, 2.1 Hz), 3.77 m (1H), 3.6 dd (1H, J = 17.3, 2.1 Hz), 3.49 dd (1H, J = 17.3, 8.8 Hz). ^{13}C NMR ($CDCl_3$, 101 MHz): δ 196.0, 135.1, 132.2, 129.8, 129.2, 102.5, 78.9, 40.7.

4,4,4-Trichloro-1-(3-fluorophenyl)-3-hydroxybutan-1-one (1g). It was obtained from 3-fluoroacetophenone (1.0 g, 7.3 mmol) and anhydrous chloral (1.6 g, 10.9 mmol) in 6 days. Yield: 1.55 g (75%). White prisms, mp 65-67 °C (cyclohexane). ^{1}H NMR ($CDCl_3$, 400 MHz): δ 7.80 dt (1H, J = 7.8, 1.3 Hz), 7.70 ddd (1H, J = 9.4, 2.7, 1.6 Hz), 7.51 td (1H, J = 8.0, 5.5 Hz), 7.35 m (1H), 4.90 dd (1H, J = 8.8, 2.1 Hz), 3.63 dd (2H, J = 17.4, 2.1 Hz), 3.51 dd (1H, J = 17.4, 8.8 Hz). ^{13}C NMR ($CDCl_3$, 101 MHz): δ 195.7, 162.9 d (*J* = 248.7 Hz), 138.4 d (*J* = 6.2 Hz), 130.5 d (*J* = 7.7 Hz), 124.0 d (*J* = 3.0 Hz), 120.9 d (*J* = 21.5 Hz), 115.0 d (*J* = 22.5 Hz), 102.5, 78.9, 41.0. ^{19}F NMR ($CDCl_3$, 376 MHz): δ -111.2 s (F).

4,4,4-Trichloro-1-(2,4-dichlorophenyl)-3-hydroxybutan-1-one (1h). It was obtained from 2,4-dichloroacetophenone (1.0 g, 5.3 mmol) and anhydrous chloral (1.17 g, 7.9 mmol) in 6 days. Yield: 1.28 g (72%). Pale yellow needles, mp 82-84 °C (hexane). ^{1}H NMR ($CDCl_3$, 400 MHz): δ 7.58 d (1H, J = 8.4 Hz), 7.50 d (1H, J = 2.0 Hz), 7.38 dd (1H, J = 8.4, 2.0 Hz), 4.84 dd (1H, J = 9.1, 2.1 Hz), 3.67 dd (1H, J = 17.3, 2.1 Hz), 3.47 dd (2H, J = 17.3, 9.1 Hz). ^{13}C NMR ($CDCl_3$, 101 MHz): δ 198.1, 138.4, 136.4, 132.5, 130.8, 130.7, 127.6, 102.3, 79.1, 45.0. HRMS (ESI) m/z: $[M+Na]^+$ Calcd for $C_{10}H_7Cl_5O_2Na$ 356.8781; Found 356.8782.

4,4,4-Trichloro-1-(4-(trifluoromethyl)phenyl)-3-hydroxybutan-1-one (1i). It was obtained from 4-(trifluoromethyl)acetophenone (300 mg, 1.6 mmol) and anhydrous chloral (360 mg, 2.4 mmol) in 2 days. Yield: 211 mg (38%). White needles, mp 85-87 °C (cyclohexane). ^{1}H NMR ($CDCl_3$, 400 MHz): δ 8.12 d (2H, J = 8.1 Hz), 7.79 d (2H, J = 8.1 Hz), 4.92 ddd (1H, J = 8.5, 4.6, 2.4 Hz), 3.75 d (1H, 4.6 Hz), 3.65 dd (1H, J = 17.5, 2.4 Hz), 3.57 dd

(1H, J = 17.4, 8.5 Hz). ^{13}C NMR ($CDCl_3$, 101 MHz): δ 195.9, 139.0, 135.1 d (*J* = 32.9 Hz), 128.6, 125.9 q (*J* = 3.7 Hz), 123.4 d (*J* = 272.8 Hz), 102.5, 78.9, 41.1. ^{19}F NMR ($CDCl_3$, 376 MHz): δ -63.2 s (CF_3). HRMS (MALDI) m/z: $[M+H]^+$ Calcd for $C_{11}H_9Cl_3F_3O_2$ 316.9509; Found 316.9511.

4,4,4-Trichloro-3-hydroxy-1-(4-methoxyphenyl)butan-1-one (1j) [7]. It was obtained from 4-methoxyacetophenone (1.0 g, 6.6 mmol) and anhydrous chloral (1.47 g, 9.9 mmol) in 6 days. Yield: 921 mg (46%). Pale brown needles, mp 109-111 °C (cyclohexane) (lit.: mp 109-110 °C [7]). ^{1}H NMR ($CDCl_3$, 400 MHz): δ 8.00 d (2H, J = 8.9 Hz), 6.98 d (2H, J = 8.9 Hz), 4.86 d (1H, J = 8.9 Hz), 3.98 s (1H), 3.91 s (3H), 3.63 dd (1H, J = 17.2, 2.1 Hz), 3.45 dd (1H, J = 17.2, 8.9 Hz). ^{13}C NMR ($CDCl_3$, 101 MHz): δ 195.7, 164.2, 130.7, 129.4, 114.0, 102.6, 79.2, 55.6, 40.2.

4,4,4-Trichloro-3-hydroxy-1-(3,4-dimethoxyphenyl)butan-1-one (1k). It was obtained from 3,4-dimethoxyacetophenone (1.0 g, 5.5 mmol) and anhydrous chloral (1.23 g, 8.3 mmol) in 6 days. Yield: 1.472 g (81%). Reddish needles, mp 96-98 °C (cyclohexane). ^{1}H NMR ($CDCl_3$, 400 MHz): δ 7.64 dd (1H, J = 8.5, 2.0 Hz), 7.56 d (1H, J = 2.0), 6.93 d (1H, J = 8.5 Hz), 4.86 ddd (1H, J = 9.1, 4.3, 2.1 Hz), 3.98 s (3H), 3.96 s (3H), 3.93 d (1H, J = 4.3 Hz), 3.62 dd (1H, J = 17.0, 2.1 Hz), 3.46 dd (1H, J = 17.0, 9.1 Hz). ^{13}C NMR ($CDCl_3$, 101 MHz): δ 195.7, 154.1, 149.3, 129.6, 123.3, 110.2, 110.1, 102.6, 79.3, 55.2, 55.1, 40.1. HRMS (MALDI) m/z: $[M+H]^+$ Calcd for $C_{12}H_{14}Cl_3O_4$ 326.9952; Found 326.9949.

4,4,4-Trichloro-3-hydroxy-1-(3-nitrophenyl)butan-1-one (1l). It was obtained from 3-nitroacetophenone (1.0 g, 6.0 mmol) and anhydrous chloral (1.34 g, 9.0 mmol) in 6 days. Yield: 804 mg (42%). Yellow needles, mp 104-106 °C (carbon tetrachloride). ^{1}H NMR ($CDCl_3$, 400 MHz): δ 8.83 t (1H, J = 2.0 Hz), 8.49 ddd (1H, J = 8.2, 2.3, 1.1 Hz), 8.35 dt (1H, J = 7.8, 1.4 Hz), 7.76 t (1H, J = 8.0 Hz), 4.93 dt (1H, J = 8.0, 2.8 Hz), 3.44 – 3.71 m (3H). ^{13}C NMR ($CDCl_3$, 101 MHz): δ 194.5, 148.6, 137.7, 133.7, 130.2, 128.0, 123.2, 102.4, 78.9, 41.1. HRMS (MALDI) m/z: $[M+H]^+$ Calcd for $C_{10}H_9Cl_3NO_4$ 311.9591; Found 311.9593.

4,4,4-Trichloro-3-hydroxy-1-(4-nitrophenyl)butan-1-one (1m) [7]. It was obtained from 4-nitroacetophenone (1.0 g, 6.0 mmol) and anhydrous chloral (1.34 g, 9.0 mmol) in 6 days. Yield: 479 mg (25%). Yellow needles, mp 115-117 °C (carbon tetrachloride) (lit.: mp 115-116 °C [7]). ^{1}H NMR ($CDCl_3$, 400 MHz): δ 8.36 d (2H, J = 8.7 Hz), 8.18 d (2H, J = 8.7 Hz), 4.92 dt (1H, J = 7.2, 3.3 Hz), 3.54 – 3.69 m (3H). ^{13}C NMR ($CDCl_3$, 101 MHz): δ 195.2, 150.7, 140.7, 129.4, 124.0, 102.4, 78.9, 41.4.

4,4,4-Trichloro-3-hydroxy-1-(naphthalen-2-yl)butan-1-one (1n) [7]. It was obtained from 1-(naphthalen-2-yl)ethanone (1.0 g, 5.9 mmol) and anhydrous chloral (1.3 g, 8.8 mmol) in 6 days. Yield: 597 mg (32%). White needles, mp 133-135 °C (ethylacetate) (lit.: mp 133-134 °C

[7]). ^{1}H NMR ($CDCl_3$, 400 MHz): δ 8.52 s (1H), 8.06 dd (1H, J = 8.6, 1.8 Hz), 8.01 d (1H, J = 8.0), 7.92 t (2H, J = 8.6 Hz), 7.56-7.70 m (2H), 4.97 dd (1H, J = 8.8, 2.1 Hz), 4.00 s (1H), 3.79 dd (1H, J = 17.3, 2.1 Hz), 3.67 dd (1H, J = 17.3, 8.8 Hz). ^{13}C NMR ($CDCl_3$, 101 MHz): δ 197.1, 135.9, 133.7, 132.4, 130.3, 129.7, 129.0, 128.7, 127.8, 127.0, 123.6, 102.7, 79.2, 40.8.

1-(4-Phenylphenyl)-4,4,4-trichloro-3-hydroxybutan-1-one (1o) [7]. It was obtained from 4-phenylacetophenone (2.0 g, 10.2 mmol) and anhydrous chloral (2.25 g, 15.3 mmol) in 6 days. Yield: 2.58 g (72%). White prisms, mp 118-119 °C (lit.: mp 117-119 °C [7]). ^{1}H NMR ($CDCl_3$, 400 MHz): δ 8.10 d (2H, *J* = 8.5 Hz), 7.75 d (2H, *J* = 8.5 Hz), 7.66 d (2H, *J* = 7.3 Hz), 7.51 t (2H, *J* = 7.4 Hz), 7.45 t (1H, *J* = 7.3 Hz), 4.92 ddd (1H, *J* = 8.9, 4.4, 2.1 Hz), 3.77 d (1H, *J* = 4.4 Hz), 3.70 dd (1H, *J* = 17.3, 2.1 Hz).), 3.55 dd (1H, *J* = 17.3, 8.9 Hz). ^{13}C NMR ($CDCl_3$, 101 MHz): δ 196.7, 146.6, 139.6, 135.0, 129.0, 128.9, 128.5, 127.4, 127.3, 102.5, 79.1, 40.7.

4,4,4-Trichloro-3-hydroxy-1-(3,4-dimethylphenyl)butan-1-one (1p). It was obtained from *o*-xylene (112 mg, 1.0 mmol), 4-(trichloromethyl)oxetan-2-one (200 mg, 1.0 mmol) and aluminium chloride (528 mg, 4.0 mmol) at room temperature in 12 h. Yield: 81 mg (26%). White needles, mp 110-111 °C (cyclohexane). ^{1}H NMR ($CDCl_3$, 400 MHz): δ 7.78 s (1H), 7.75 d (1H, J = 7.8 Hz), 7.27 d (1H, J = 7.8 Hz), 4.87 ddd (1H, J = 9.0, 4.3, 2.1 Hz), 3.80 d (1H, J = 4.3 Hz), 3.65 dd (1H, J = 17.3, 2.1 Hz), 3.47 dd (1H, J = 17.3, 9.0 Hz), 2.36 s (6H). ^{13}C NMR ($CDCl_3$, 101 MHz): δ 197.1, 143.7, 137.3, 134.3, 130.0, 129.3, 126.0, 102.5, 79.2, 40.5, 20.1, 19.8. HRMS (ESI) m/z: $[M+Na]^+$ Calcd for $C_{12}H_{14}Cl_3O_2Na$ 316.9873; Found 316.9875.

4,4,4-Trichloro-3-hydroxy-1-(2,4-dimethylphenyl)butan-1-one (1q) [7]. It was obtained from *m*-xylene (112 mg, 1.0 mmol), 4-(trichloromethyl)oxetan-2-one (200 mg, 1.0 mmol) and aluminium chloride (528 mg, 4.0 mmol) at room temperature in 12 h. Yield: 188 mg (60%). White plates, mp 94-96 °C (cyclohexane) (lit.: mp 99-100 °C [7]). ^{1}H NMR ($CDCl_3$, 400 MHz): δ 7.68 d (1H, J = 8.3 Hz), 7.13 d (2H, J = 7.6 Hz), 4.83 ddd (1H, J = 9.0, 4.4, 2.1 Hz), 3.76 d (1H, J = 4.4 Hz), 3.62 dd (1H, J = 17.0, 2.1 Hz), 3.41 dd (1H, J = 17.0, 9.0 Hz), 2.56 s (3H), 2.40 s (3H). ^{13}C NMR ($CDCl_3$, 199.8, 143.1, 139.5, 133.9, 133.2, 129.5, 126.6, 102.6, 79.4, 42.8, 21.7, 21.4.

4,4,4-Trichloro-3-hydroxy-1-(2,5-dimethylphenyl)butan-1-one (1r). It was obtained from *p*-xylene (113 mg, 1.0 mmol), 4-(trichloromethyl)oxetan-2-one (200 mg, 1.0 mmol) and aluminium chloride (528 mg, 4.0 mmol) at room temperature in 12 h. Yield: 117 mg (38%). White plates, mp 89-91 °C (cyclohexane). ^{1}H NMR ($CDCl_3$, 400 MHz): δ 7.51 s (1H), 7.26 d (1H, J = 7.8 Hz), 7.19 d (1H, J = 7.8 Hz), 4.85 ddd (1H, J = 9.0, 4.3, 2.0 Hz), 3.71 d (1H, J = 4.3 Hz), 3.60 dd (1H, J = 17.2, 2.0 Hz), 3.41 dd (1H, J = 17.2, 9.0 Hz), 2.51 s (3H), 2.41 s (3H). ^{13}C NMR ($CDCl_3$, 101 MHz): δ 200.7, 136.8, 135.7, 135.5, 132.9, 132.2, 129.3, 102.6, 79.3, 43.2, 21.0, 20.9. HRMS (ESI) m/z: $[M+Na]^+$ Calcd for $C_{12}H_{14}Cl_3O_2Na$ 316.9873; Found 316.9871.

4,4,4-Trichloro-3-hydroxy-1-(1,2,4-trimethylphenyl)butan-1-one (1s). It was obtained from pseudocumene (127 mg, 1.0 mmol), 4-(trichloromethyl)oxetan-2-one (200 mg, 1.0 mmol) and aluminium chloride (528 mg, 4.0 mmol) at room temperature in 12 h. Yield: 193 mg (59%). Pale green needles, mp 123-125 °C (cyclohexane). ^{1}H NMR ($CDCl_3$, 400 MHz): δ 7.51 s (1H), 7.07 s (1H), 4.83 ddd (1H, *J* = 9.1, 4.4, 2.1 Hz, -CH-), 3.80 d (1H, *J* = 4.4 Hz), 3.61 dd (1H, J = 17.1, 2.1 Hz), 3.41 dd (1H, J = 17.1, 9.0 Hz), 2.51 s (3H), 2.32 d (6H, *J* = 8.0 Hz). ^{13}C NMR ($CDCl_3$, 101 MHz): δ 200.0, 141.8, 136.7, 134.0 d (*J* = 10.3 Hz), 133.7, 130.5, 102.6, 79.4, 42.8, 21.2, 19.8, 19.3. HRMS (ESI) m/z: $[M+Na]^+$ Calcd for $C_{13}H_{15}Cl_3O_2Na$ 331.0030; Found 331.0032.

4,4,4-Trichloro-3-hydroxy-1-(2,4,6-trimethylphenyl)butan-1-one (1t). It was obtained from mesitylene (127 mg, 1.0 mmol), 4-(trichloromethyl)oxetan-2-one (200 mg, 1.0 mmol) and aluminium chloride (528 mg, 4.0 mmol) at room temperature in 12 h. Yield: 113 mg (35%). Pale green needles, mp 98-100 °C (cyclohexane). ^{1}H NMR ($CDCl_3$, 400 MHz): δ 6.89 s (2H), 4.88 ddd (1H, J = 9.2, 4.4, 1.5 Hz), 3.58 m (1H), 3.41 d (1H, J = 18.2 Hz), 3.20 dd (1H, J = 18.2, 9.2 Hz), 2.32 s (3H), 2.27 s (6H). ^{13}C NMR ($CDCl_3$, 101 MHz): δ 207.1, 139.1, 138.4, 132.8, 128.7,102.5, 78.6, 46.9, 21.1, 19.1. HRMS (ESI) m/z: $[M+Na]^+$ Calcd for $C_{13}H_{15}Cl_3O_2Na$ 331.0030; Found 331.0030.

4,4,4-Trichloro-3-hydroxy-1-(2,3,5,6-tetramethylphenyl)butan-1-one (1u). It was obtained from durene (142 mg, 1.0 mmol), 4-(trichloromethyl)oxetan-2-one (200 mg, 1.0 mmol) and aluminium chloride (528 mg, 4.0 mmol) at room temperature in 12 h. Yield: 200 mg (57%). Pale yellow needles, mp 108-110 °C (cyclohexane). ^{1}H NMR ($CDCl_3$, 400 MHz): δ 7.02 s (1H), 4.92 d (1H, J = 9.0 Hz), 3.58 s (1H), 3.40 d (1H, J = 18.6 Hz), 3.41 dd (1H, J = 18.6, 9.0 Hz), 2.24 s (6H), 2.14 s (6H). ^{13}C NMR ($CDCl_3$, 101 MHz): δ 208.1, 141.5, 134.7, 132.2, 128.1, 102.5, 78.3, 47.4, 19.4, 15.9. HRMS (ESI) m/z: $[M+Na]^+$ Calcd for $C_{14}H_{17}Cl_3O_2Na$ 345.0186; Found 345.0188.

1-(4-*tert*-Butylphenyl)-4,4,4-trichloro-3-hydroxybutan-1-one (1v). It was obtained from *tert*-butylbenzene (142 mg, 1.0 mmol), 4-(trichloromethyl)oxetan-2-one (200 mg, 1.0 mmol) and aluminium chloride (528 mg, 4.0 mmol) at room temperature in 12 h. Yield: 73 mg (21%). White prisms, mp 116-118 °C (cyclohexane). ^{1}H NMR ($CDCl_3$, 400 MHz): δ 7.96 d (2H, J = 8.5 Hz), 7.54 d (2H, J = 8.5 Hz), 4.88 ddd (1H, J = 8.9, 4.2, 2.0 Hz), 3.83 m, 3.66 dd (1H, J = 17.3, 2.0 Hz), 3.49 dd (1H, J = 17.3, 8.9 Hz), 1.38 s (9H). ^{13}C NMR ($CDCl_3$, 101 MHz): δ 196.9, 157.9, 133.8, 128.3, 125.8, 102.6, 79.1, 40.5, 35.2, 31.0. HRMS (ESI) m/z: $[M+Na]^+$ Calcd for $C_{14}H_{17}Cl_3O_2Na$ 345.0186; Found 345.0187.

(*E*)-4,4,4-Trichloro-1-phenylbut-2-en-1-one (2a) [7]. It was obtained from 4,4,4-trichloro-3-hydroxy-1-phenylbutan-1-one (**1a**, 500 mg, 1.87 mmol) and *p*-toluenesulfonic acid

hydrate (180 mg, 0.95 mmol) in 4 h. Yield: 438 mg (94%). White plates, mp 101-102 °C (hexane) (lit.: mp 100 °C [7]). ^{1}H NMR ($CDCl_3$, 400 MHz): δ 7.99 d (2H, *J* 7.33 Hz), 7.64 t (1H, *J* 7.4 Hz), 7.53 t (2H, *J* 7.6 Hz), 7.42 d (1H, *J* 14.6 Hz), 7.27 d (1H, *J* 14.6 Hz). ^{13}C NMR ($CDCl_3$, 101 MHz): δ 189.0, 145.6, 137.0, 134.0, 129.1, 128.9, 124.3, 93.1.

(*E*)-4,4,4-Trichloro-1-(2-methylphenyl)but-2-en-1-one (2b). It was obtained from 4,4,4-trichloro-3-hydroxy-1-(2-methylphenyl)butan-1-one **(1b**, 100 mg, 0.35 mmol) and *p*-toluenesulfonic acid monohydrate (34 mg, 0.18 mmol) in 4 h. Yield: 92 mg (99%). Reddish oil. ^{1}H NMR ($CDCl_3$, 400 MHz): δ 7.60 dd (1H, J = 7.6, 1.7 Hz), 7.46 td (1H, J = 7.5, 1.5 Hz), 7.34 t (2H, J = 7.7 Hz), 7.14 d (1H, J = 14.8 Hz), 7.08 d (1H, J = 14.8 Hz), 2.53 s (3H). ^{13}C NMR ($CDCl_3$, 101 MHz): δ193.2, 145.6, 138.4, 137.2, 131.9, 129.0, 127.7, 125.8, 92.8. HRMS (MALDI) m/z: $[M+H]^+$ Calcd for $C_{11}H_{10}Cl_3O$ 262.9791; Found 262.9786.

(*E*,*Z*-)-4,4,4-Trichloro-1-(4-methylphenyl)but-2-en-1-one (2c) [7] was obtained from 4,4,4-trichloro-3-hydroxy-1-(4-methylphenyl)butan-1-one **(1b**, 100 mg, 0.35 mmol) and *p*-toluenesulfonic acid monohydrate (34 mg, 0.18 mmol) in 4 h in total yield: 89 mg (94%) with an *E/Z*-ratio of 5:1. Colorless oil. *E*-isomer: ^{1}H NMR ($CDCl_3$, 400 MHz): δ (from spectrum of isomer mixture) 7.92 d (2H, J = 8.1 Hz), 7.43 d (1H, J = 14.6 Hz), 7.34 d (2H, J = 8.1 Hz), 7.28 d (1H, J = 14.6 Hz), 2.47 s (3H). *Z*-isomer: ^{1}H NMR ($CDCl_3$, 400 MHz): δ (from spectrum of isomer mixture) 7.92 d (2H, J = 8.1 Hz), 7.34 d (2H, J = 8.1 Hz), 6.51 d (1H, J = 9.6 Hz), 5.91 d (1H, J = 9.6 Hz), 2.47 s (3H). *E*-isomer: ^{13}C NMR ($CDCl_3$, 101 MHz): δ (selected signals from spectrum of isomer mixture) 188.3 (C=O), 93.1 (CCl_3); *Z*-isomer: ^{13}C NMR ($CDCl_3$, 101 MHz): δ (selected signals from spectrum of isomer mixture) 189.3 (C=O), 86.7 (CCl_3). For mixture of *E-/Z*-isomers: ^{13}C NMR ($CDCl_3$, 101 MHz): δ 145.2, 145.0, 134.4, 129.7, 129.6, 129.2, 128.9, 128.4, 124.5, 124.2, 21.8.

(*E*)-4,4,4-Trichloro-1-(4-fluorophenyl)but-2-en-1-one (2d) [7]. It was obtained from 4,4,4-trichloro-1-(4-fluorophenyl)-3-hydroxybutan-1-one **(1d**, 500 mg, 1.75 mmol) and *p*-toluenesulfonic acid monohydrate (166 mg, 0.88 mmol) in 4 h. Yield: 407 mg (87%). Light brown plates, mp 116-118 °C (hexane) (lit.: mp 117-119 °C [7]). ^{1}H NMR ($CDCl_3$, 400 MHz): δ 7.99 – 8.08 m (2H), 7.38 d (1H, *J* = 14.6 Hz), 7.26 d (1H, *J* = 14.5 Hz), 7.19 t (2H, *J* = 8.6 Hz). ^{13}C NMR ($CDCl_3$, 101 MHz): δ 187.3, 166.4 d (*J* = 256.6 Hz), 146.8, 133.4 d (*J* = 3.0 Hz), 131.6 d (*J* = 9.6 Hz), 123.9, 116.3 d (*J* = 22.0 Hz), 93.0. ^{19}F NMR ($CDCl_3$, 376 MHz): δ -103.2 s (F).

(*E*)-4,4,4-Trichloro-1-(4-chlorophenyl)but-2-en-1-one (2e) [7]. It was obtained from 4,4,4-trichloro-1-(4-chlorophenyl)-3-hydroxybutan-1-one **(1e**, 500 mg, 1.65 mmol) and *p*-toluenesulfonic acid monohydrate (157 mg, 0.83 mmol) in 4 h. Yield: 423 mg (90%). Pale yellow plates, mp 114-116 °C (hexane) (lit.: mp 115-116 °C [7]). ^{1}H NMR ($CDCl_3$, 400 MHz): δ

7.88 – 7.95 m (2H), 7.46 – 7.53 m (2H), 7.36 d (1H, J = 14.6 Hz), 7.27 d (1H, J = 14.6 Hz). ^{13}C NMR ($CDCl_3$, 101 MHz): δ 187.7, 146.0, 140.6, 135.2, 130.3, 129.4, 123.7, 92.9.

(*E*)-1-(4-Bromophenyl)-4,4,4-trichlorobut-2-en-1-one (2f) [7]. It was obtained from 1-(4-bromophenyl)-4,4,4-trichloro-3-hydroxybutan-1-one **(1f**, 118 mg, 0.34 mmol) and *p*-toluenesulfonic acid monohydrate (34 mg, 0.18 mmol) in 4 h. Yield: 106 mg (95%). Reddish needles, mp 119-121 °C (ethanol) (lit.: mp 122 °C [7]). ^{1}H NMR ($CDCl_3$, 400 MHz): δ 7.88 d (2H, J = 8.6 Hz), 7.69 d (2H, J = 8.6 Hz), 7.38 d (1H, J = 14.6 Hz), 7.30 d (2H, J = 14.6 Hz). ^{13}C NMR ($CDCl_3$, 101 MHz): δ 187.8, 145.9, 135.5, 132.3, 130.2, 129.3, 123.5, 92.8.

(*E*)-4,4,4-Trichloro-1-(3-fluorophenyl)but-2-en-1-one (2g). It was obtained from 4,4,4-trichloro-1-(3-fluorophenyl)-3-hydroxybutan-1-one **(1g**, 104 mg, 0.36 mmol) and *p*-toluenesulfonic acid monohydrate (34 mg, 0.18 mmol) in 4 h. Yield: 88 mg (91%). Reddish oil. ^{1}H NMR ($CDCl_3$, 400 MHz): δ 7.79 dt (1H, J = 7.8, 1.3 Hz), 7.69 dt (1H, J = 9.2, 2.1 Hz), 7.54 td (1H, J = 8.0, 5.4 Hz), 7.38 d (2H, J = 14.6 Hz), 7.30 d (1H, J = 14.6 Hz). ^{13}C NMR ($CDCl_3$, 101 MHz): δ 187.6 d (*J* = 2.3 Hz), 162.9 d (*J* = 249.0 Hz), 146.0, 138.8 d (*J* = 6.4 Hz), 130.7 d (*J* = 7.7 Hz), 124.5 d (*J* = 3.0 Hz), 123.6, 121.0 d (*J* = 21.5 Hz), 115.5 d (*J* = 2 2.5 Hz), 92.7. ^{19}F NMR ($CDCl_3$, 376 MHz): δ -111.2 s (F). HRMS (MALDI) m/z: $[M+H]^+$ Calcd for $C_{10}H_7Cl_3FO$ 266.9541; Found 266.9542.

(*E*)-4,4,4-Trichloro-1-(2,4-dichlorophenyl)but-2-en-1-one (2h). It was obtained 4,4,4-trichloro-1-(2,4-dichlorophenyl)-3-hydroxybutan-1-one **(1h**, 140 mg, 0.42 mmol) and *p*-toluenesulfonic acid monohydrate (41 mg, 0.21 mmol) in 4 h. Yield: 120 mg (91%). Reddish oil. ^{1}H NMR ($CDCl_3$, 400 MHz): δ 7.50 – 7.57 m (2H), 7.41 dd (1H, J = 8.3, 2.0 Hz), 7.14 d (1H, J = 14.8 Hz), 7.08 d (1H, J = 14.8 Hz). ^{13}C NMR ($CDCl_3$, 101 MHz): δ 190.3, 145.6, 138.6, 136.0, 133.0, 131.1, 130.6, 127.7, 127.2, 92.4. HRMS (MALDI) m/z: $[M+H]^+$ Calcd for $C_{10}H_7Cl_5O_2$ 316.8855; Found 316.8856.

(*E*,*Z*-)-4,4,4-Trichloro-1-((4-trifluoromethyl)phenyl)but-2-en-1-one (2i) was obtained from 4,4,4-trichloro-1-(4-(trifluoromethyl)phenyl)-3-hydroxybutan-1-one **(1i**, 100 mg, 0.35 mmol) and *p*-toluenesulfonic acid monohydrate (34 mg, 0.18 mmol) in 4 h in a total yield of 89 mg (94%) with an *E/Z*-ratio of 3:1. Colorless oil. *E*-isomer: ^{1}H NMR ($CDCl_3$, 400 MHz): δ (from spectrum of isomer mixture) 8.11 d (2H, J = 8.4 Hz), 7.81 d (2H, J = 8.4 Hz), 7.41 d (1H, J = 14.6 Hz), 7.32 d (1H, J = 14.6 Hz). *Z*-isomer: ^{1}H NMR ($CDCl_3$, 400 MHz): δ (from spectrum of isomer mixture) 8.11 d (2H, J = 8.4 Hz), 7.80 d (2H, J = 8.4 Hz), 6.62 d (1H, J = 12.4 Hz), 6.32 d (1H, J = 12.4 Hz). *E*-isomer: ^{13}C NMR ($CDCl_3$, 101 MHz): δ (selected signals from spectrum of isomer mixture) 188.1 (C=O), 92.6 (CCl_3). *Z*-isomer: ^{13}C NMR ($CDCl_3$, 101 MHz): δ (selected signals from spectrum of isomer mixture) 192.1 (C=O), 91.2 (CCl_3). *E*-isomer: ^{19}F NMR ($CDCl_3$, 376 MHz): δ (selected signals from spectrum of isomer mixture) -63.23. *Z*-

isomer: ^{19}F NMR ($CDCl_3$, 376 MHz): δ (selected signals from spectrum of isomer mixture) -63.22. For mixture of *E-/Z*-isomers: ^{13}C NMR ($CDCl_3$, 101 MHz): δ 146.4, 135.0 q (1C, J = 32.7 Hz), 129.4, 129.0, 126.0 q (1C, J = 3.9 Hz), 123.5, 123.4, q (1C, J = 273 Hz). HRMS (ESI) m/z: $[M+Na]^+$ Calcd for $C_{11}H_6Cl_3F_3O_2$ $[M+Na]^+$ 338.9329; Found 338.9320.

(*E*)-4,4,4-Trichloro-1-(4-methoxyphenyl)but-2-en-1-one (2j) [7]. It was obtained 4,4,4-trichloro-3-hydroxy-1-(4-methoxyphenyl)butan-1-one **(1j**, 500 mg, 1.68 mmol) and *p*-toluenesulfonic acid monohydrate (160 mg, 0.84 mmol) in 4 h. Yield: 376 mg (80%). Pale yellow prisms, mp 67-69 °C (ethanol) (lit.: mp 66-68 °C [7]). ^{1}H NMR ($CDCl_3$, 400 MHz): δ 7.98 d (2H, J = 8.9 Hz), 7.40 d (1H, J = 14.5 Hz), 7.24 d (1H, J = 14.5 Hz), 6.98 d (1H, J = 8.9 Hz). ^{13}C NMR ($CDCl_3$, 101 MHz): δ 187.7, 164.4, 144.9, 131.3, 130.0, 124.3, 114.3, 93.3, 55.7.

(*E*)-4,4,4-Trichloro-1-(3-nitrophenyl)but-2-en-1-one (2l). It was obtained from 4,4,4-trichloro-3-hydroxy-1-(3-nitrophenyl)butan-1-one **(1l**, 335 mg, 1.07 mmol) and *p*-toluenesulfonic acid monohydrate (113 mg, 0.54 mmol) in 4 h. Yield: 253 mg (83%). Pale yellow needles, mp 92-95 °C. ^{1}H NMR ($CDCl_3$, 400 MHz): δ 8.83 t (1H, J = 2.0 Hz), 8.51 ddd (1H, J = 8.2, 2.3, 1.1 Hz), 8.34 dt (1H, J = 7.8, 1.4 Hz), 7.78 t (1H, J = 8.0 Hz), 7.45 d (1H, J = 14.5 Hz), 7.37 d (1H, J = 14.5 Hz). ^{13}C NMR ($CDCl_3$, 101 MHz): δ 186.7, 148.6, 147.0, 138.0, 134.1, 130.3, 128.0, 123.5, 122.8, 92.4. HRMS (MALDI) m/z: $[M+H]^+$ Calcd for $C_{10}H_6Cl_3NO_3$ 293.9486; Found 293.9485.

(*E*,*Z*-)-4,4,4-Trichloro-1-(4-nitrophenyl)but-2-en-1-one (2m) [7] was obtained from 4,4,4-trichloro-1-(4-nitrophenyl)-3-hydroxybutan-1-one **(1m**, 77 mg, 0.25 mmol) and *p*-toluenesulfonic acid monohydrate (23 mg, 0.12 mmol) in 4 h in a total yield of 59 mg (82%) with an *E*/*Z*-ratio of 6.6:1. Yellow needles, mp 101-103 °C (lit.: mp 109 °C [7]). *E*-isomer: ^{1}H NMR ($CDCl_3$, 400 MHz): δ (from spectrum of isomer mixture) 8.40 d (2H, J = 8.8 Hz), 8.17 d (2H, J = 8.8 Hz), 7.42 d (1H, J = 14.6 Hz), 7.35 d (1H, J = 14.6 Hz). *Z*-isomer: ^{1}H NMR ($CDCl_3$, 400 MHz): δ (from spectrum of isomer mixture) 8.40 d (2H, J = 8.8 Hz), 8.17 d (2H, J = 8.8 Hz), 6.65 d (1H, J = 12.4 Hz), 6.33 d (1H, J = 12.4 Hz). *E*-isomer: ^{13}C NMR ($CDCl_3$, 101 MHz): δ (selected signals from spectrum of isomer mixture) 187.5 (C=O), 92.4 (CCl_3). *Z*-isomer: ^{13}C NMR ($CDCl_3$, 101 MHz): δ (selected signals from spectrum of isomer mixture) 191.5 (C=O), 91.1 (CCl_3). For mixture of *E-/Z*-isomers: ^{13}C NMR ($CDCl_3$, 101 MHz): δ 150.7, 146.9, 141.2, 140.3, 138.9, 130.0, 129.7, 127.1, 124.1, 123.2.

(*E*)-4,4,4-Trichloro-1-(naphthalene-2-yl)but-2-en-1-one (2n) [7]. It was obtained from 4,4,4-trichloro-3-hydroxy-1-(naphthalen-2-yl)butan-1-one **(1n**, 500 mg, 1.57 mmol) and *p*-toluenesulfonic acid monohydrate (150 mg, 0.79 mmol) in 4 h. Yield: 410 mg (87%). White plates, mp 98-100 °C (lit.: mp 98-99 °C [7]). ^{1}H NMR ($CDCl_3$, 400 MHz): δ 8.48 d (1H, J = 1.7 Hz), 8.05 dd (1H, J = 8.6, 1.8 Hz), 8.00 d (1H, J = 8.0 Hz), 7.94 d (1H, J = 8.6 Hz), 7.90 d (1H, J

= 8.2 Hz), 7.64 ddd (1H, J = 8.2, 6.9, 1.5 Hz), 7.57-7.61 m (1H), 7.57 d (1H, J = 14.5 Hz), 7.35 d (1H, J = 14.5 Hz). ^{13}C NMR ($CDCl_3$, 101 MHz): δ 188.1, 145.6, 136.0, 134.3, 132.6, 130.9, 129.9, 129.2, 129.1, 128.0, 127.2, 124.2, 124.2, 93.2.

(*E*)-1-(4-Phenylphenyl)-4,4,4-trichlorobut-2-en-1-one (2o) [7]. It was obtained from 1-(biphenyl-4-yl)-4,4,4-trichloro-3-hydroxybutan-1-one (**1o**, 500 mg, 1.46 mmol) and *p*-toluenesulfonic acid monohydrate (140 mg, 0.73 mmol) in 4 h. Yield: 441 mg (93%). Pale yellow plates, mp 119-121°C (lit.: mp 120-121 °C [7]). ^{1}H NMR ($CDCl_3$, 400 MHz): δ 8.07 d (2H, *J* = 8.5 Hz), 7.75 d (2H, *J* = 8.5 Hz), 7.64-7.66 m (2H), 7.47-7.51 m (3H), 7.47 s (1H), 7.31 d (1H, *J* = 14.5 Hz). ^{13}C NMR ($CDCl_3$, 101 MHz): δ 188.4, 146.8, 145.5, 139.7, 135.6, 129.5, 129.2, 128.6, 127.7, 127.4, 124.2, 93.1.

(*E*)-4,4,4-Trichloro-1-(2,4,6-trimethylphenyl)but-2-en-1-one (2t). It was obtained from 4,4,4-trichloro-3-hydroxy-1-(2,4,6-trimethylphenyl)butan-1-one (**1t**, 96 mg, 0.31 mmol) and *p*-toluenesulfonic acid monohydrate (34 mg, 0.18 mmol) in 4 h. Yield: 89 mg (98%). Reddish oil. ^{1}H NMR ($CDCl_3$, 400 MHz): δ 6.92 s (2H), 6.79 m (2H), 2.34 s (3H), 2.21 (6H). ^{13}C NMR ($CDCl_3$, 101 MHz): δ 199.4, 146.9, 139.5, 135.9, 134.3, 129.5, 128.8, 92.5, 21.2, 19.4. HRMS (ESI) m/z: $[M+H]^+$ Calcd for $C_{13}H_{14}Cl_3O$ 291.0104; Found 291.0105.

(*E*)-4,4,4-Trichloro-1-(2,3,5,6-tetramethylphenyl)but-2-en-1-one (2u). It was obtained 4,4,4-trichloro-3-hydroxy-1-(2,3,5,6-tetramethylphenyl)butan-1-one (**1u**, 109 mg, 0.34 mmol) and *p*-toluenesulfonic acid monohydrate (34 mg, 0.18 mmol) in 4 h. Yield: 91 mg (88%). Yellow plates, mp 118-120 °C. ^{1}H NMR ($CDCl_3$, 400 MHz): δ 7.06 s (1H), 6.81 d (1H, J = 15.0 Hz), 6.74 d (1H, J = 15.0 Hz), 2.27 s (6H), 2.08 (6H). ^{13}C NMR ($CDCl_3$, 101 MHz): δ 200.7, 147.3, 139.1, 134.6, 132.6, 129.7, 129.6, 92.5, 19.5, 16.2. HRMS (MALDI) m/z: $[M+H]^+$ Calcd for $C_{14}H_{16}Cl_3O$ 305.0261; Found 305.0260.

(*E*)-1-(4-*tert*-Butylphenyl)-4,4,4-trichlorobut-2-en-1-one (2v). It was obtained from 1-(4-*tert*-butylphenyl)-4,4,4-trichloro-3-hydroxybutan-1-one (**1v**, 90 mg, 0.28 mmol) and *p*-toluenesulfonic acid monohydrate (34 mg, 0.18 mmol) in 4 h. Yield: 80 mg (80%). Yellowish oil. ^{1}H NMR ($CDCl_3$, 400 MHz): δ7.96 d (2H, J = 8.5 Hz), 7.56 d (2H, J = 8.5 Hz), 7.43 d (1H, J = 14.7 Hz), 7.28 d (1H, J = 14.7 Hz), 1.39 s (9H). ^{13}C NMR ($CDCl_3$, 101 MHz): δ 188.4, 157.9, 145.1, 134.3, 128.8, 125.9, 124.3, 93.1, 35.3, 31.0. HRMS (MALDI) m/z: $[M+H]^+$ Calcd for $C_{14}H_{16}Cl_3O$ 305.0261; Found 305.0263.

3-(Trichloromethyl)indan-1-one (3a). It was obtained from (*E*)-4,4,4-trichloro-1-phenylbut-2-en-1-one (**2a**, 34 mg, 0.14 mmol) in 2 h. Yield: 30 mg (88%). In the same way, it was obtained from hydroxy ketone **1a** in 3 h in 82% yield. Pale yellow solid, mp 72-74°C. ^{1}H NMR ($CDCl_3$, 400 MHz): δ 8.11 d (1H, J = 7.6 Hz), 7.85 d (1H, J = 7.6 Hz), 7.70 td (1H, J = 7.6, 1.3 Hz), 7.57 t (1H, J = 7.6 Hz), 4.63 dd (1H, J = 6.5, 4.1 Hz), 3.03–3.13 (2H). ^{13}C NMR ($CDCl_3$,

101 MHz): δ 201.9, 149.5, 138.6, 134.6, 129.9, 128.0, 123.9, 101.7, 58.9, 42.8. HRMS (ESI) m/z: $[M+H]^+$ Calcd for $C_{10}H_8Cl_3O$ 248.9635; Found 248.9635.

7-Methyl-3-(trichloromethyl)indan-1-one (3b). It was obtained from 4,4,4-trichloro-3-hydroxy-1-(2-methylphenyl)butan-1-one **(1b**, 50 mg, 0.18 mmol) in 3 h. Yield: 33 mg (70%). Colorless oil. ^{1}H NMR ($CDCl_3$, 400 MHz): δ 7.92 d (1H, J = 7.7 Hz), 7.54 t (1H, J = 7.7 Hz), 7.31 d (1H, J = 7.7 Hz), 4.55 dd (1H, J = 6.6, 4.2 Hz), 3.04 m (2H), 2.69 s (3H). ^{13}C NMR ($CDCl_3$, 101 MHz): δ 202.9, 150.1, 139.1, 135.8, 133.8, 131.8, 125.4, 102.0, 58.3, 43.3, 18.4. HRMS (ESI) m/z: $[M+H]^+$ Calcd for $C_{11}H_{10}Cl_3O$ 262.9791; Found 262.9792.

5-Methyl-3-(trichloromethyl)indan-1-one (3c). It was obtained from 4,4,4-trichloro-3-hydroxy-1-(4-methylphenyl)butan-1-one **(1c**, 73 mg, 0.26 mmol) in 18 h. Yield: 27 mg (40%). Reddish solid, mp 115-117 °C. ^{1}H NMR ($CDCl_3$, 400 MHz): δ 7.89 s, 7.74 d (1H, J = 7.8 Hz), 7.38 d (1H, J = 7.8 Hz), 4.57 dd (1H, J = 6.8, 3.9 Hz), 3.06 m (2H), 2.53 s (3H). ^{13}C NMR ($CDCl_3$, 101 MHz): δ 201.5, 149.9, 146.0, 136.3, 131.0, 128.3, 123.7, 101.8, 58.8, 43.0, 22.4.. HRMS (ESI) m/z: $[M+H]^+$ Calcd for $C_{11}H_{10}Cl_3O$ 262.9791; Found 262.9792.

5-Fluoro-3-(trichloromethyl)indan-1-one (3d). It was obtained from (*E*)-4,4,4-trichloro-1-(4-fluorophenyl)but-2-en-1-one **(2d**, 34 mg, 0.13 mmol) in 10 h. Yield: 11 mg (32%). In the same way, it was obtained from hydroxy ketone **1d** in 15 h in 35% yield. Pale yellow solid, mp 114-116 °C. ^{1}H NMR ($CDCl_3$, 400 MHz): δ 7.86 dd (1H, J = 8.5, 5.4 Hz), 7.79 dd (1H, J = 9.0, 2.2 Hz), 7.24–7.34 m (1H), 4.60 dd (1H, J = 7.0, 3.8 Hz), 3.03–3.16 m (2H). ^{13}C NMR ($CDCl_3$, 101 MHz): δ 199.9, 166.6 d (*J* = 256.8 Hz), 152.0 d (*J* = 10.3 Hz), 135.0, 126.2 d (*J* = 10.4 Hz), 118.1 d (*J* = 23.7 Hz), 115.1 d (*J* = 24.1 Hz), 101.1, 58.5, 42.9. ^{19}F NMR ($CDCl_3$, 376 MHz): δ -101.0. HRMS (ESI) m/z: $[M+H]^+$ Calcd for $C_{10}H_7Cl_3FO$ 266.9541; Found 266.9545.

5-Chloro-3-(trichloromethyl)indan-1-one (3e). It was obtained from (*E*)-4,4,4-trichloro-1-(4-chlorophenyl)but-2-en-1-one **(2e**, 34 mg, 0.12 mmol) in 2 h. Yield: 9 mg (27%). Pale yellow solid, mp 115-117 °C. ^{1}H NMR ($CDCl_3$, 400 MHz): δ 8.10 s (1H), 7.78 d (1H, J = 8.2 Hz), 7.56 dd (1H, J = 8.2, 1.6 Hz), 4.59 dd (1H, J = 6.6, 4.1 Hz), 3.03–3.15 m (2H). ^{13}C NMR ($CDCl_3$, 101 MHz): δ 200.3, 150.8, 141.3, 137.0, 130.6, 128.3, 125.0, 101.1, 58.6, 42.8. HRMS (ESI) m/z: $[M+H]^+$ Calcd for $C_{10}H_7Cl_4O$ 282.9246; Found 282.9243.

5-Bromo-3-(trichloromethyl)indan-1-one (3f). It was obtained from (*E*)-1-(4-bromophenyl)-4,4,4-trichlorobut-2-en-1-one **(2f**, 104 mg, 0.31 mmol) in 8 h. Yield: 35 mg (34%). Pale yellow needles, mp 125-127 °C. ^{1}H NMR ($CDCl_3$, 400 MHz): δ 8.28 s (1H), 7.67 - 7.76 m (2H), 4.60 dd (1H, J = 6.6, 4.1 Hz), 3.00 – 3.15 m (2H). ^{13}C NMR ($CDCl_3$, 101 MHz): δ 200.5, 150.9, 137.4, 133.5, 131.3, 130.0, 125.0, 101.1, 58.5, 42.7. HRMS (ESI) m/z: $[M+H]^+$ Calcd for $C_{10}H_7BrCl_3O$ 326.8740; Found 326.8740.

6-Fluoro-3-(trichloromethyl)indan-1-one (3g). It was obtained from (*E*)-4,4,4-trichloro-1-(3-fluorophenyl)but-2-en-1-one **(2g**, 40 mg, 0.15 mmol) in 5 h. Yield: 37 mg (92%). Pale yellow solid, mp 48-50 °C. ^{1}H NMR ($CDCl_3$, 400 MHz): δ 8.09 dd (1H, J = 8.5, 4.5 Hz), 7.48 dd (1H, J = 7.2, 2.6 Hz), 7.40 td (1H, J = 8.5, 2.6 Hz), 4.60 dd (1H, J = 6.7, 3.8 Hz), 3.04 – 3.18 m (2H). ^{13}C NMR ($CDCl_3$, 101 MHz): δ 200.7 d (J = 2.7), 163.7 d (J = 252.3), 145.0 d (J = 2.5), 140.7 d (J = 7.6), 129.8 d (J = 8.3), 122.2 d (J = 23.6), 109.9 d (J = 22.2), 101.6, 58.5, 43.3. ^{19}F NMR ($CDCl_3$, 376 MHz): δ - 110.1. HRMS (MALDI) m/z: $[M+H]^+$ Calcd for $C_{10}H_7Cl_3FO$ 266.9541; Found 266.9539.

5,7-Dichloro-3-(trichloromethyl)indan-1-one (3h). It was obtained from 4,4,4-trichloro-1-(2,4-dichlorophenyl)-3-hydroxybutan-1-one **(1h**, 57 mg, 0.20 mmol) in 5 h. Yield: 25 mg (38%). Reddish solid, mp 80-82 °C. ^{1}H NMR ($CDCl_3$, 400 MHz): δ 8.01 s (1H), 7.54 s (1H), 4.52 t (1H, J = 5.5 Hz), 3.05-3.20 m (2H). ^{13}C NMR ($CDCl_3$, 101 MHz): δ 197.3, 152.3, 141.0, 139.1, 132.9, 131.8, 126.9, 100.7, 57.6, 43.4. HRMS (MALDI) m/z: $[M+H]^+$ Calcd for $C_{10}H_6Cl_5O$ 316.8856; Found 316.8856.

3-(Trichloromethyl)-5-(trifluoromethyl)indan-1-one (3i). It was obtained from the mixture of (*E*-, *Z*-)-4,4,4-trichloro-1-((4-trifluoromethyl)phenyl)but-2-en-1-ones (**2i**, 34 mg, 0.14 mmol) in 10 h. Yield: 27 mg (60%). In the same way, it was obtained from hydroxy ketone **1i** in 15 h in 65% yield. Pale yellow solid, mp 85-87 °C. ^{1}H NMR ($CDCl_3$, 400 MHz): δ 8.37 s (1H), 7.96 d (1H, J = 8.0 Hz), 7.84 d (1H, J = 8.0 Hz), 4.68 t (1H, J = 5.3 Hz), 3.10–3.21 m (2H). ^{13}C NMR ($CDCl_3$, 101 MHz): δ 200.7, 149.6, 141.1, 136.0 q (*J* = 32.7 Hz), 127.1 q (*J* = 3.5 Hz), 125.2 q (*J* = 4.0 Hz), 124.5, 123.3 q (*J* = 273.3 Hz), 100.9, 58.8, 42.9. ^{19}F NMR ($CDCl_3$, 376 MHz): δ -62.8 (CF_3). HRMS (MALDI) m/z: $[M+H]^+$ Calcd for $C_{11}H_7Cl_3F_3O$ 316.9509; Found 316.9511.

5-Phenoxy-3-(trichloromethyl)indan-1-one (3j). It was obtained from (*E*)-4,4,4-trichloro-1-(4-methoxyphenyl)but-2-en-1-one **(2j**, 104 mg, 0.37 mmol) in 4 h. Yield: 14 mg (13%). Pale yellow solid, mp 103-105 °C. ^{1}H NMR ($CDCl_3$, 400 MHz): δ 8.30 d (1H, J = 1.5 Hz), 7.91 d (1H, J = 8.0 Hz), 7.80 dd (1H, J = 8.0, 1.5 Hz), 7.64 - 7.69 m (2H), 7.44 – 7.55 m (3H), 4.67 dd (1H, J = 6.6, 4.0 Hz), 3.06 – 3.19 m (2H). ^{13}C NMR ($CDCl_3$, 101 MHz): δ 201.5, 150.2, 147.9, 139.8, 137.3, 129.2, 129.1, 128.7, 127.6, 126.6, 124.2, 101.8, 58.9, 43.1. HRMS (ESI) m/z: $[M+H]^+$ Calcd for $C_{16}H_{12}Cl_3O$ 340.9897; Found 340.9897.

6-Hydroxy-5-methoxy-3-(trichloromethyl)indan-1-one (3k). It was obtained from 4,4,4-trichloro-3-hydroxy-1-(3,4-dimethoxyphenyl)butan-1-one **(1k)** (108 mg, 0.36 mmol) in 5 h. Yield: 16 mg (15%). Pale yellow solid, mp 163-165 °C. ^{1}H NMR ($CDCl_3$, 400 MHz): δ 7.50 s (1H), 7.31 s (1H), 5.93 s (1H), 4.51 dd (1H, J = 7.2, 3.2 Hz), 4.05 s (3H), 2.91 – 3.11 m (2H). ^{13}C

NMR ($CDCl_3$, 101 MHz): δ 200.6, 152.2, 147.6, 143.4, 132.7, 108.6, 108.0, 102.2, 58.6, 56.4, 42.8. HRMS (MALDI) m/z: $[M+H]^+$ Calcd for $C_{11}H_{10}Cl_3O_3$ 294.9690; Found 294.9693.

1-(Trichloromethyl)-cyclopentanaphthalen-3-one (3n). It was obtained from 4,4,4-trichloro-3-hydroxy-1-(naphthalen-2-yl)butan-1-one **(1n**, 50 mg, 0.16 mmol) in 3 h in TfOH. Yield: 43 mg (86%). In the same way, it was obtained from enone **2n** in 2 h in TfOH in 89% yield, and quantitatively in H_2SO_4. Pale yellow solid, mp 127-129°C. ^1H NMR ($CDCl_3$, 400 MHz): δ 8.52 d (1H, J = 7.4 Hz), 8.02 d (1H, J = 8.4 Hz), 7.98 d (1H, J = 7.4 Hz), 7.84 d (1H, J = 8.4 Hz), 7.68 m (2H), 5.20 d (1H, J = 6.9 Hz), 3.29 d (1H, J = 18.0 Hz), 3.10 dd (1H, J = 18.0, 6.9 Hz). ^{13}C NMR ($CDCl_3$, 101 MHz): δ 202.0, 148.9, 137.5, 137.1, 131.8, 130.8, 129.0, 128.9, 126.9, 126.7, 119.0, 103.2, 58.2, 45.3, 22.4. HRMS (ESI) m/z: $[M+H]^+$ Calcd for $C_{14}H_{10}Cl_3O$ 298.9791; Found 298.9798.

5,6-Dimethyl-3-(trichloromethyl)indan-1-one (3p). It was obtained from 4,4,4-trichloro-3-hydroxy-1-(3,4-dimethylphenyl)butan-1-one **(1p**, 80 mg, 0.27 mmol) in 5 h. Yield: 10 mg (15%). Pale yellow solid, mp 160-162 °C. ^1H NMR ($CDCl_3$, 400 MHz): δ 7.85 s (1H), 7.61 s (1H), 4.55 dd (1H, J = 6.9, 3.7 Hz), 2.95-3.10 m (2H), 2.42 s (3H), 2.37 s (3H). ^{13}C NMR ($CDCl_3$, 101 MHz): δ 201.5, 149.9, 146.0, 136.3, 131.0, 128.3, 123.7, 101.8, 58.8, 43.0, 22.4. HRMS (ESI) m/z: $[M+H]^+$ Calcd for $C_{12}H_{12}Cl_3O$ 276.9948; Found 276.9945.

5,7-Dimethyl-3-(trichloromethyl)indan-1-one (3q). It was obtained from 4,4,4-trichloro-3-hydroxy-1-(2,4-dimethylphenyl)butan-1-one **(1q**, 30 mg, 0.10 mmol) in 5 h. Yield: 13 mg (43%). Colorless oil. ^1H NMR ($CDCl_3$, 400 MHz): δ 7.71 s (1H), 7.12 s (1H), 4.49 dd (1H, J = 6.8, 4.1 Hz), 2.95-3.08 m (2H), 2.65 s (3H), 2.46 s (3H). ^{13}C NMR ($CDCl_3$, 101 MHz): δ 202.4, 150.6, 145.0, 138.8, 133.6, 132.9, 125.9, 102.8, 58.2, 43.5, 22.1, 18.3. HRMS (ESI) m/z: $[M+H]^+$ Calcd for $C_{12}H_{12}Cl_3O$ 276.9948; Found 276.9951.

4,7-Dimethyl-3-(trichloromethyl)indan-1-one (3r). It was obtained from 4,4,4-trichloro-3-hydroxy-1-(2,5-dimethylphenyl)butan-1-one **(1r**, 50 mg, 0.26 mmol) in 3 h. Yield: 40 mg (80%). Reddish solid, mp 81-83 °C. ^1H NMR ($CDCl_3$, 400 MHz): δ 7.37 d (1H, J = 7.8 Hz), 7.22 d (1H, J = 7.8 Hz), 4.60 d (1H, J = 7.3 Hz), 3.10 d (1H, J = 17.9 Hz), 2.92 dd (1H, J = 17.9, 7.3 Hz), 2.65 s (3H), 2.58 s (3H). ^{13}C NMR ($CDCl_3$, 101 MHz): δ 201.5, 149.9, 146.0, 136.3, 131.0, 128.3, 123.7, 101.8, 58.8, 43.0, 22.4. HRMS (ESI) m/z: $[M+H]^+$ Calcd for $C_{12}H_{12}Cl_3O$ 276.9948; Found 276.9948.

4,5,7-Trimethyl-3-(trichloromethyl)indan-1-one (3s). It was obtained from 4,4,4-trichloro-3-hydroxy-1-(2,4,5-trimethylphenyl)butan-1-one **(1s**, 60 mg, 0.21 mmol) in 5 h. Yield: 15 mg (25%). Reddish solid, mp 124-126 °C. ^1H NMR ($CDCl_3$, 400 MHz): δ 7.13 s (1H), 4.63 d (1H, J = 7.2 Hz), 3.09 d (1H, J = 17.8 Hz), 2.93 dd (1H, J = 17.8, 7.2 Hz), 2.62 s (3H), 2.44 s (3H), 2.37 s (3H). ^{13}C NMR ($CDCl_3$, 101 MHz): δ 203.0, 147.9, 144.3, 135.7, 134.1, 134.0,

133.5, 104.3, 57.8, 45.8, 20.6, 18.2, 17.8. HRMS (MALDI) m/z: $[M+H]^+$ Calcd for $C_{13}H_{14}Cl_3O$ 291.0104; Found 291.0107.

5-*tert*-Butyl-3-(trichloromethyl)indan-1-one (3v). It was obtained from (*E*)-1-(4-*tert*-butylphenyl)-4,4,4-trichlorobut-2-en-1-one (**2v**, 30 mg, 0.10 mmol) in 5 h. Yield: 15 mg (50%). Yellow oil. ^{1}H NMR ($CDCl_3$, 400 MHz): δ 8.10 s (1H), 7.77 d (1H, J = 8.1 Hz), 7.62 dd (1H, J = 8.1, 1.4 Hz), 4.59 dd (1H, J = 6.5, 4.1 Hz), 2.97 - 3.13 m (2H), 1.41 s (9H). ^{13}C NMR ($CDCl_3$, 101 MHz): δ 201.6, 159.0, 149.7, 136.2, 127.4, 124.8, 123.4, 102.0, 58.9, 43.1, 35.7, 31.2. HRMS (MALDI) m/z: $[M+H]^+$ Calcd for $C_{14}H_{16}Cl_3O$ 305.0261; Found 305.0260.

Generating and NMR registration of cations A, B in TfOH. Cations **Aa**, **Ac**, and **Ad** were generated by dissolving 10 mg of the corresponding hydroxy ketone **1a**, **1c**, or **1d** in 0.5 mL TfOH in an NMR tube at room temperature. Cations **Ba**, **Bc**, **Bd**, and **Bm** were generated by dissolving 10 mg of the corresponding enone **2a**, **2c**, **2d**, or **2m** in 0.5 mL TfOH in an NMR tube at room temperature. A small amount of CH_2Cl_2 was added as NMR internal standard. Additionally, cation *E*-**Bm** was obtained upon dissolving of hydroxy ketone **1m** in TfOH due to fast dehydration of the latter.

Cation Aa. ^{1}H NMR ($CDCl_3$, 400 MHz): δ 8.58 d (2H, J = 8.0 Hz), 8.27 t (1H, J = 7.5 Hz), 7.88 t (2H, J = 7.9 Hz), 5.21 dd (1H, J = 7.7, 4.1 Hz), 4.54 dd (1H, J = 18.6, 4.1 Hz), 4.15 dd (1H, J = 18.6, 7.7 Hz). ^{13}C NMR ($CDCl_3$, 101 MHz): 218.2, 145.9, 135.4, 131.4, 129.7, 98.9, 81.0, 34.2.

Cation Ac. ^{1}H NMR ($CDCl_3$, 400 MHz): δ 8.47 d (2H, J = 8.2 Hz), 7.71 d (2H, J = 8.2 Hz), 5.15 dd (1H, J = 7.8, 3.9 Hz), 4.46 dd (1H, J = 18.3, 4.0 Hz), 4.07 dd (1H, J = 18.3, 7.8 Hz), 2.70 c (3H). ^{13}C NMR ($CDCl_3$, 101 MHz): 214.5, 162.0, 135.8, 132.3, 127.1, 99.0, 80.9, 33.9, 22.6.

Cation Ad. ^{1}H NMR ($CDCl_3$, 400 MHz): δ 8.69 dd (2H, J = 8.9, 4.8 Hz), 7.54 t (2H, J = 8.3 Hz), 5.20 dd (1H, J = 7.6, 3.9 Hz), 4.49 dd (1H, J = 18.6, 4.0 Hz), 4.11 dd (1H, J = 18.6, 7.6 Hz). ^{13}C NMR ($CDCl_3$, 101 MHz): 215.3, 174.3 d (*J* = 277.7 Hz), 139.6 d (*J* = 13.1 Hz), 126.3, 119.4 d (*J* = 23.0 Hz), 98.8, 80.9, 34.1. ^{19}F NMR ($CDCl_3$, 376 MHz): δ -77.9.

Cation Ba. ^{1}H NMR ($CDCl_3$, 400 MHz) δ, м.d.: 8.43 d (2H, J = 8.3 Hz), 7.90 d (1H, J = 12.0 Hz), 7.86 d (1H, J = 12.0 Hz), 7.77 d (2H, J = 8.3 Hz), 2.74 s (3H). ^{13}C NMR ($CDCl_3$, 101 MHz): 204.9, 159.0, 146.0, 136.3, 131.6, 129.7, 120.7, 90.0.

Cation Bc. ^{1}H NMR ($CDCl_3$, 400 MHz): δ 8.43 d (2H, J = 8.3 Hz), 7.84 d (1H, J = 12.0 Hz), 7.82 d (1H, J = 12.0 Hz), 7.77 d (2H, J = 8.3 Hz), 2.74 c (3H). ^{13}C NMR ($CDCl_3$, 101 MHz): 201.0, 163.3, 157.4, 136.9, 132.9, 127.1, 120.4, 90.2, 22.8.

Cation Bd. ^{1}H NMR ($CDCl_3$, 400 MHz): δ 8.64 dd (2H, J = 8.6, 4.8 Hz), 7.84 d (1H, J = 16.0 Hz), 7.80 d (1H, J = 16.0 Hz), 7.61 t (2H, J = 8.6 Hz). ^{13}C NMR ($CDCl_3$, 101 MHz): 202.0,

174.5 d (J = 279.4 Hz), 158.1, 140.8 d (J = 13.3 Hz), 126.2, 119.8 d (J = 23.1 Hz), 89.9. ^{19}F NMR ($CDCl_3$, 376 MHz): δ -77.9.

Cation Bm, mixture of *E*-, *Z*-isomers. *E*-**Bm**. ^{1}H NMR ($CDCl_3$, 400 MHz): δ 8.71 d (2H, J = 8.7 Hz), 8.62 d (2H, J = 8.7 Hz), 7.96 d (1H, J = 14.9 Hz), 7.85 d (1H, J = 14.9 Hz). ^{13}C NMR ($CDCl_3$, 101 MHz): 208.9, 162.9, 154.7, 137.2, 136.7, 126.0, 121.6, 89.6. *Z*-**Bm**. ^{1}H NMR ($CDCl_3$, 400 MHz): δ 8.76 d (2H, J = 8.7 Hz), 8.67 d (2H, J = 8.7 Hz), 7.18 d (1H, J = 12.4 Hz), 6.82 d (1H, J = 12.4 Hz). ^{13}C NMR ($CDCl_3$, 101 MHz): 214.5, 155.2, 145.8, 136.1, 135.5, 126.1, 118.2, 90.0.

2. References

1. Dolomanov, O. V.; Bourhis, L. J.; Gildea, R. J; Howard, J. A. K.; Puschmann, H. OLEX2: a complete structure solution, refinement and analysis program. *J. Appl. Cryst.* **2009**, *42*, 339–341.

2. Sheldrick, G. M. SHELXT - integrated space-group and crystalstructure determination. *Acta Crystallogr., Sect. A: Found. Crystallogr.* **2015**, *A71*, 3−8.

3. Sheldrick, G. M. Crystal structure refinement with SHELXL. *Acta Crystallogr., Sect. C: Struct. Chem.* **2015**, *C71*, 3−8.

4. T.A. Gus'kova, A.D. Durnev, D.V. Reikhart, A. P. Chernyavtseva, Robb, J. R.Cheeseman, G. Scalmani, V. Barone, B. Mennucci, G.A. Petersson, H. Nakatsuji, M. Caricato, X. Li, H.P. Hratchian, A.F. Izmaylov, J. Bloino, G. Zheng, J.L. Sonnenberg, M. Hada, M. Ehara, K. Toyota, R. Fukuda, J. Hasegawa, M. Ishida, T. Nakajima, Y. Honda, O. Kitao, H. Nakai, T.Jr. Vreven,, J.A. Montgomery, J.E. Peralta, F. Ogliaro, M. Bearpark, J.J. Heyd, E. Brothers, K.N. Kudin, V.N. Staroverov, T. Keith, R. Kobayashi, J. Normand, K. Raghavachari, A. Rendell, J.C. Burant, S.S. Iyengar, J. Tomasi, M. Cossi, N. Rega, J.M. Millam, M. Klene, J.E. Knox, J.B. Cross, V. Bakken, C. Adamo, J. Jaramillo, R. Gomperts, R.E. Stratmann, O. Yazyev, A.J. Austin, R. Cammi, C. Pomelli, J.W. Ochterski, R.L. Martin, K. Morokuma, V.G. Zakrzewski, G.A. Voth, P. Salvador, J.J. Dannenberg, S. Dapprich, A.D. Daniels, O. Farkas, J.B. Foresman, J.V. Ortiz, J. Cioslowski, D.J. Fox in Gaussian 09, Revision C.01, Gaussian, Inc., Wallingford CT, **2010**.

5. Koenigs, W. Ueber Condensationen von Chloral und von Butylchloral mit Paraldehyd und mit Ketonen. *Chem. Berichte*, **1892**, *25*, 798-802.

6. Ganta, A.; Shamshina, J. L.; Cafiero, L. R.; Snowden, T. S. Stereoselective synthesis of *cis*- or *trans*-2,4-disubstituted butyrolactones from Wynberg lactone. *Tetrahedron*, **2012**, *68*, 5396 – 5405

7. Guirado, A.; Martiz, B.; Andreu, R.; Bautista, D.; Gálvez. J. First synthesis of 3-aryl-5-dichloromethyl-2-pyrazolines. The electrochemical generation of 2,2-dichlorovinylacetophenones as a key step. *Tetrahedron*, **2007**, *63*, 1175-1182.

3. ^{1}H, $^{13}C\{^{1}H\}$, $^{19}F\{^{1}H\}$, NOESY $^{1}H,^{1}H$ NMR spectra of compounds 1–3.

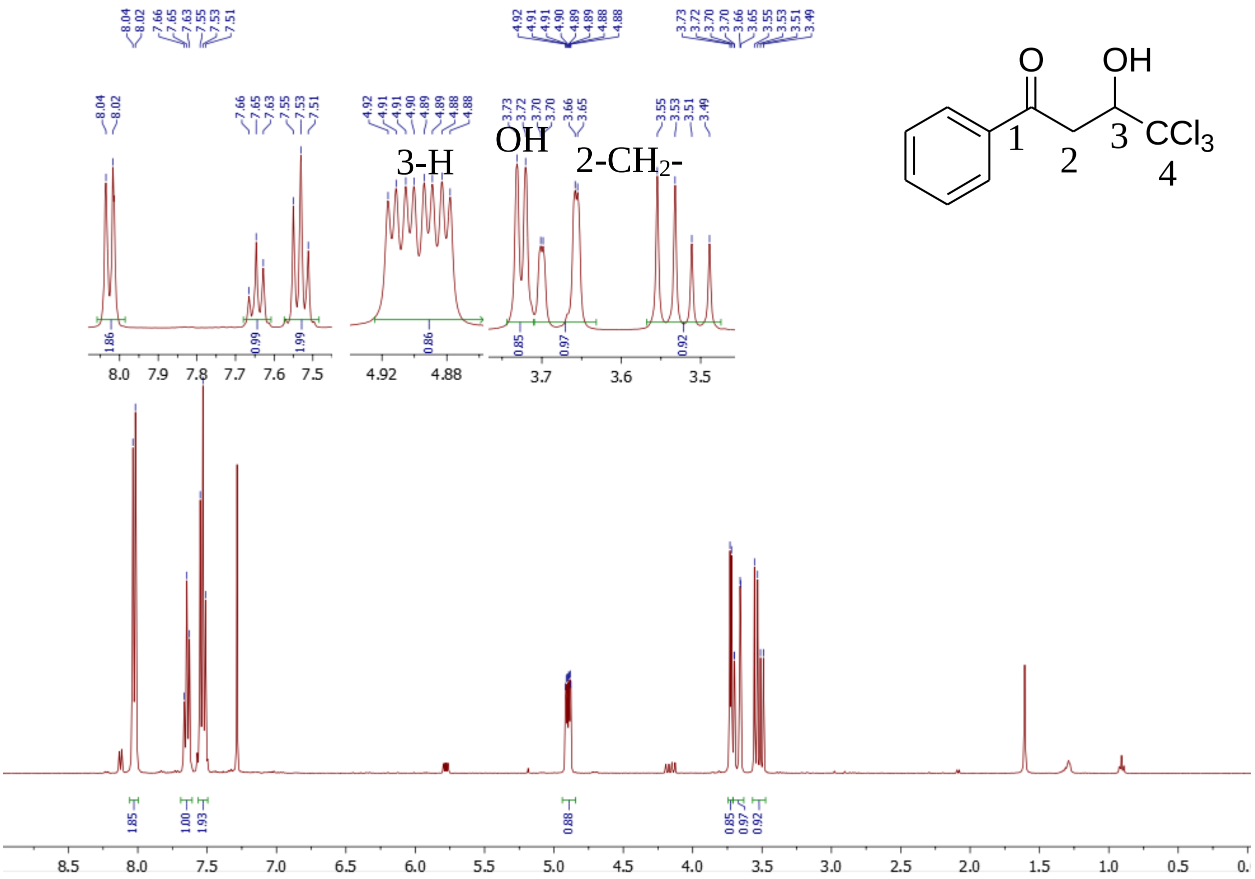

Figure S1. ^{1}H NMR spectrum of the compound **1a** ($CDCl_3$, 400 MHz).

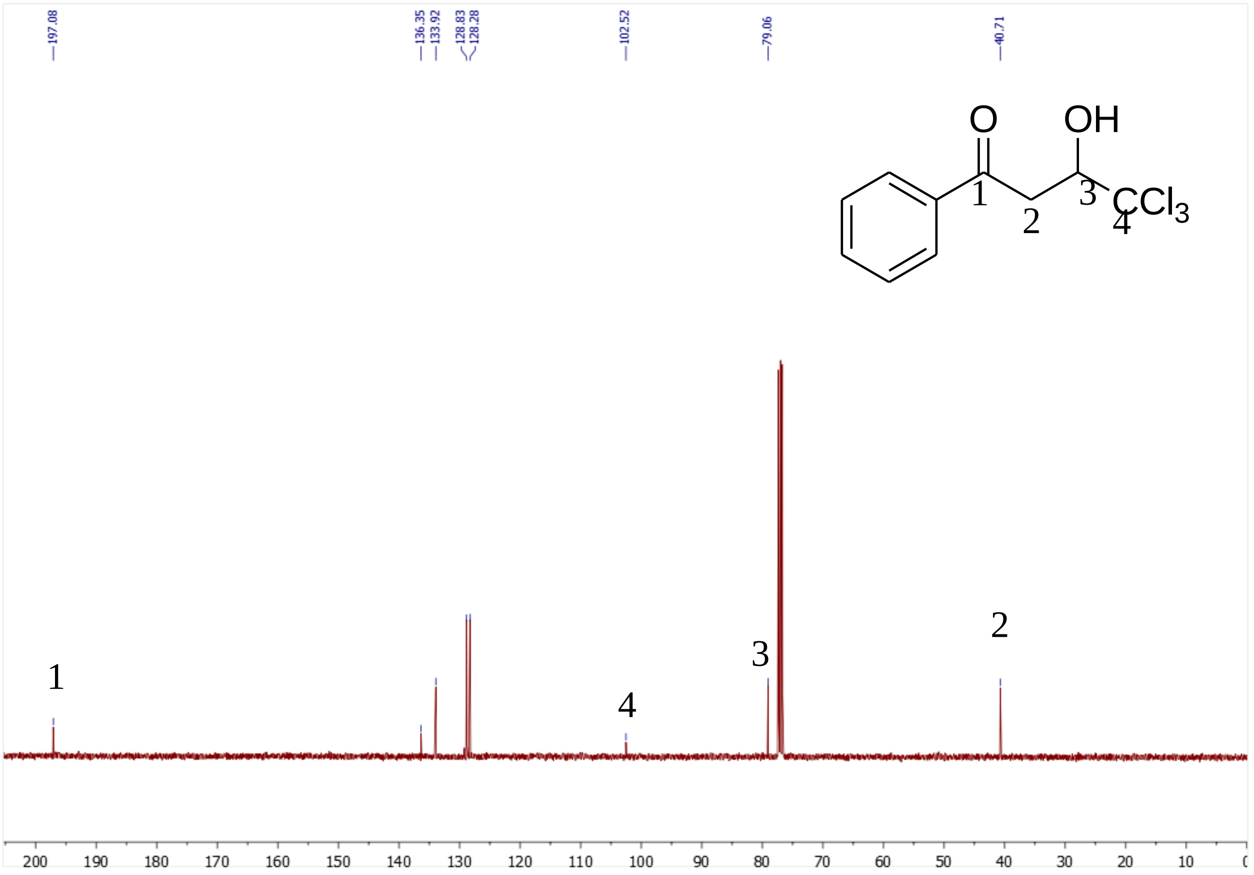

Figure S2. $^{13}C\{^{1}H\}$ NMR spectrum of the compound **1a** ($CDCl_3$, 101 MHz).

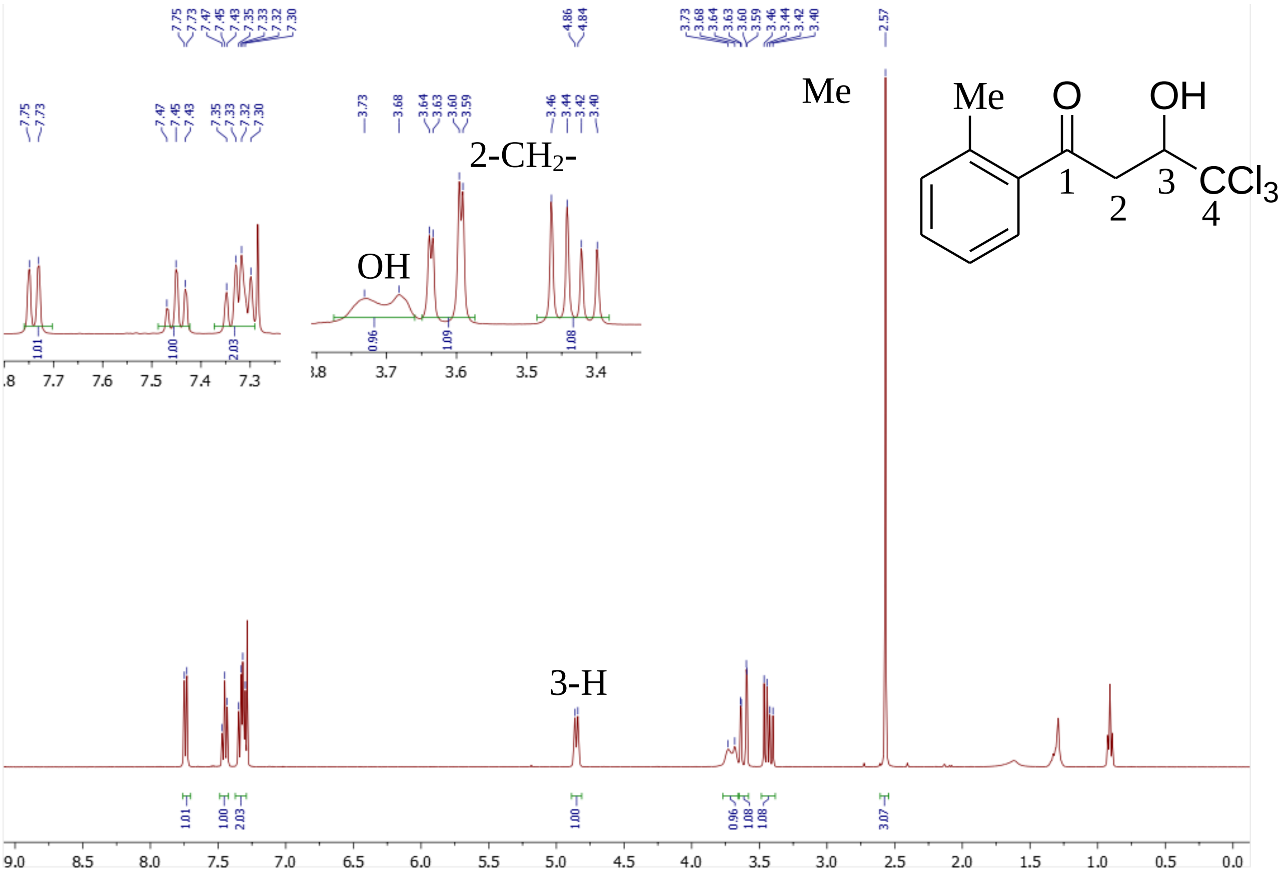

Figure S3. ^{1}H NMR spectrum of the compound **1b** ($CDCl_3$, 400 MHz).

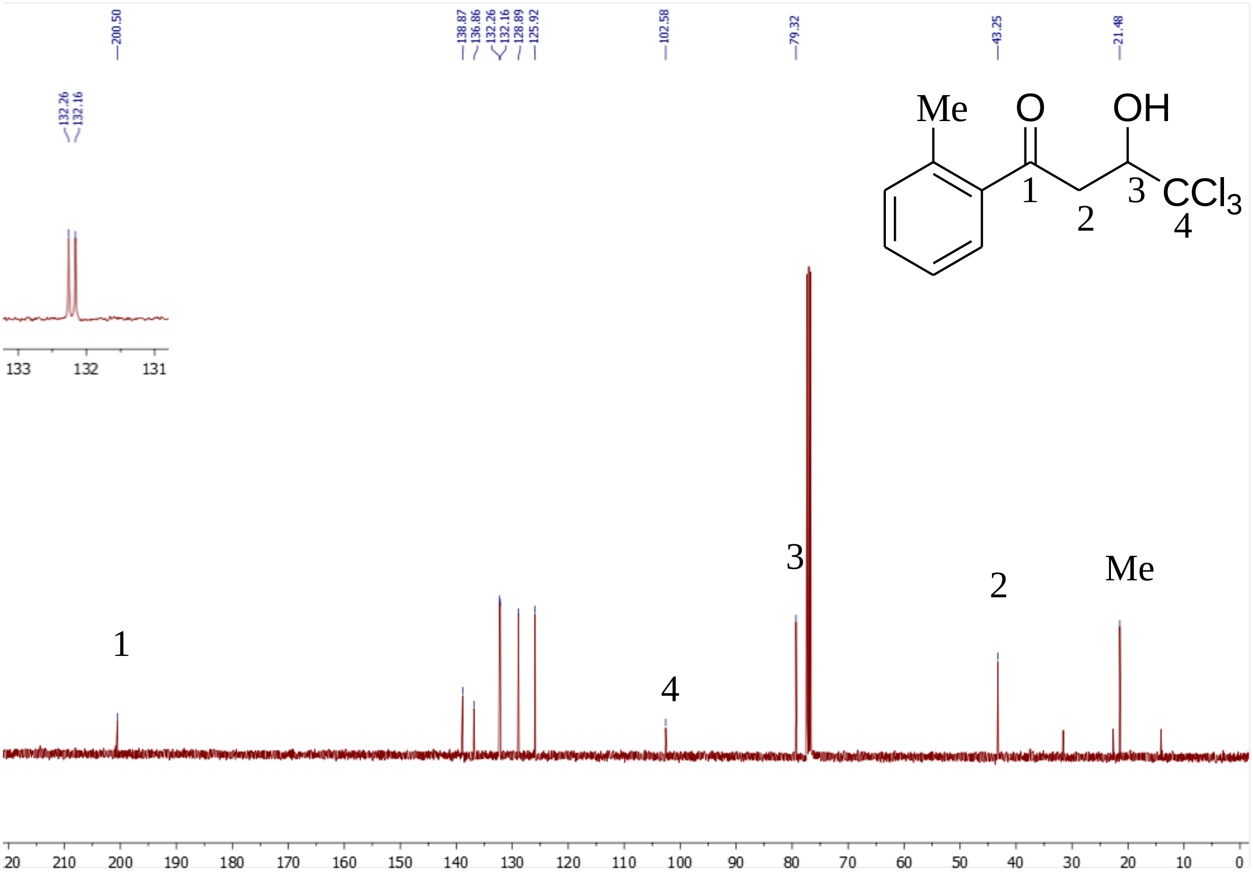

Figure S4. $^{13}C\{^1H\}$ NMR spectrum of the compound **1b** ($CDCl_3$, 101 MHz).

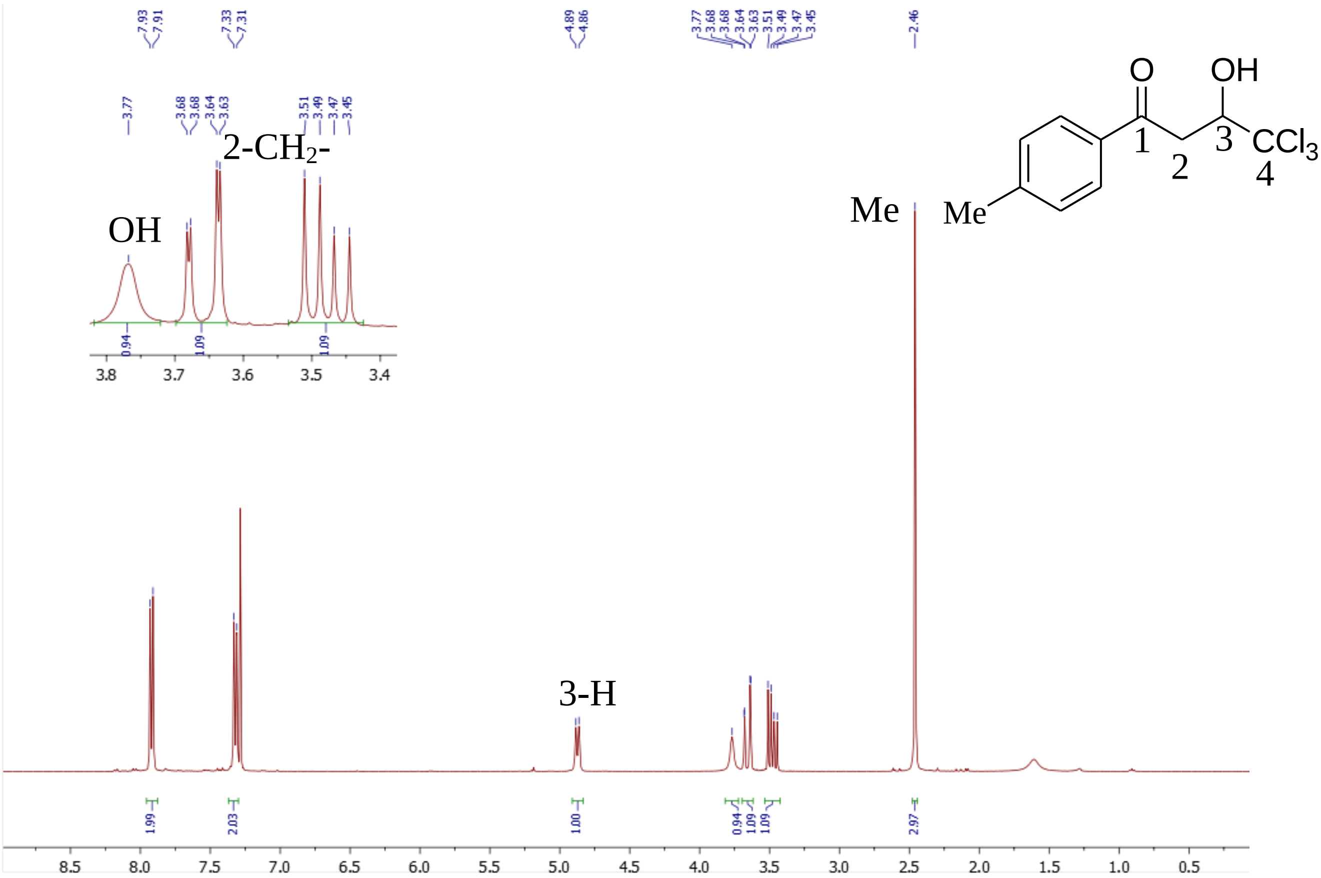

Figure S5. ^{1}H NMR spectrum of the compound **1c** ($CDCl_3$, 400 MHz).

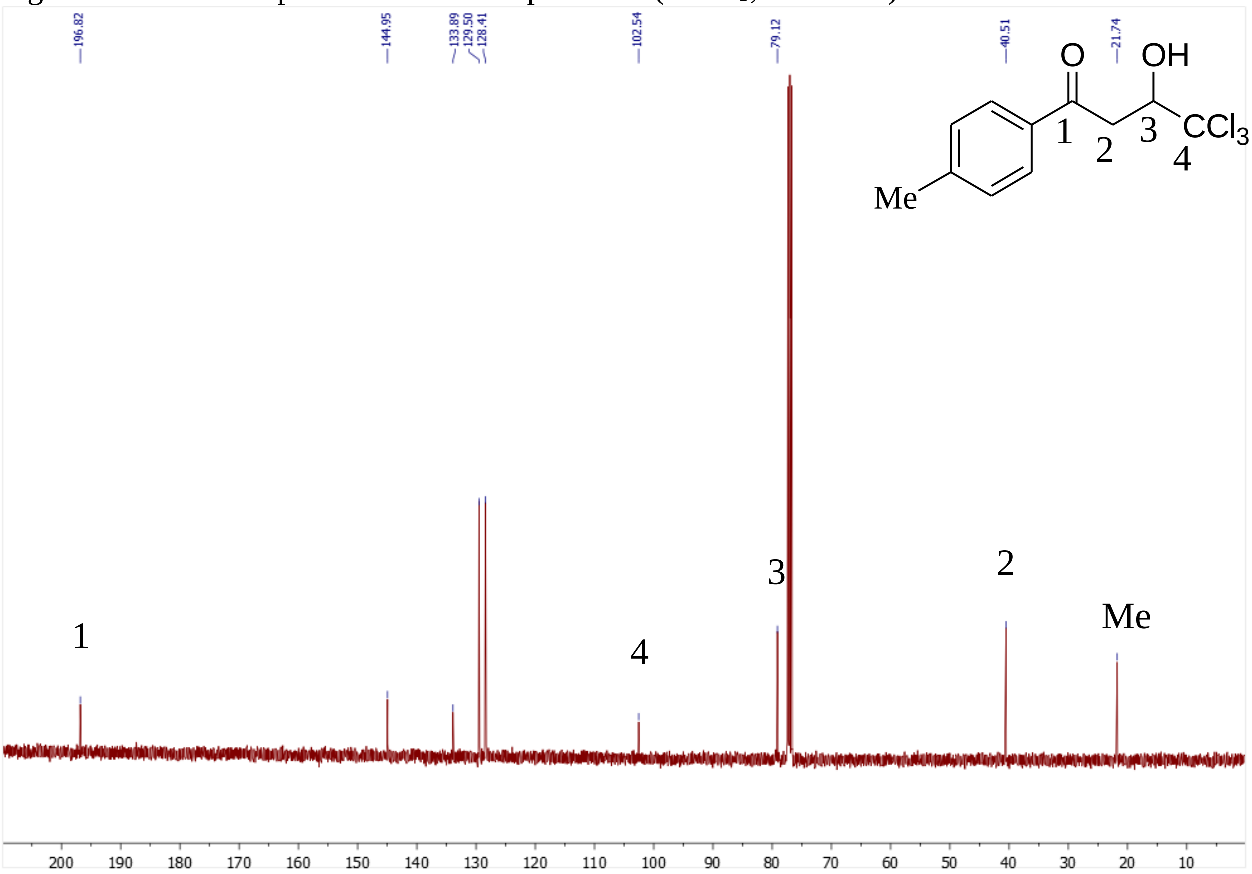

Figure S6. ^{13}C{^{1}H} NMR spectrum of the compound **1c** ($CDCl_3$, 101 MHz).

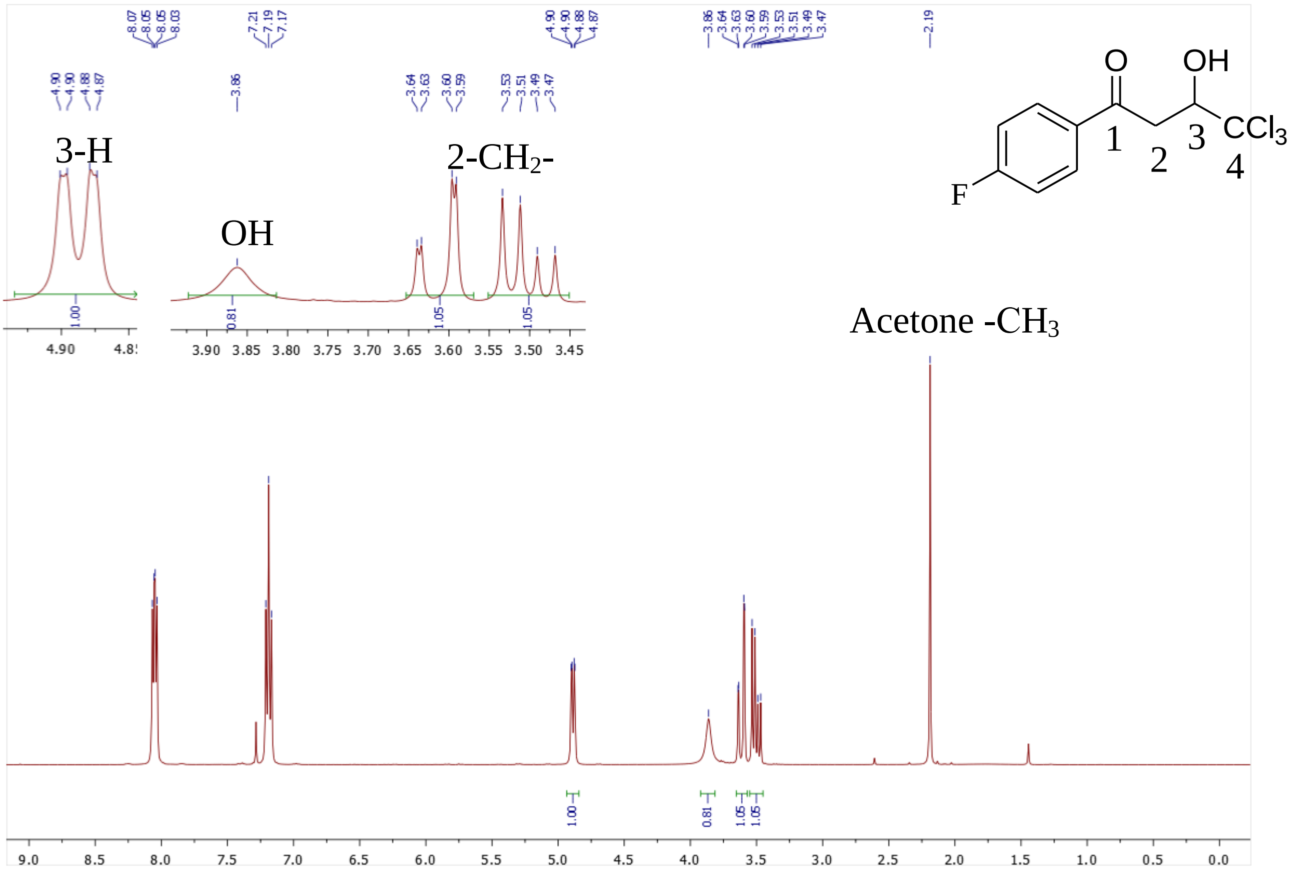

Figure S7. ^{1}H NMR spectrum of the compound **1d** ($CDCl_3$, 400 MHz).

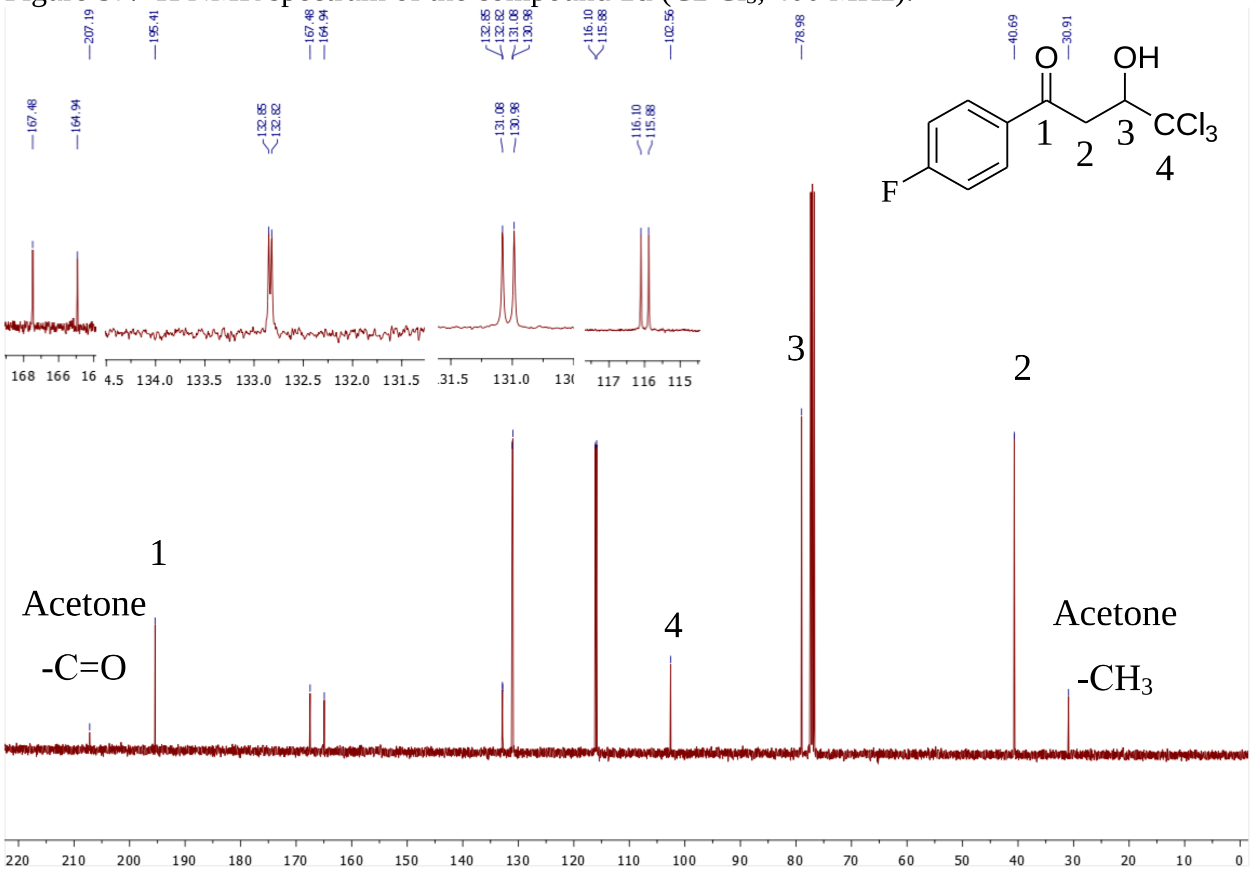

Figure S8. $^{13}C\{^{1}H\}$ NMR spectrum of the compound **1d** ($CDCl_3$, 101 MHz).

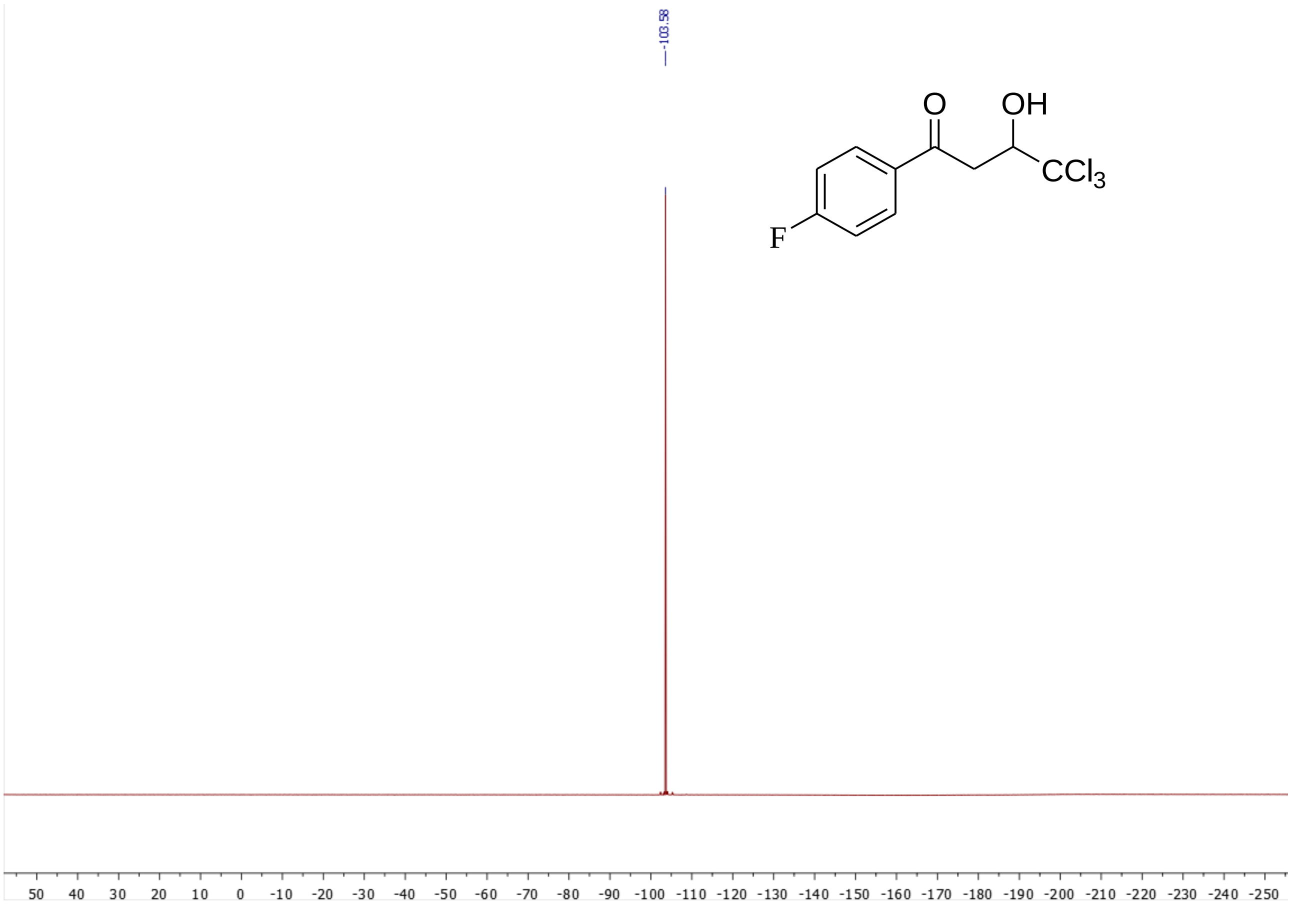

Figure S9. ^{19}F{^{1}H} NMR spectrum of the compound **1d** ($CDCl_3$, 376 MHz).

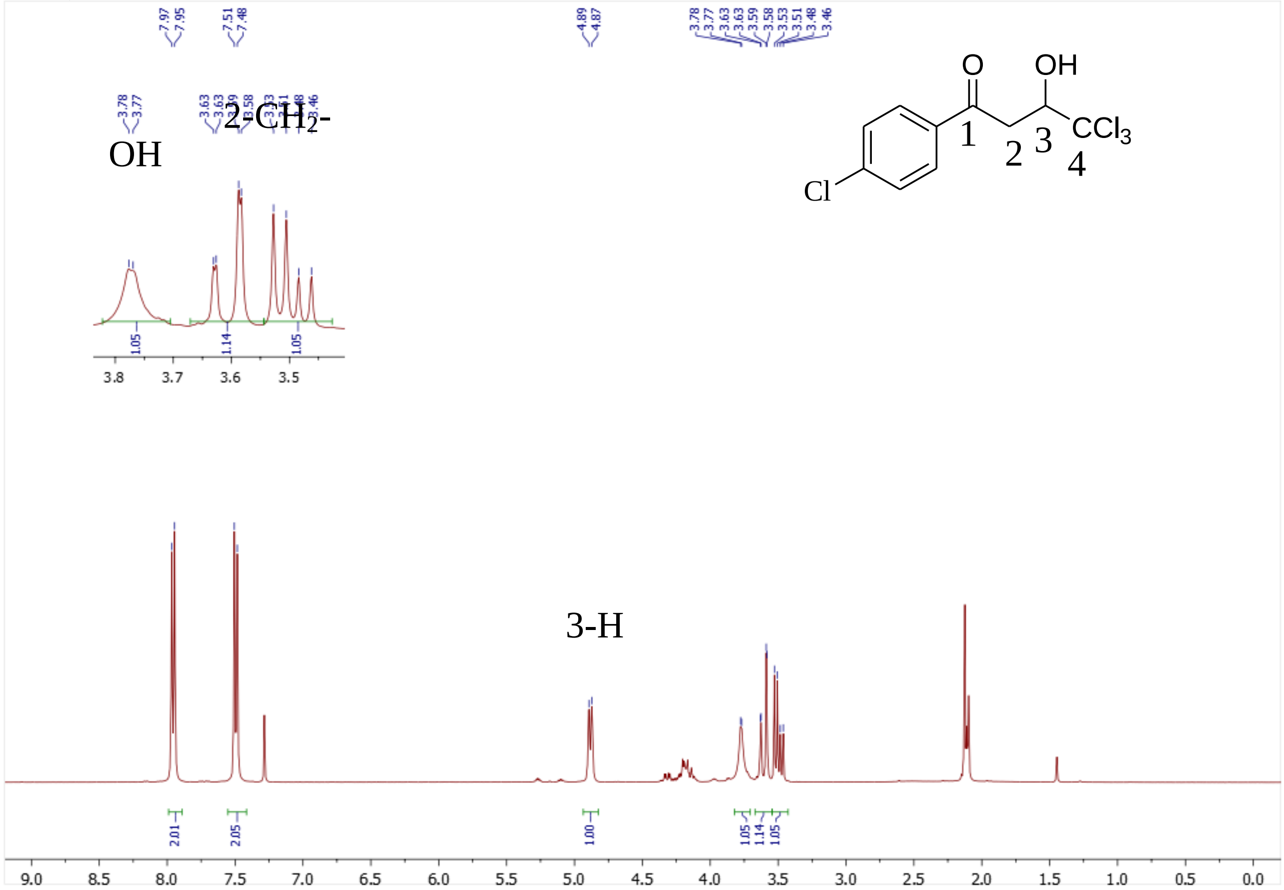

Figure S10. ^{1}H NMR spectrum of the compound **1e** ($CDCl_3$, 400 MHz).

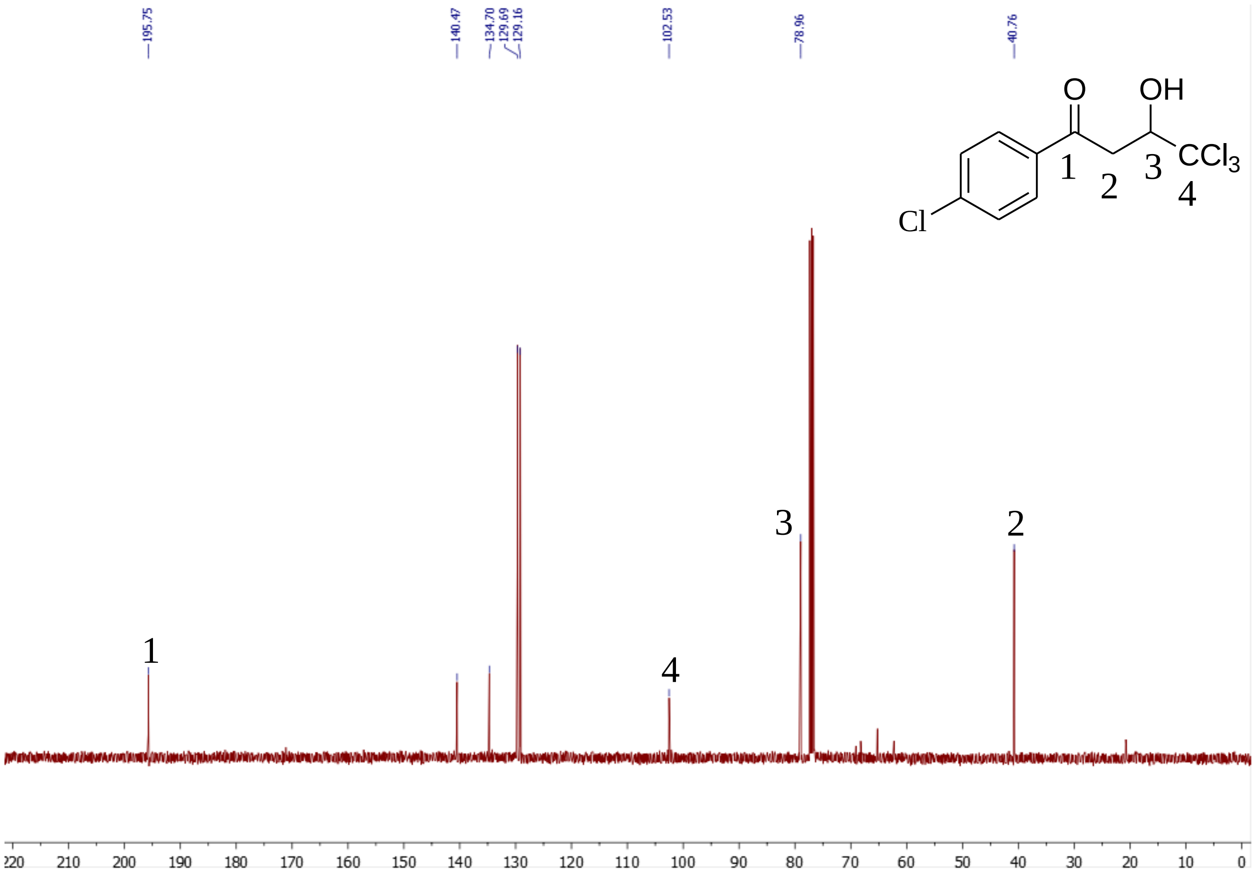

Figure S11. $^{13}C\{^{1}H\}$ NMR spectrum of the compound **1e** ($CDCl_3$, 101 MHz).

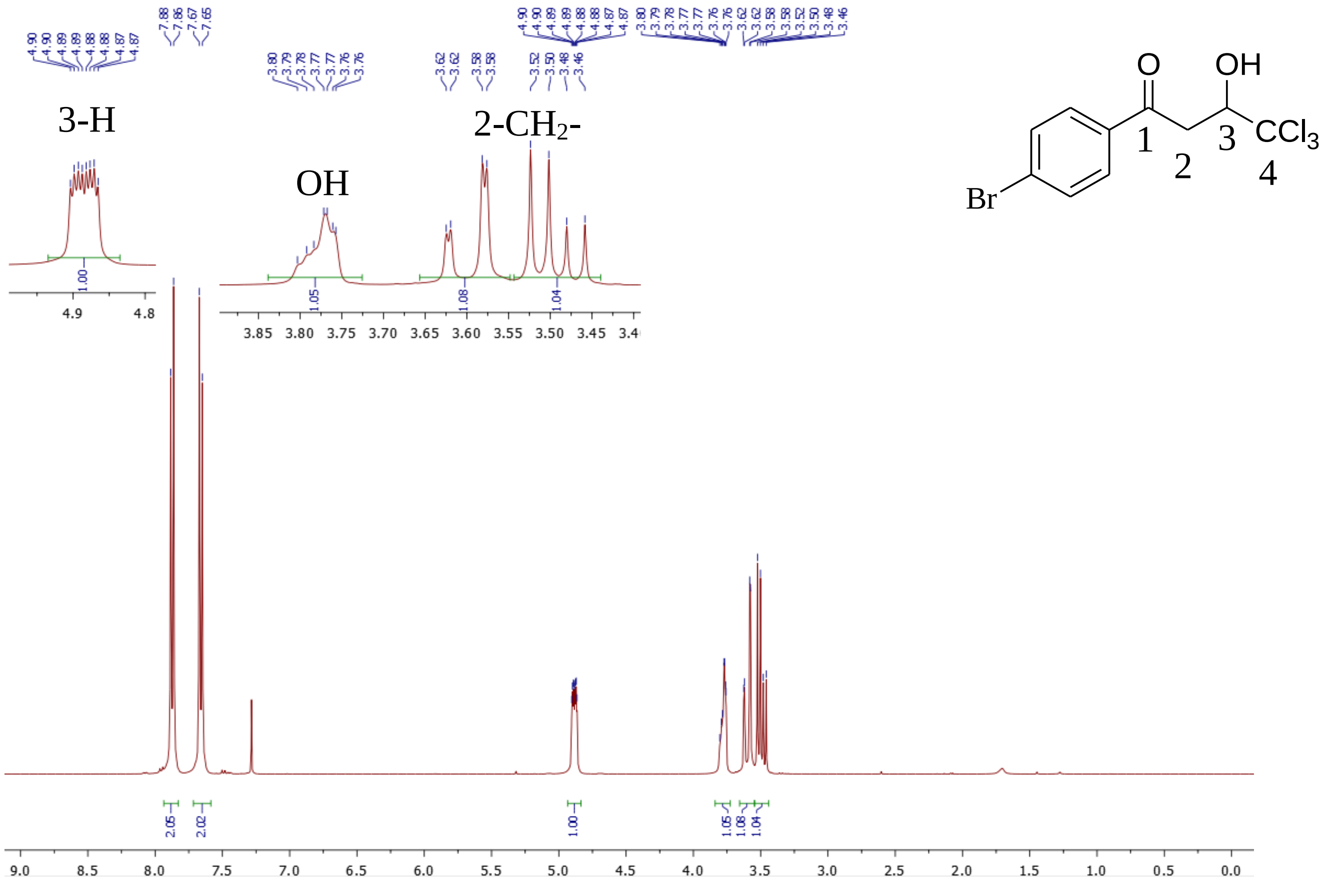

Figure S12. ^{1}H NMR spectrum of the compound **1f** ($CDCl_3$, 400 MHz).

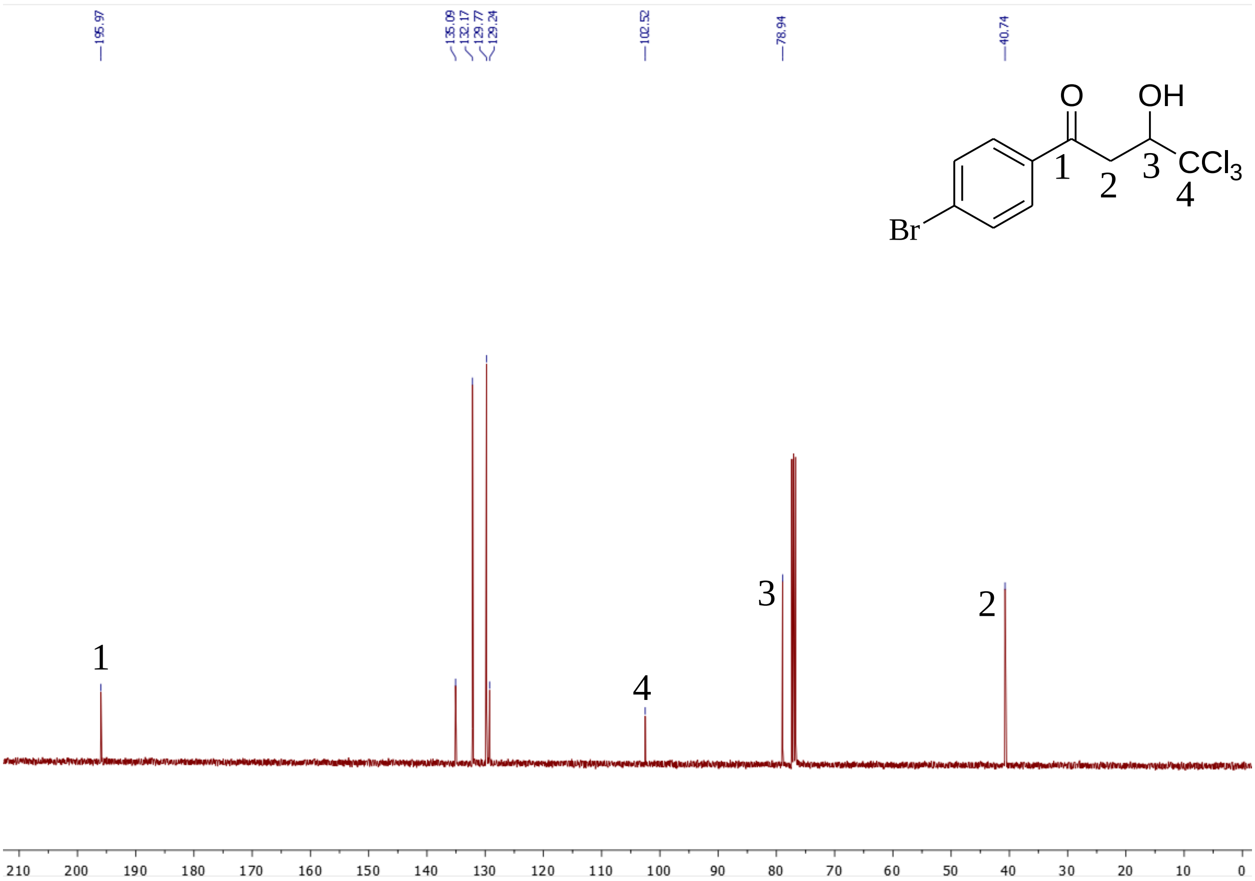

Figure S13. $^{13}C\{^{1}H\}$ NMR spectrum of the compound **1f** ($CDCl_3$, 101 MHz).

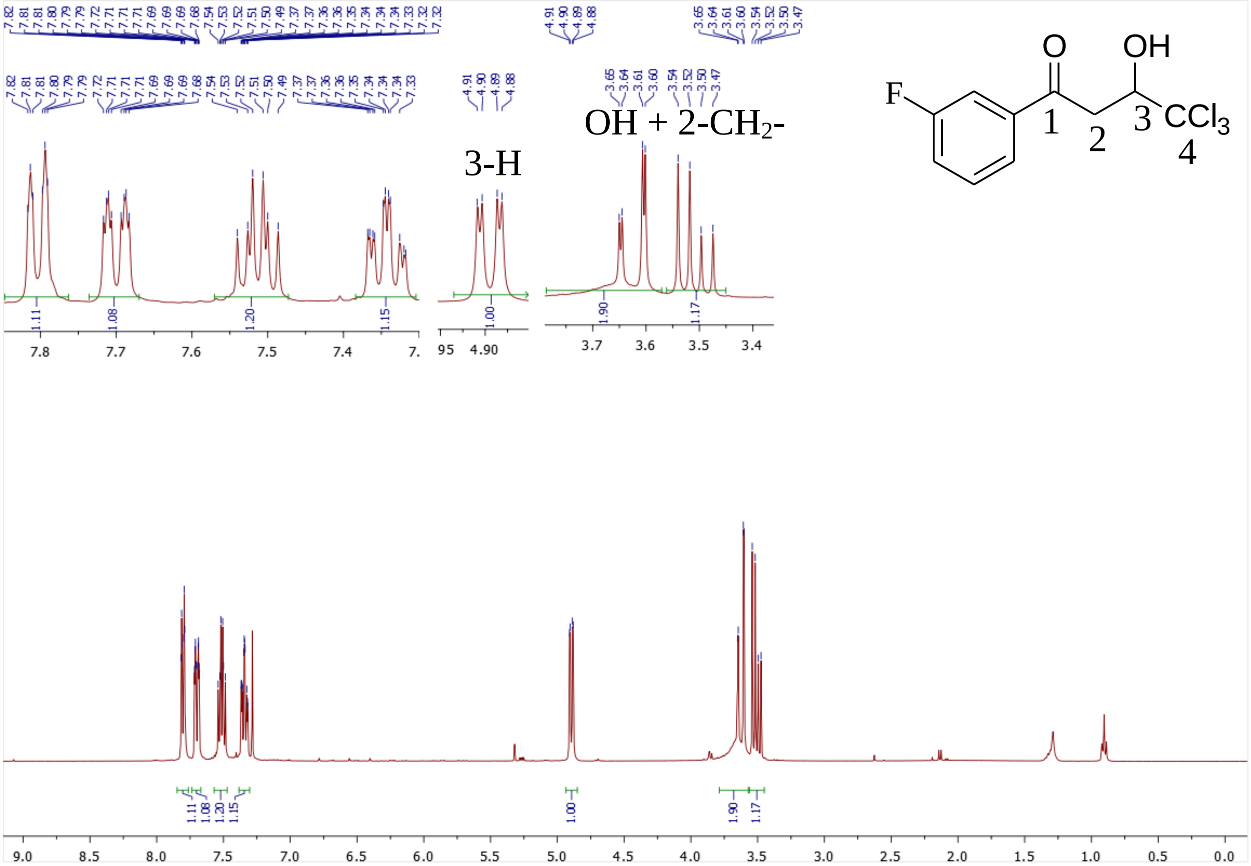

Figure S14. ^{1}H NMR spectrum of the compound **1g** ($CDCl_3$, 400 MHz).

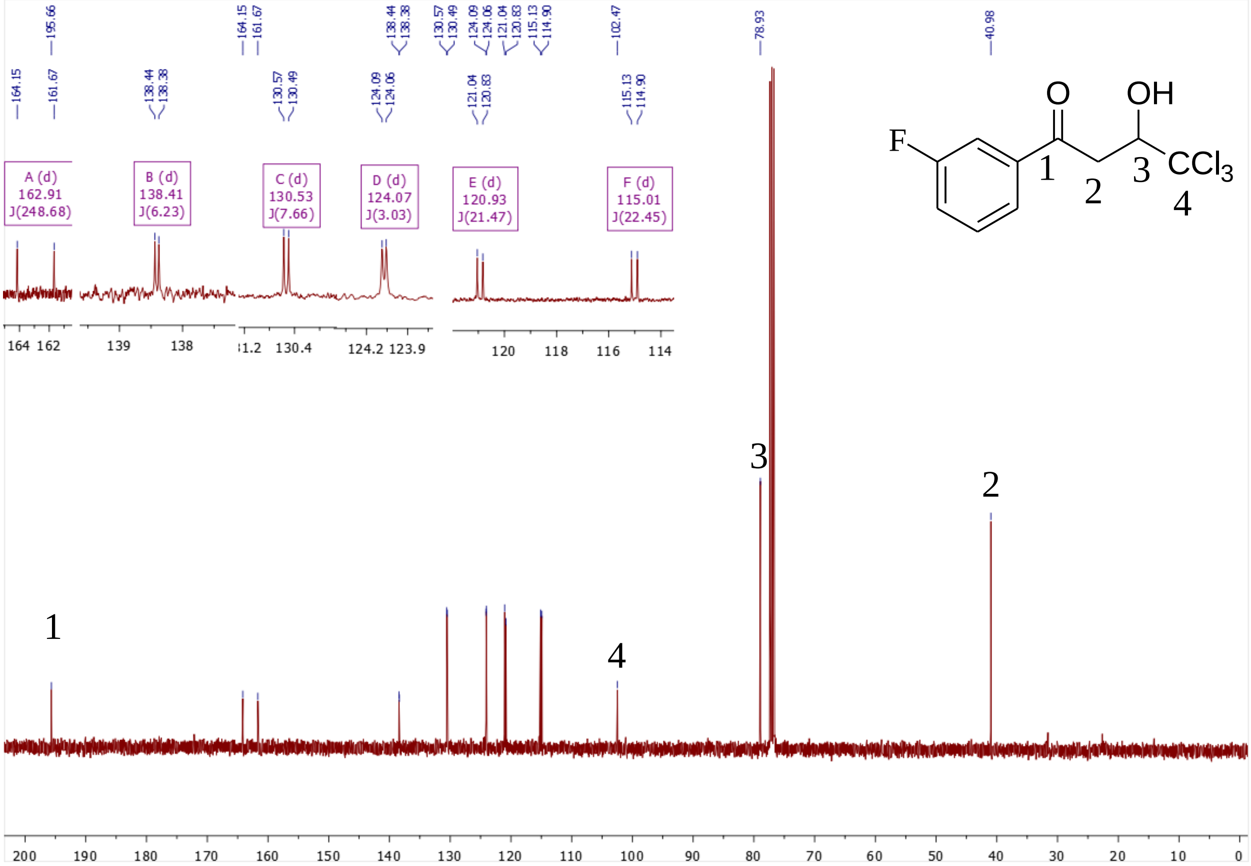

Figure S15. $^{13}C\{^1H\}$ NMR spectrum of the compound **1g** ($CDCl_3$, 101 MHz).

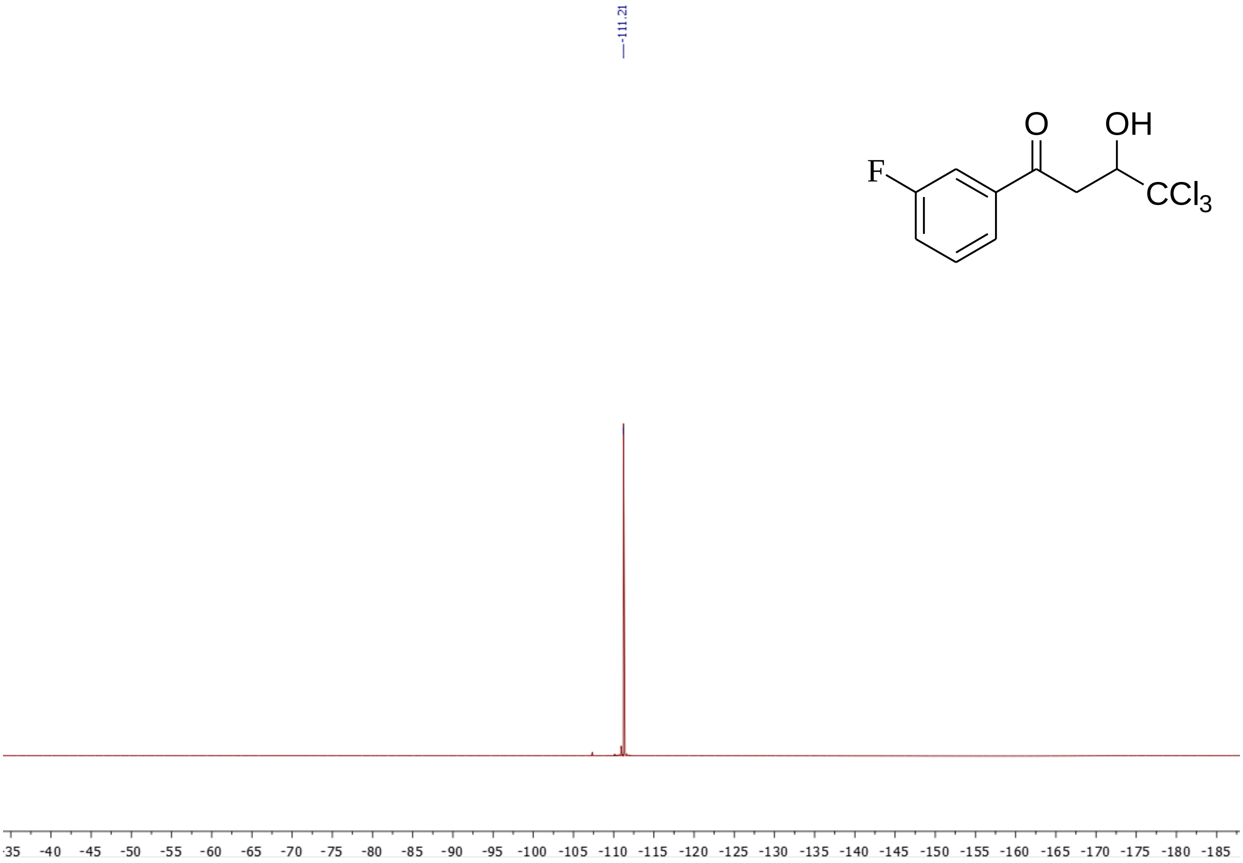

Figure S16. $^{19}F\{^1H\}$ NMR spectrum of the compound **1g** ($CDCl_3$, 376 MHz).

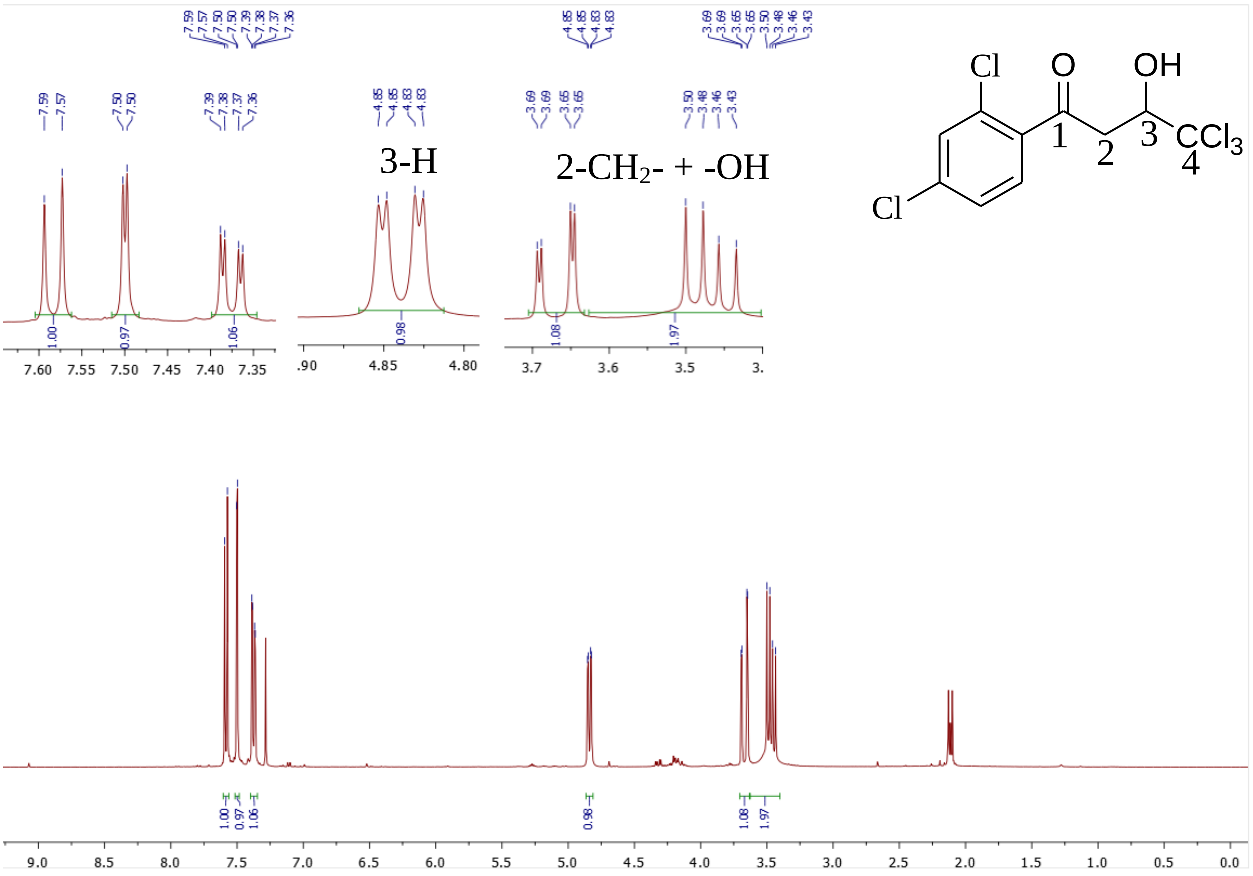

Figure S17. ^{1}H NMR spectrum of the compound **1h** ($CDCl_3$, 400 MHz).

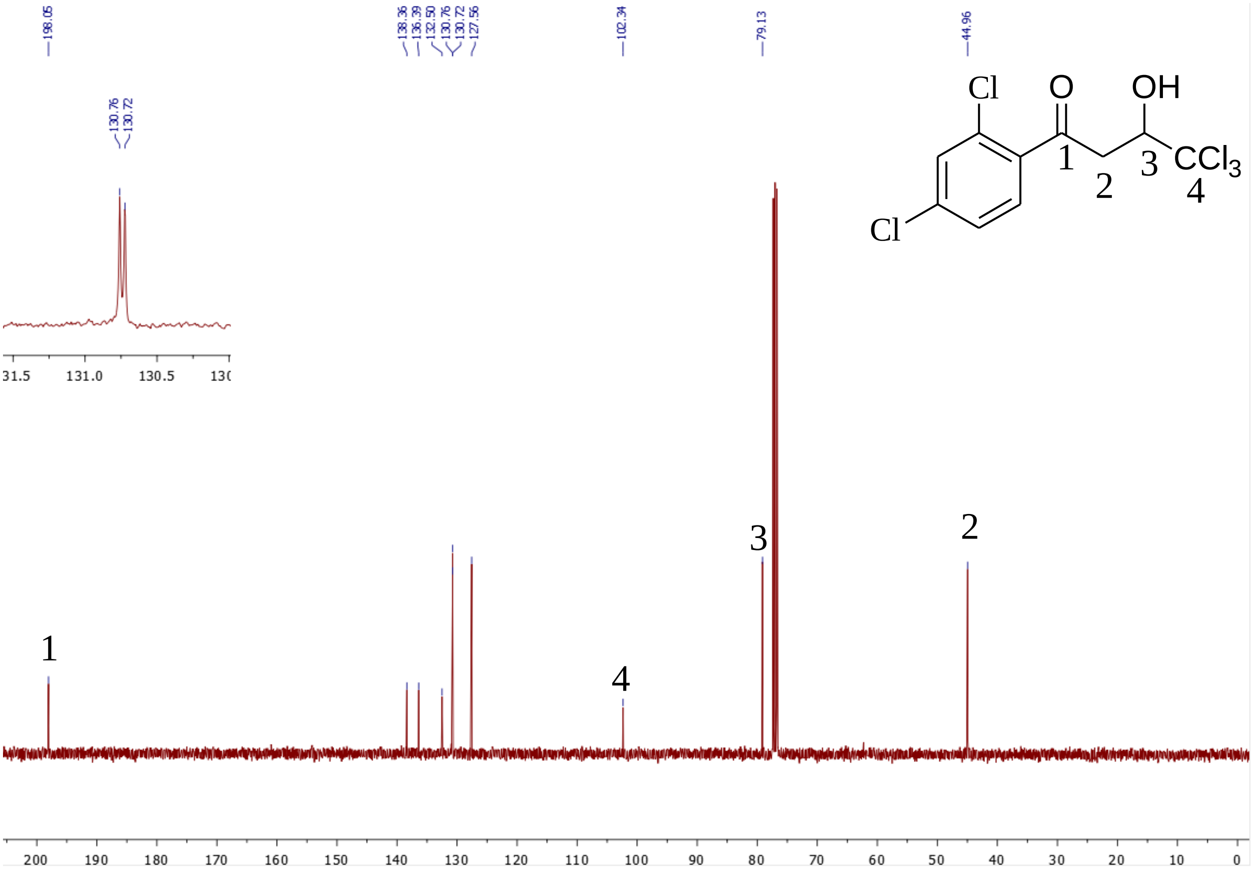

Figure S18. $^{13}C\{^{1}H\}$ NMR spectrum of the compound **1h** ($CDCl_3$, 101 MHz).

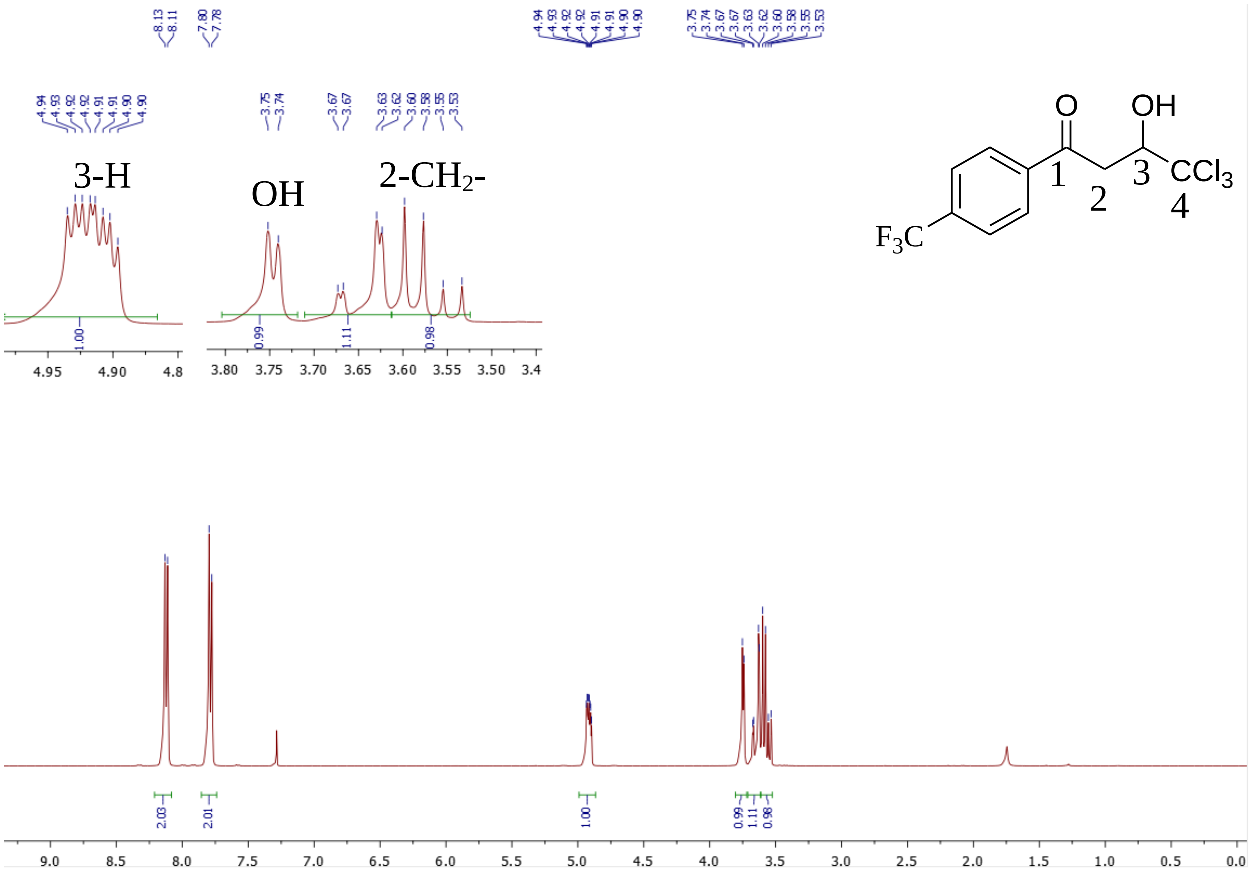

Figure S19. ^{1}H NMR spectrum of the compound **1i** ($CDCl_3$, 400 MHz).

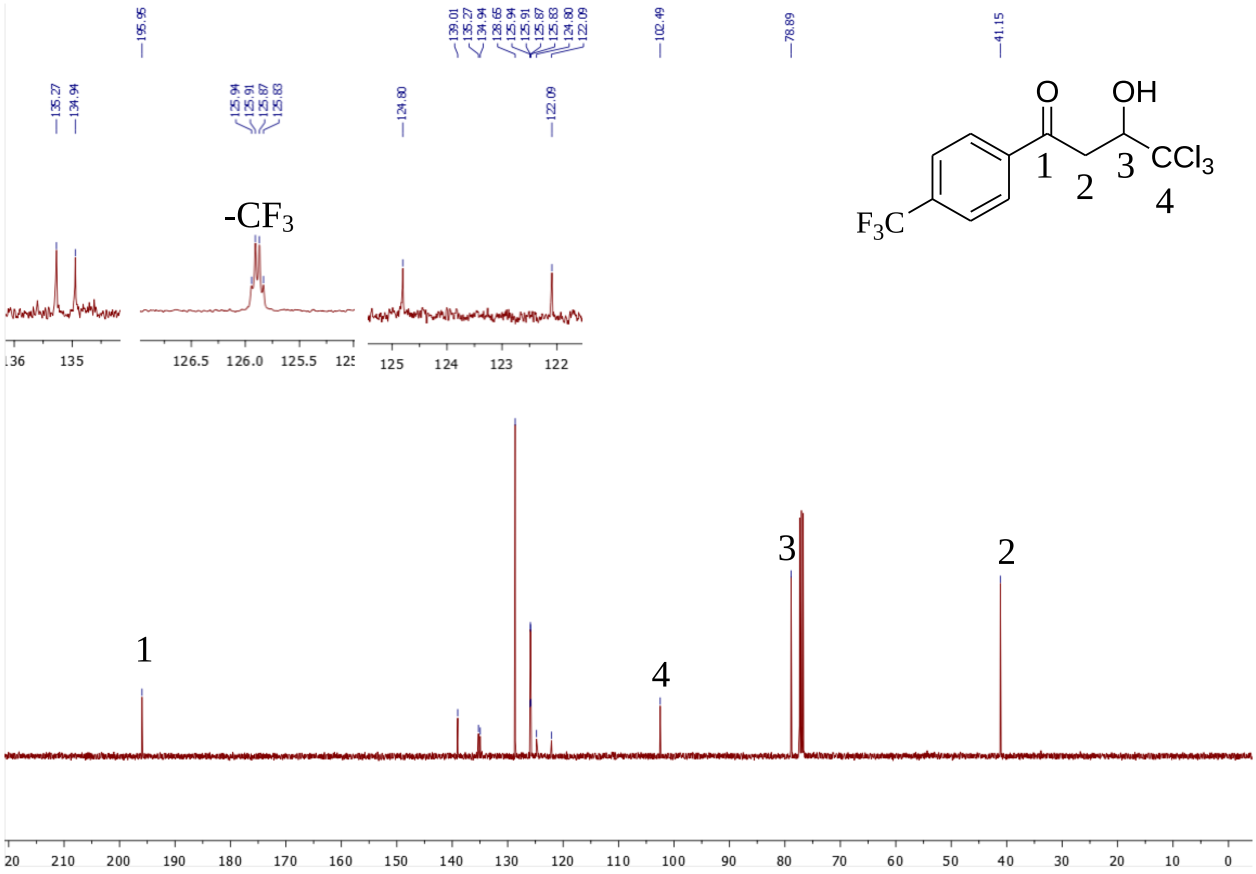

Figure S20. $^{13}C\{^{1}H\}$ NMR spectrum of the compound **1i** ($CDCl_3$, 101 MHz).

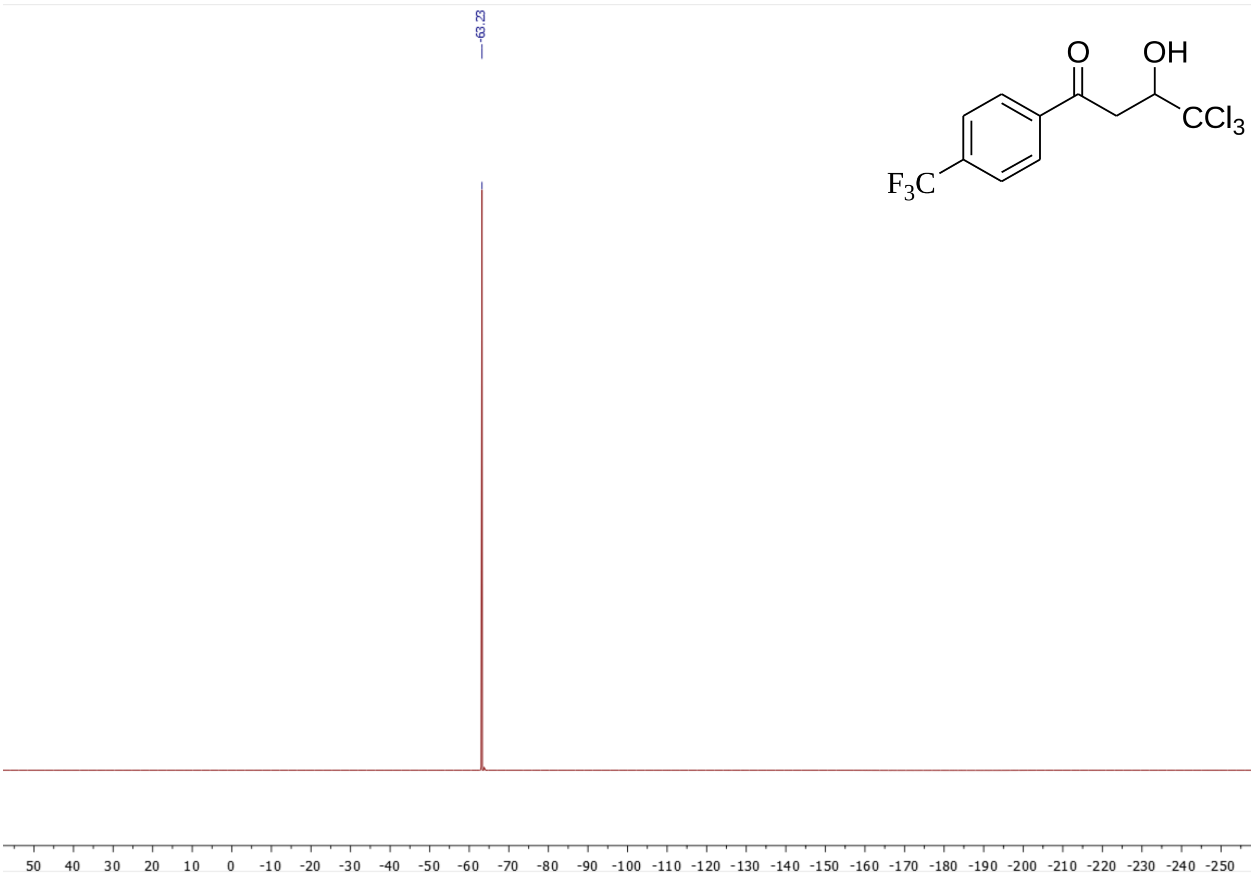

Figure S21. $^{19}F\{^{1}H\}$ NMR spectrum of the compound **1i** ($CDCl_3$, 376 MHz).

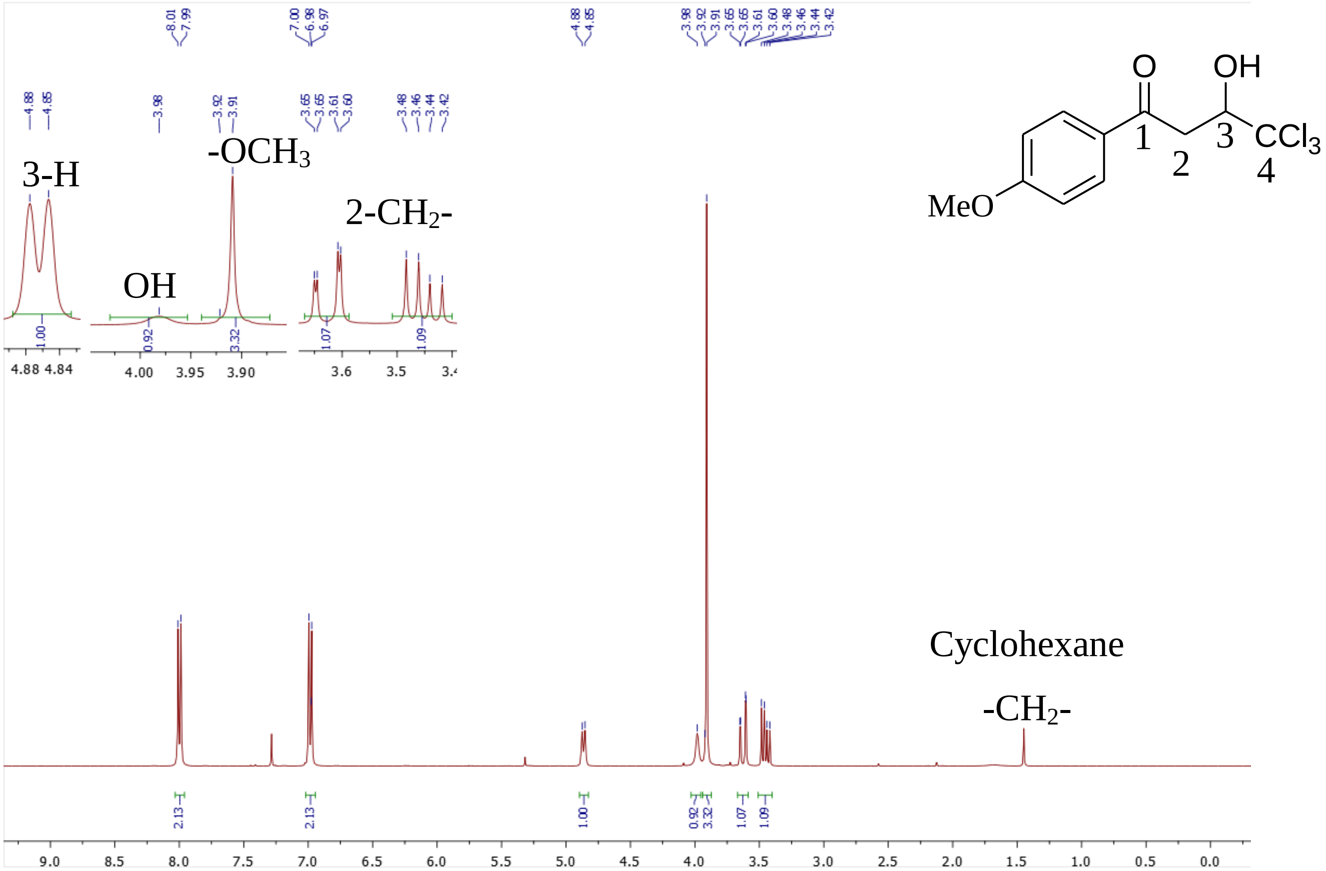

Figure S22. ^{1}H NMR spectrum of the compound **1j** ($CDCl_3$, 400 MHz).

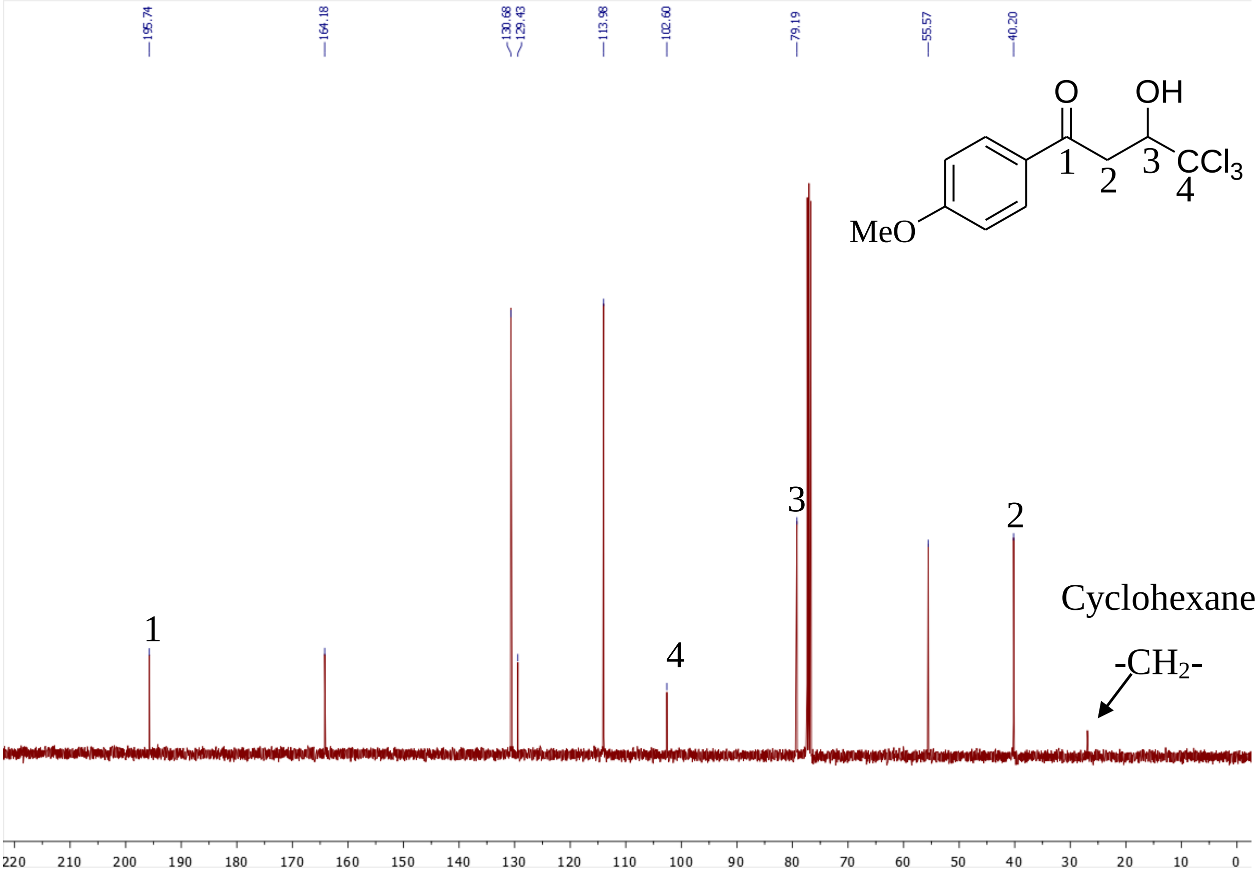

Figure S23. $^{13}C\{^{1}H\}$ NMR spectrum of the compound **1j** ($CDCl_3$, 101 MHz).

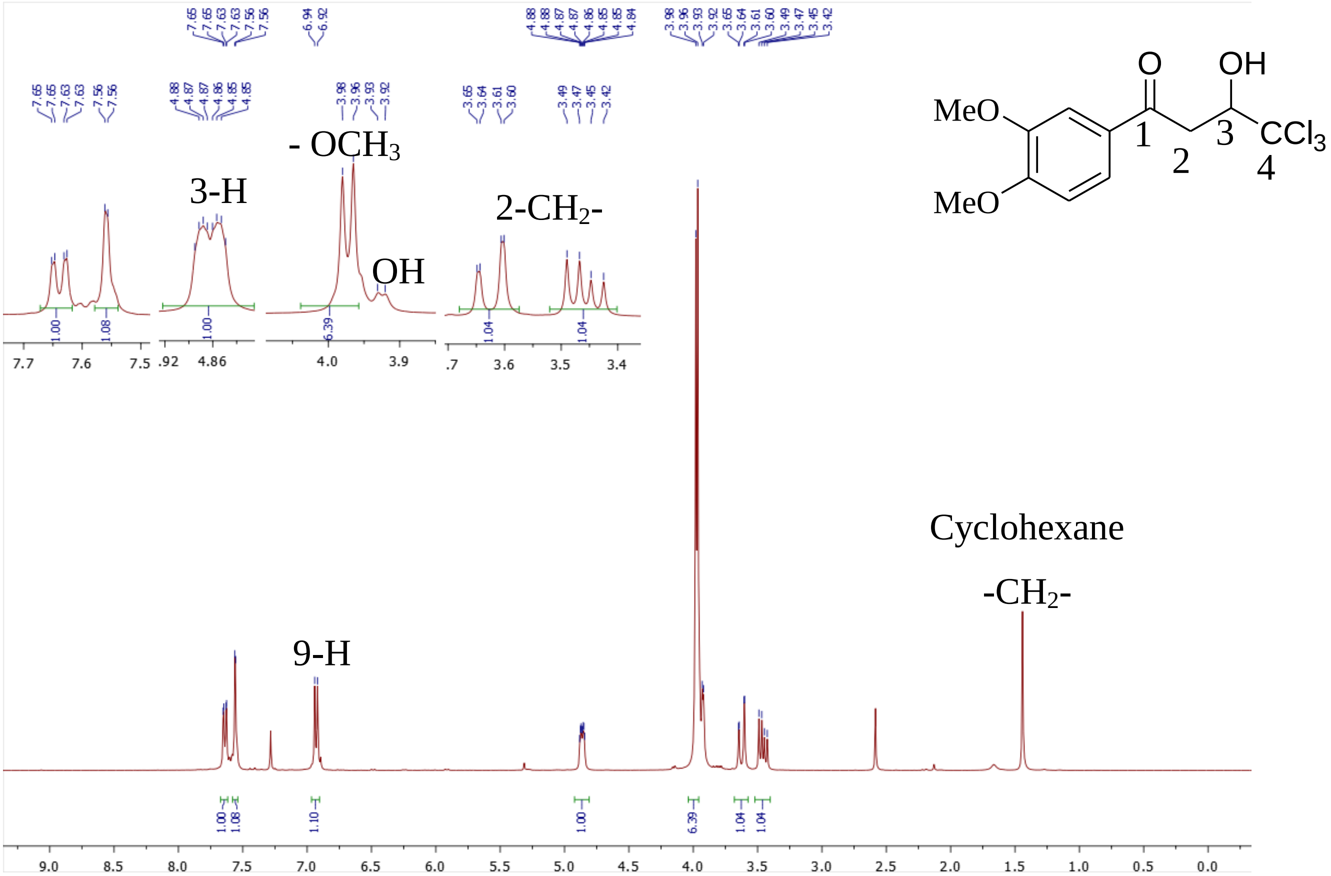

Figure S24. ^{1}H NMR spectrum of the compound **1k** ($CDCl_3$, 400 MHz).

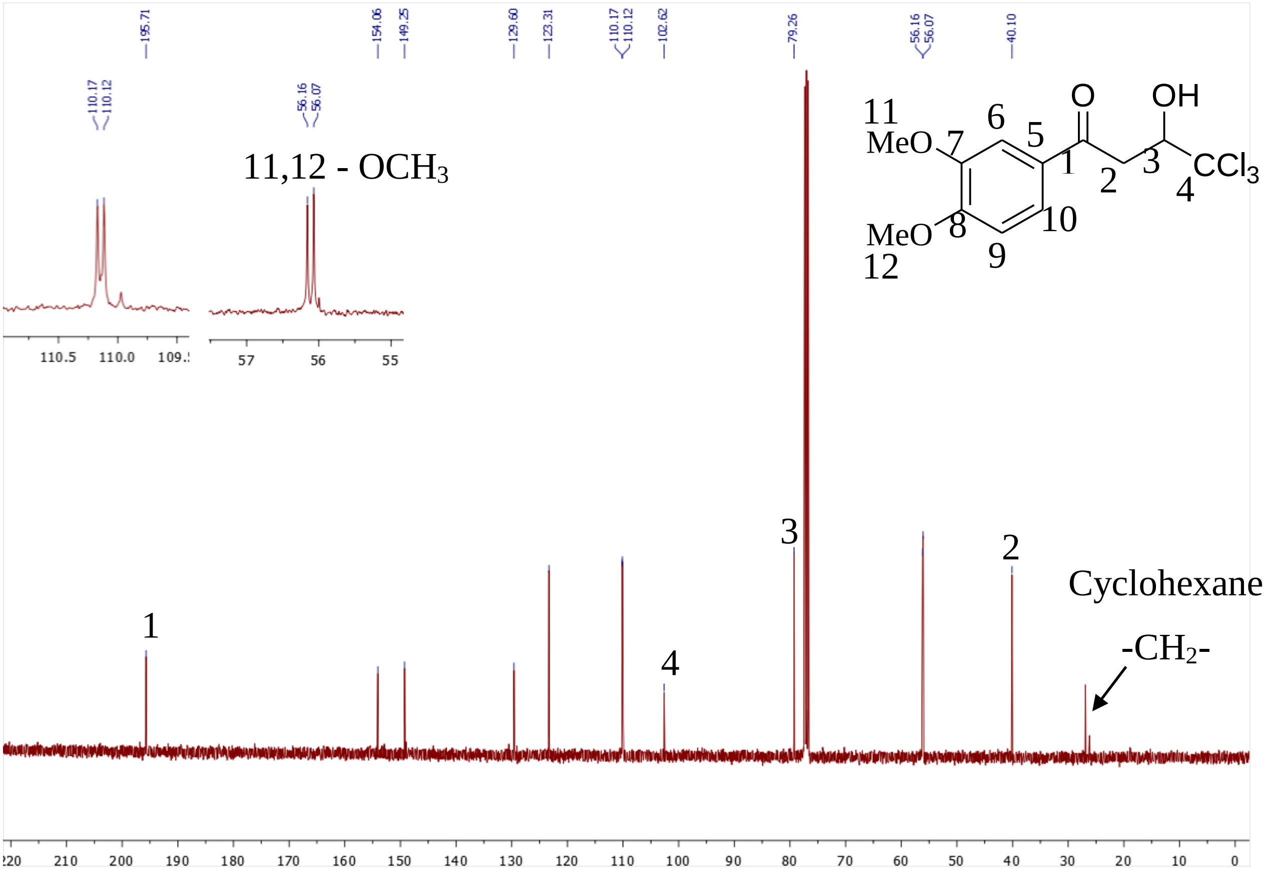

Figure S25. $^{13}C\{^1H\}$ NMR spectrum of the compound **1k** ($CDCl_3$, 101 MHz).

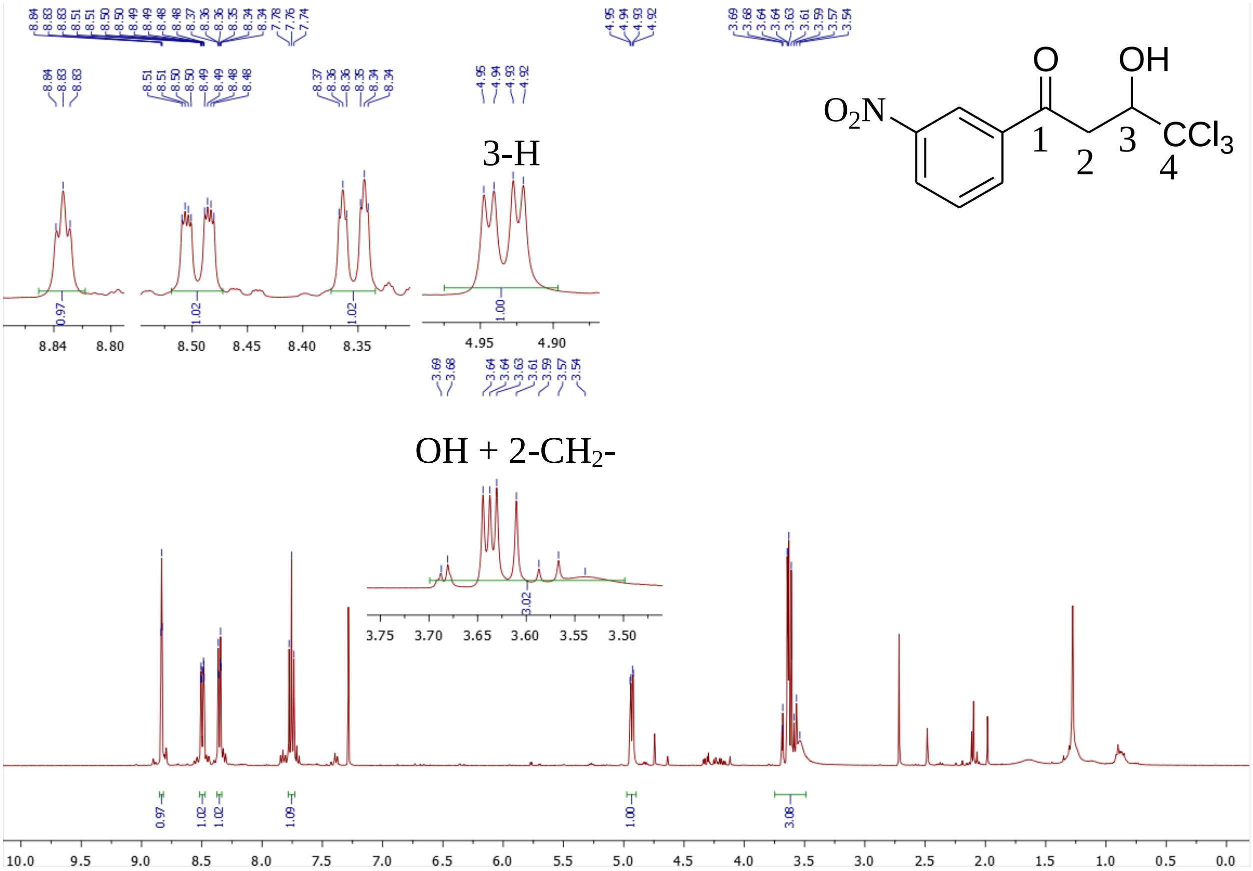

Figure S26. ^1H NMR spectrum of the compound **1l** ($CDCl_3$, 400 MHz).

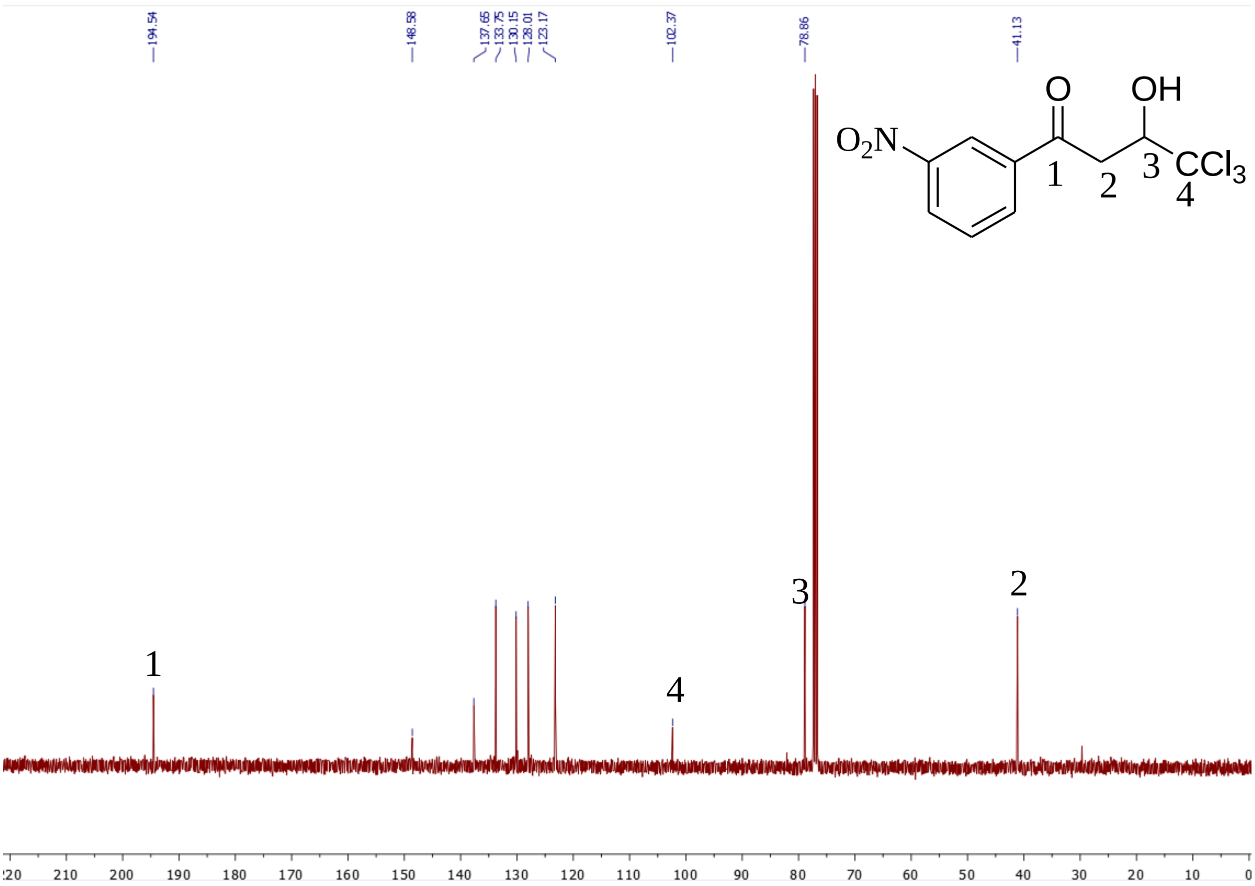

Figure S27. $^{13}C\{^{1}H\}$ NMR spectrum of the compound **1l** ($CDCl_3$, 101 MHz).

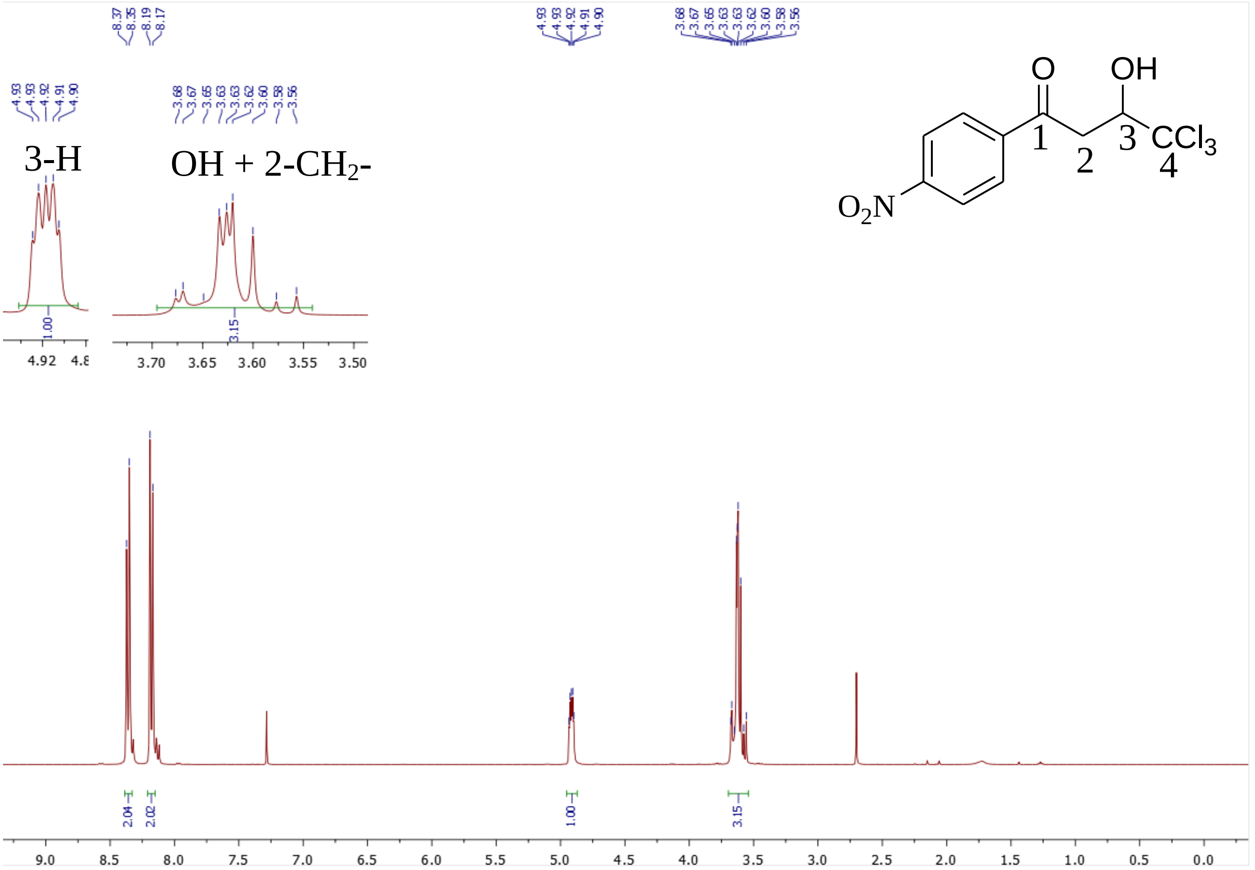

Figure S28. ^{1}H NMR spectrum of the compound **1m** ($CDCl_3$, 400 MHz).

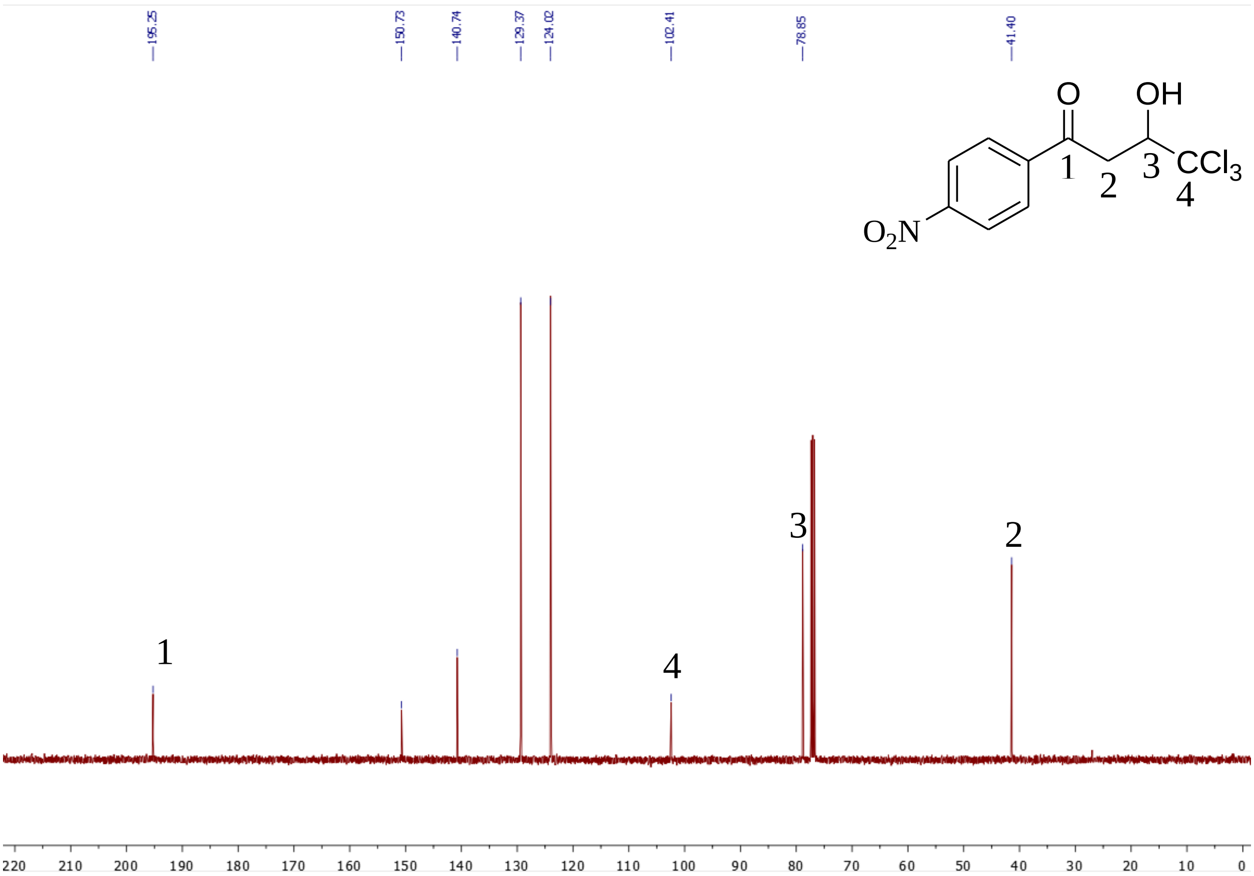

Figure S29. $^{13}C\{^{1}H\}$ NMR spectrum of the compound **1m** ($CDCl_3$, 101 MHz).

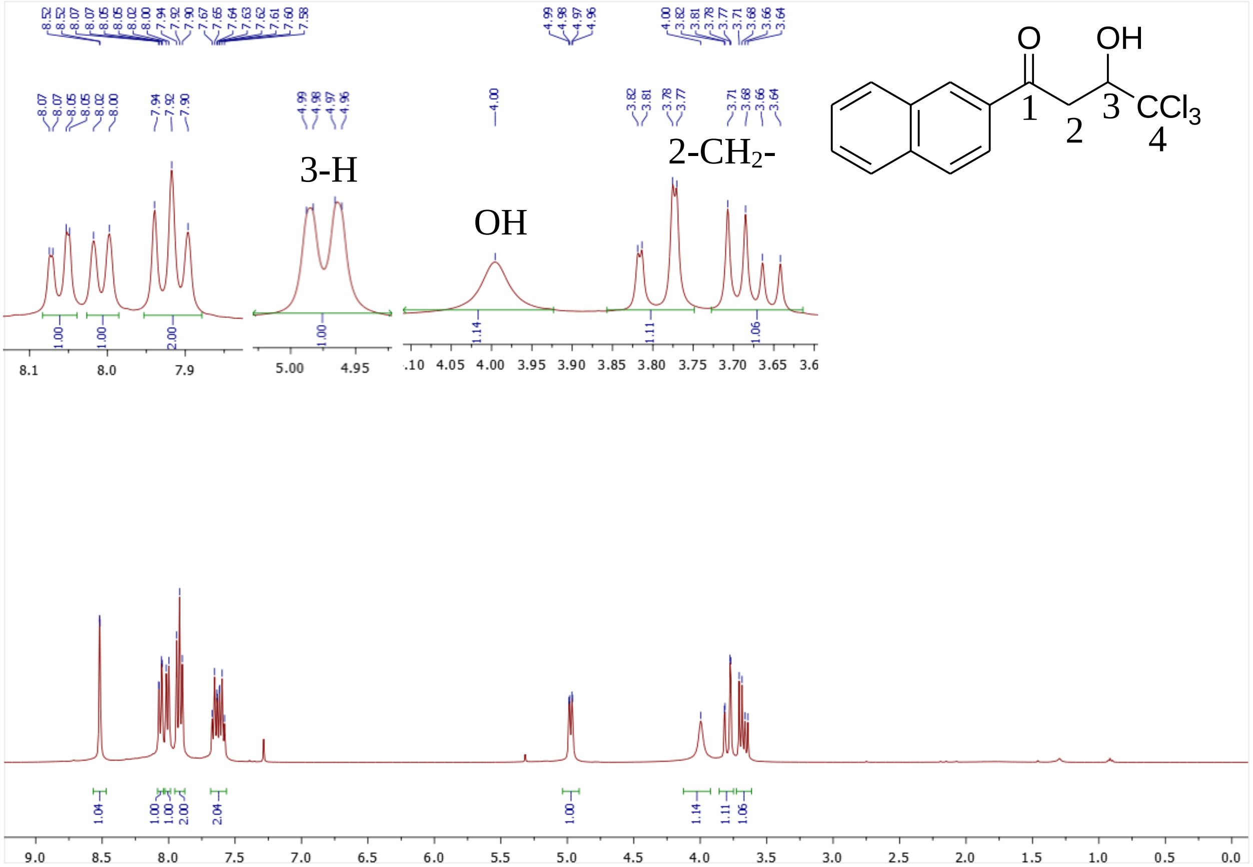

Figure S30. ^{1}H NMR spectrum of the compound **1n** ($CDCl_3$, 400 MHz).

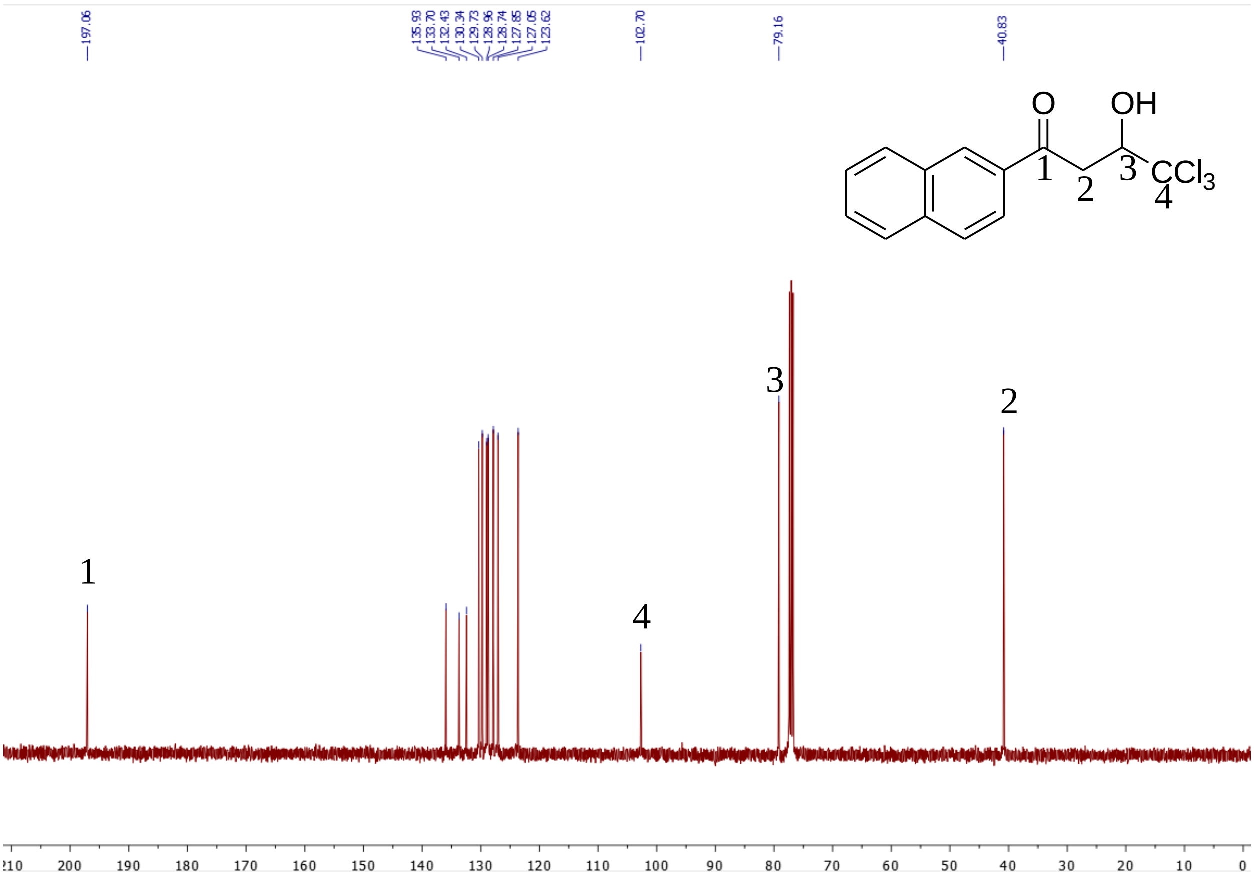

Figure S31. $^{13}C\{^{1}H\}$ NMR spectrum of the compound **1n** ($CDCl_3$, 101 MHz).

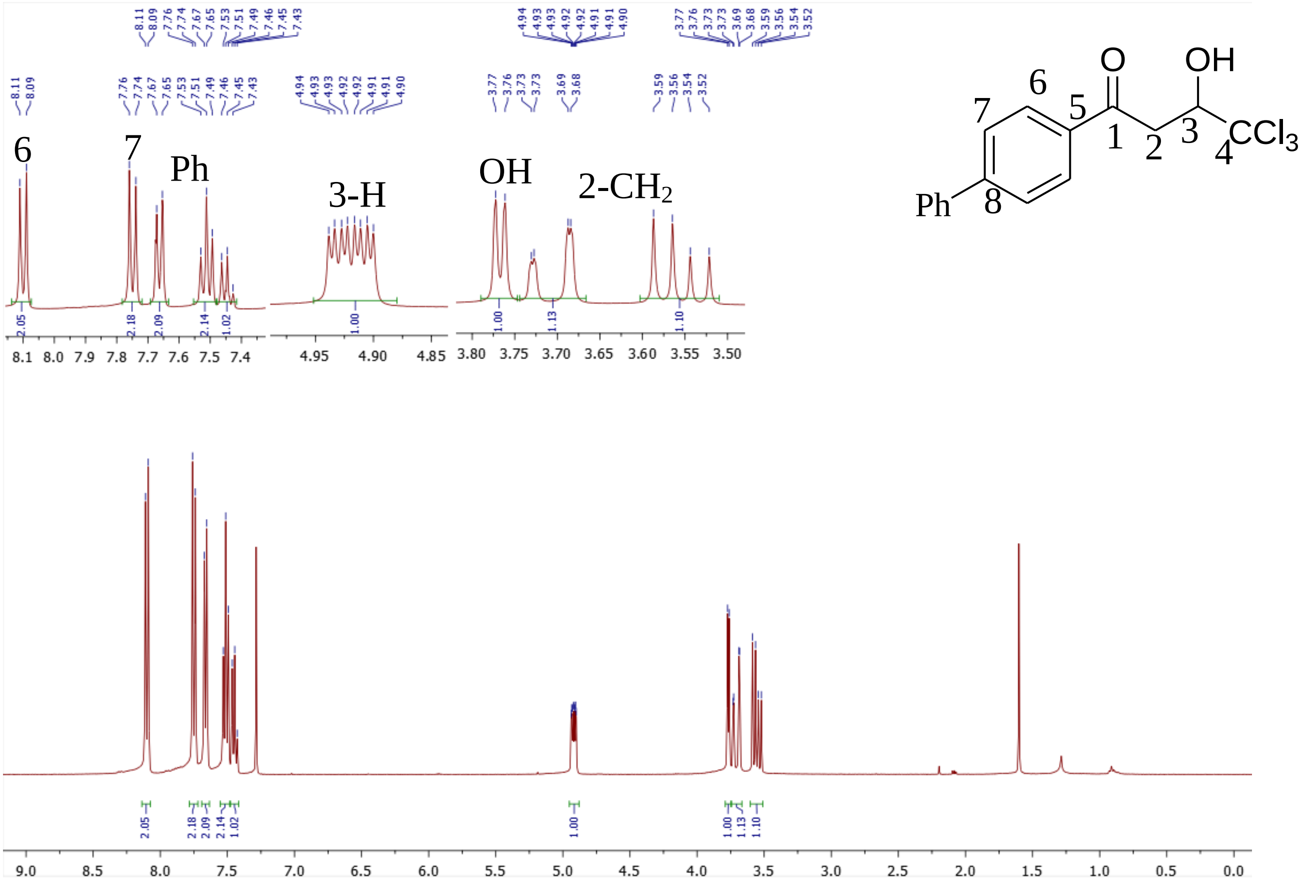

Figure S32. ^{1}H NMR spectrum of the compound **1o** ($CDCl_3$, 400 MHz).

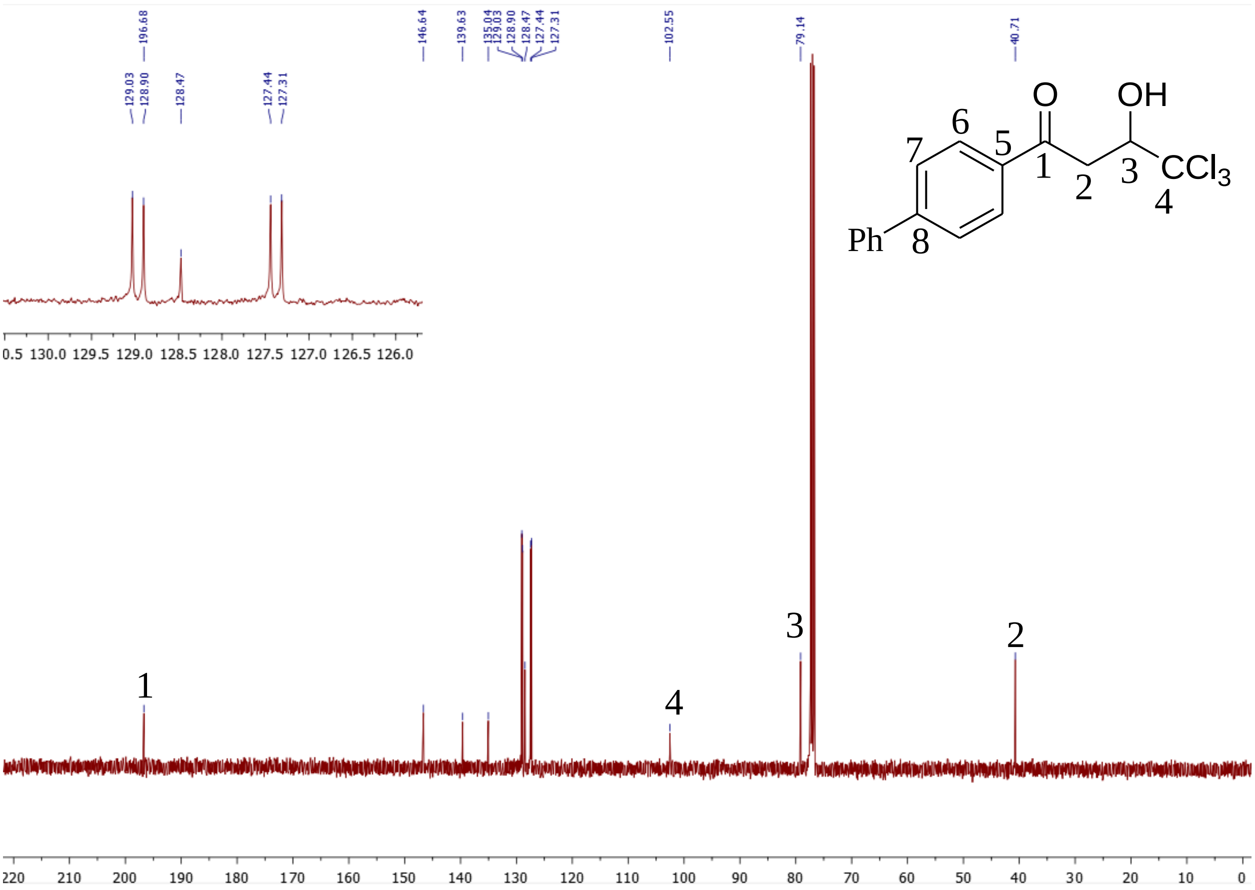

Figure S33. $^{13}C\{^1H\}$ NMR spectrum of the compound **1o** ($CDCl_3$, 101 MHz).

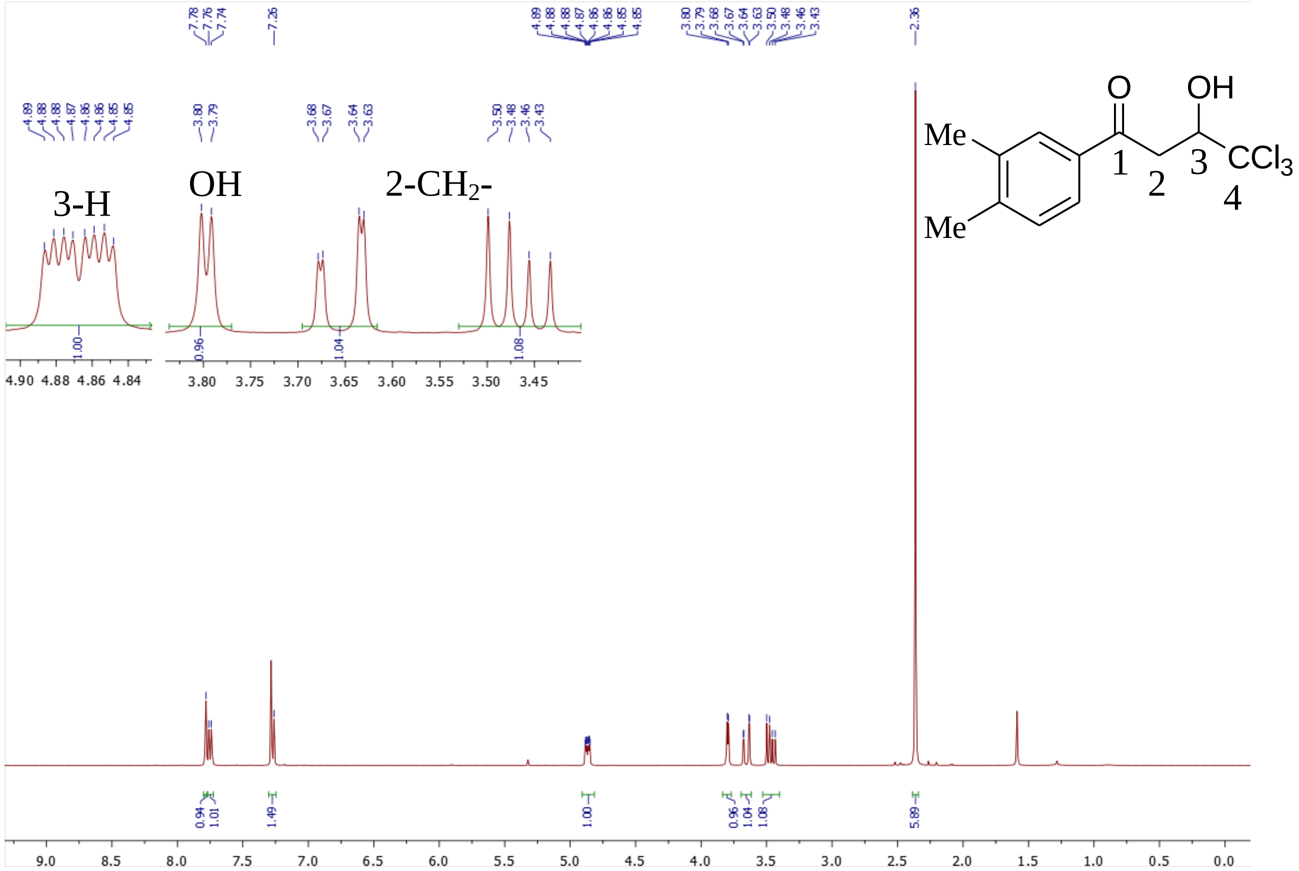

Figure S34. ^1H NMR spectrum of the compound **1p** ($CDCl_3$, 400 MHz).

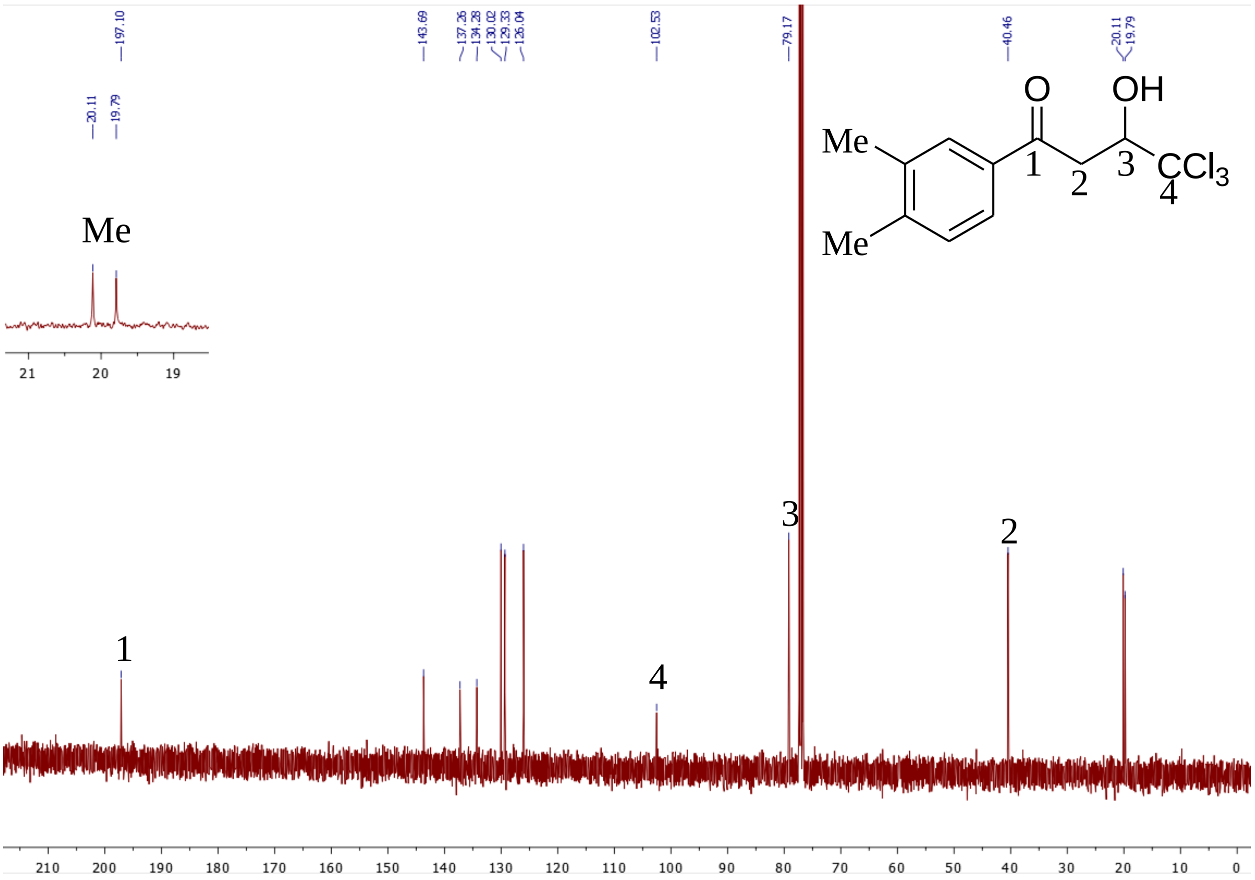

Figure S35. $^{13}C\{^{1}H\}$ NMR spectrum of the compound **1p** ($CDCl_3$, 101 MHz).

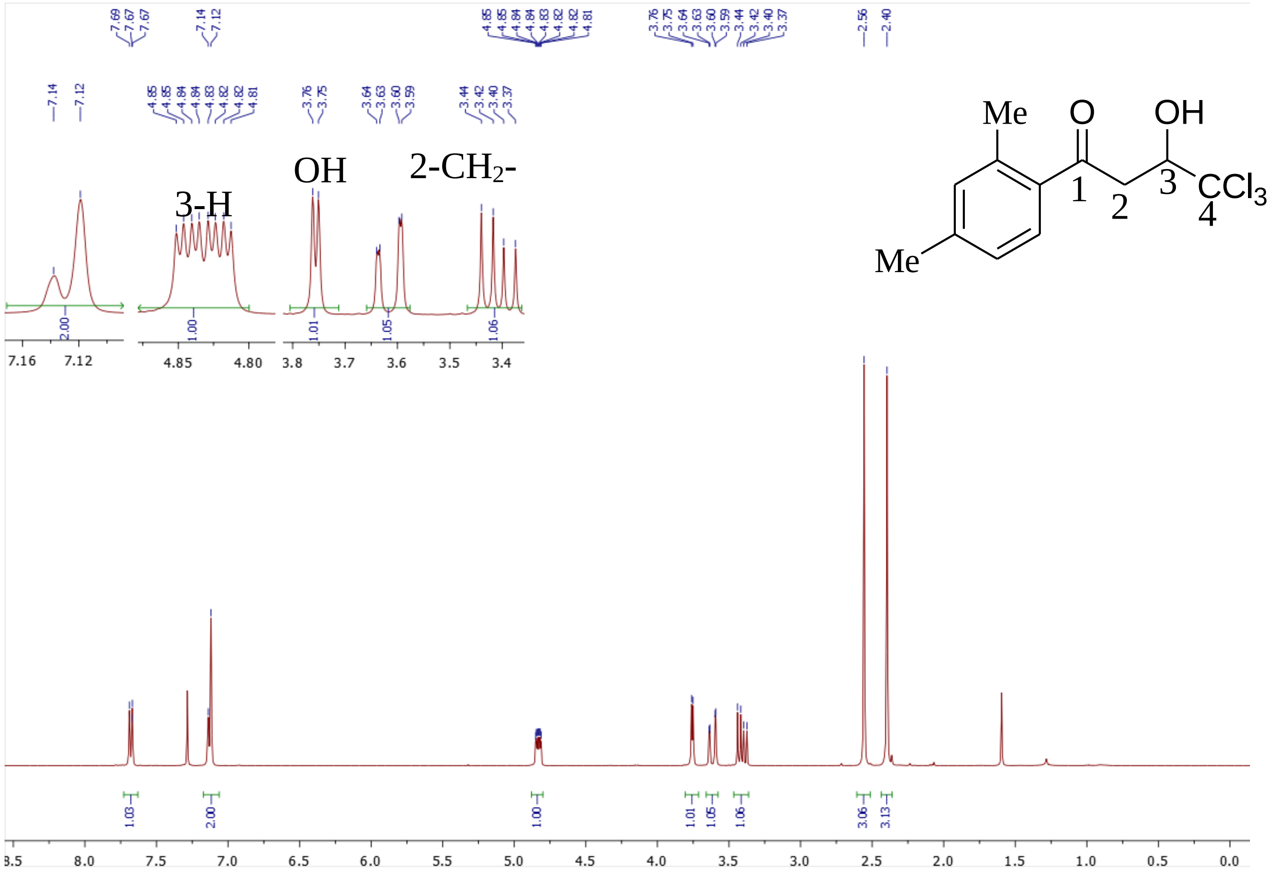

Figure S36. ^{1}H NMR spectrum of the compound **1q** ($CDCl_3$, 400 MHz).

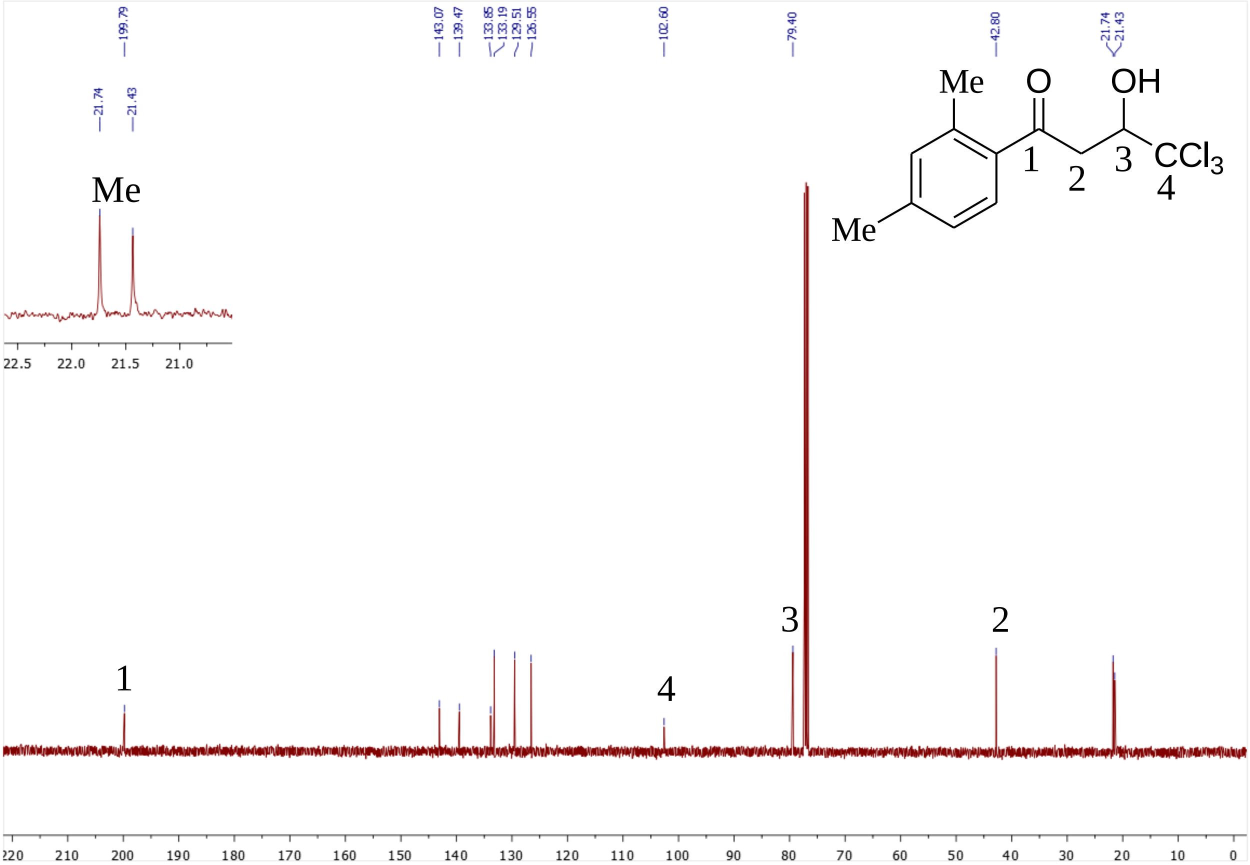

Figure S37. $^{13}C\{^{1}H\}$ NMR spectrum of the compound **1q** ($CDCl_3$, 101 MHz).

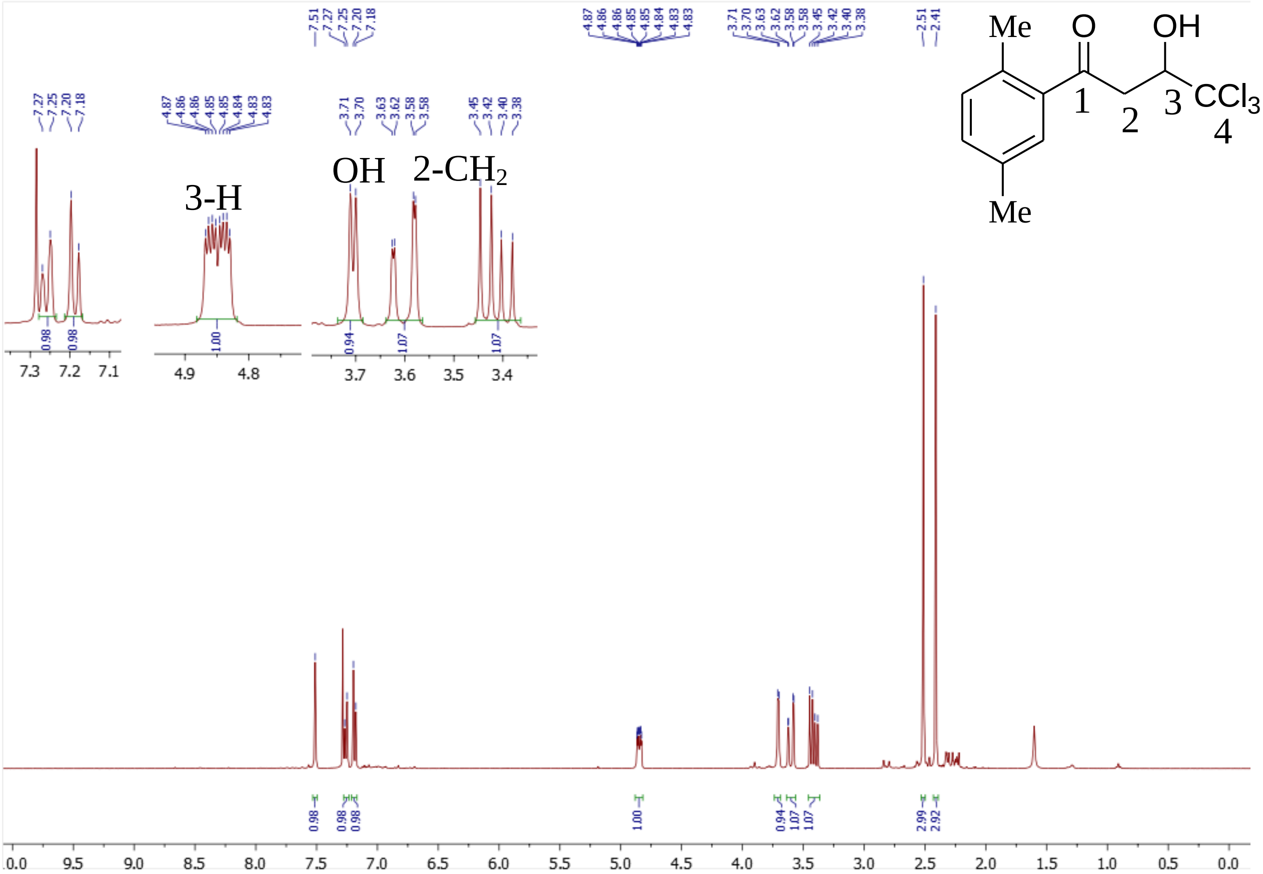

Figure S38. ^{1}H NMR spectrum of the compound **1r** ($CDCl_3$, 400 MHz).

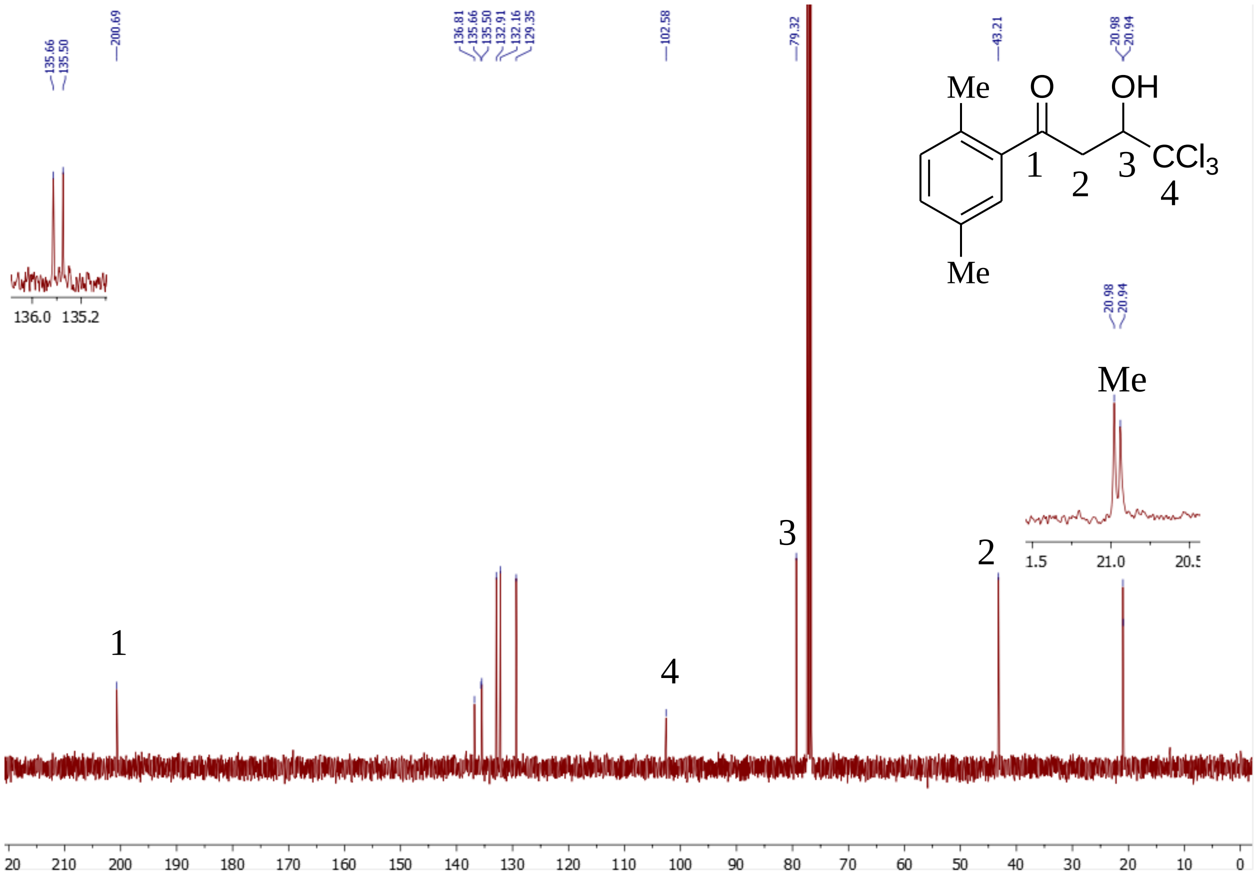

Figure S39. $^{13}C\{^{1}H\}$ NMR spectrum of the compound **1r** ($CDCl_3$, 101 MHz).

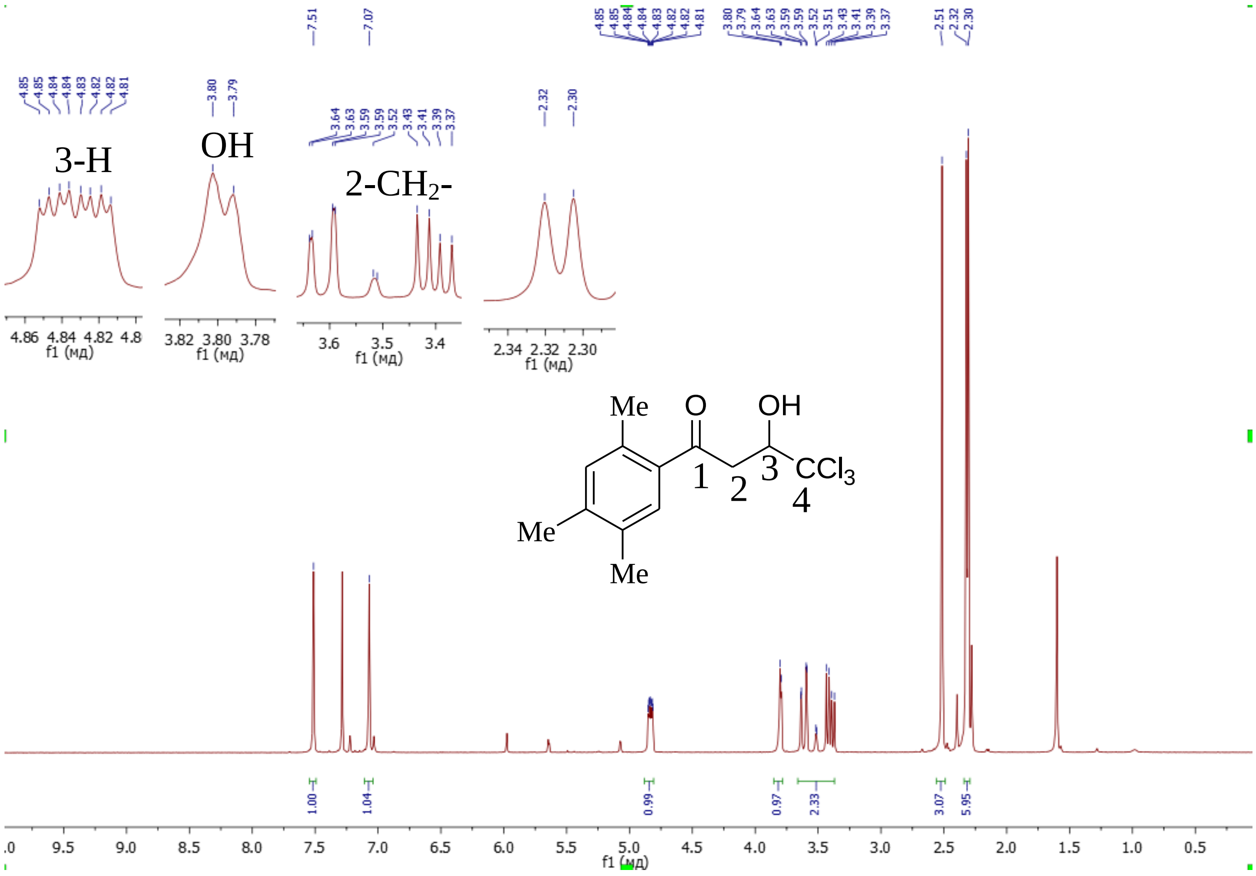

Figure S40. ^{1}H NMR spectrum of the compound **1s** ($CDCl_3$, 400 MHz).

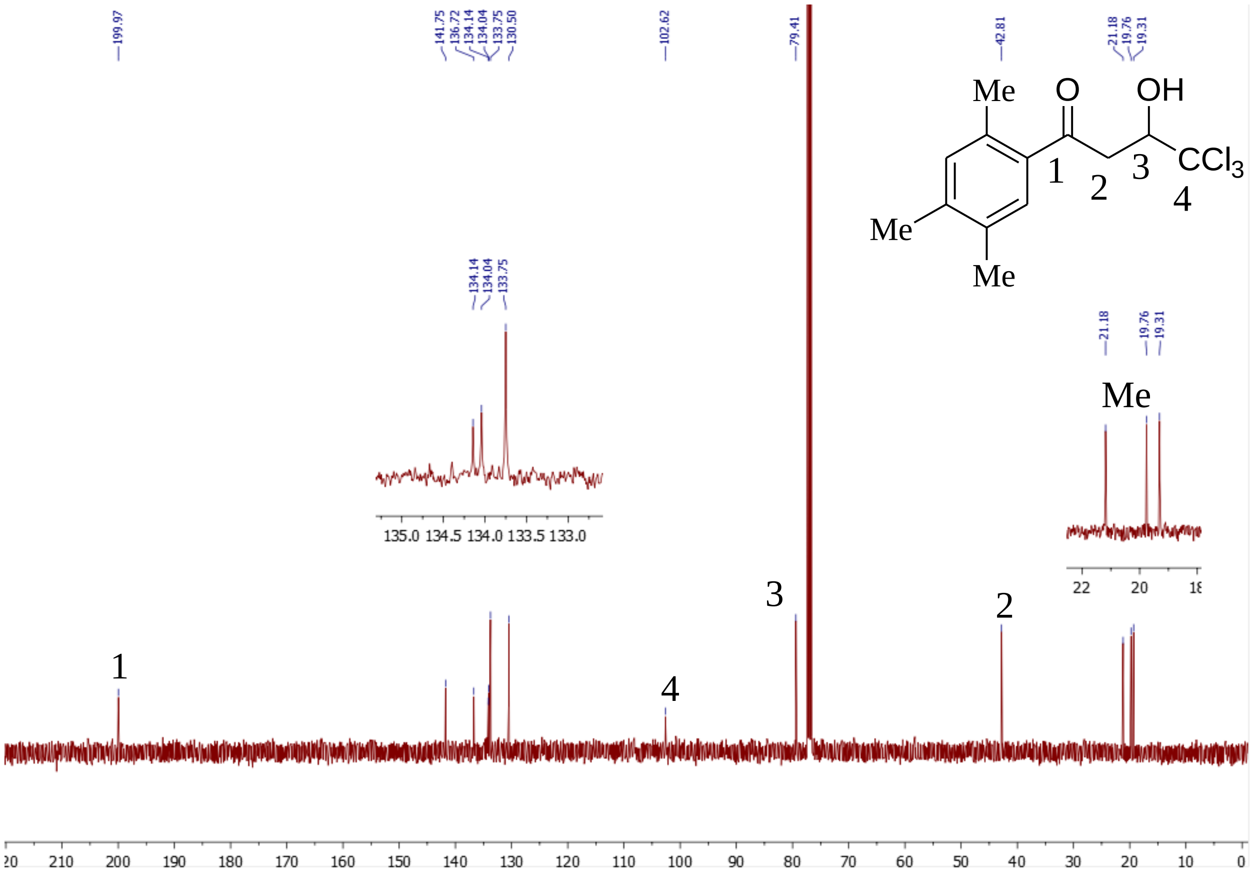

Figure S41. $^{13}C\{^1H\}$ NMR spectrum of the compound **1s** ($CDCl_3$, 101 MHz).

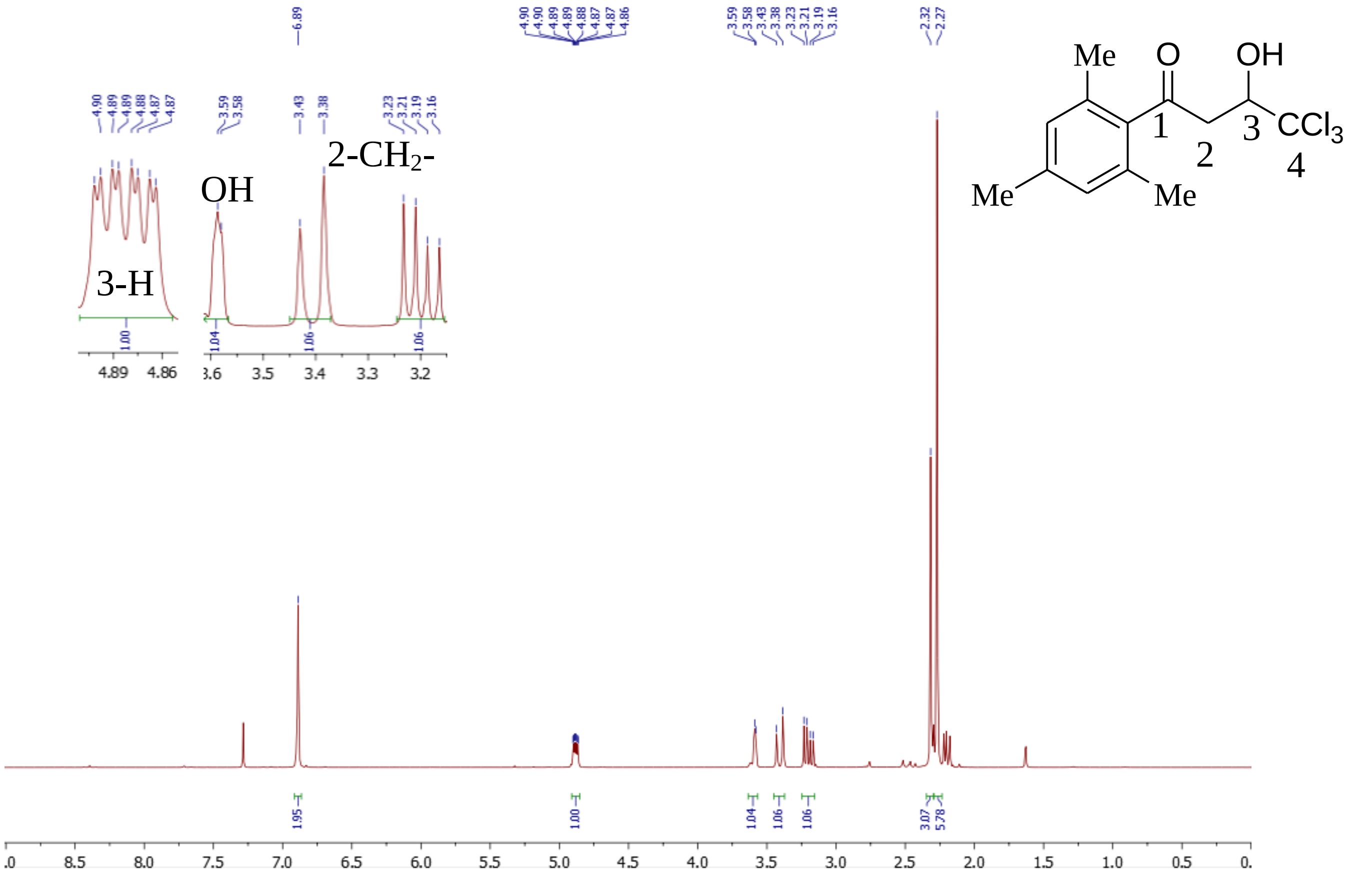

Figure S42. ^1H NMR spectrum of the compound **1t** ($CDCl_3$, 400 MHz).

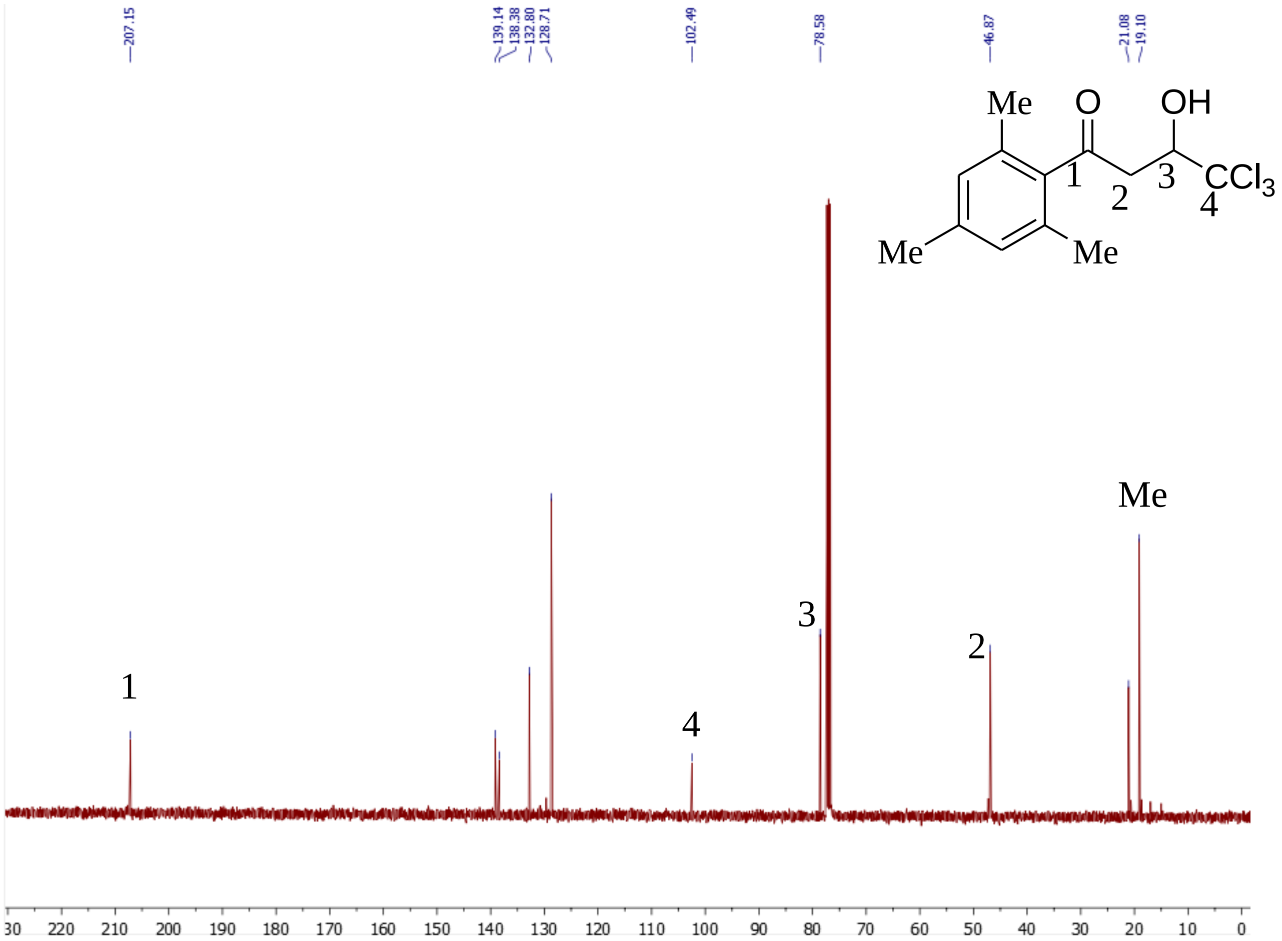

Figure S43. $^{13}C\{^{1}H\}$ NMR spectrum of the compound **1t** ($CDCl_3$, 101 MHz).

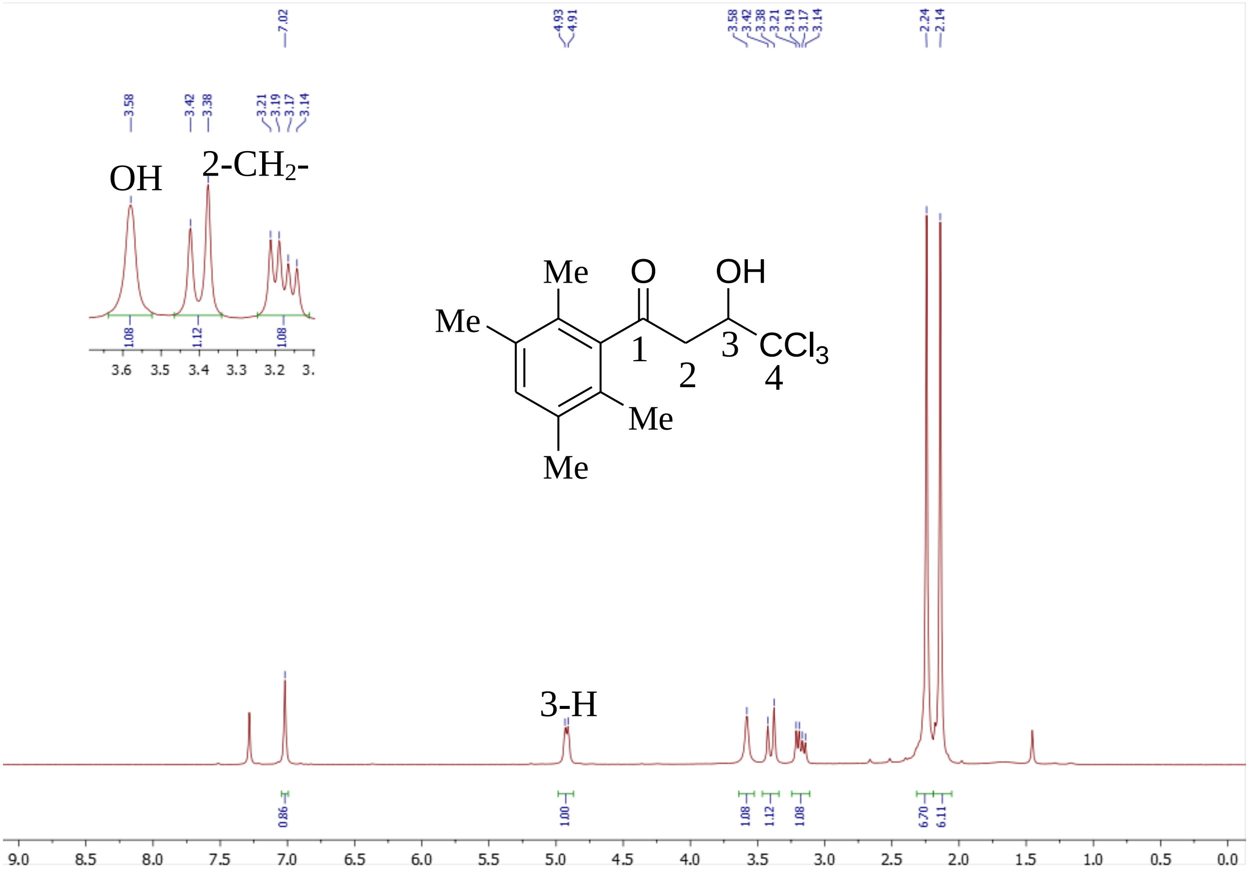

Figure S44. ^{1}H NMR spectrum of the compound **1u** ($CDCl_3$, 400 MHz).

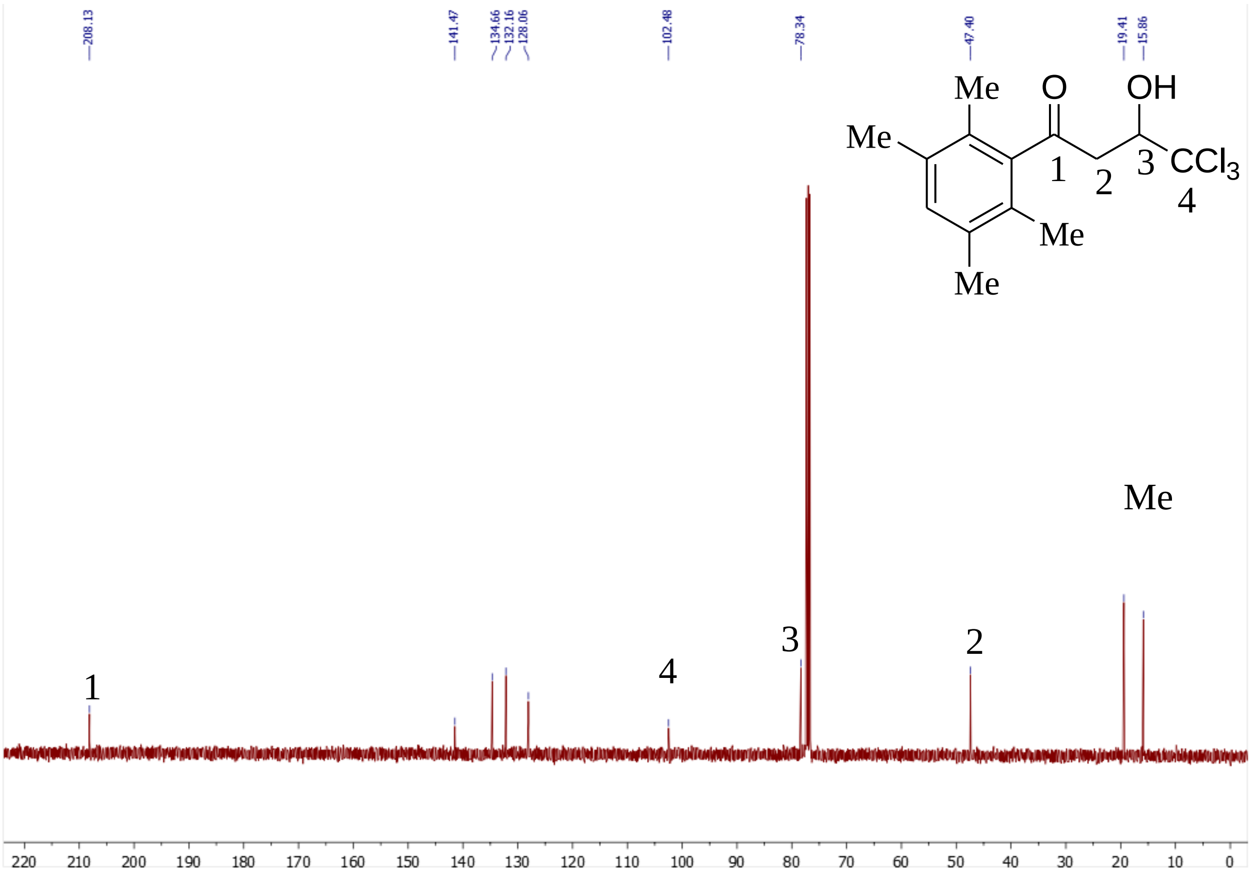

Figure S45. $^{13}C\{^{1}H\}$ NMR spectrum of the compound **1u** ($CDCl_3$, 101 MHz).

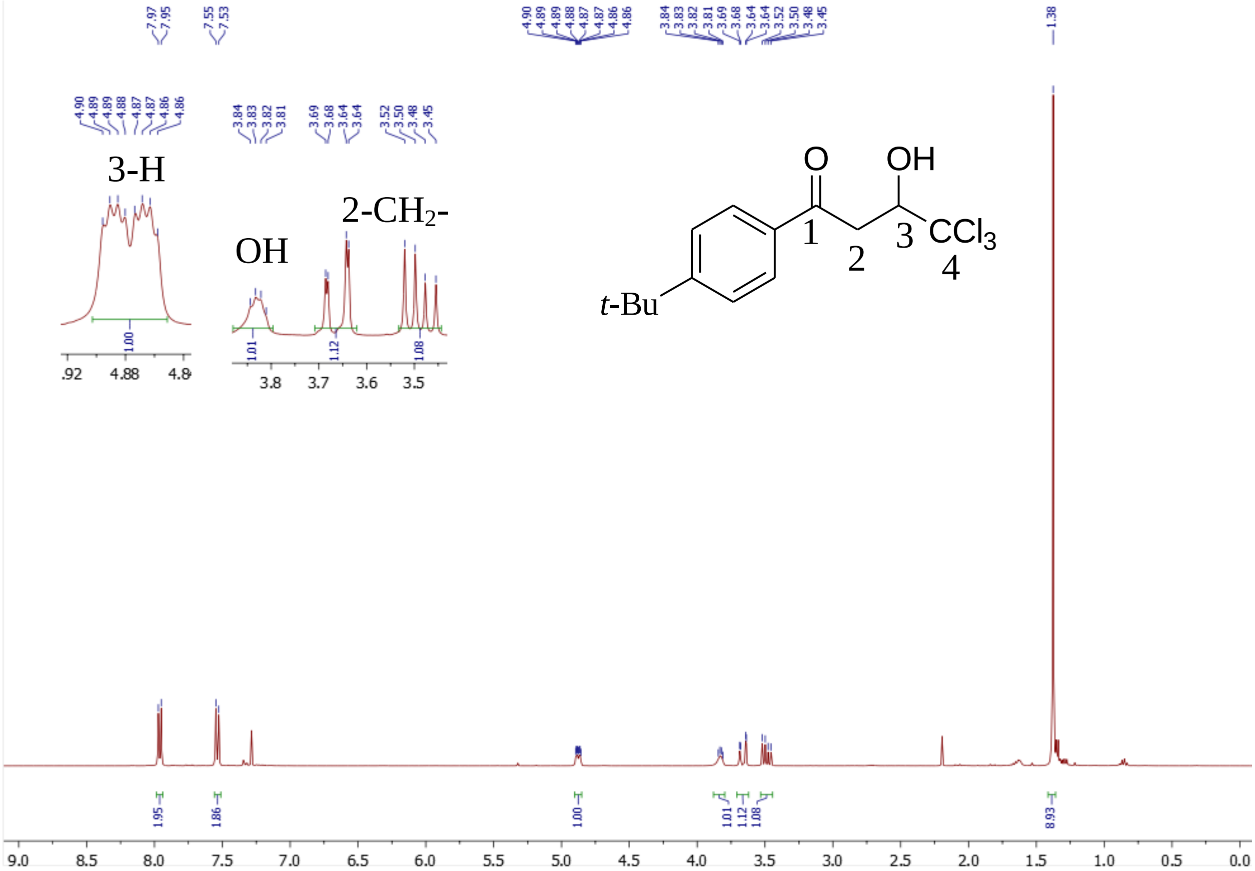

Figure S46. ^{1}H NMR spectrum of the compound **1v** ($CDCl_3$, 400 MHz).

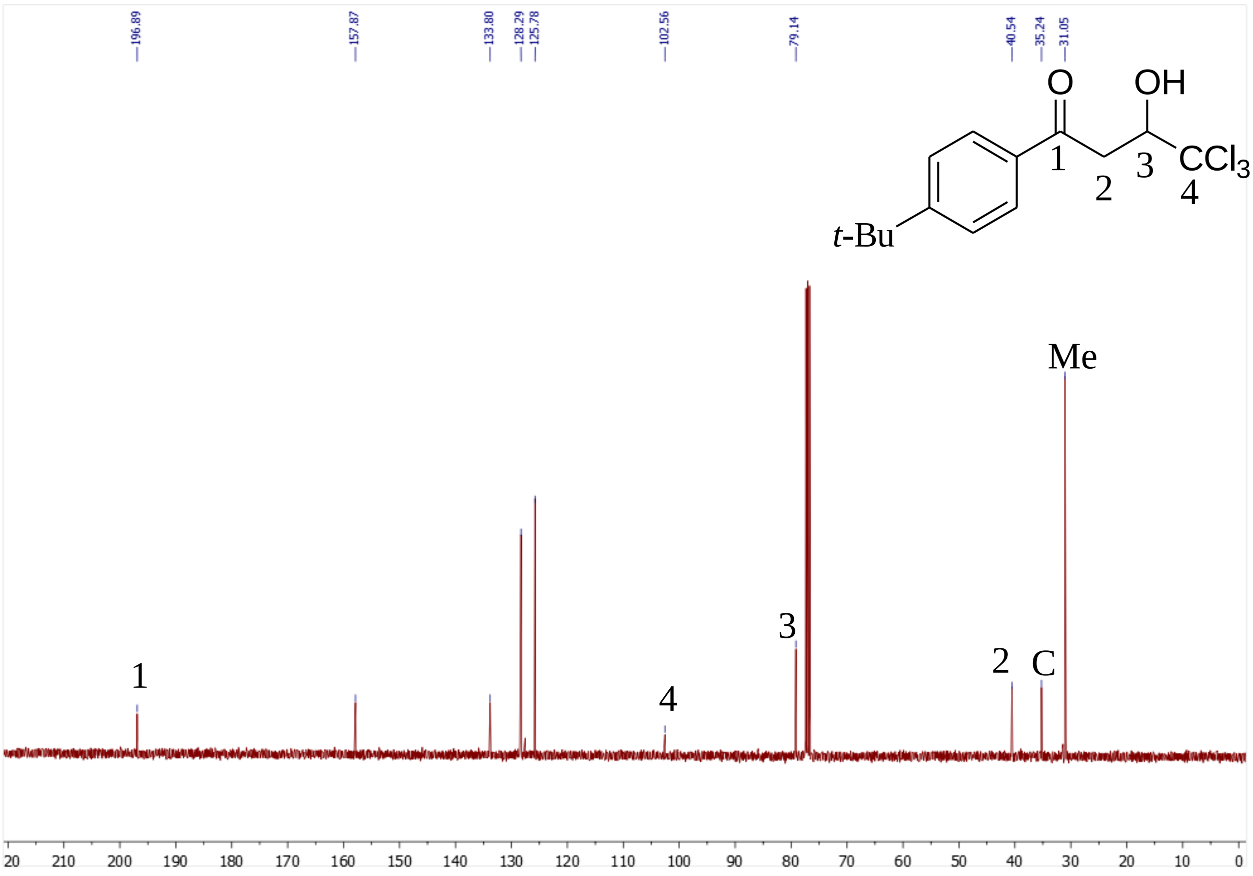

Figure S47. $^{13}C\{^1H\}$ NMR spectrum of the compound **1v** ($CDCl_3$, 101 MHz).

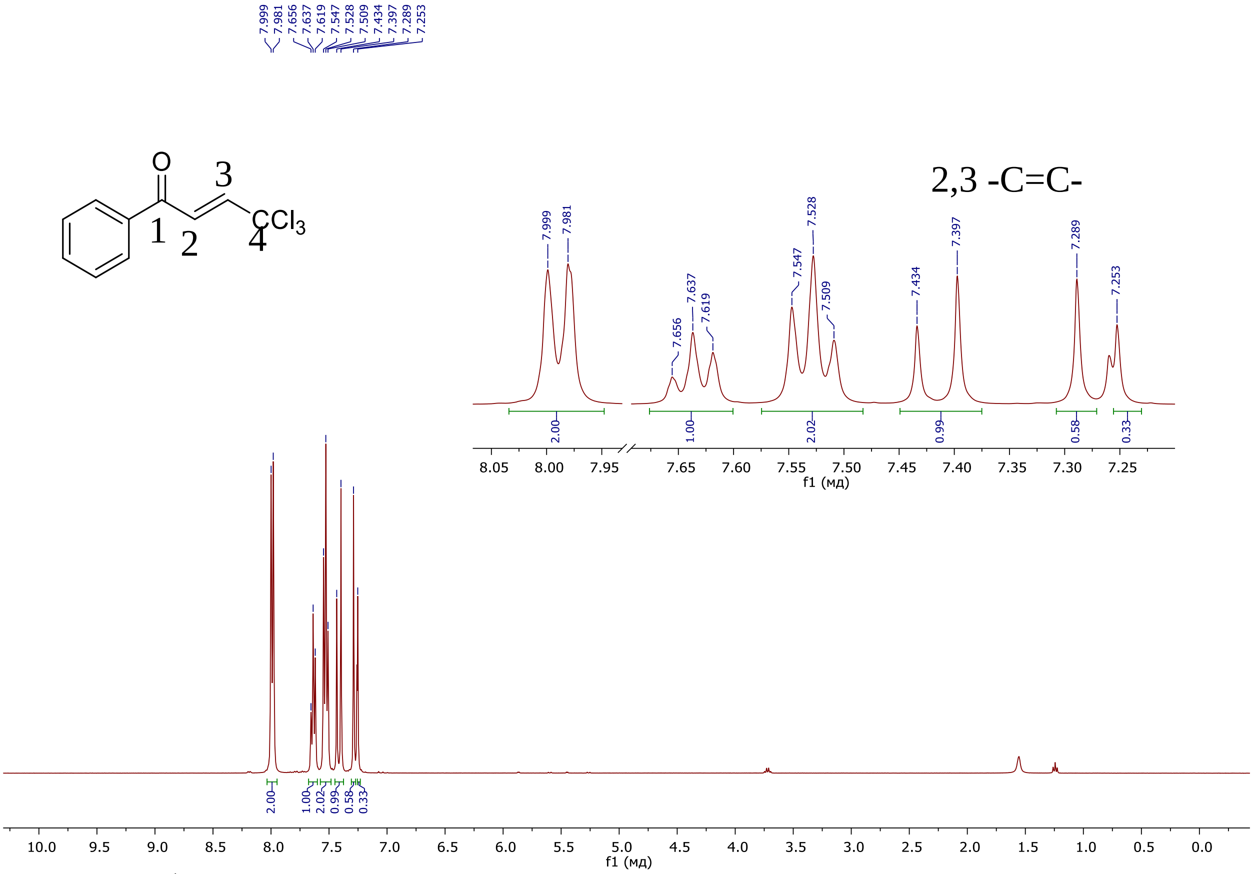

Figure S48. ^1H NMR spectrum of the compound **2a** ($CDCl_3$, 400 MHz).

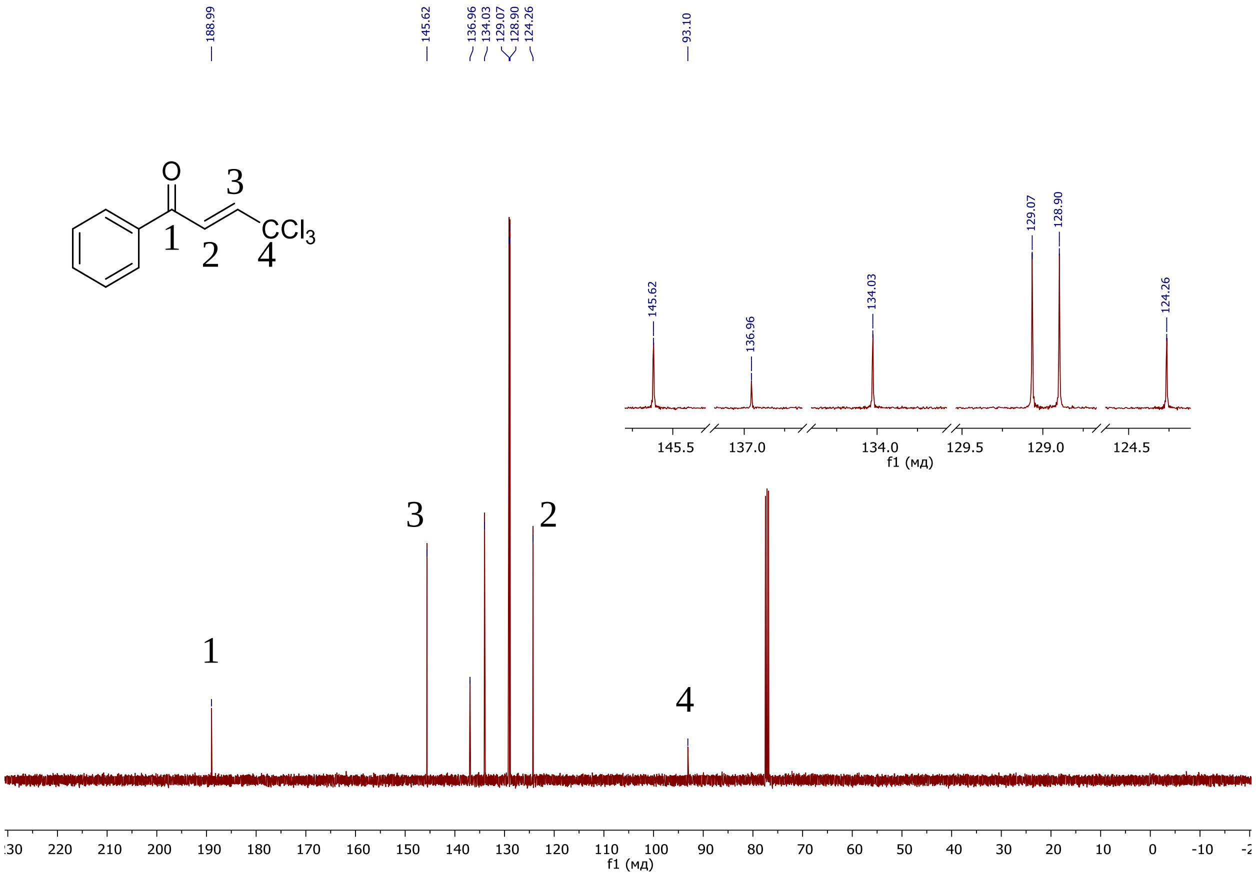

Figure S49. $^{13}C\{^1H\}$ NMR spectrum of the compound **2a** ($CDCl_3$, 101 MHz).

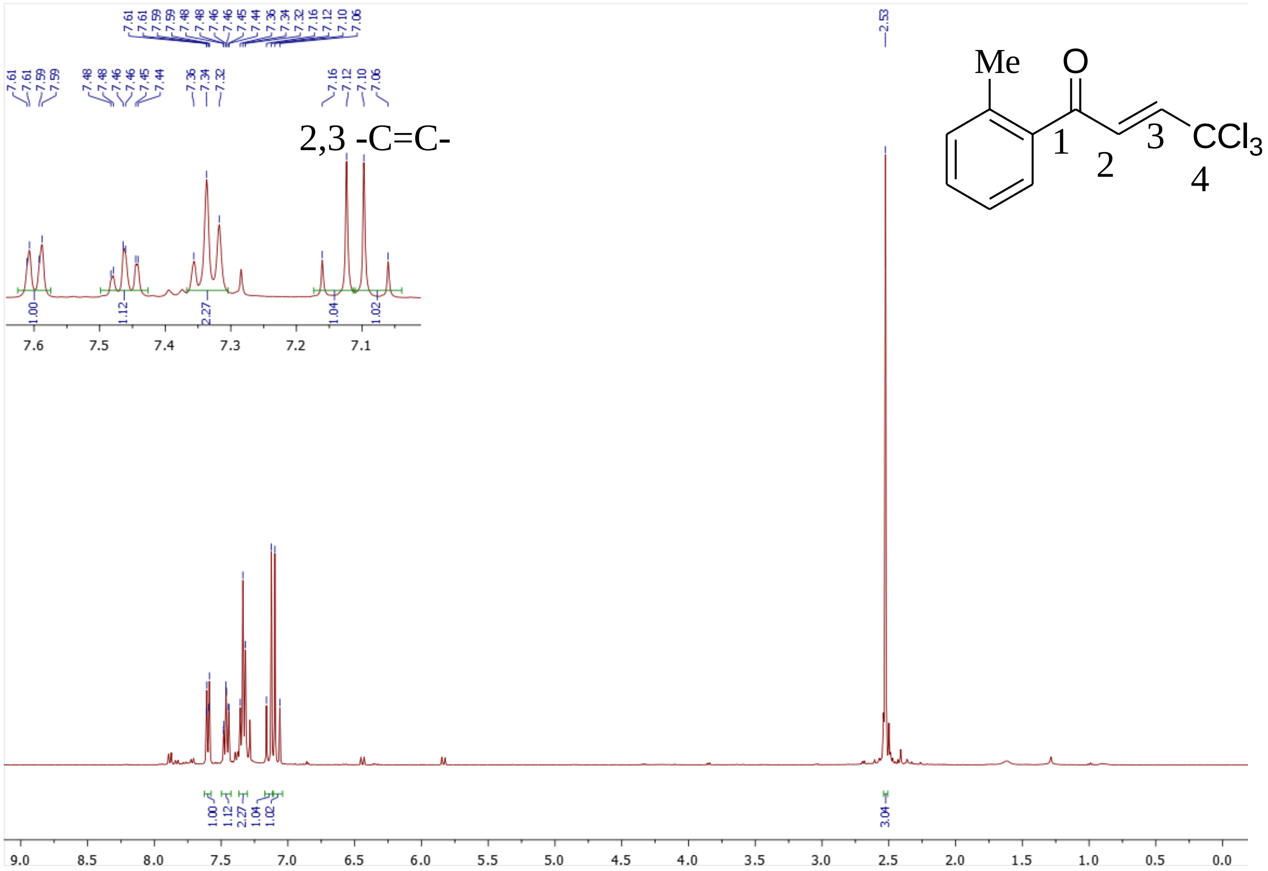

Figure S50. ^1H NMR spectrum of the compound **2b** ($CDCl_3$, 400 MHz).

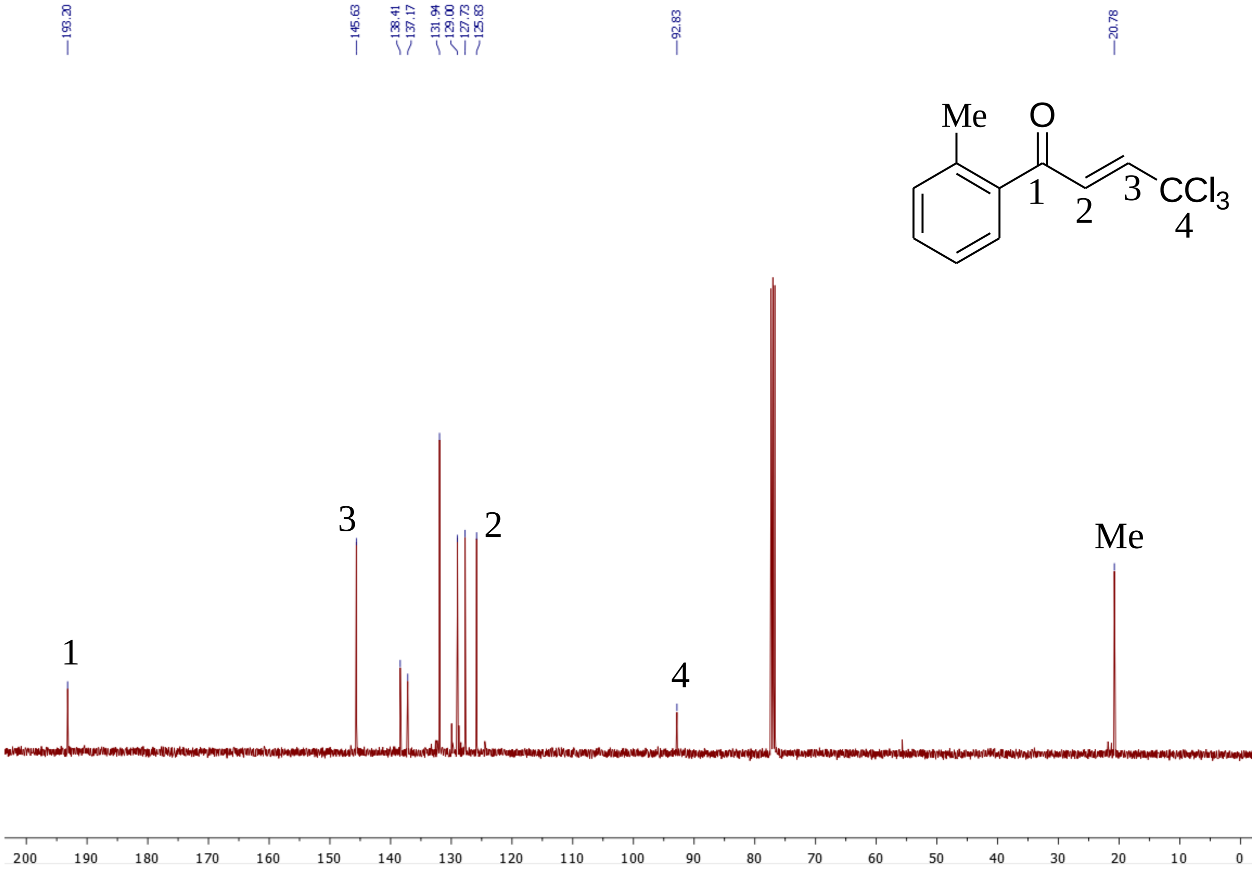

Figure S51. $^{13}C\{^{1}H\}$ NMR spectrum of the compound **2b** ($CDCl_3$, 101 MHz).

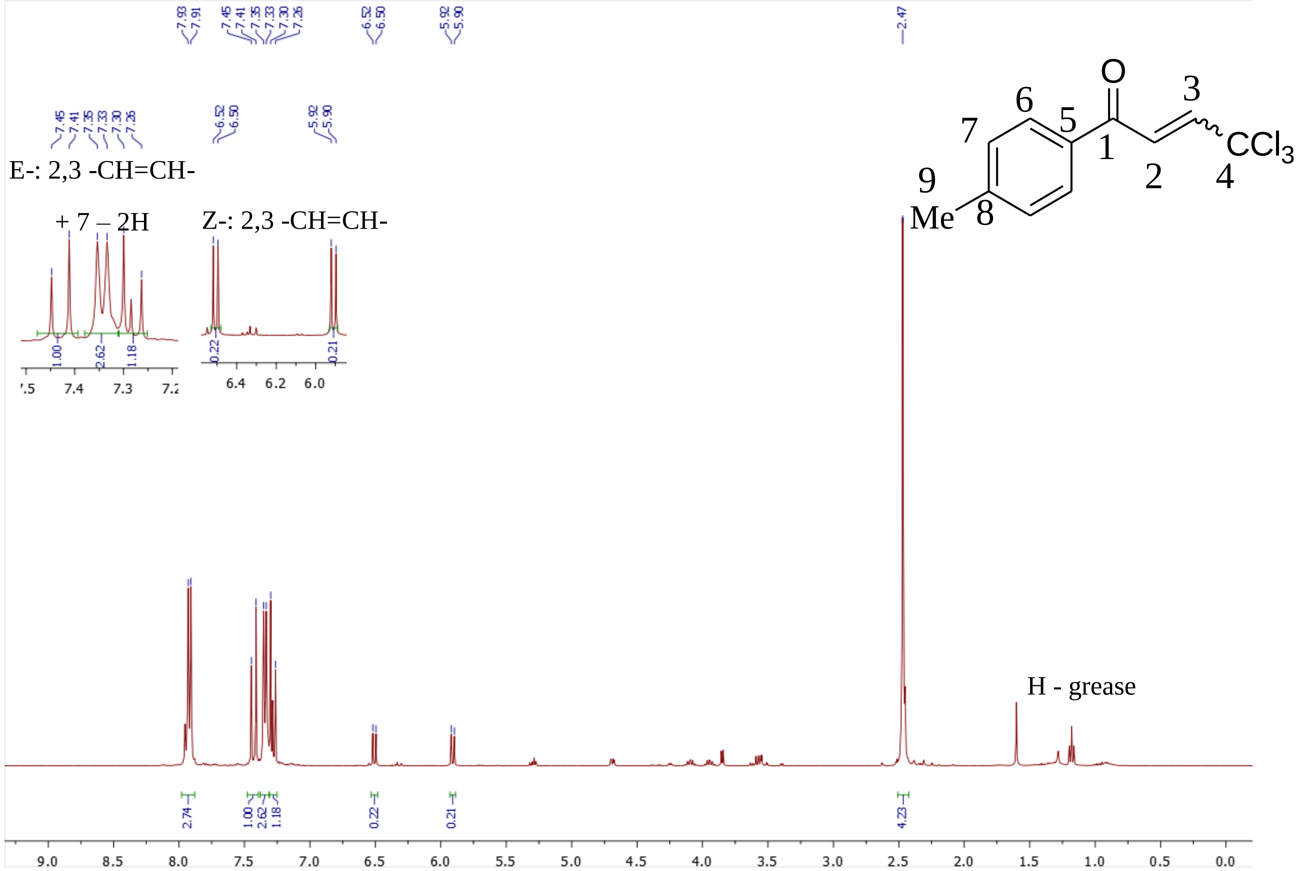

Figure S52. ^{1}H NMR spectrum of the compound **2c** ($CDCl_3$, 400 MHz).

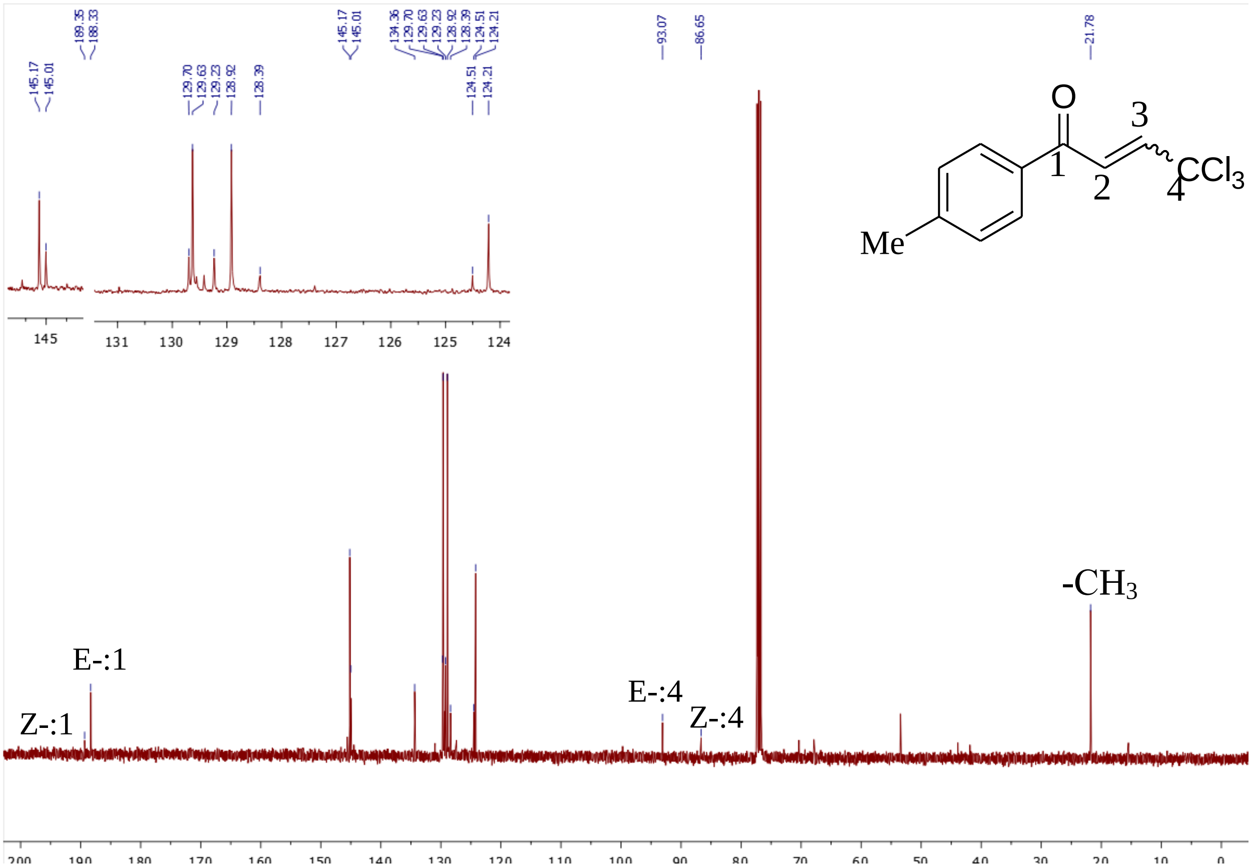

Figure S53. $^{13}C\{^{1}H\}$ NMR spectrum of the compound **2c** ($CDCl_3$, 101 MHz).

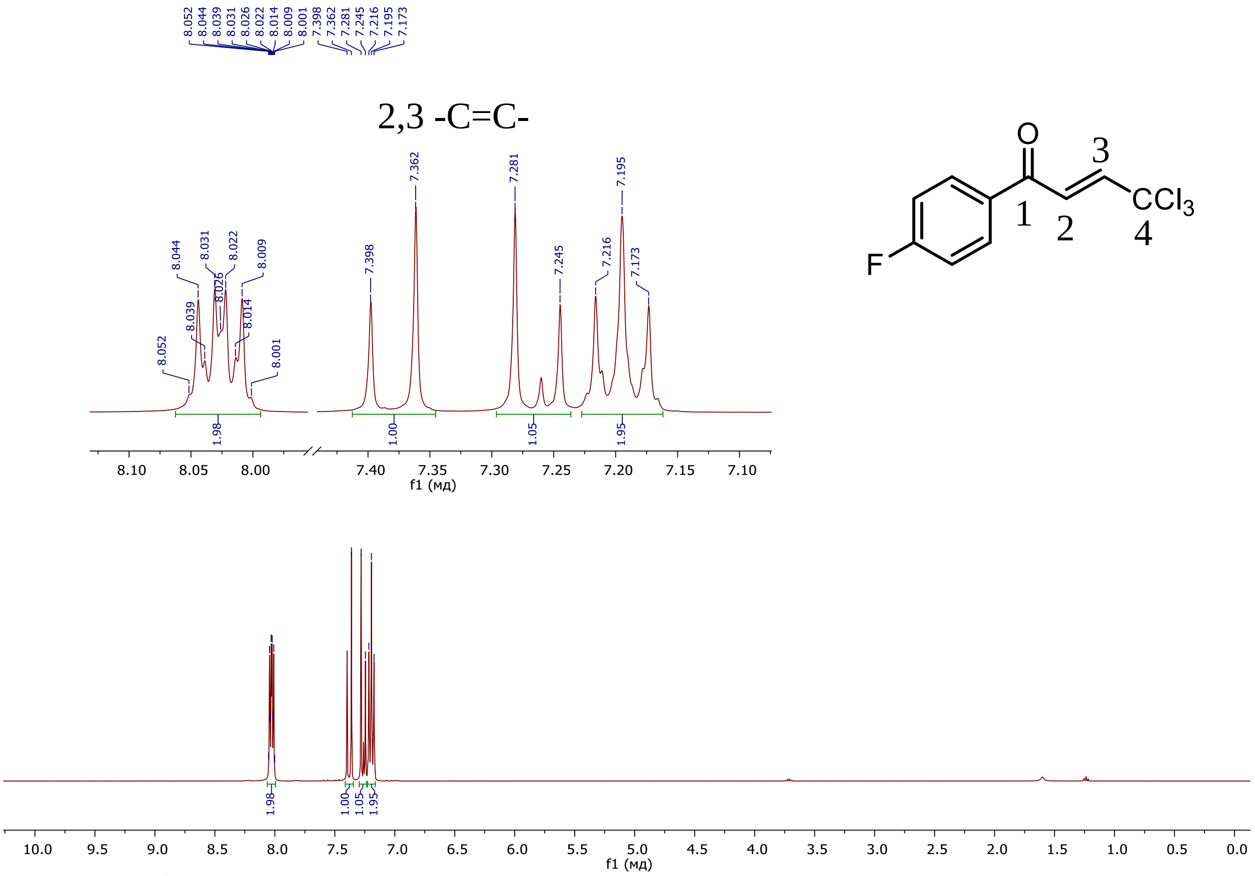

Figure S54. ^{1}H NMR spectrum of the compound **2d** ($CDCl_3$, 400 MHz).

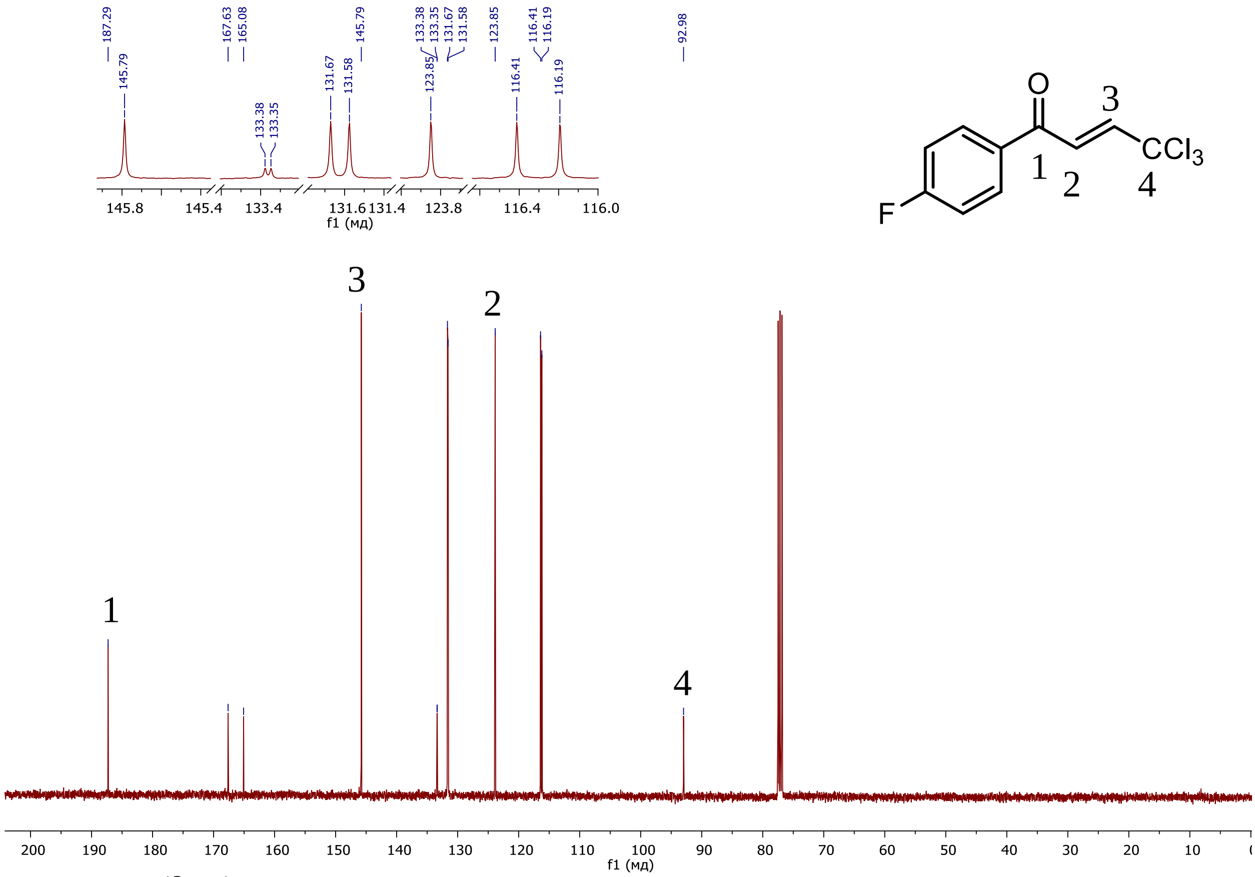

Figure S55. $^{13}C\{^{1}H\}$ NMR spectrum of the compound **2d** ($CDCl_3$, 101 MHz).

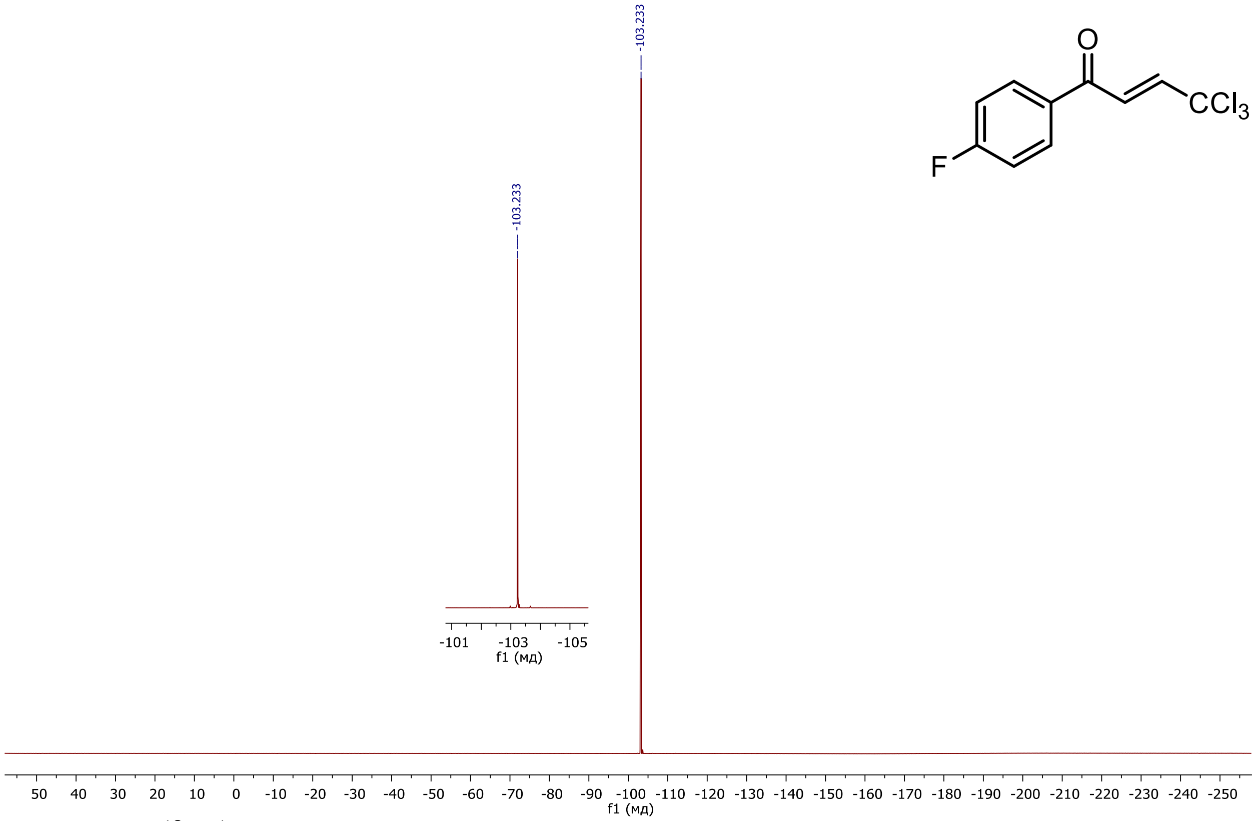

Figure S56. $^{19}F\{^{1}H\}$ NMR spectrum of the compound **2d** ($CDCl_3$, 376 MHz).

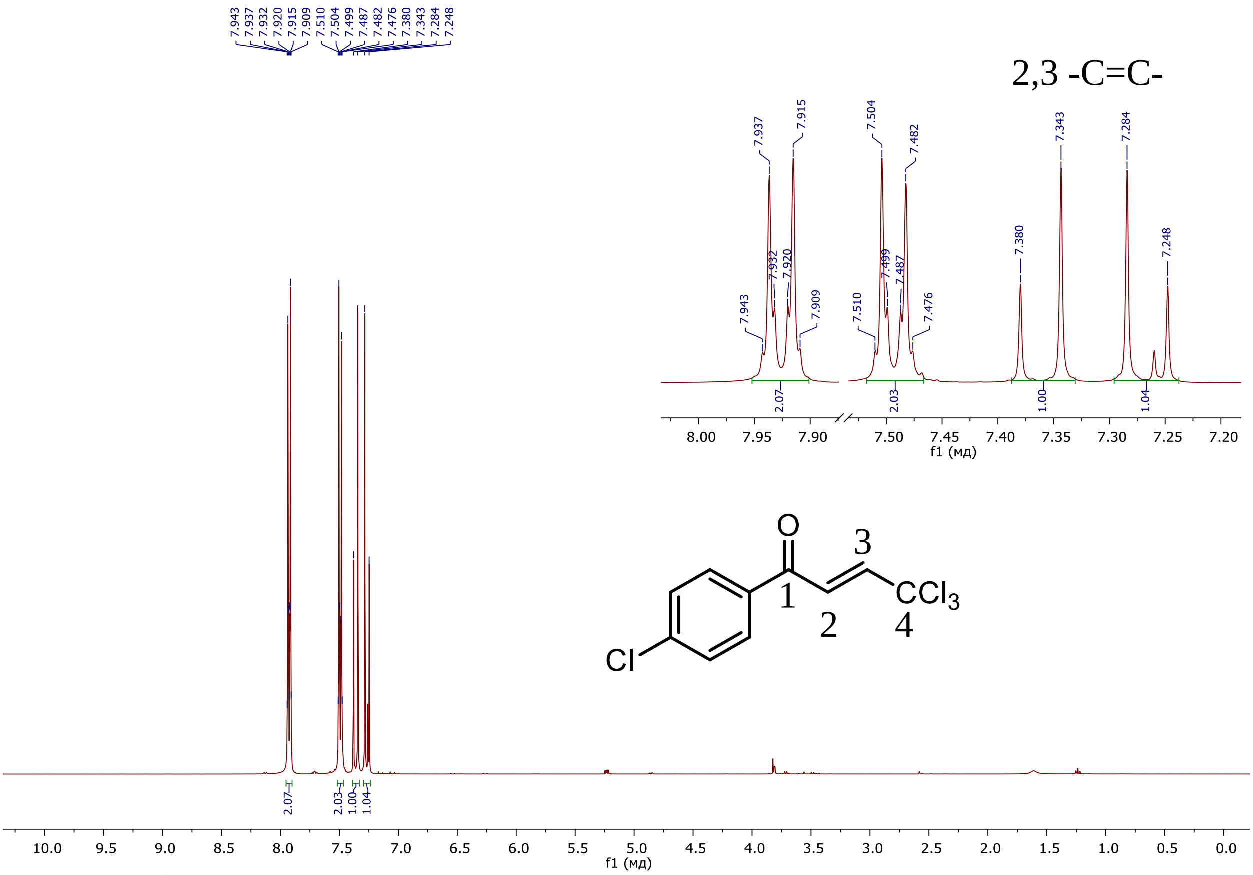

Figure S57. ^{1}H NMR spectrum of the compound **2e** ($CDCl_3$, 400 MHz).

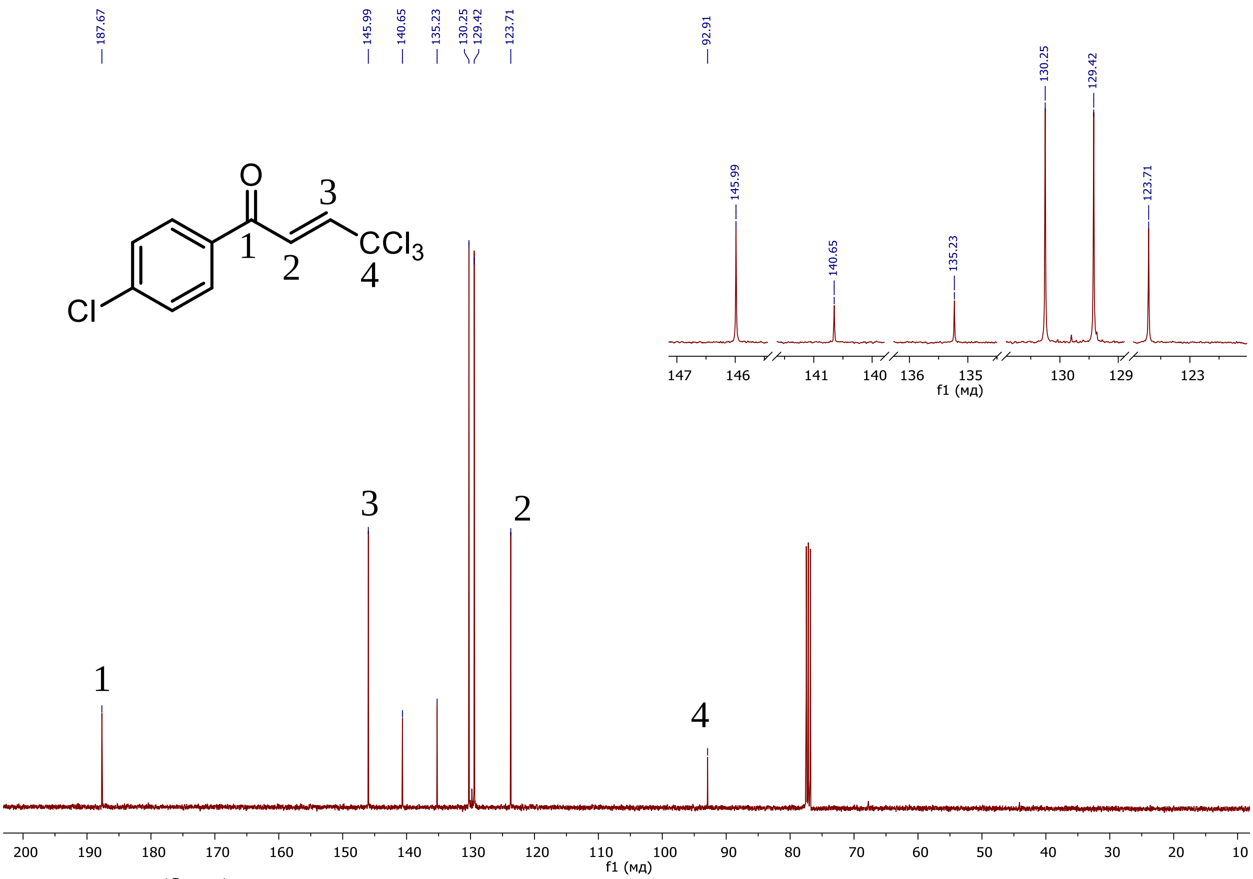

Figure S58. $^{13}C\{^{1}H\}$ NMR spectrum of the compound **2e** ($CDCl_3$, 101 MHz).

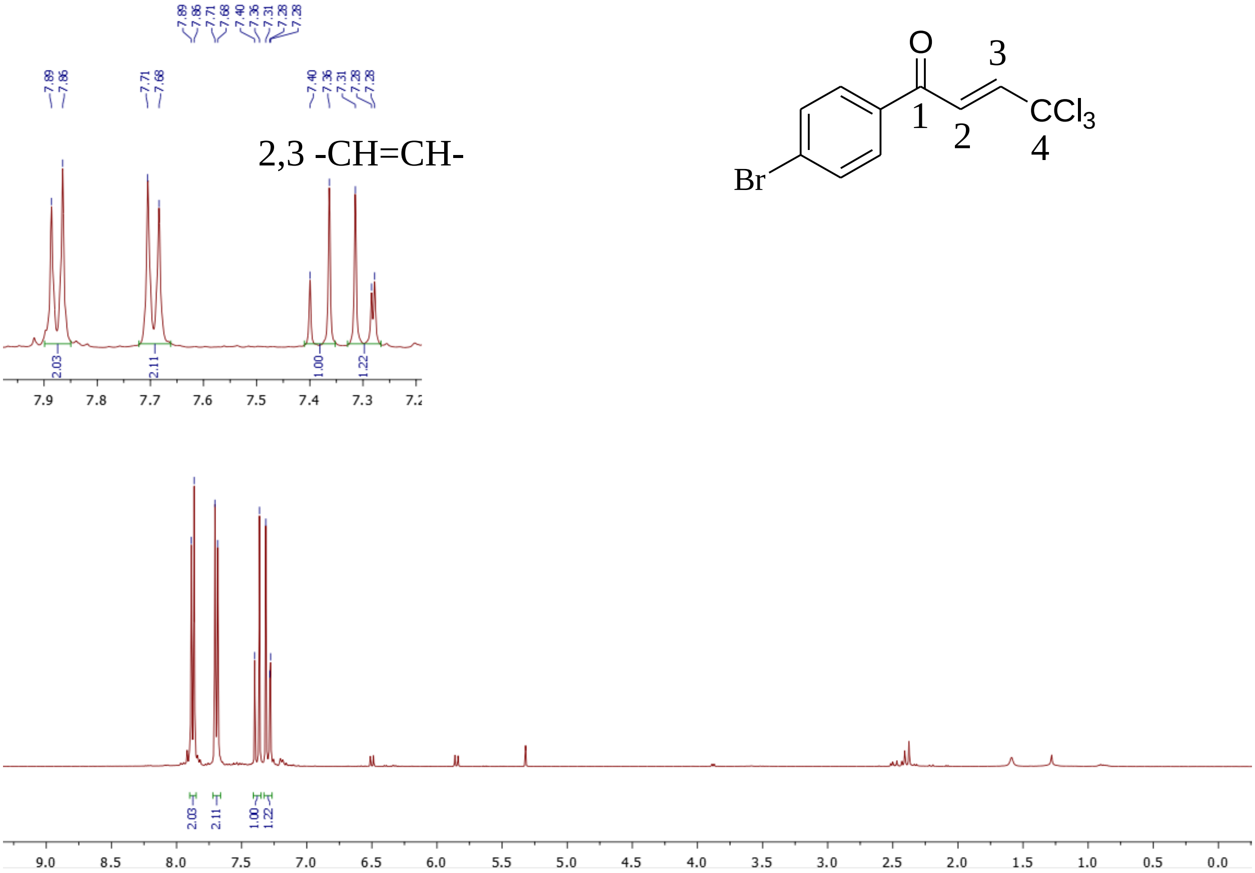

Figure S59. ^{1}H NMR spectrum of the compound **2f** ($CDCl_3$, 400 MHz).

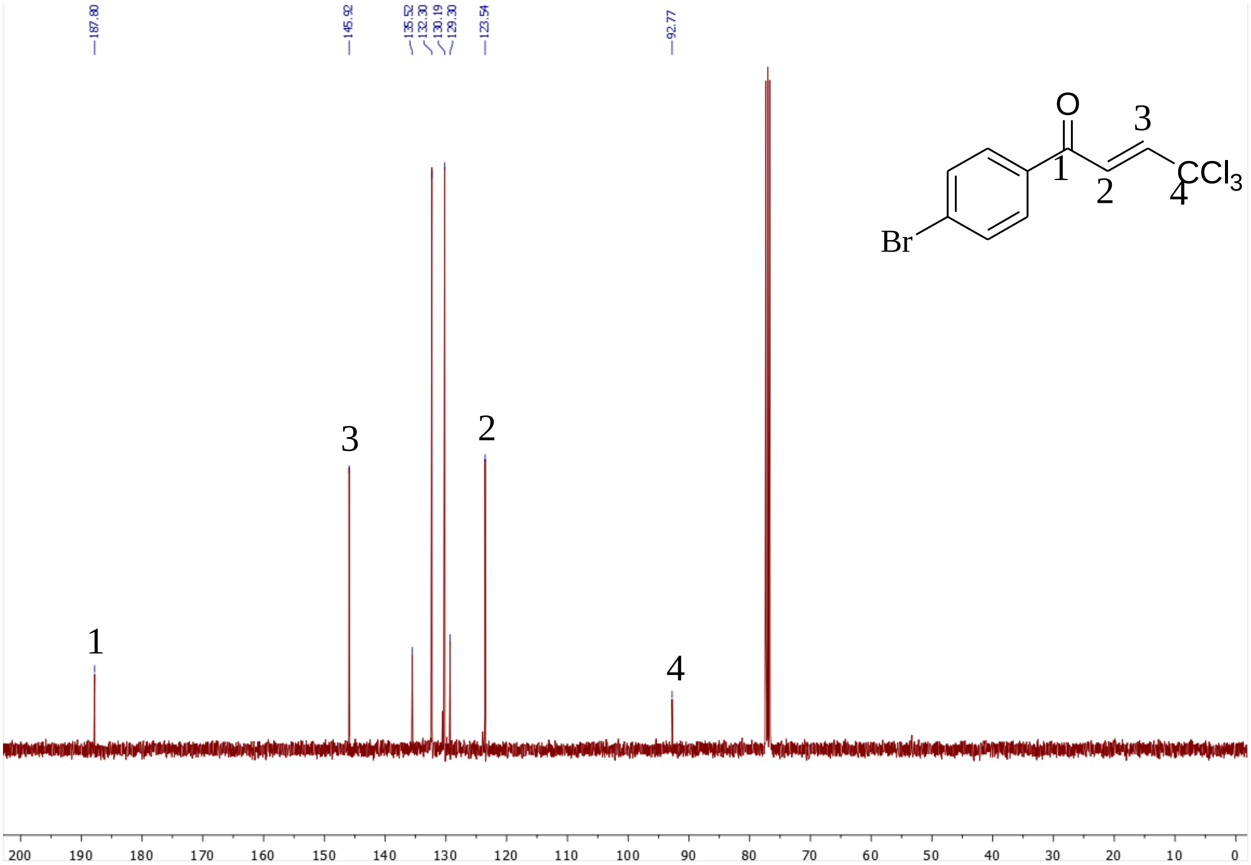

Figure S60. $^{13}C\{^{1}H\}$ NMR spectrum of the compound **2f** ($CDCl_3$, 101 MHz).

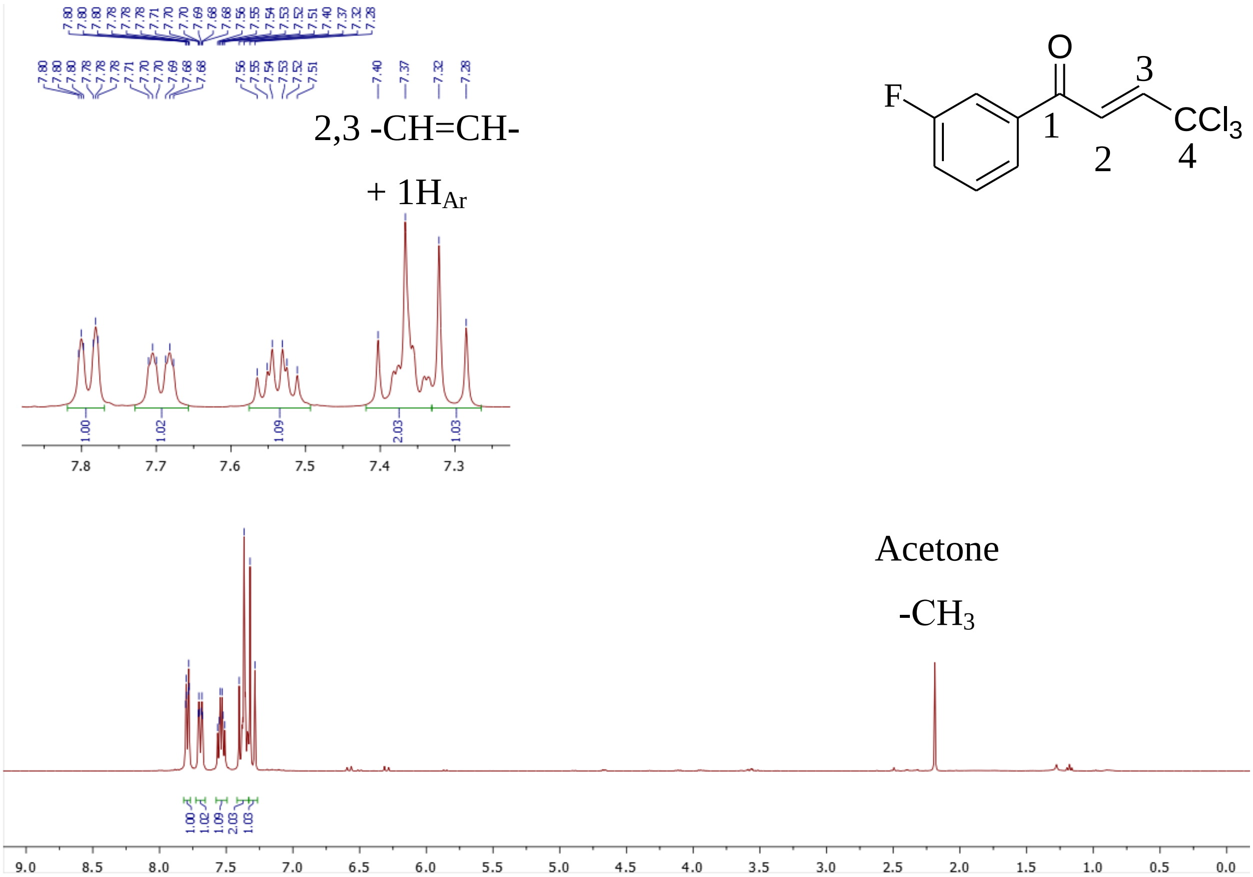

Figure S61. ^{1}H NMR spectrum of the compound **2g** ($CDCl_3$, 400 MHz).

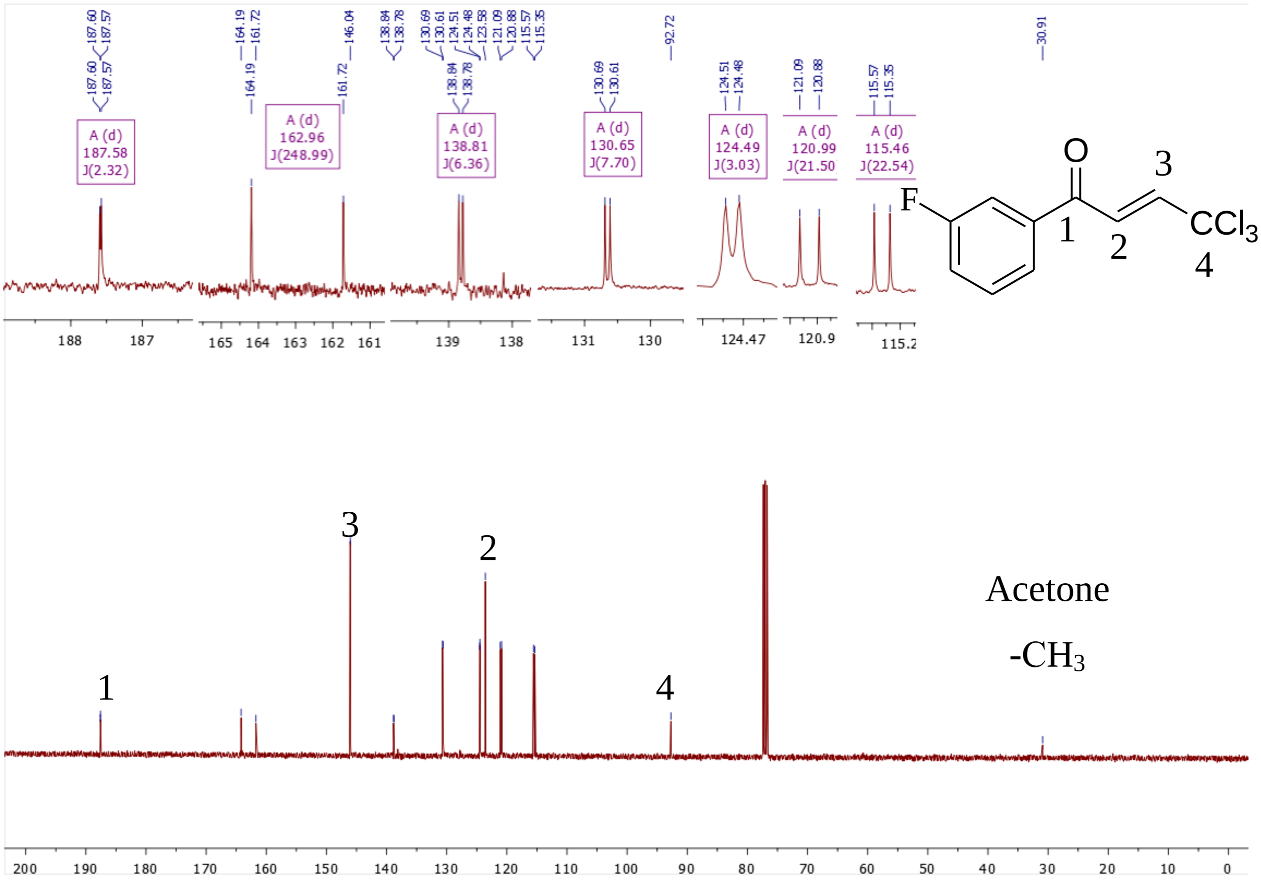

Figure S62. ^{13}C{^{1}H} NMR spectrum of the compound **2g** ($CDCl_3$, 101 MHz).

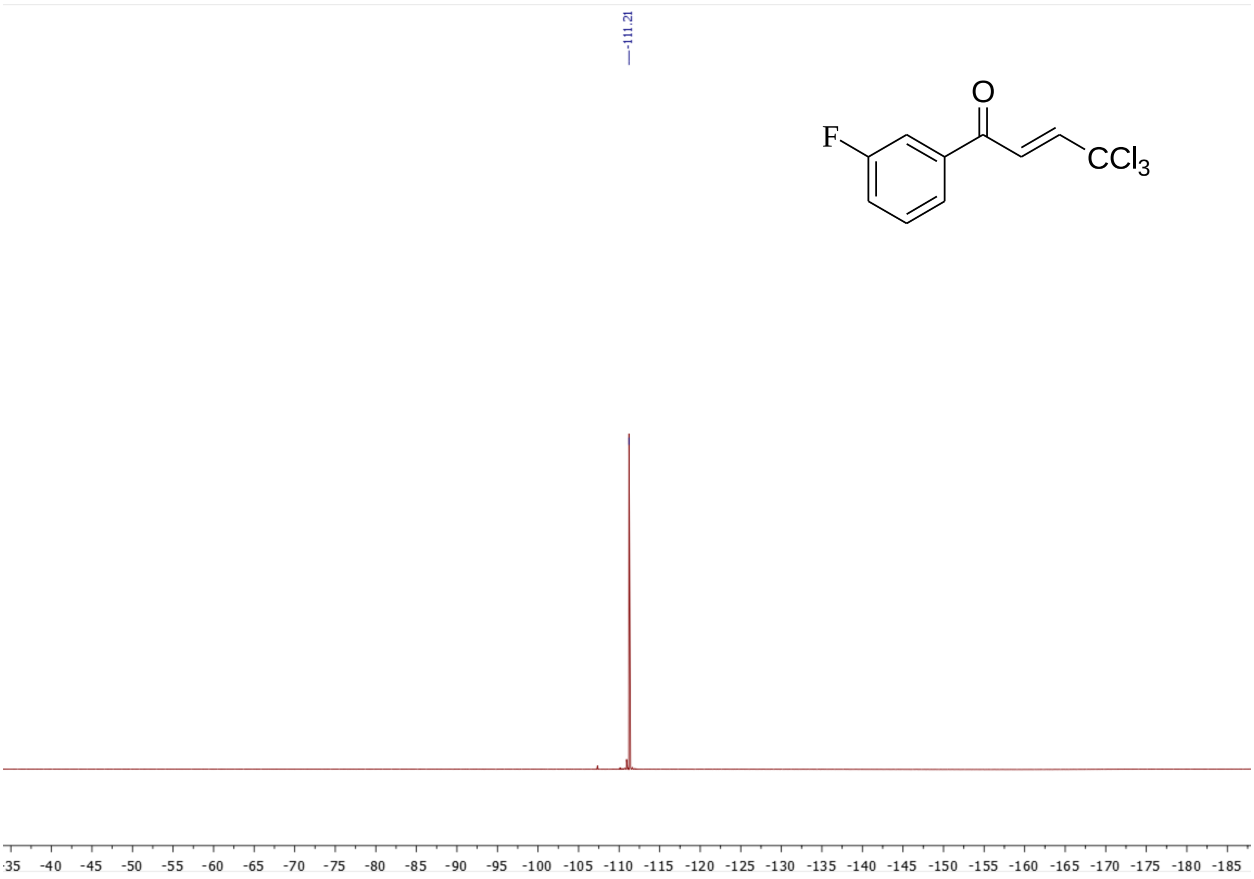

Figure S63. $^{19}F\{^{1}H\}$ NMR spectrum of the compound **2g** ($CDCl_3$, 376 MHz).

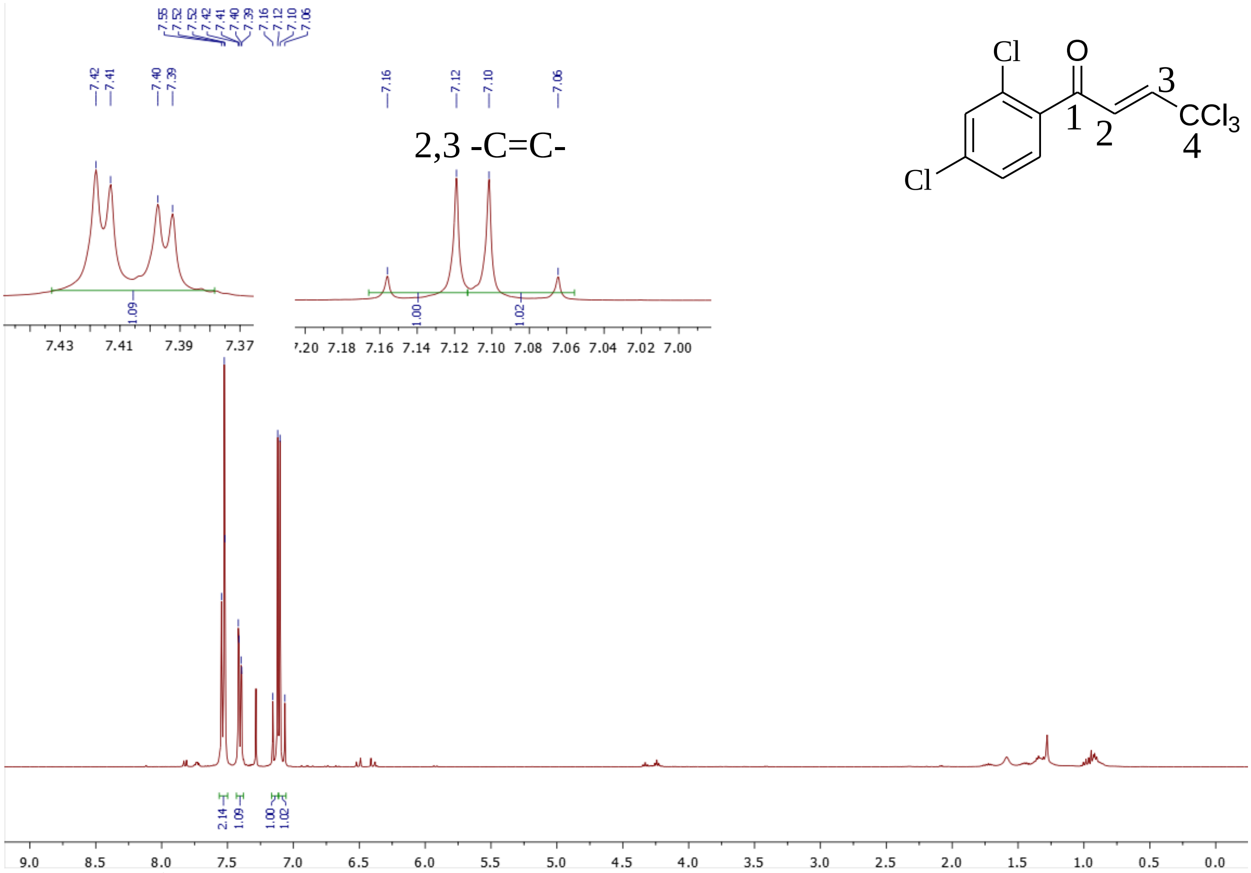

Figure S64. ^{1}H NMR spectrum of the compound **2h** ($CDCl_3$, 400 MHz).

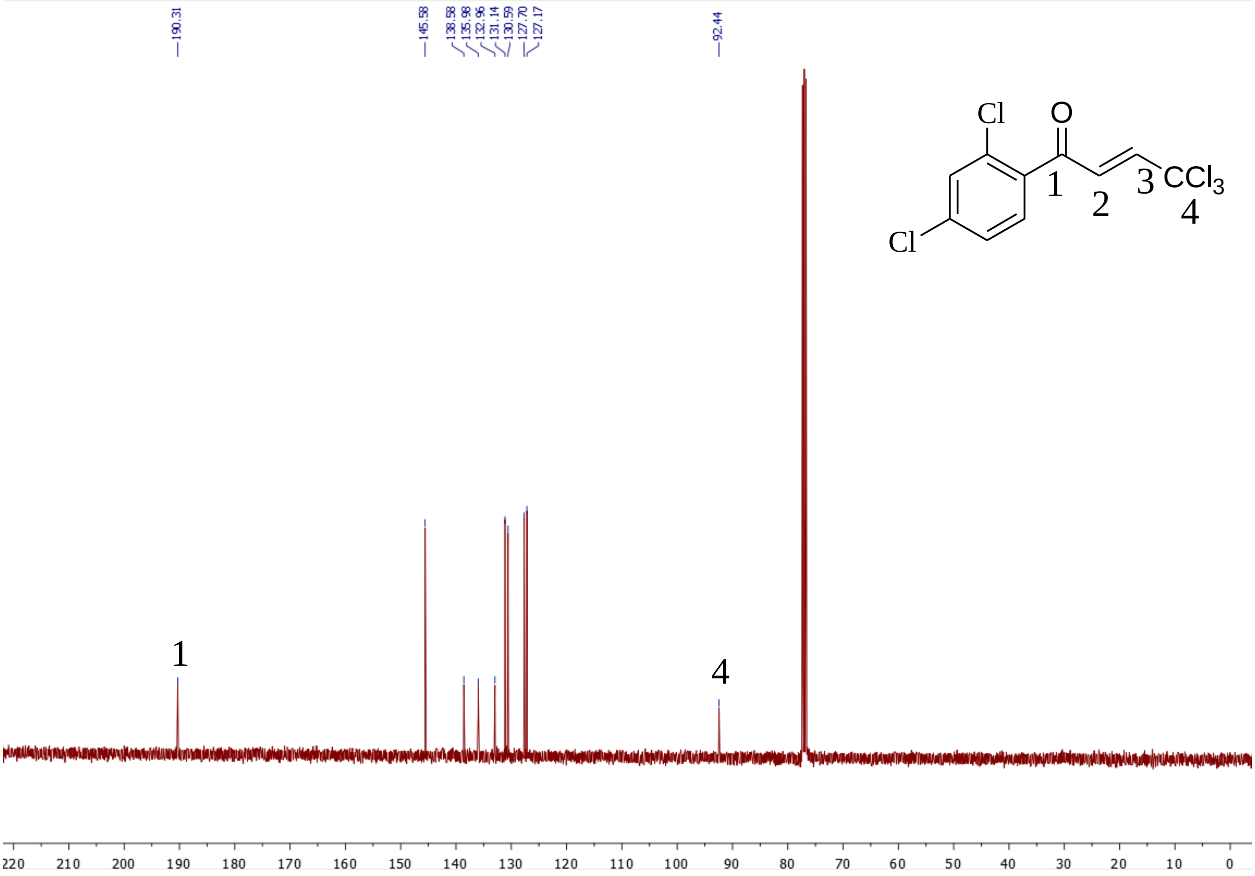

Figure S65. $^{13}C\{^{1}H\}$ NMR spectrum of the compound **2h** ($CDCl_3$, 101 MHz).

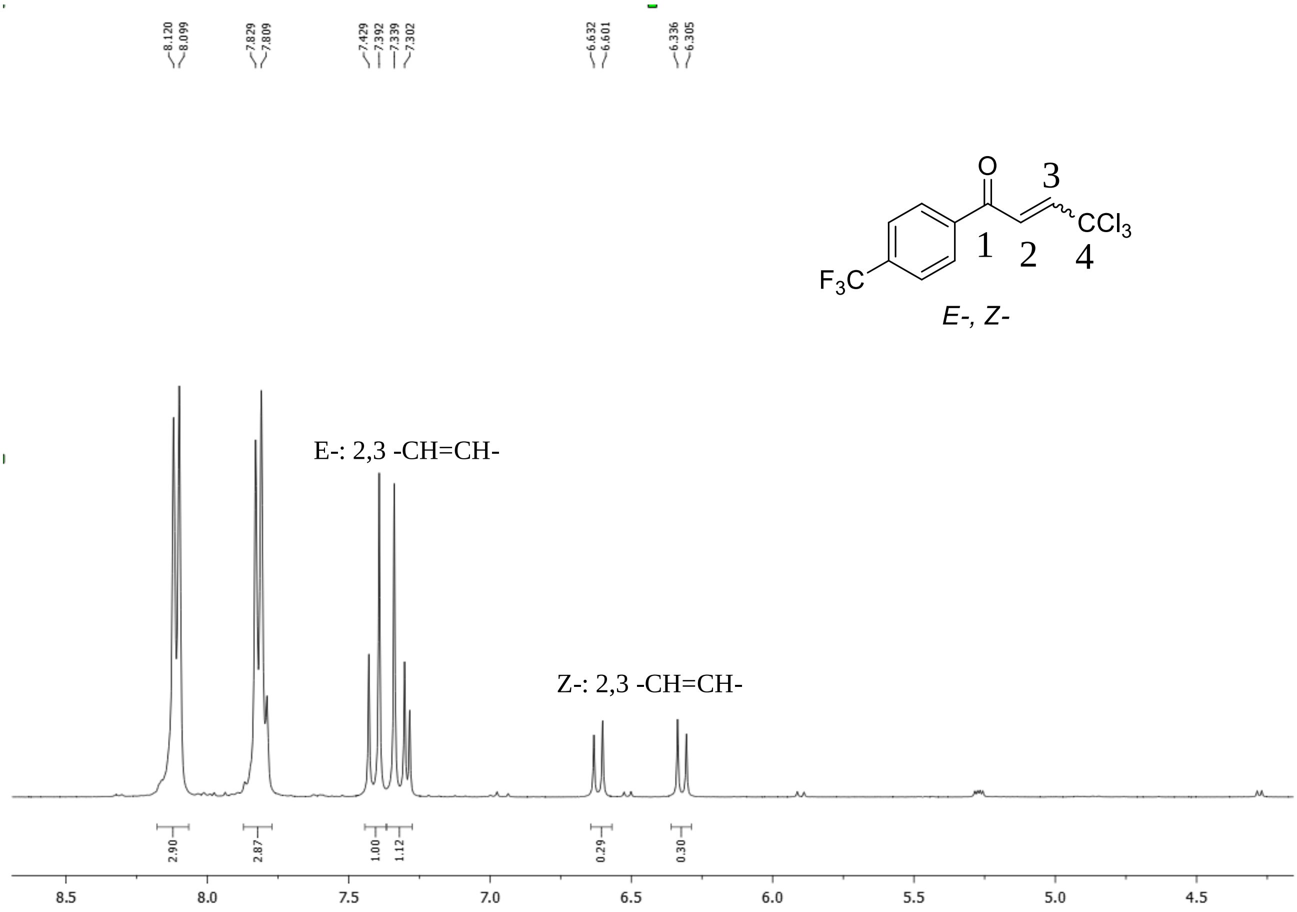

Figure S66. ^{1}H NMR spectrum of the compound **2i** ($CDCl_3$, 400 MHz).

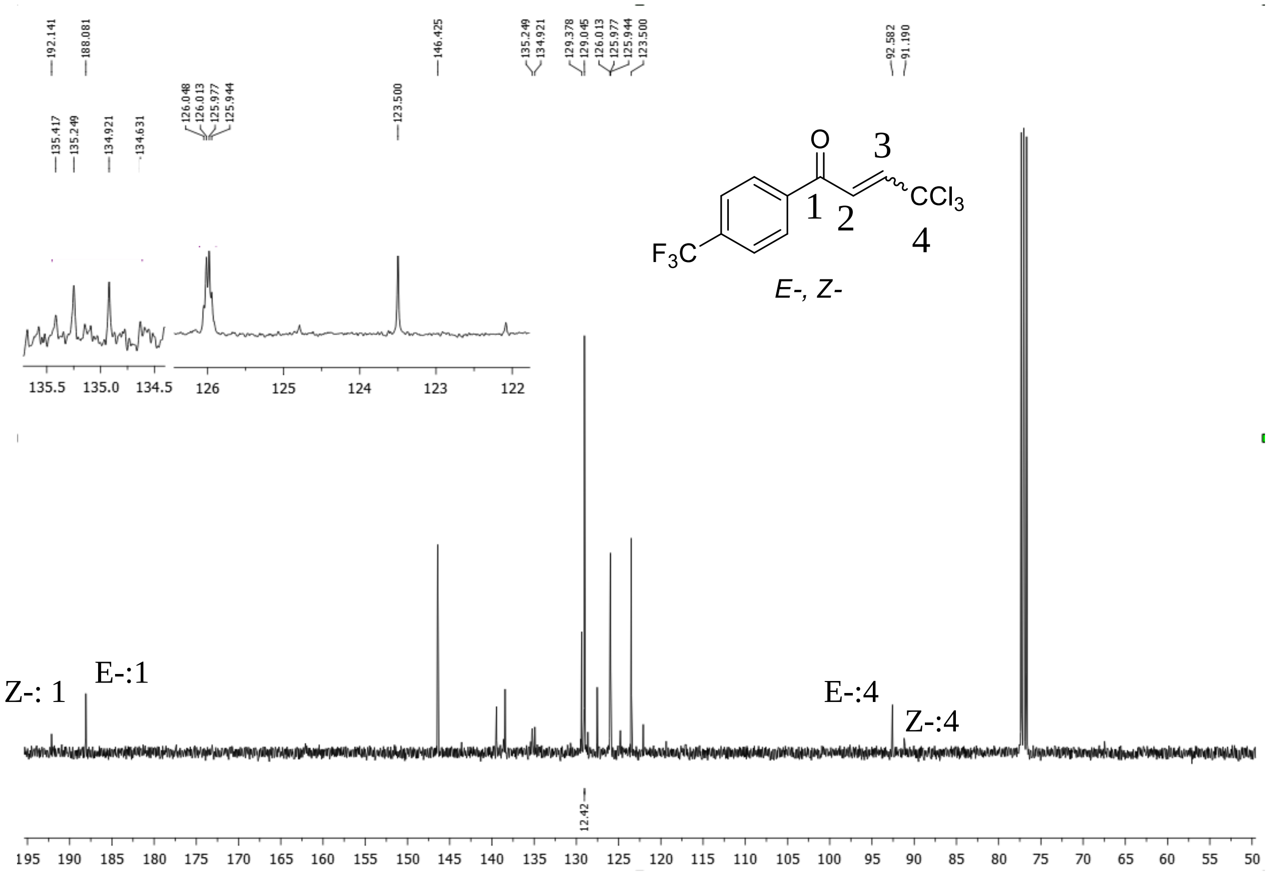

Figure S67. $^{13}C\{^{1}H\}$ NMR spectrum of the compound **2i** ($CDCl_3$, 101 MHz).

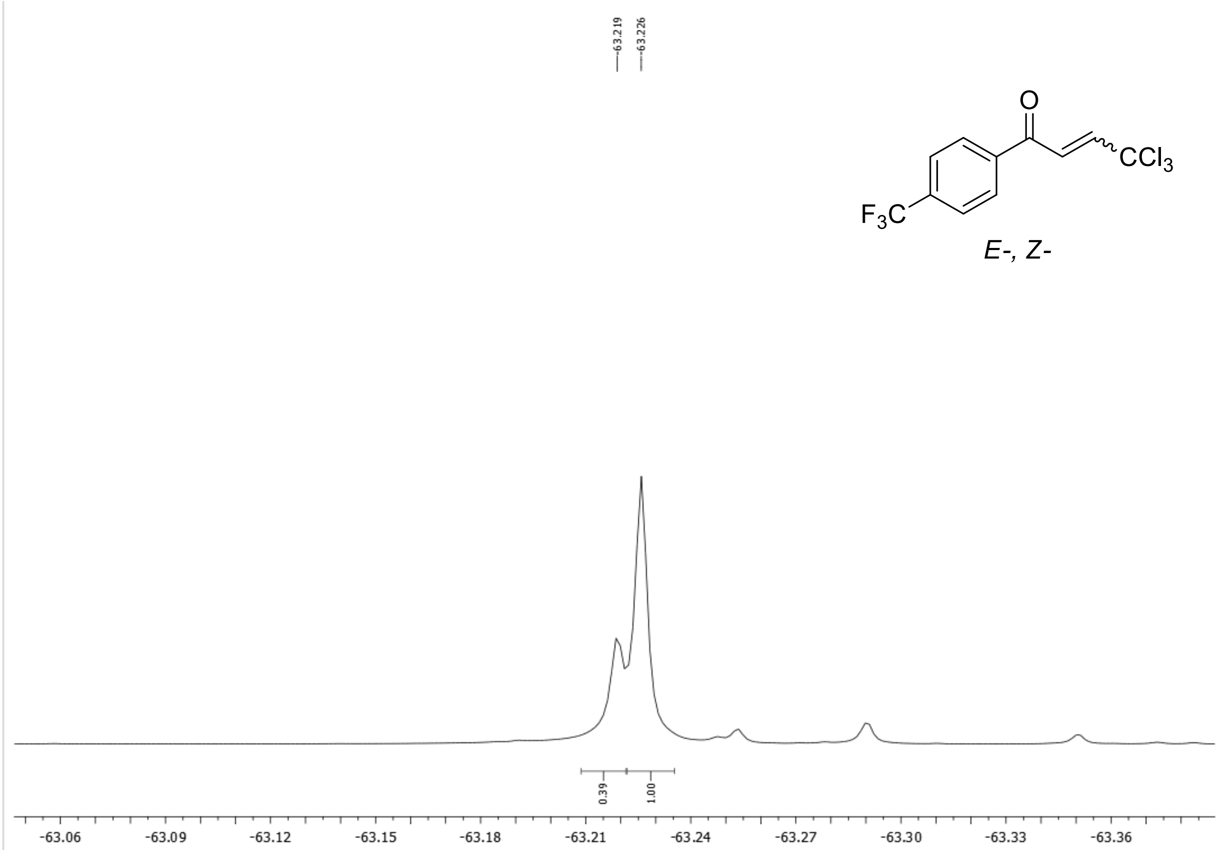

Figure S68. $^{19}F\{^{1}H\}$ NMR spectrum of the compound **2i** ($CDCl_3$, 376 MHz).

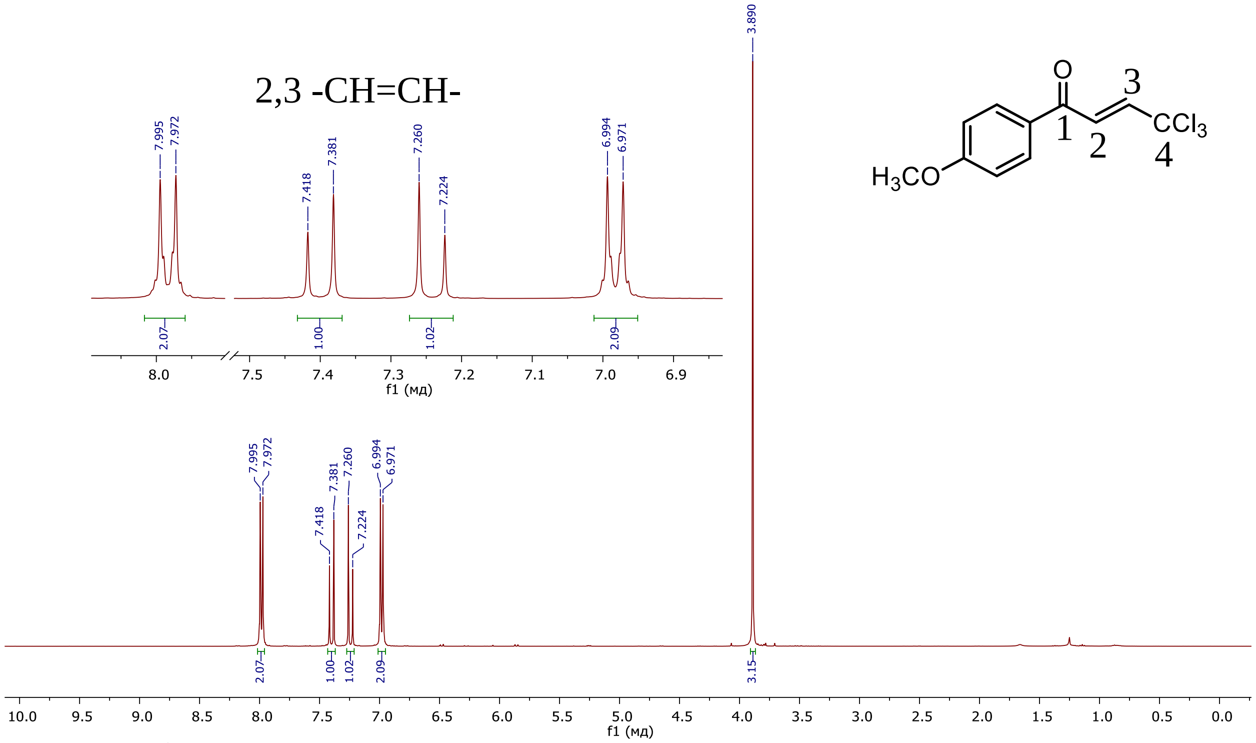

Figure S69. ^{1}H NMR spectrum of the compound **2j** ($CDCl_3$, 400 MHz).

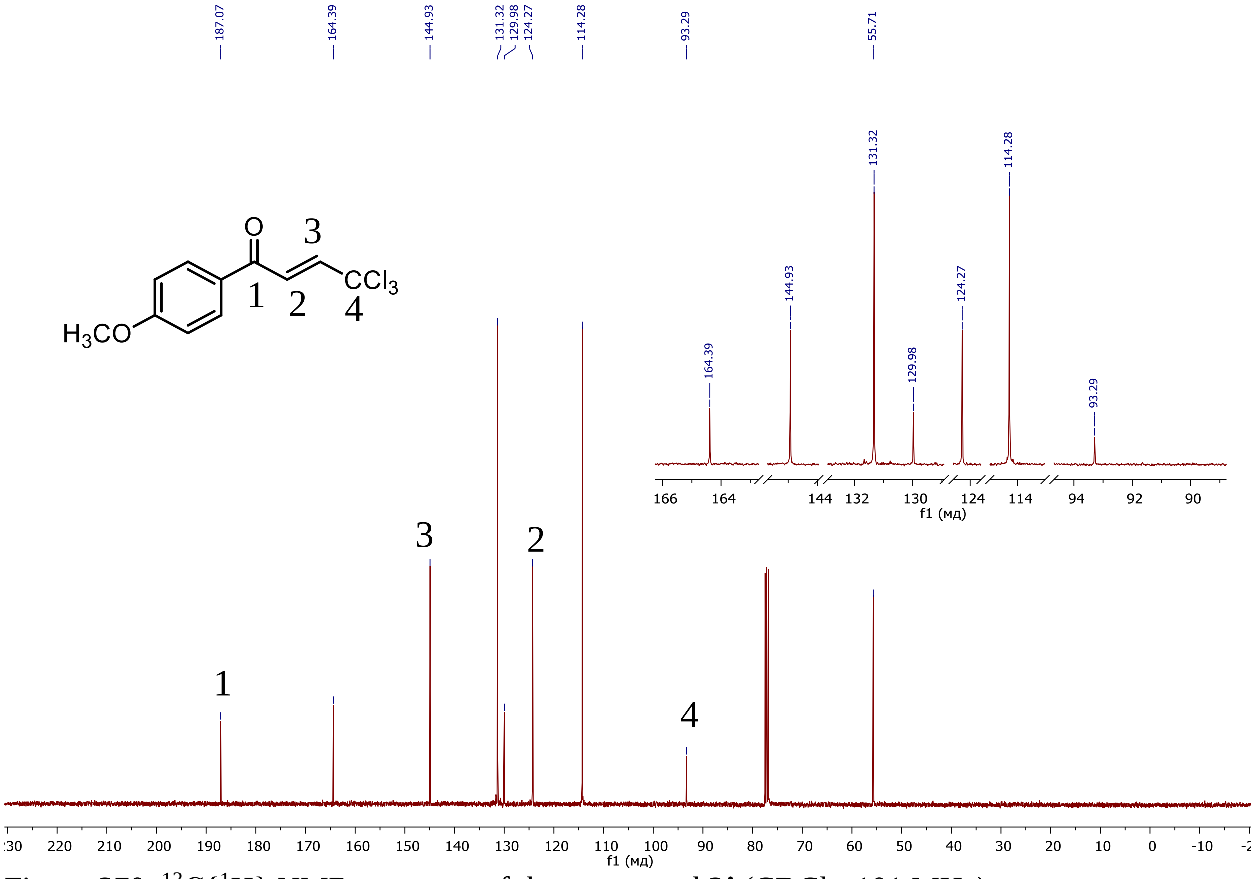

Figure S70. ^{13}C{^{1}H} NMR spectrum of the compound **2j** ($CDCl_3$, 101 MHz).

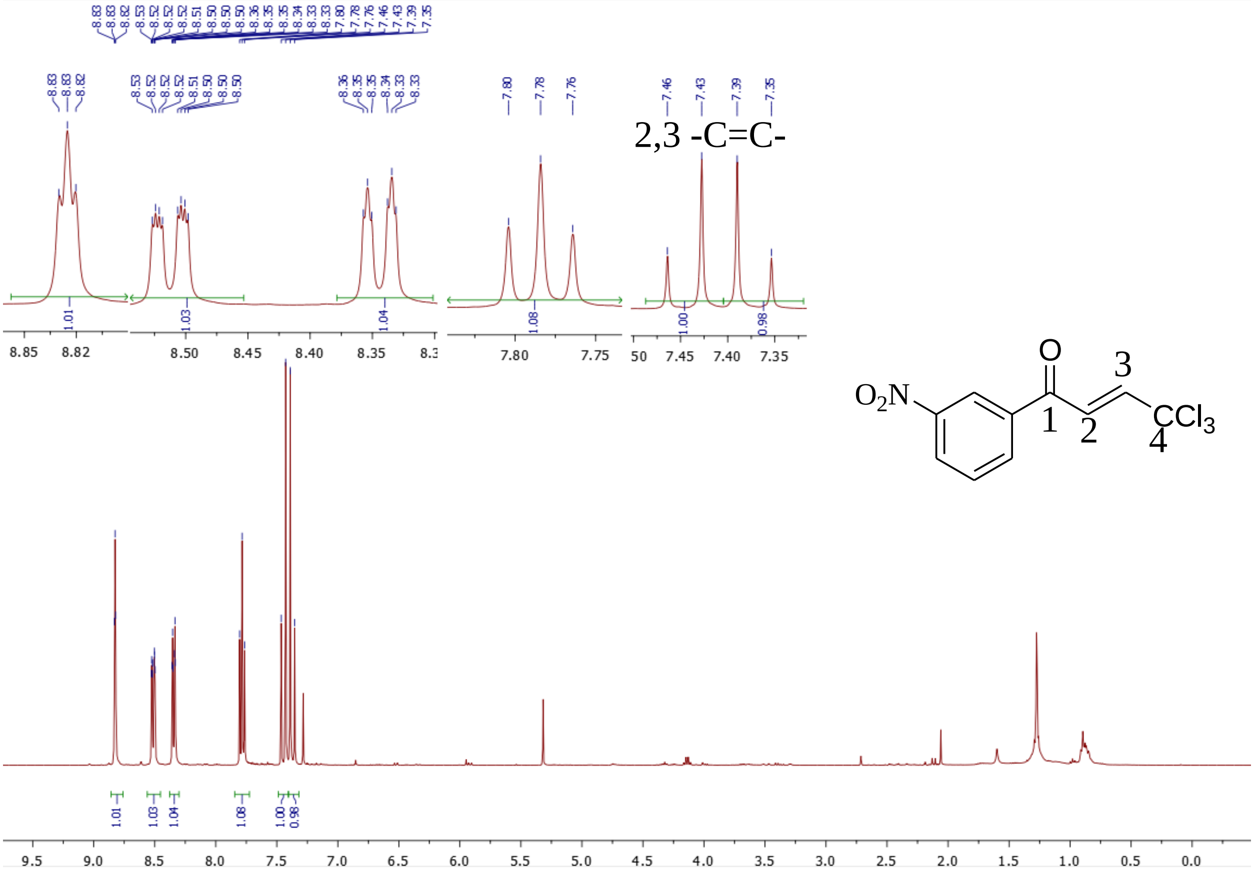

Figure S71. ^{1}H NMR spectrum of the compound **2l** ($CDCl_3$, 400 MHz).

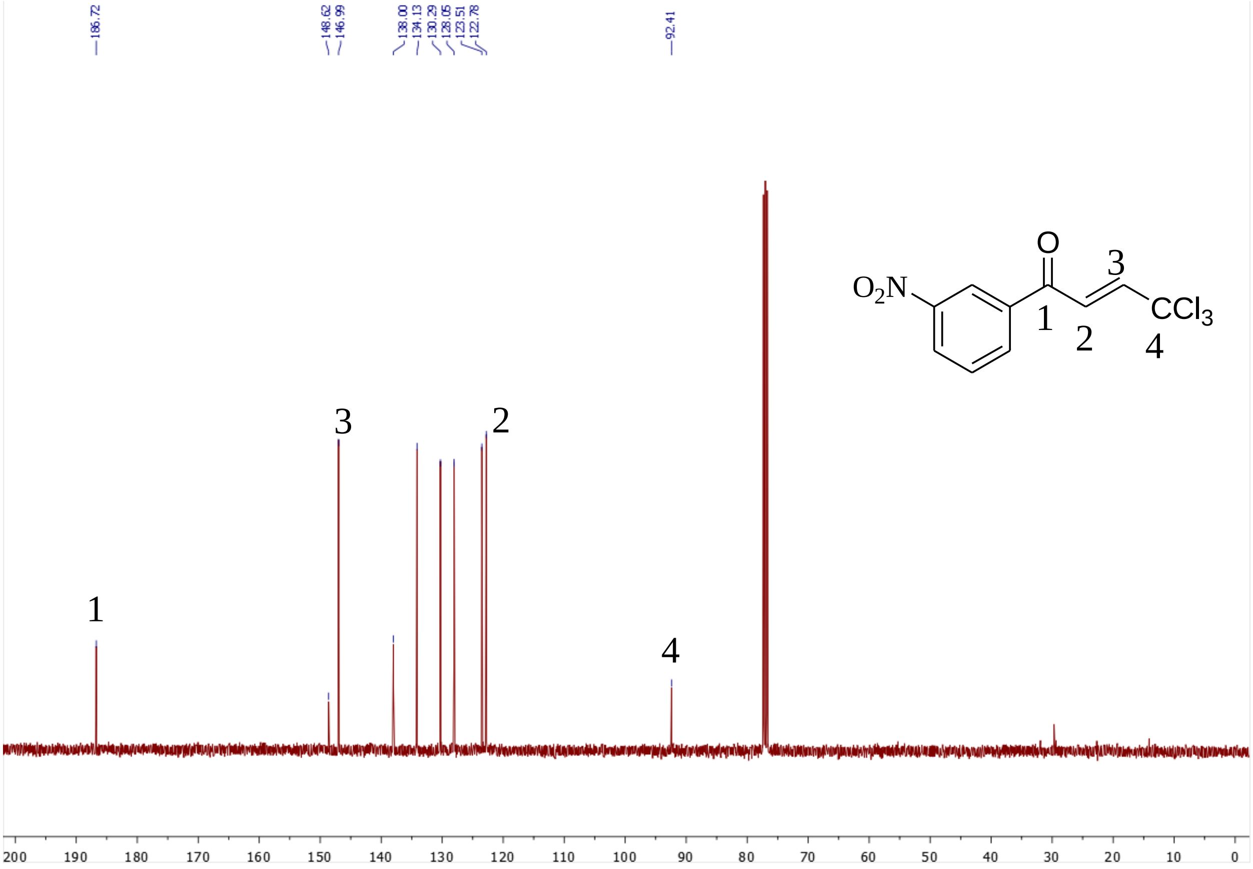

Figure S72. $^{13}C\{^1H\}$ NMR spectrum of the compound **2l** ($CDCl_3$, 101 MHz).

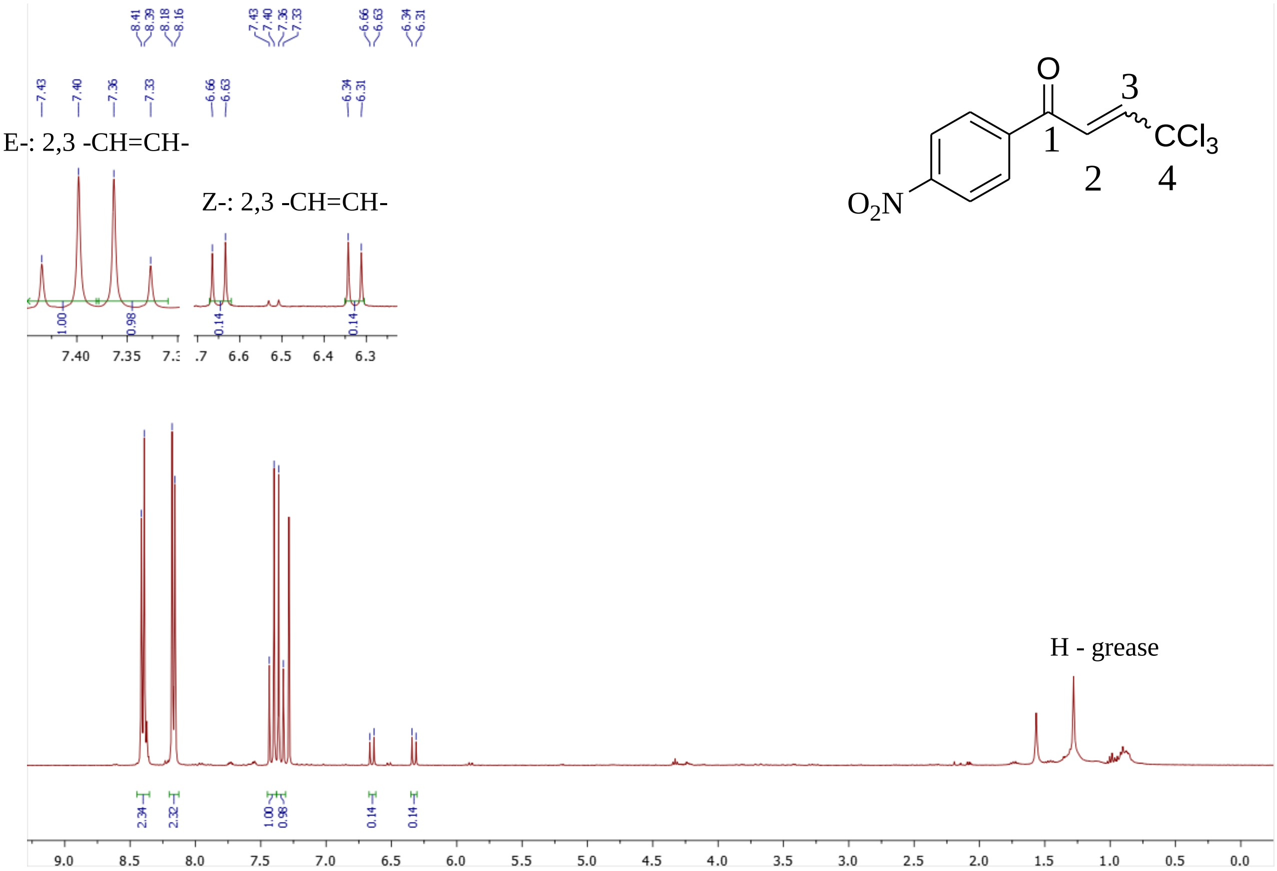

Figure S73. ^{1}H NMR spectrum of the compound **2m** ($CDCl_3$, 400 MHz).

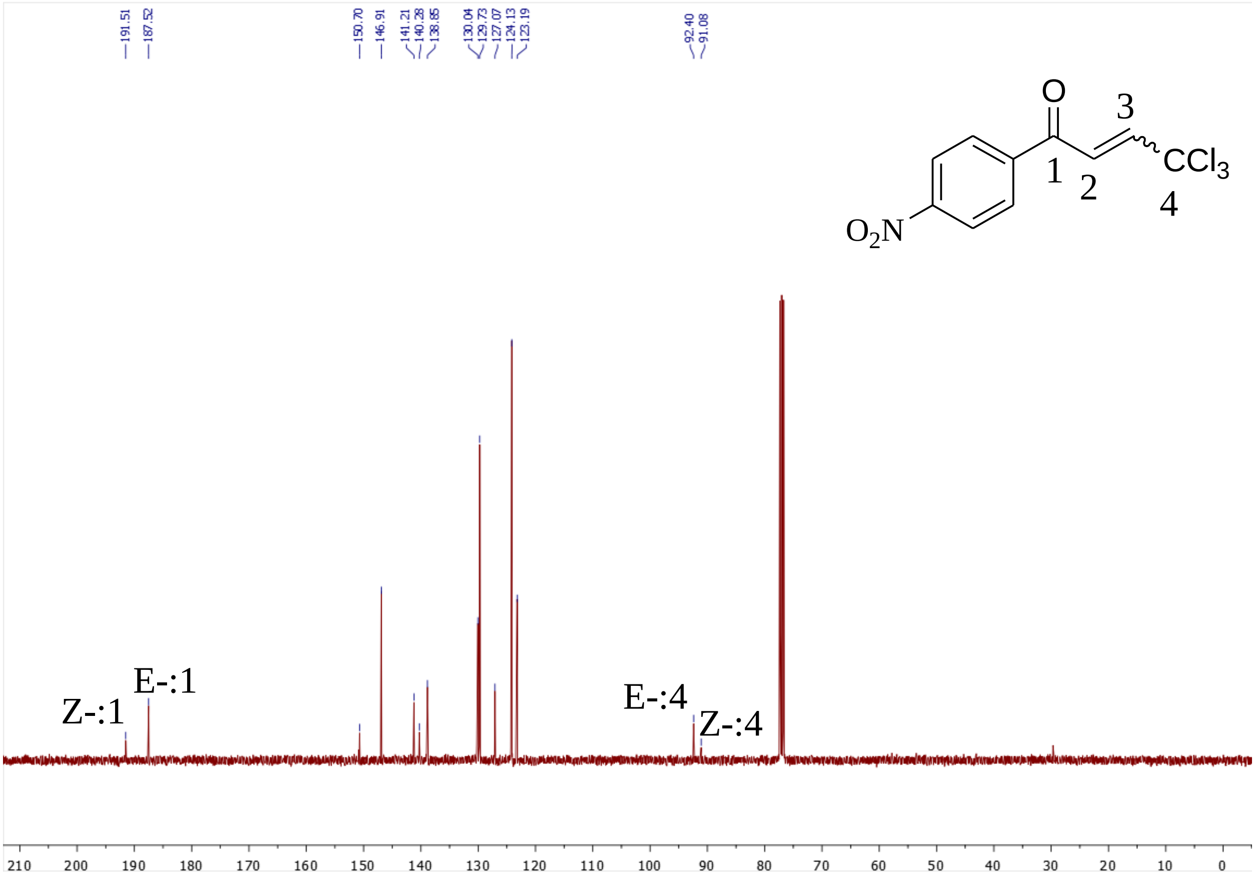

Figure S74. $^{13}C\{^{1}H\}$ NMR spectrum of the compound **2m** ($CDCl_3$, 101 MHz).

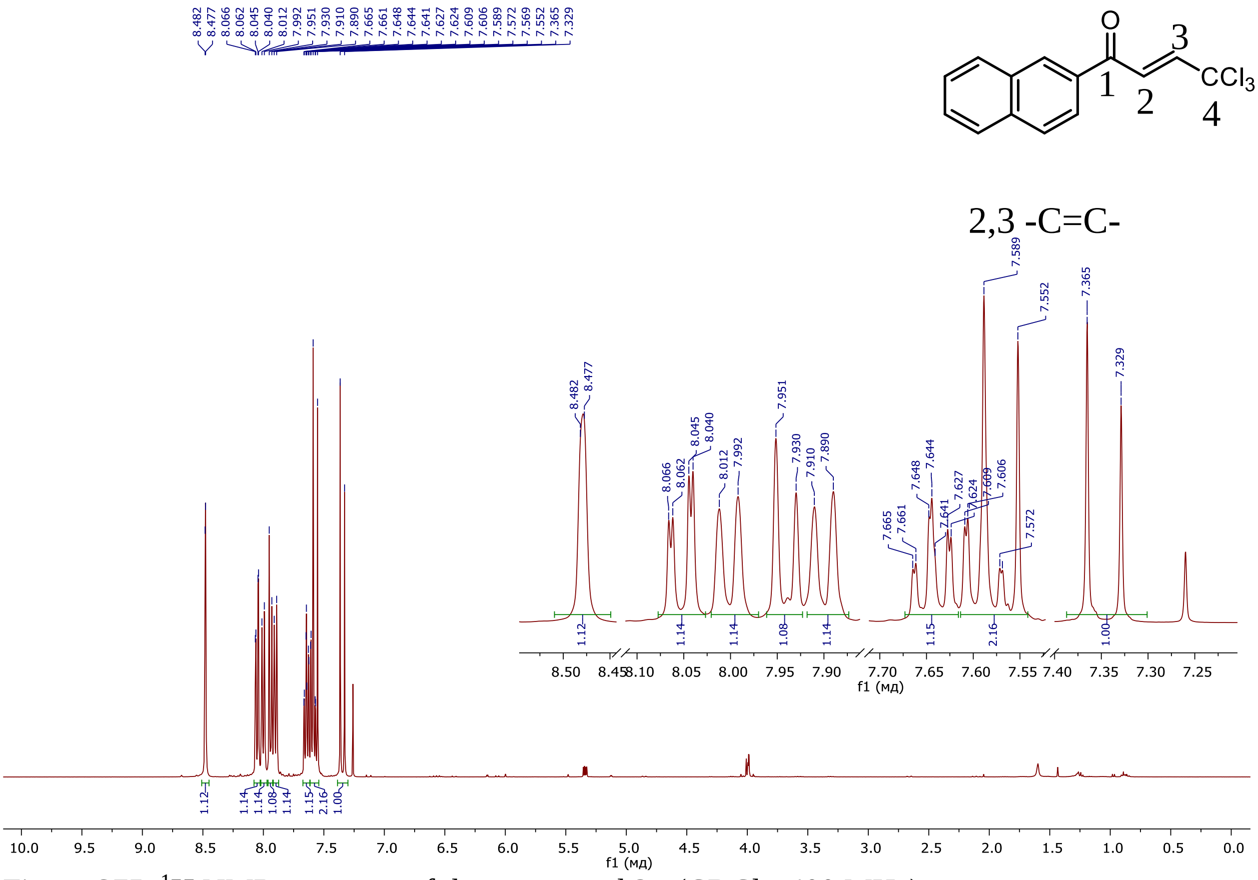

Figure S75. ^{1}H NMR spectrum of the compound **2n** ($CDCl_3$, 400 MHz).

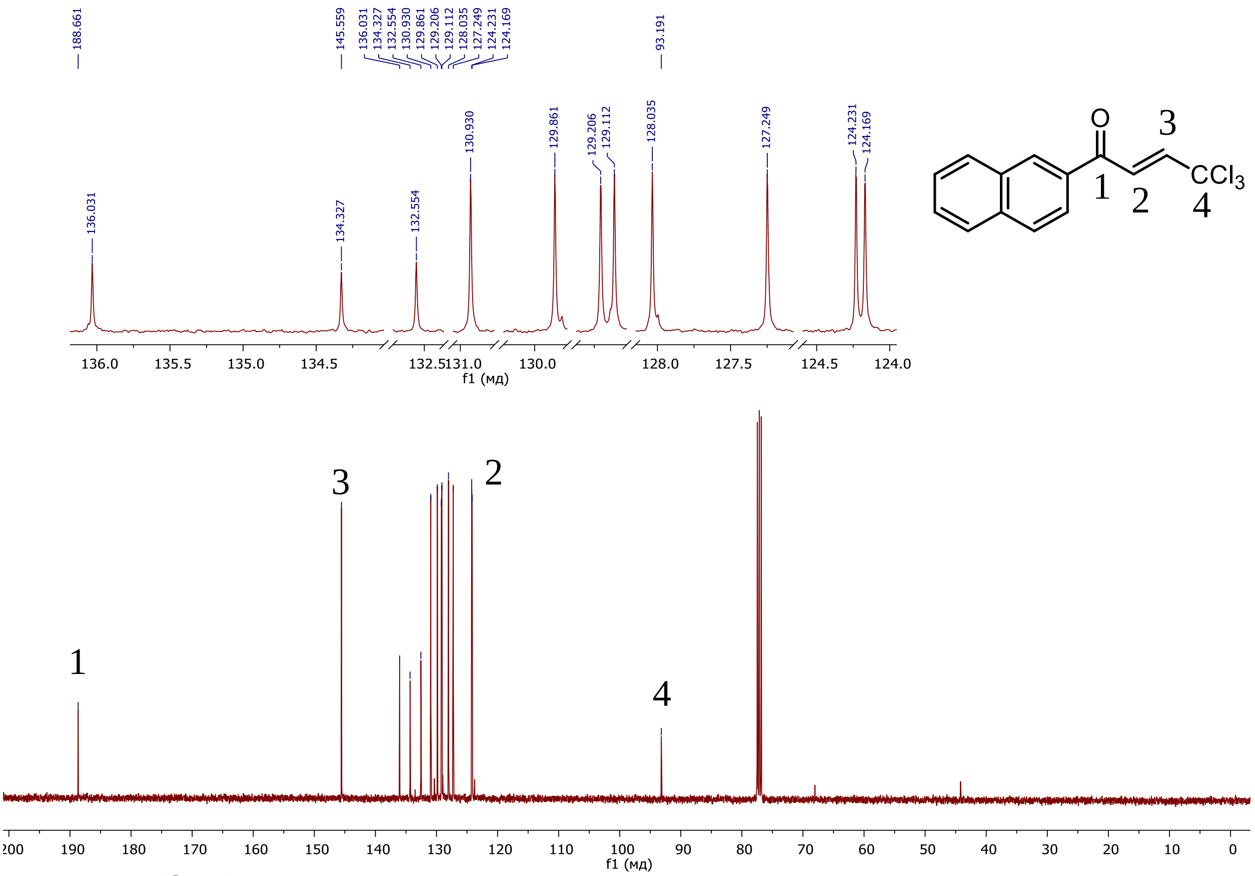

Figure S76. $^{13}C\{^{1}H\}$ NMR spectrum of the compound **2n** ($CDCl_3$, 101 MHz).

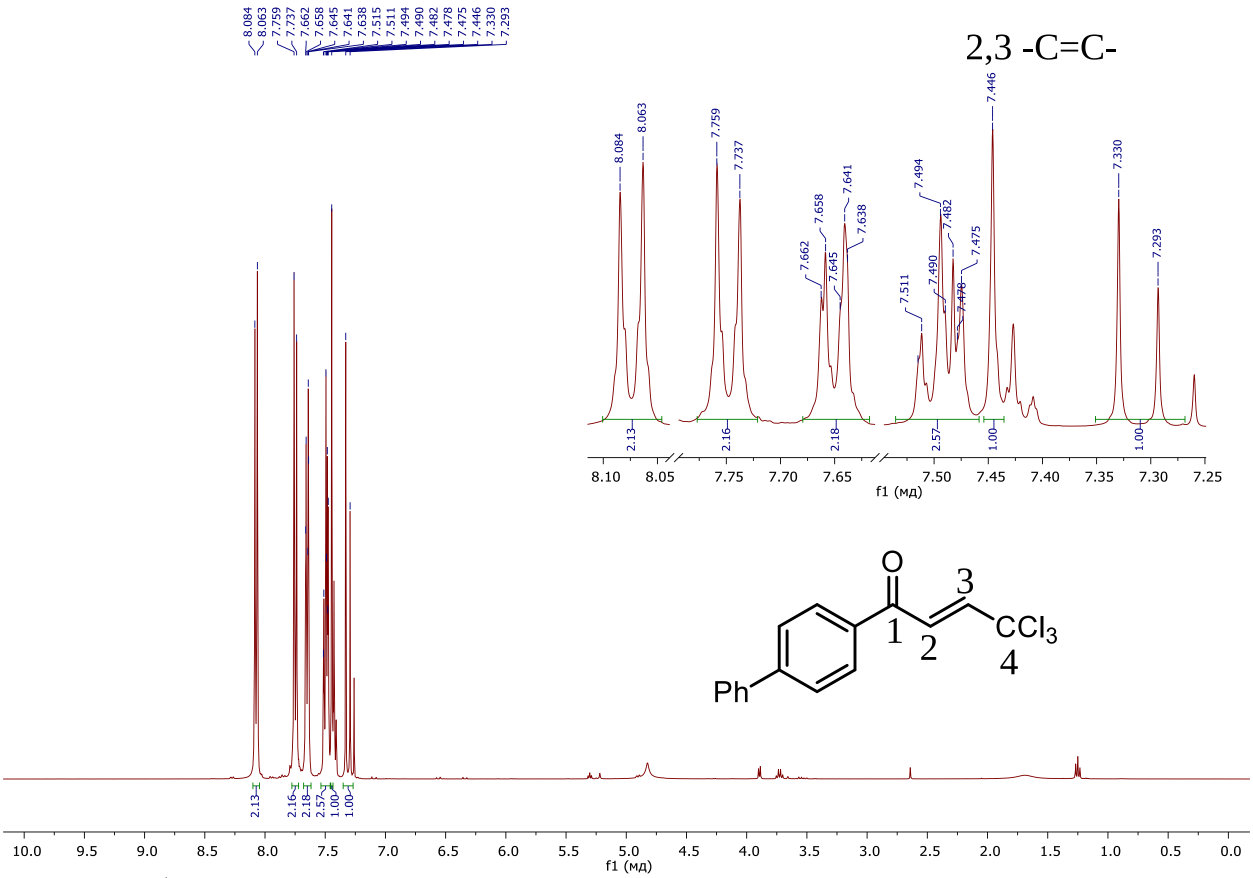

Figure S77. ^{1}H NMR spectrum of the compound **2o** ($CDCl_3$, 400 MHz).

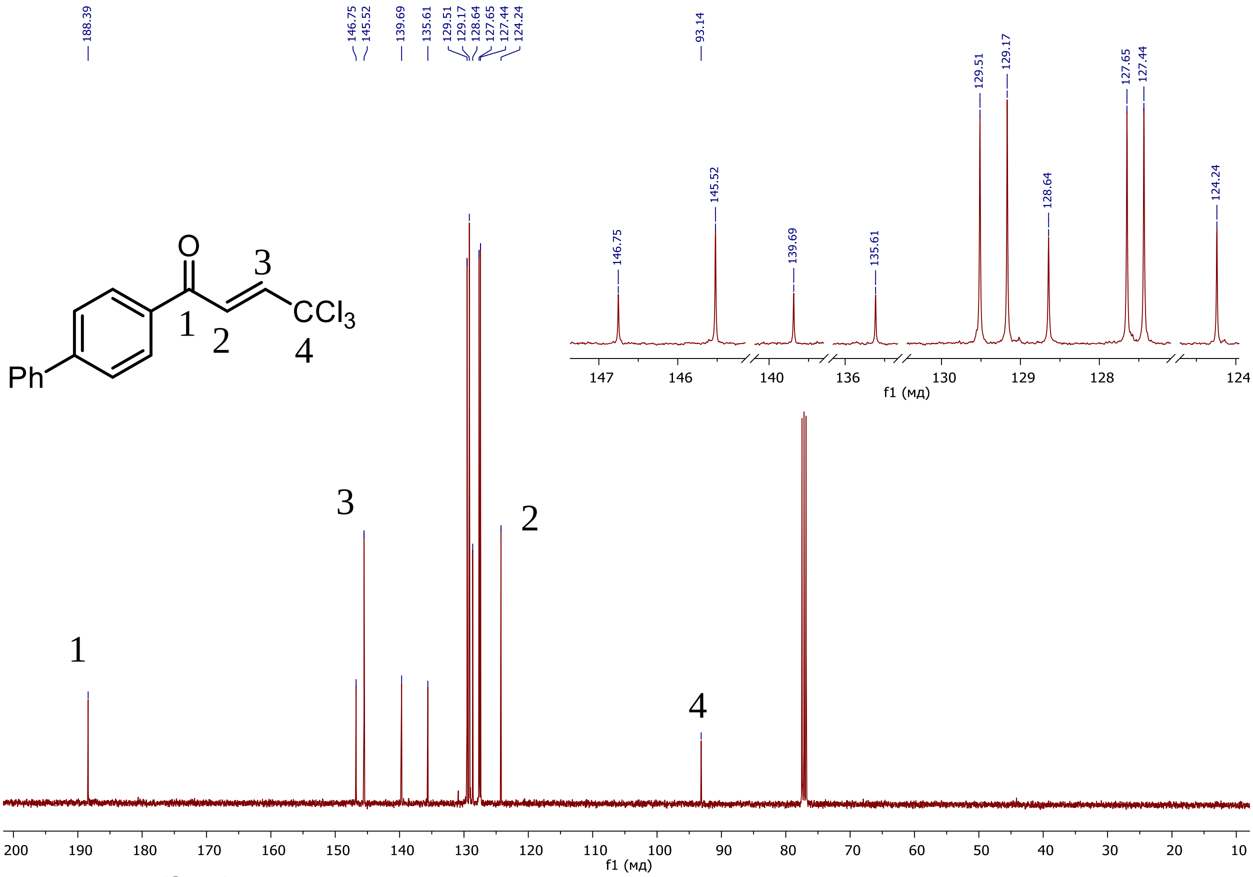

Figure S78. ^{13}C{^{1}H} NMR spectrum of the compound **2o** ($CDCl_3$, 101 MHz).

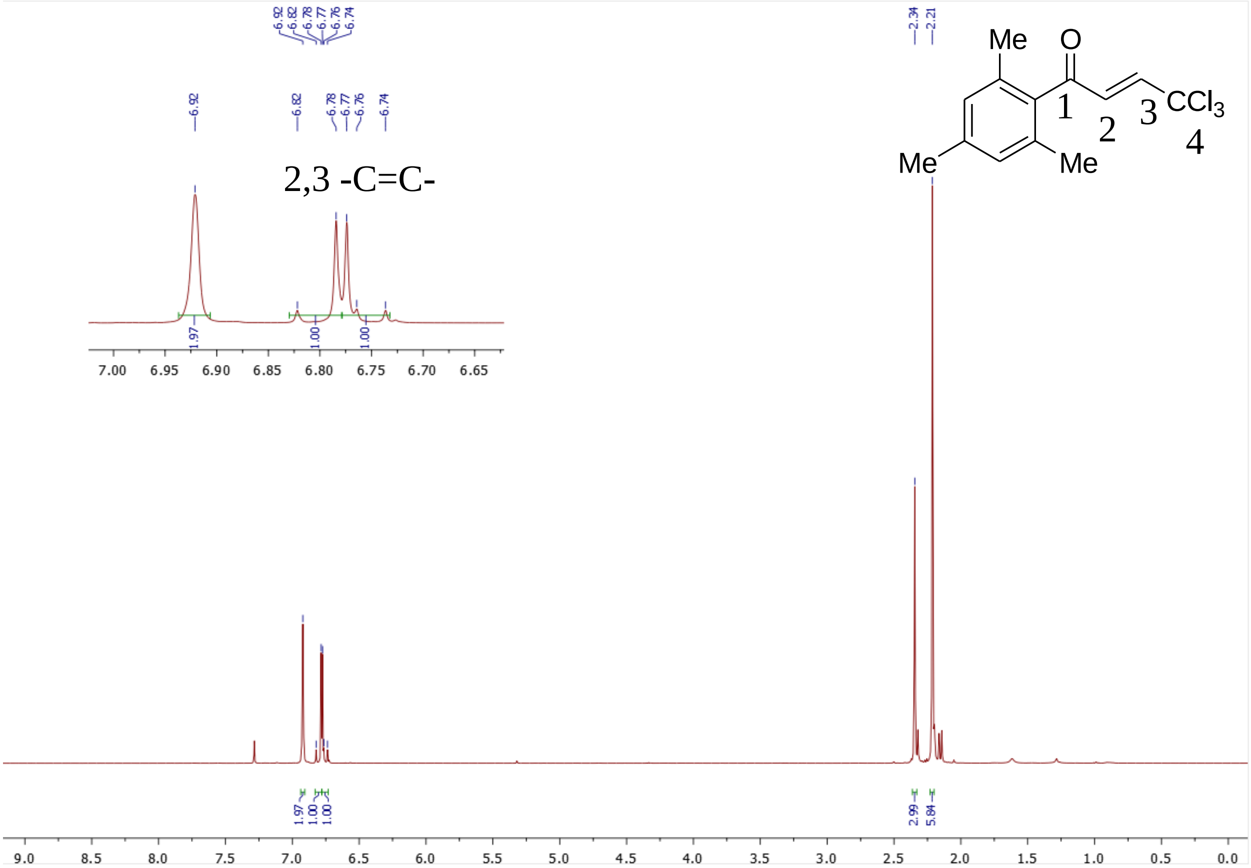

Figure S79. ^{1}H NMR spectrum of the compound **2t** ($CDCl_3$, 400 MHz).

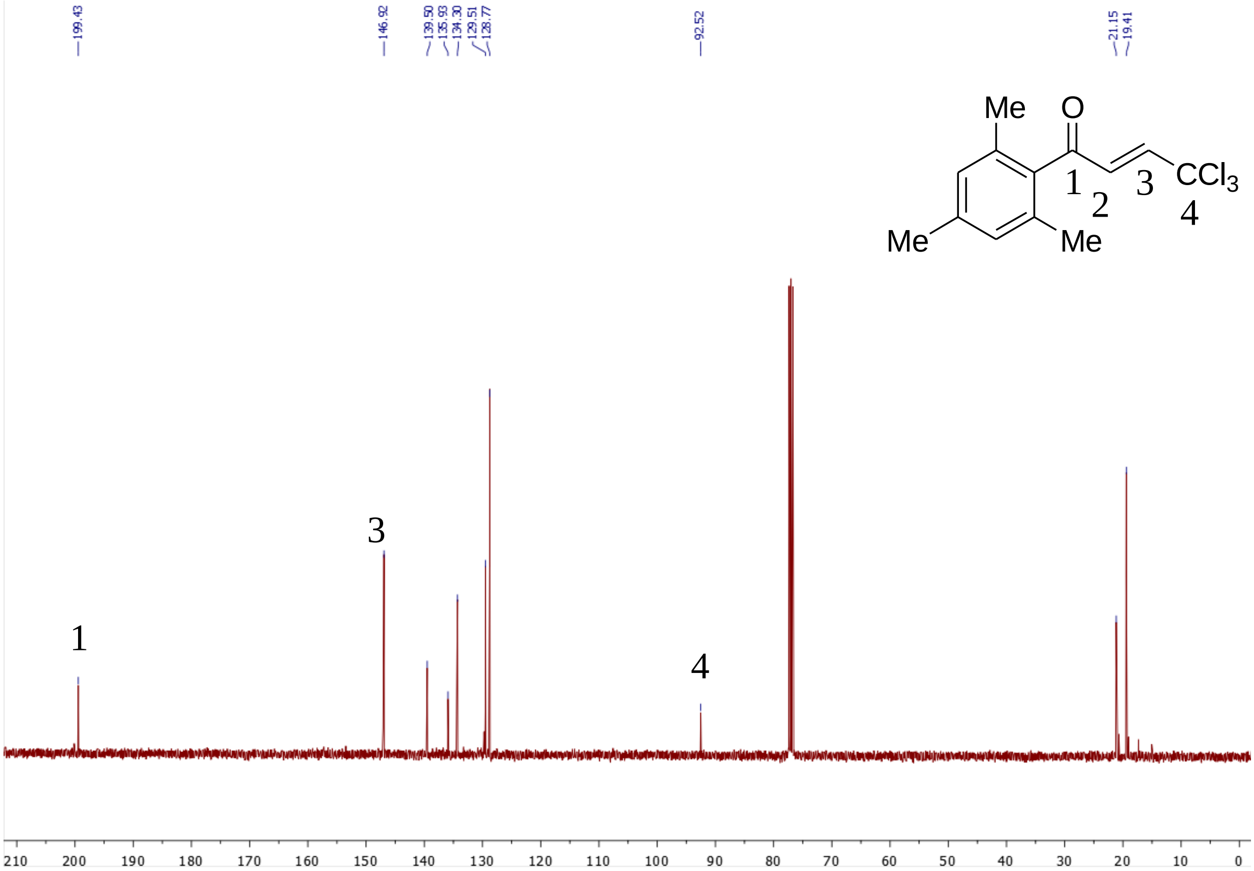

Figure S80. $^{13}C\{^1H\}$ NMR spectrum of the compound **2t** ($CDCl_3$, 101 MHz).

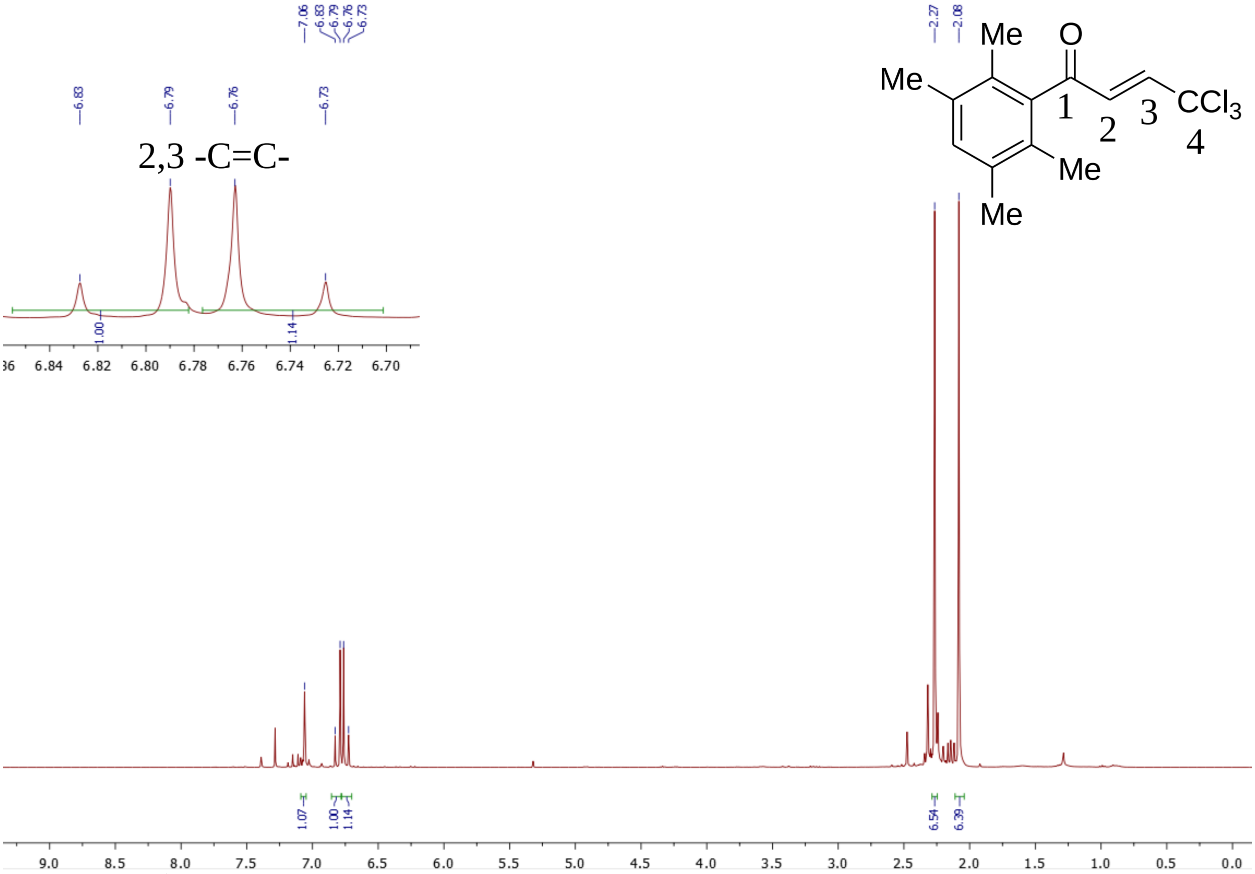

Figure S81. ^{1}H NMR spectrum of the compound **2u** ($CDCl_3$, 400 MHz).

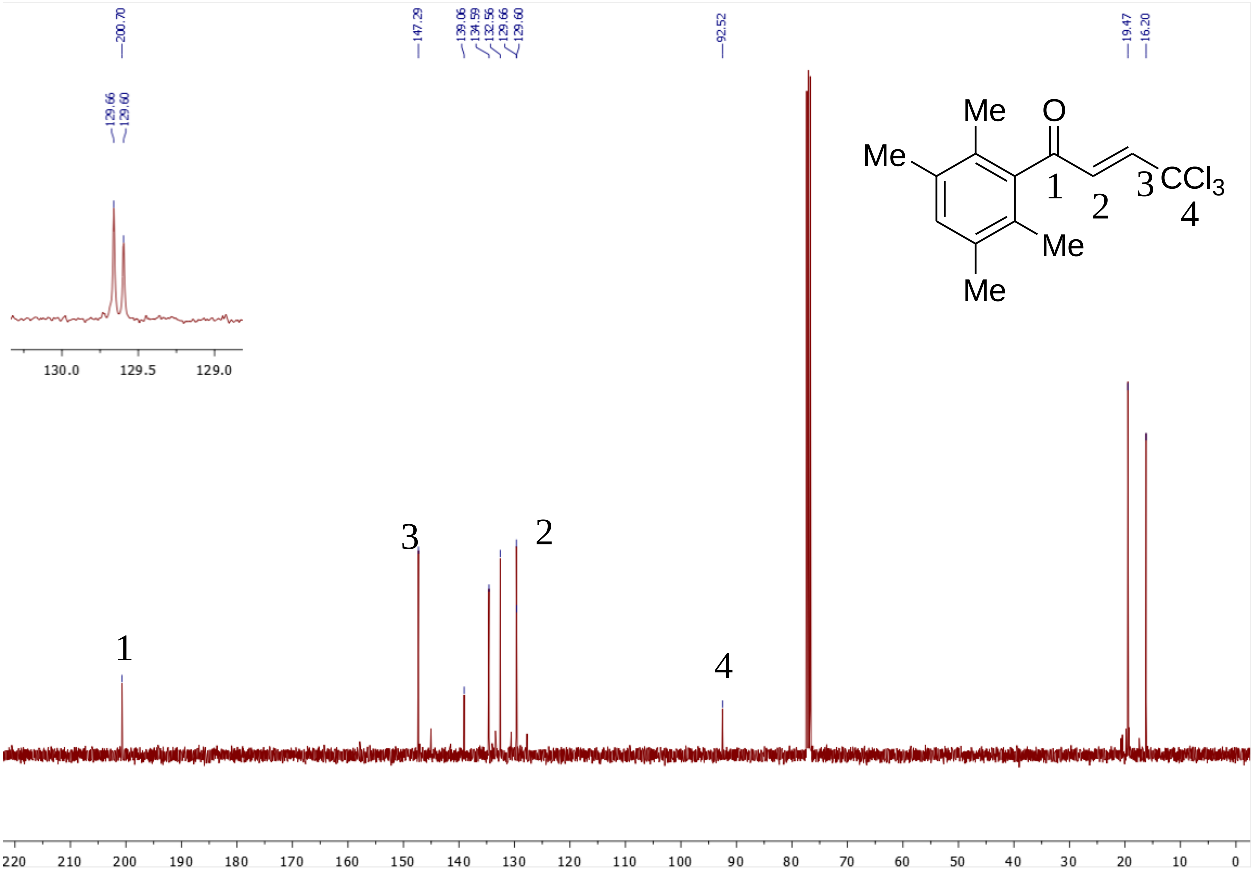

Figure S82. $^{13}C\{^1H\}$ NMR spectrum of the compound **2u** ($CDCl_3$, 101 MHz).

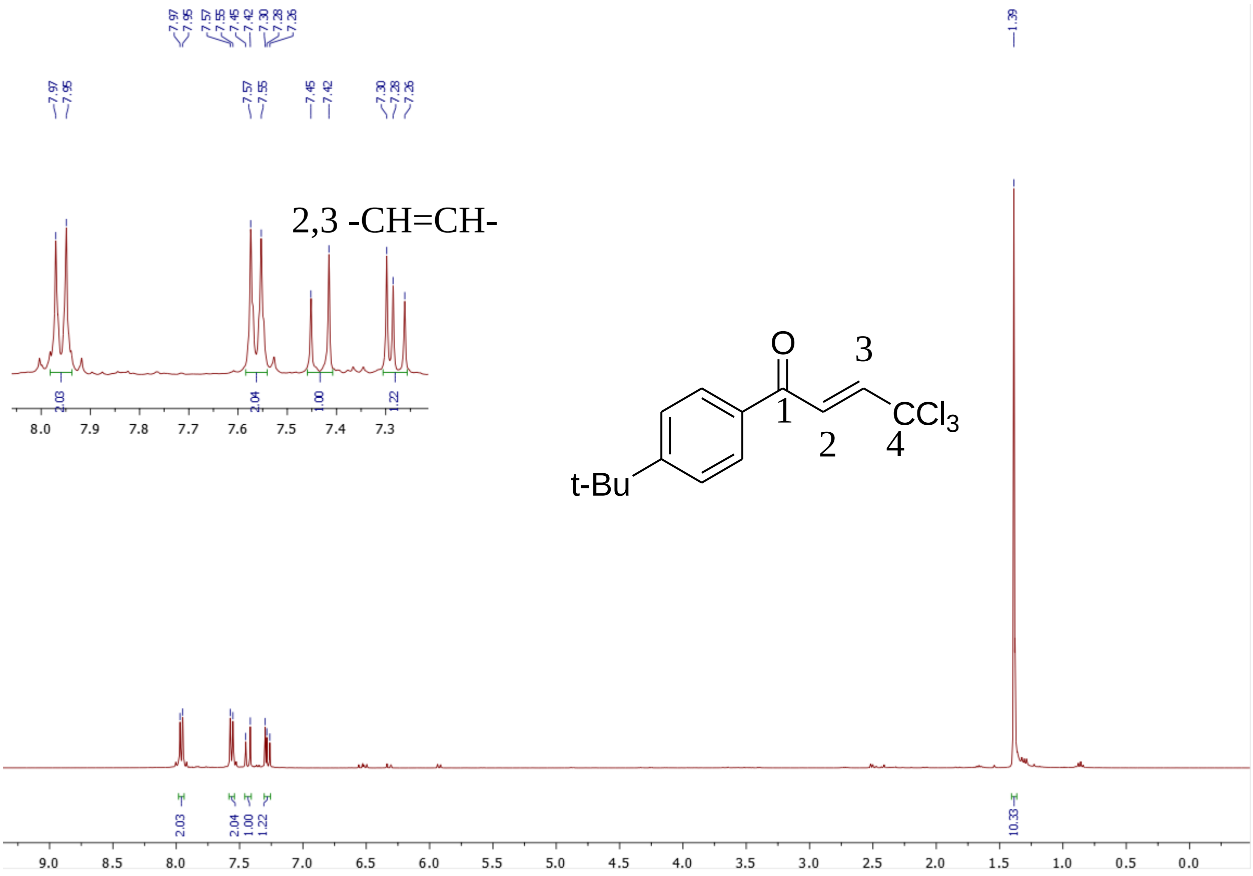

Figure S83. ^{1}H NMR spectrum of the compound **2v** ($CDCl_3$, 400 MHz).

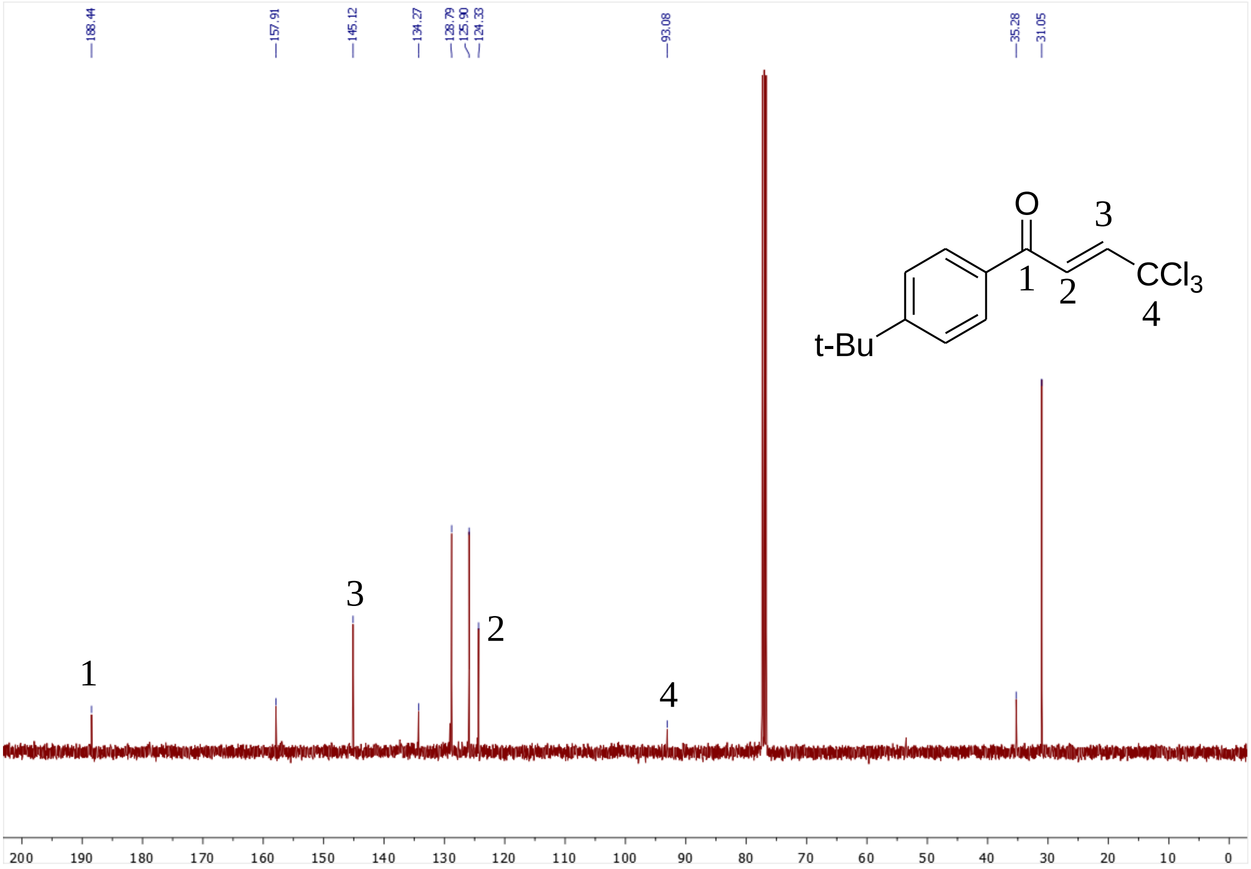

Figure S84. $^{13}C\{^{1}H\}$ NMR spectrum of the compound **2v** ($CDCl_3$, 101 MHz).

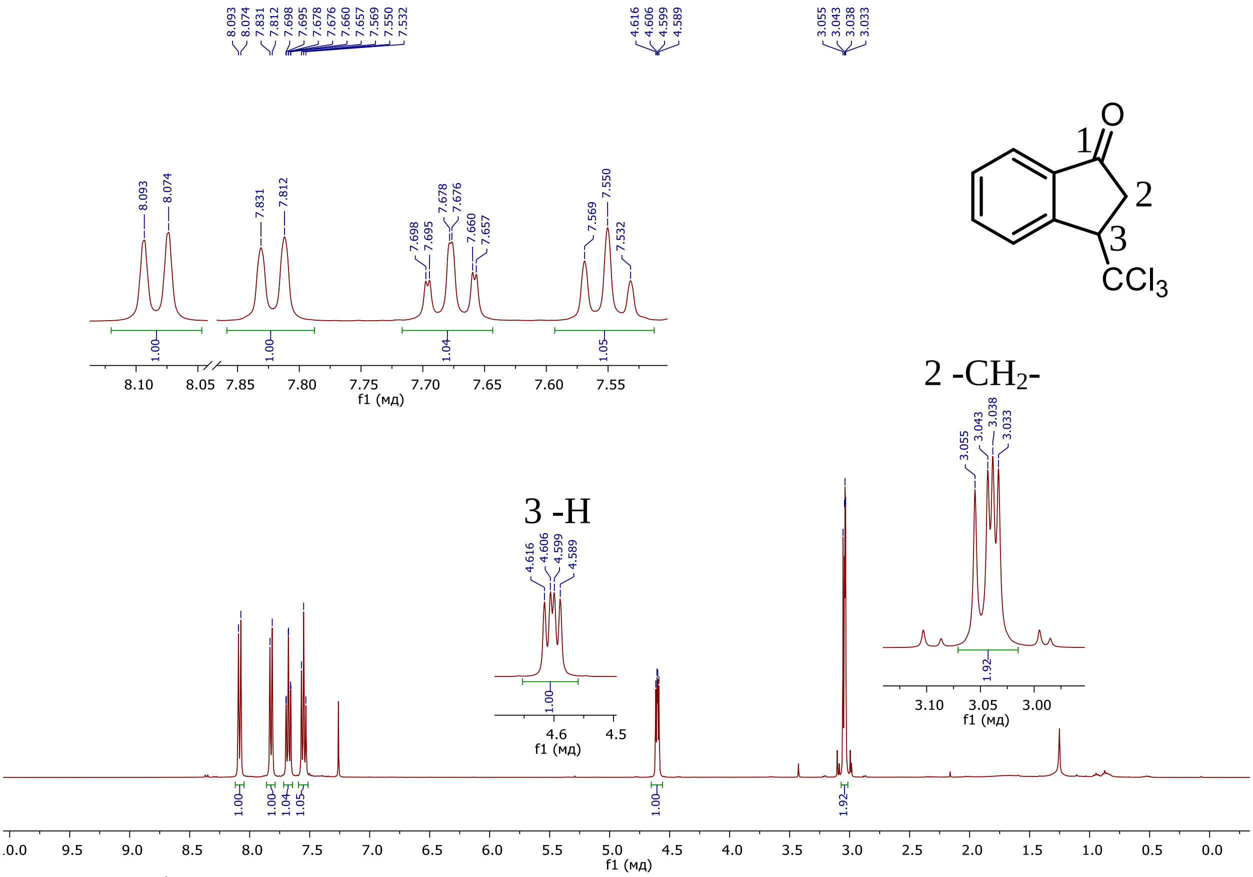

Figure S85. ^{1}H NMR spectrum of the compound **3a** ($CDCl_3$, 400 MHz).

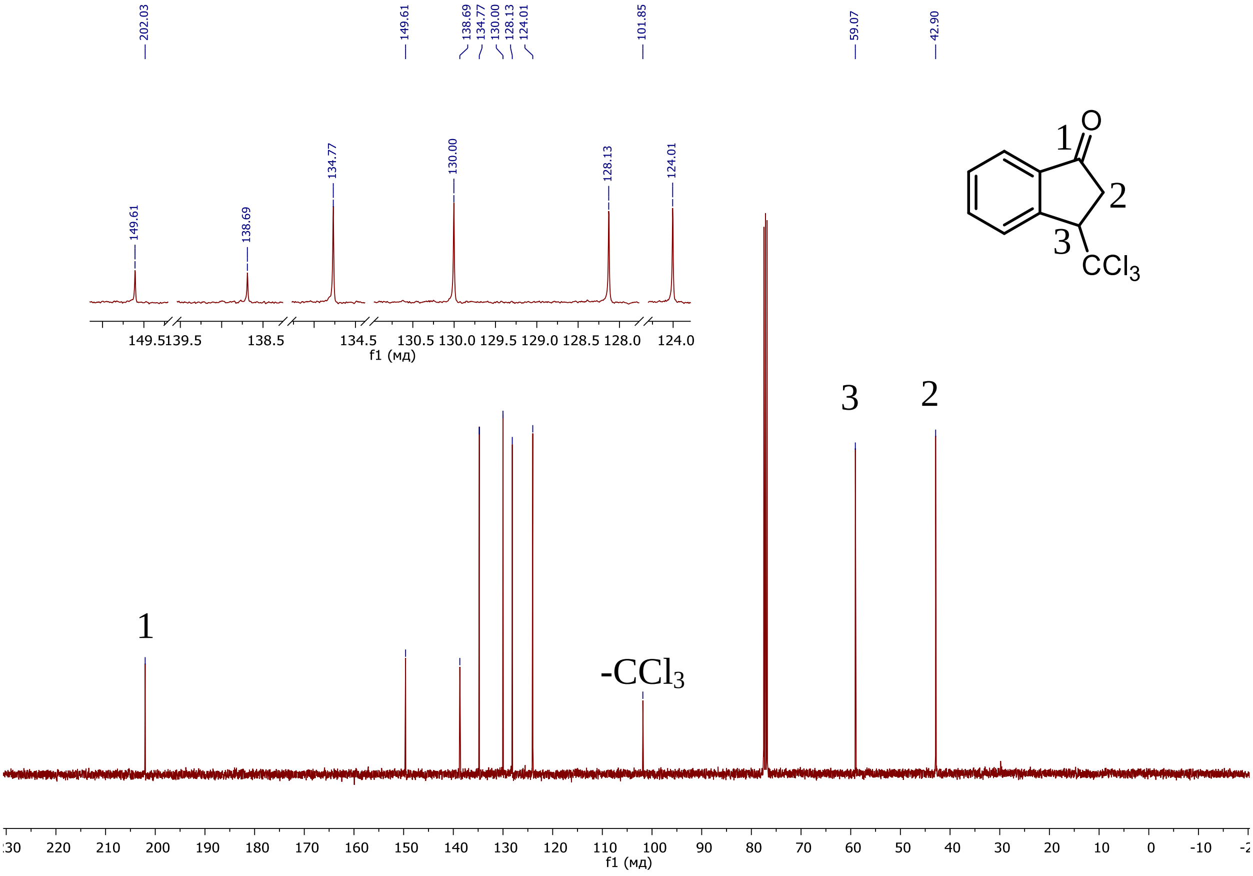

Figure S86. $^{13}C\{^1H\}$ NMR spectrum of the compound **3a** ($CDCl_3$, 101 MHz).

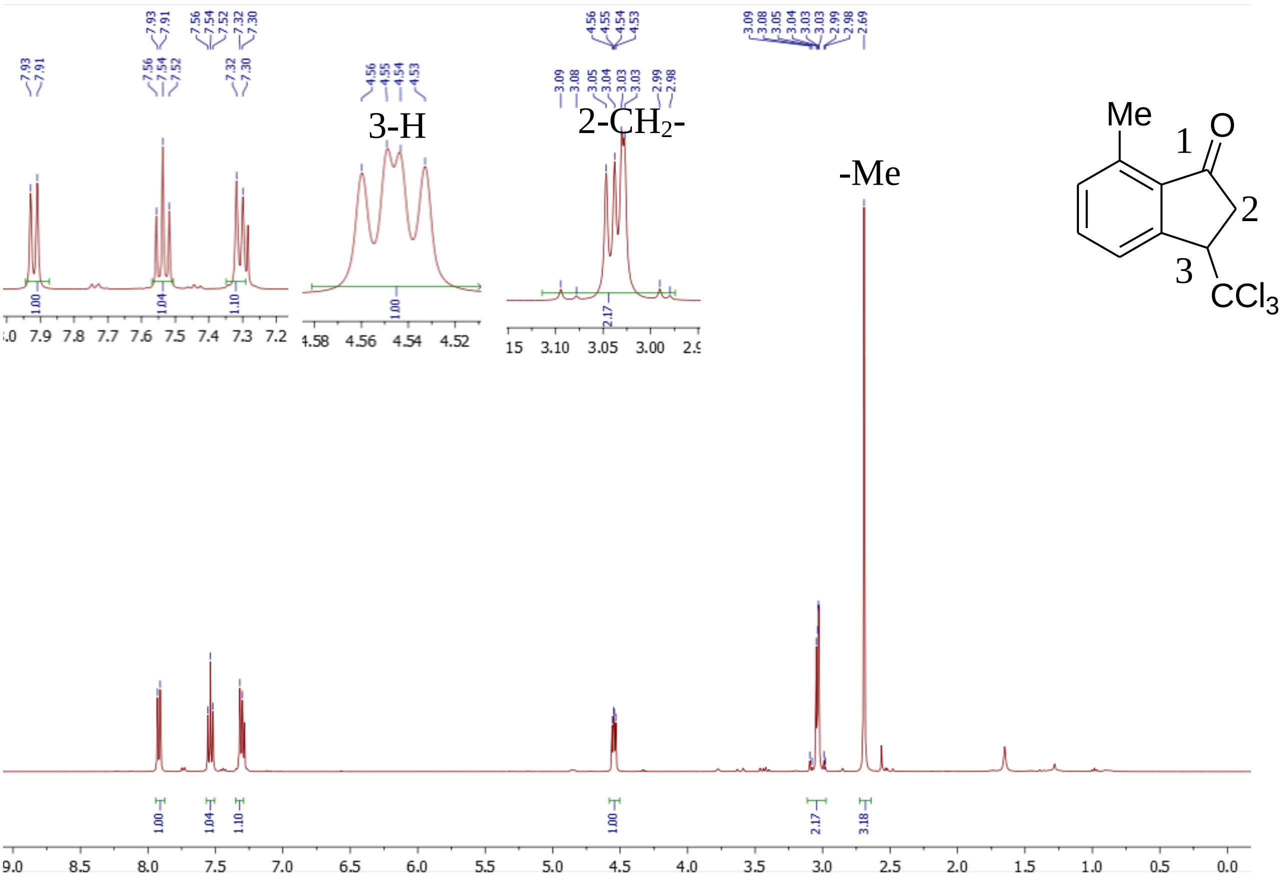

Figure S87. ^{1}H NMR spectrum of the compound **3b** ($CDCl_3$, 400 MHz).

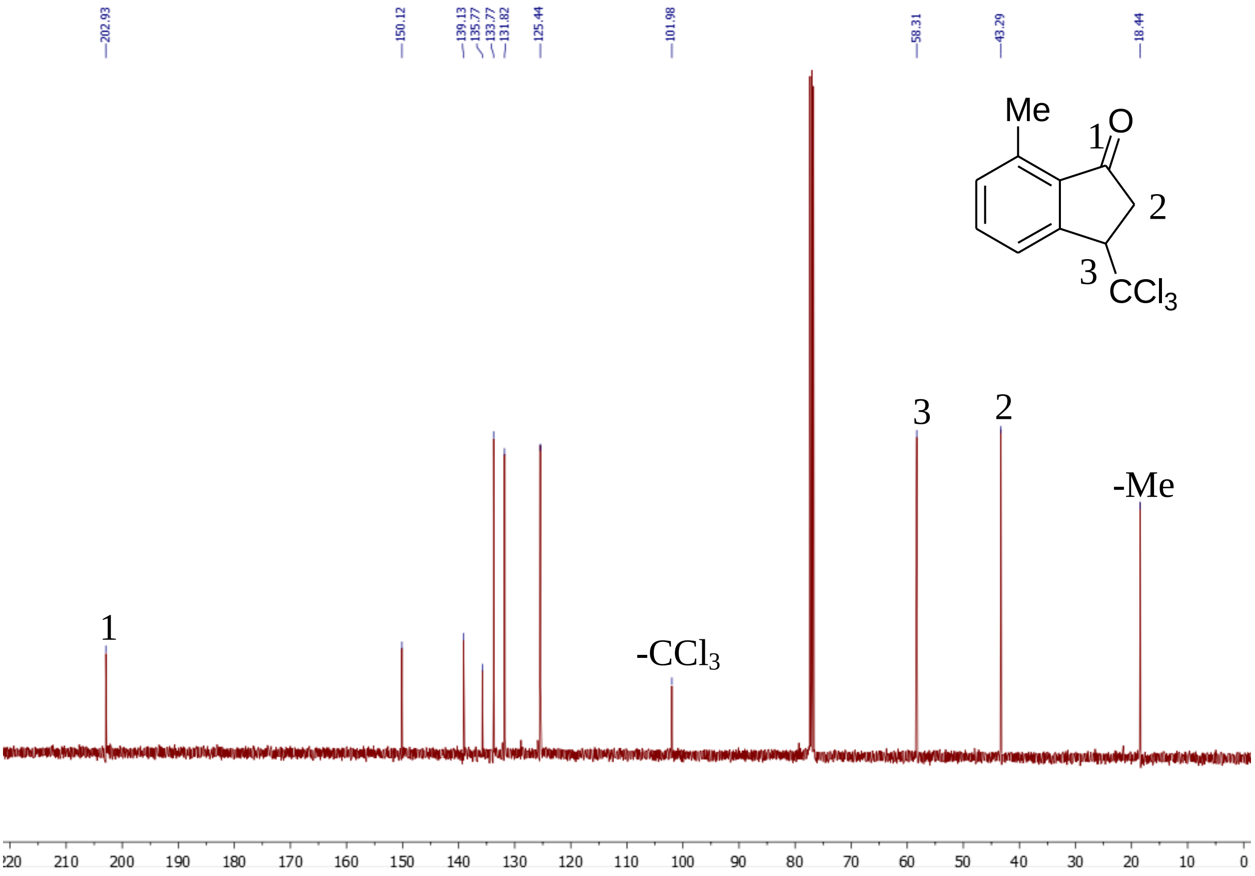

Figure S88. $^{13}C\{^1H\}$ NMR spectrum of the compound **3b** ($CDCl_3$, 101 MHz).

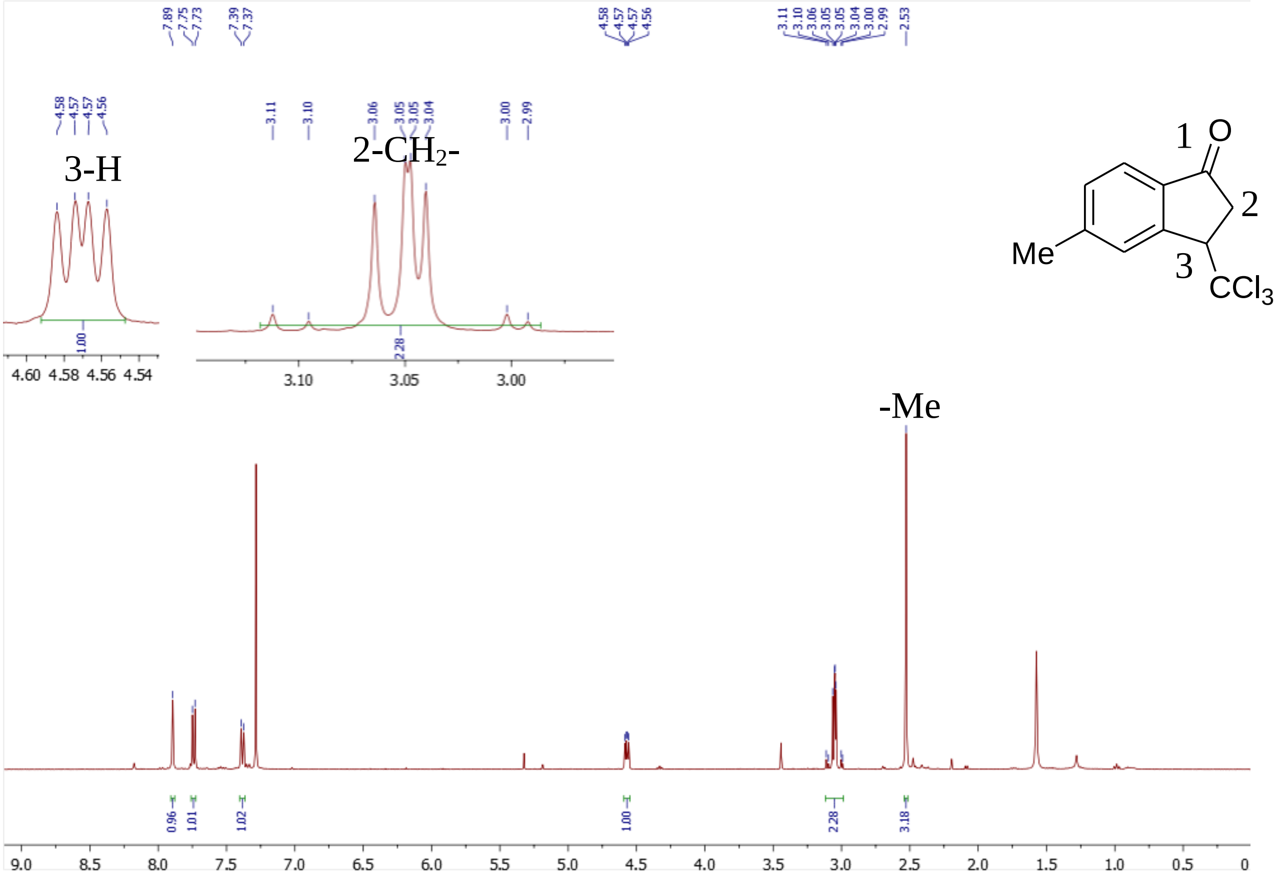

Figure S89. ^{1}H NMR spectrum of the compound **3c** ($CDCl_3$, 400 MHz).

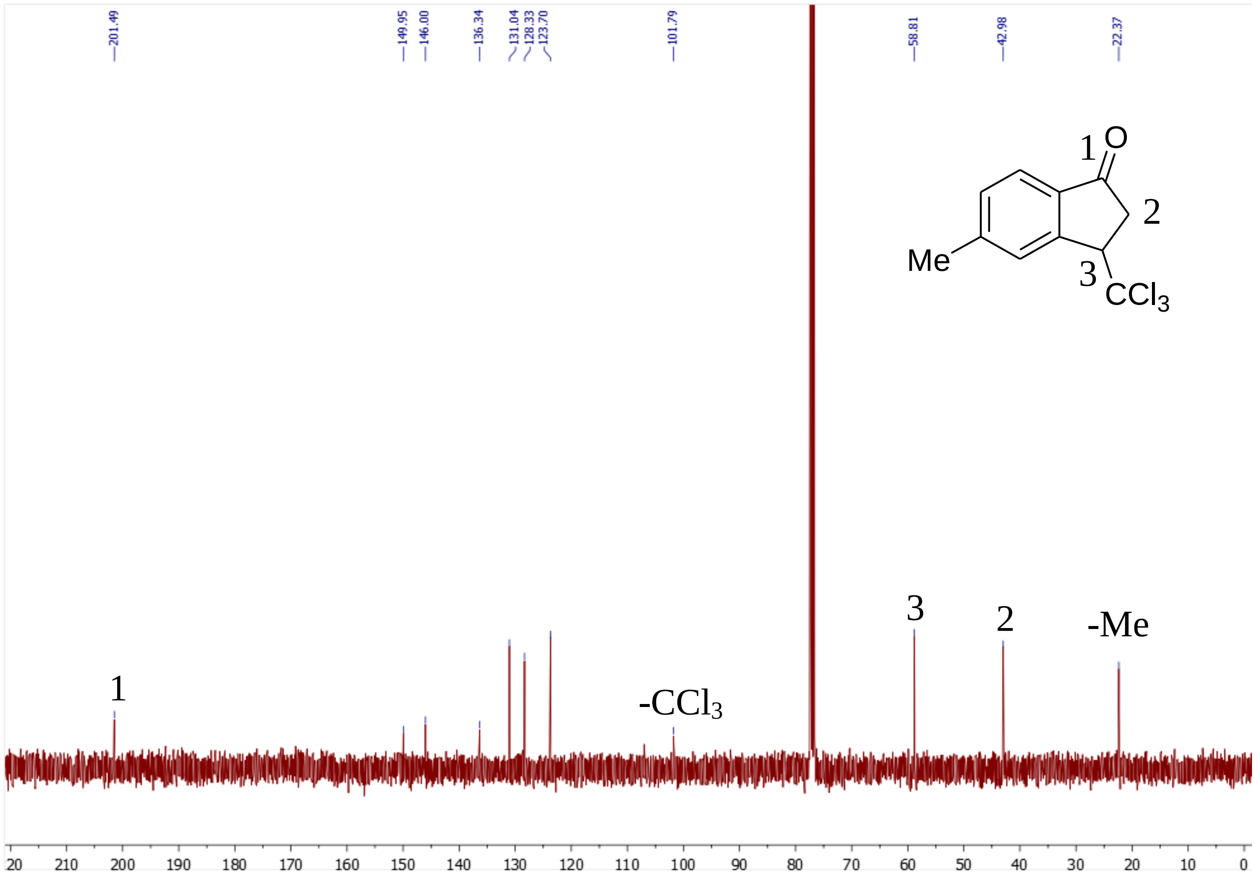

Figure S90. $^{13}C\{^1H\}$ NMR spectrum of the compound **3c** ($CDCl_3$, 101 MHz).

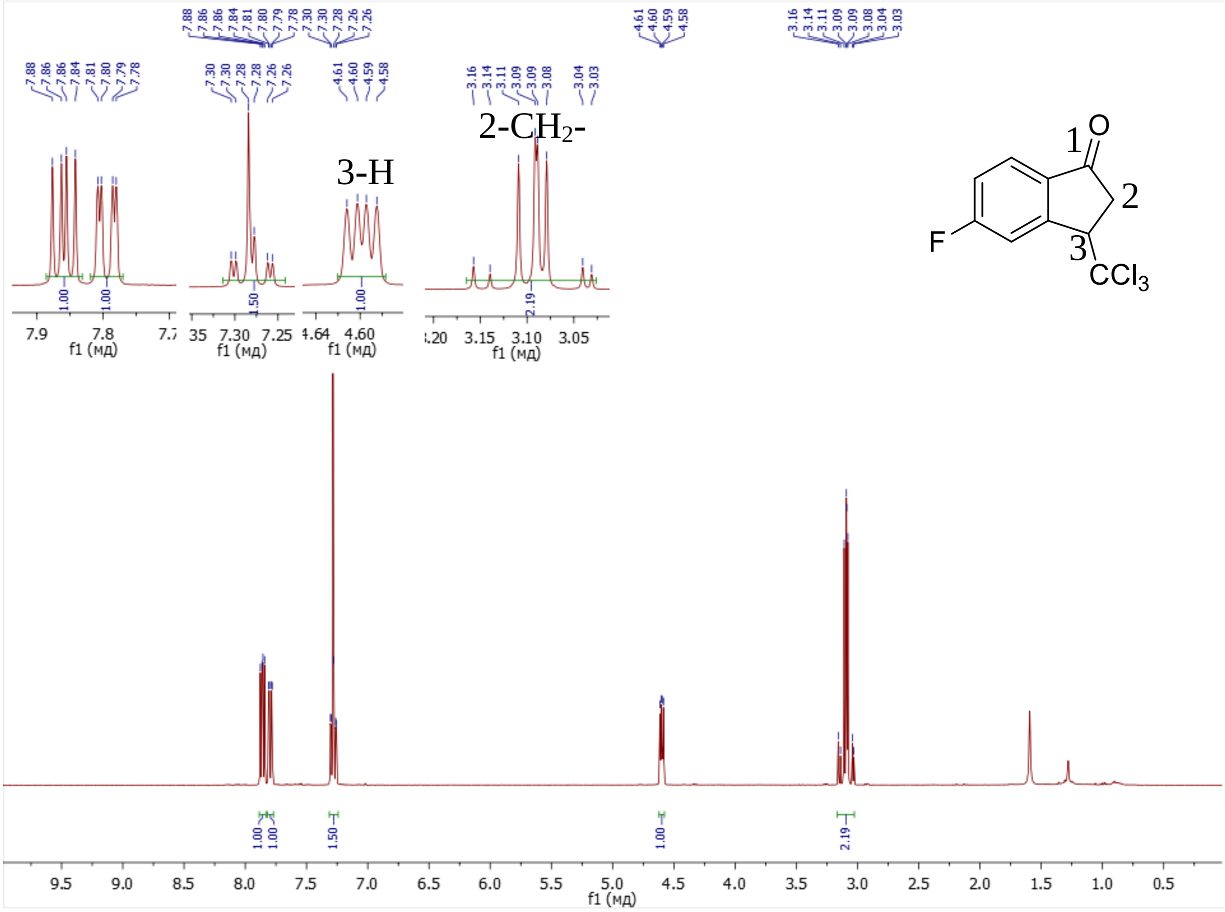

Figure S91. ^{1}H NMR spectrum of the compound **3d** ($CDCl_3$, 400 MHz).

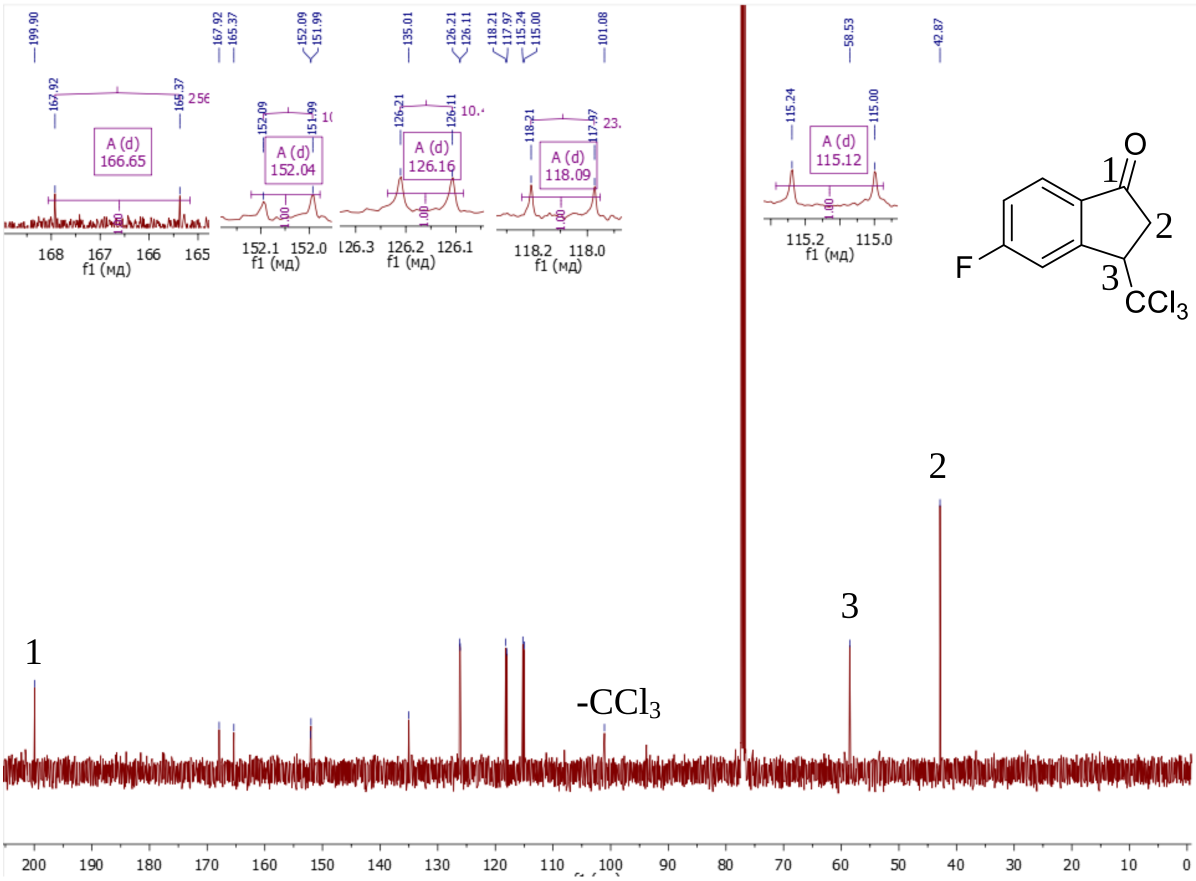

Figure S92. ^{13}C{^{1}H} NMR spectrum of the compound **3d** ($CDCl_3$, 101 MHz).

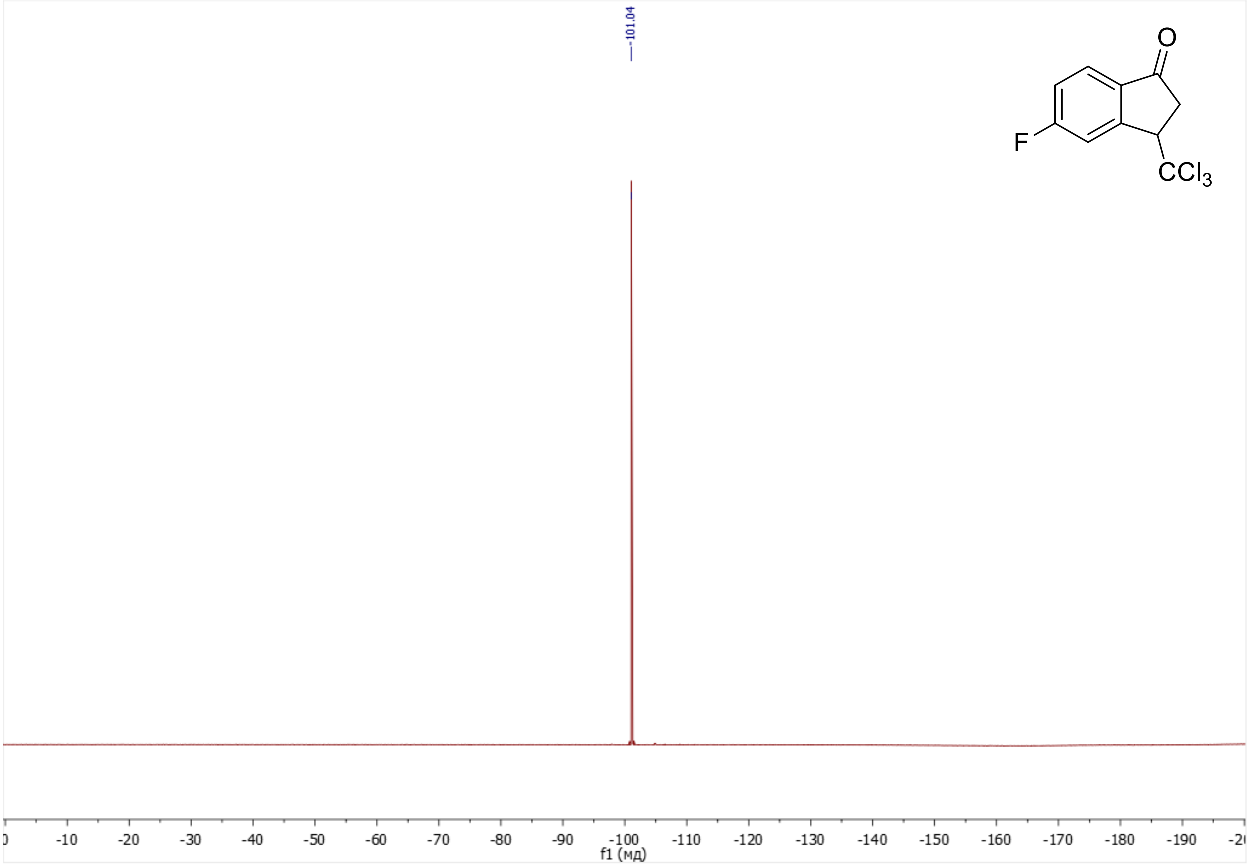

Figure S93. $^{19}F\{^1H\}$ NMR spectrum of the compound **3d** ($CDCl_3$, 376 MHz).

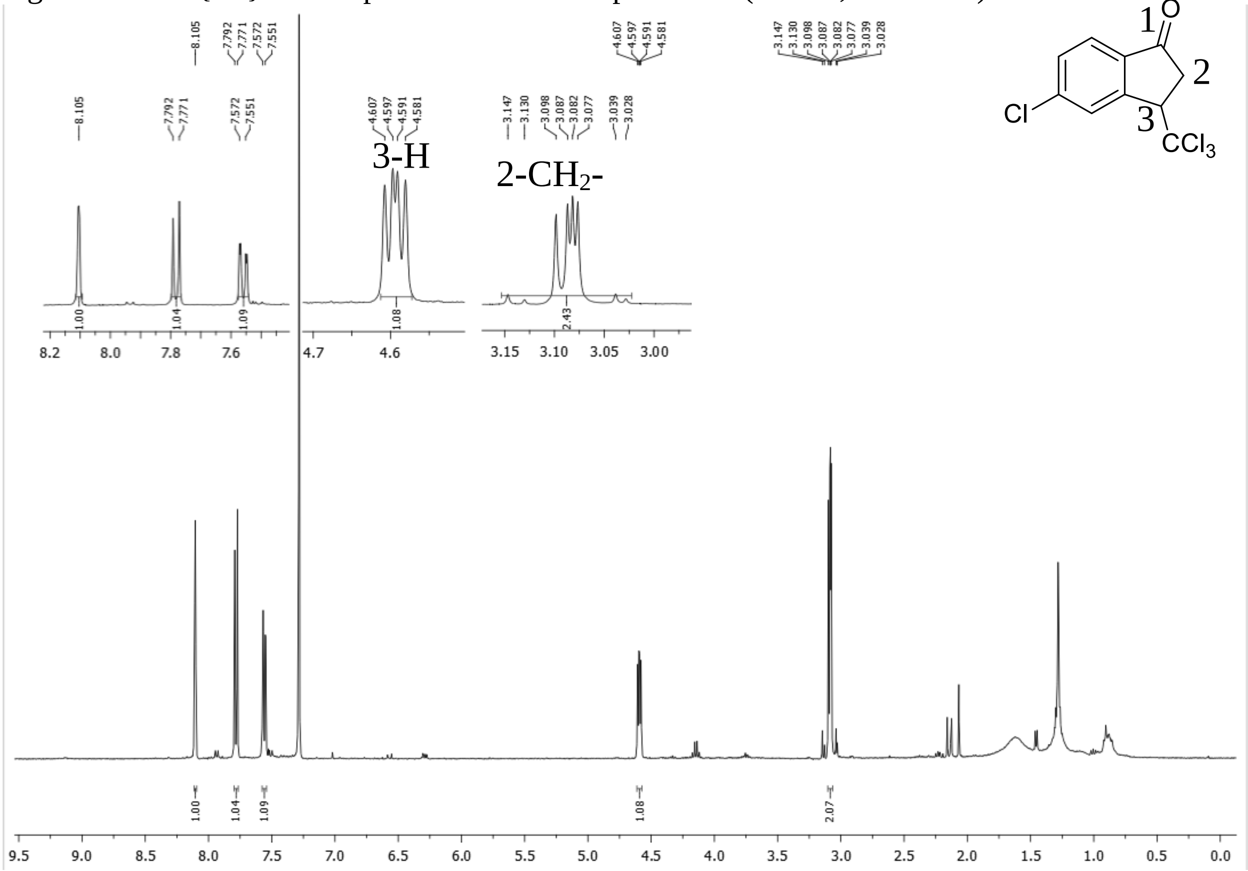

Figure S94. ^{1}H NMR spectrum of the compound **3e** ($CDCl_3$, 400 MHz).

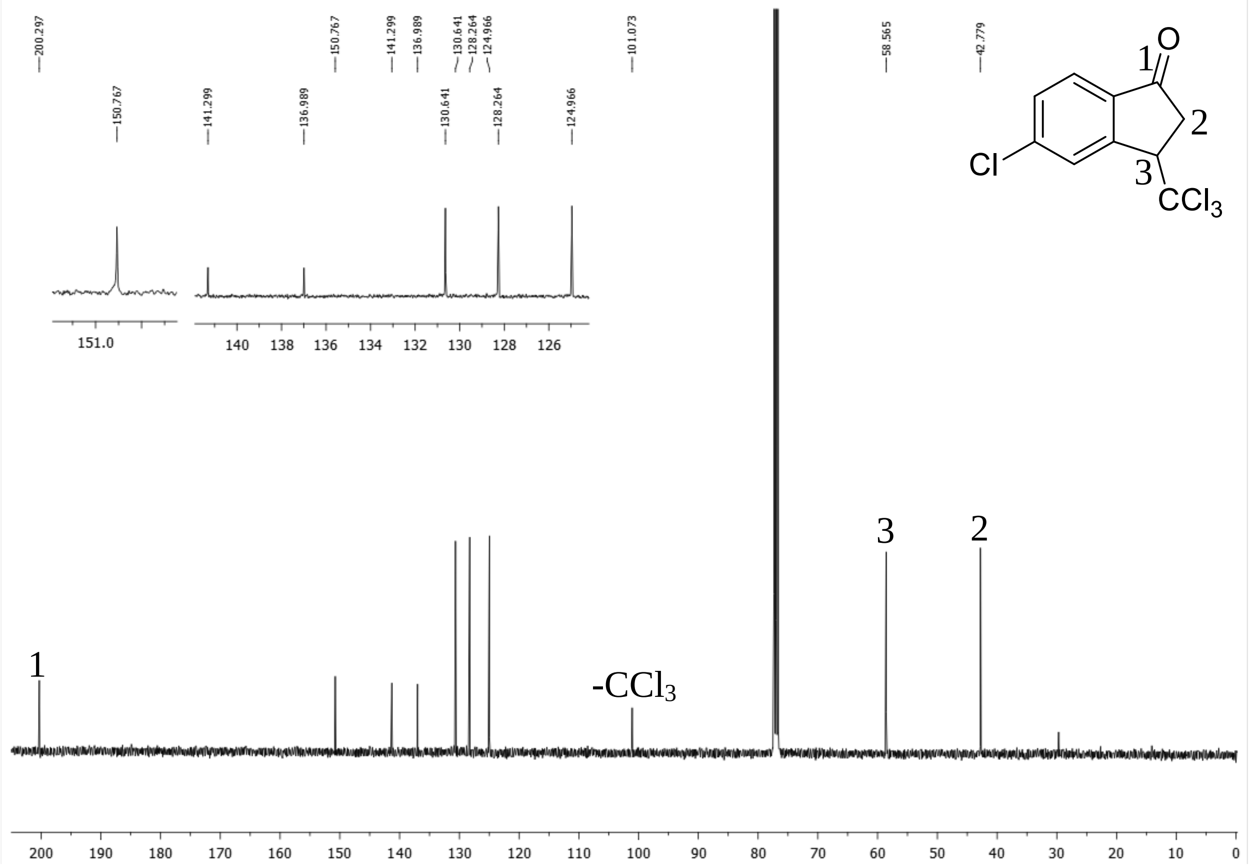

Figure S95. $^{13}C\{^{1}H\}$ NMR spectrum of the compound **3e** ($CDCl_3$, 101 MHz).

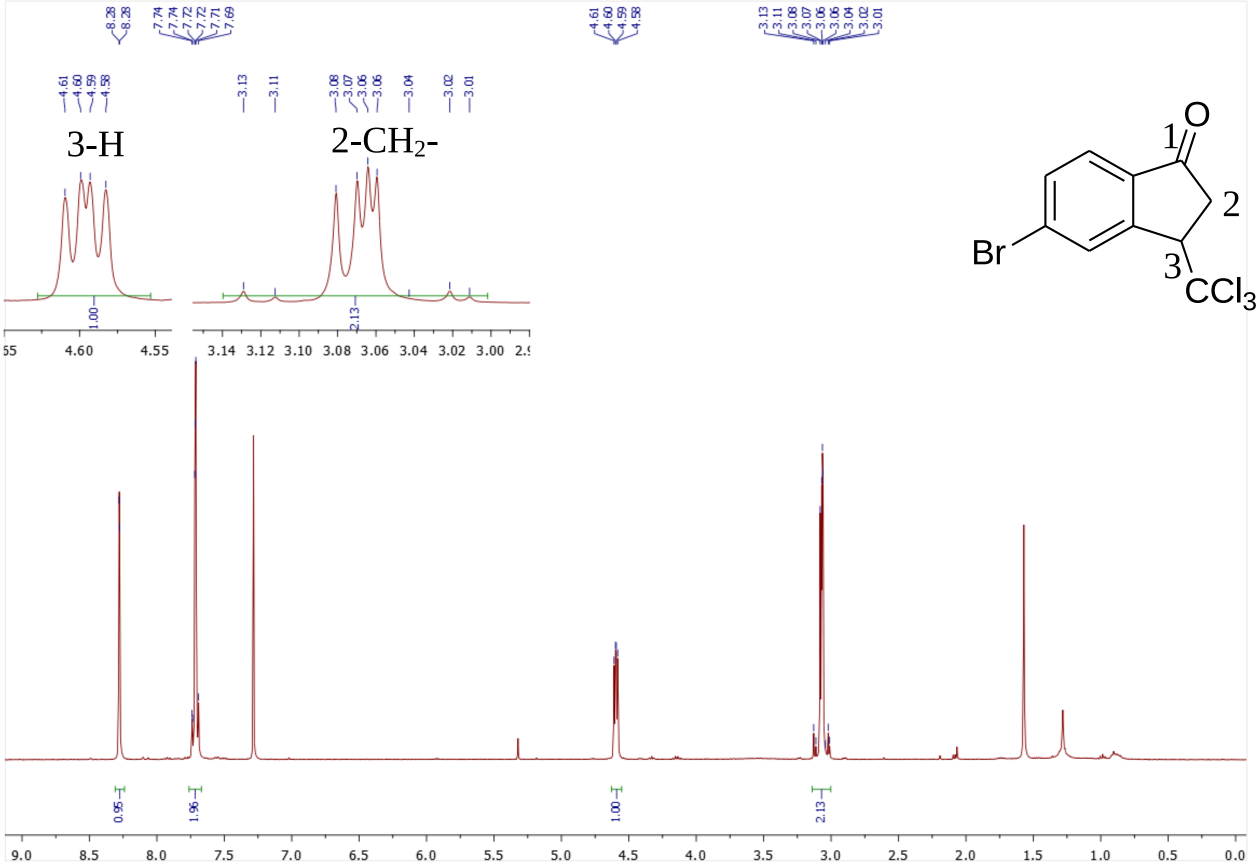

Figure S96. ^{1}H NMR spectrum of the compound **3f** ($CDCl_3$, 400 MHz).

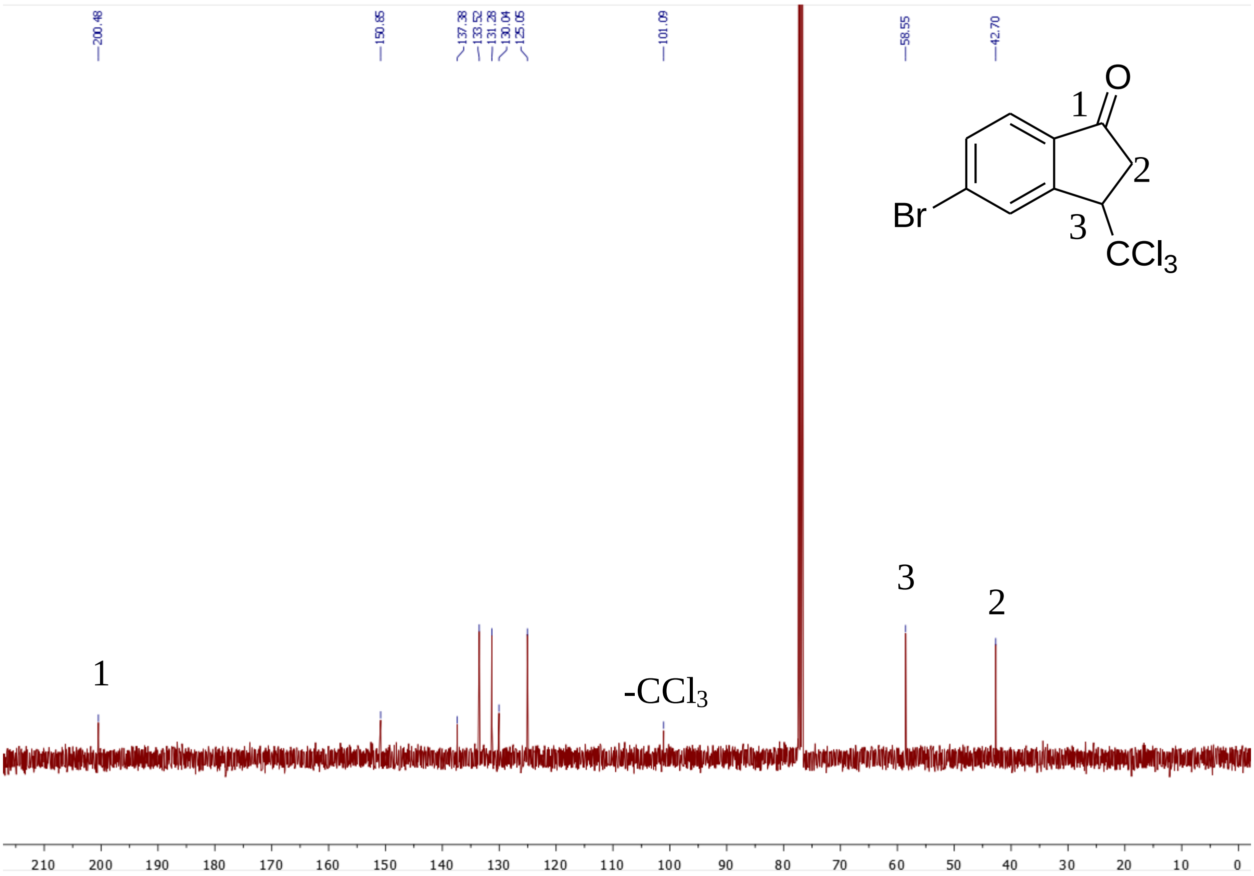

Figure S97. $^{13}C\{^{1}H\}$ NMR spectrum of the compound **3f** ($CDCl_3$, 101 MHz).

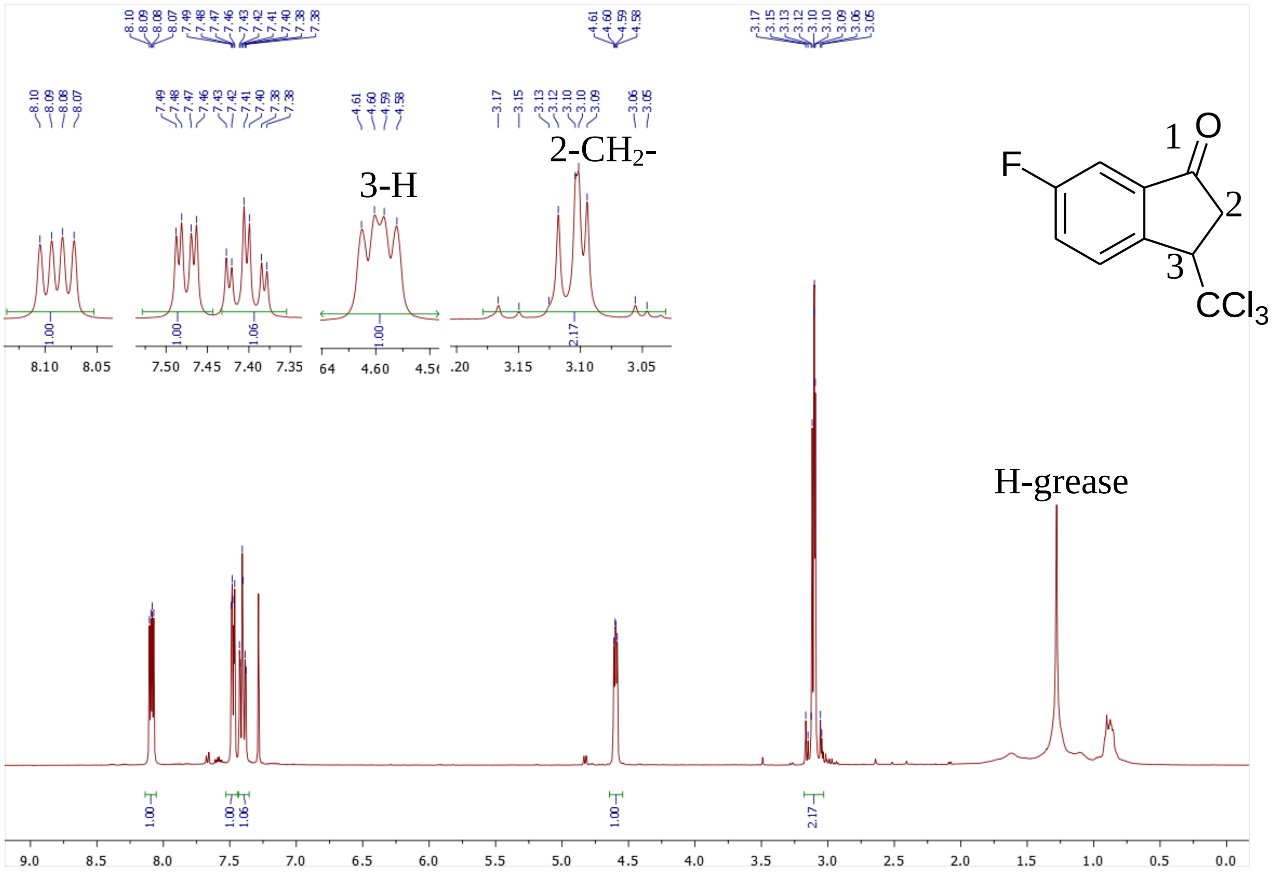

Figure S98. ^{1}H NMR spectrum of the compound **3g** ($CDCl_3$, 400 MHz).

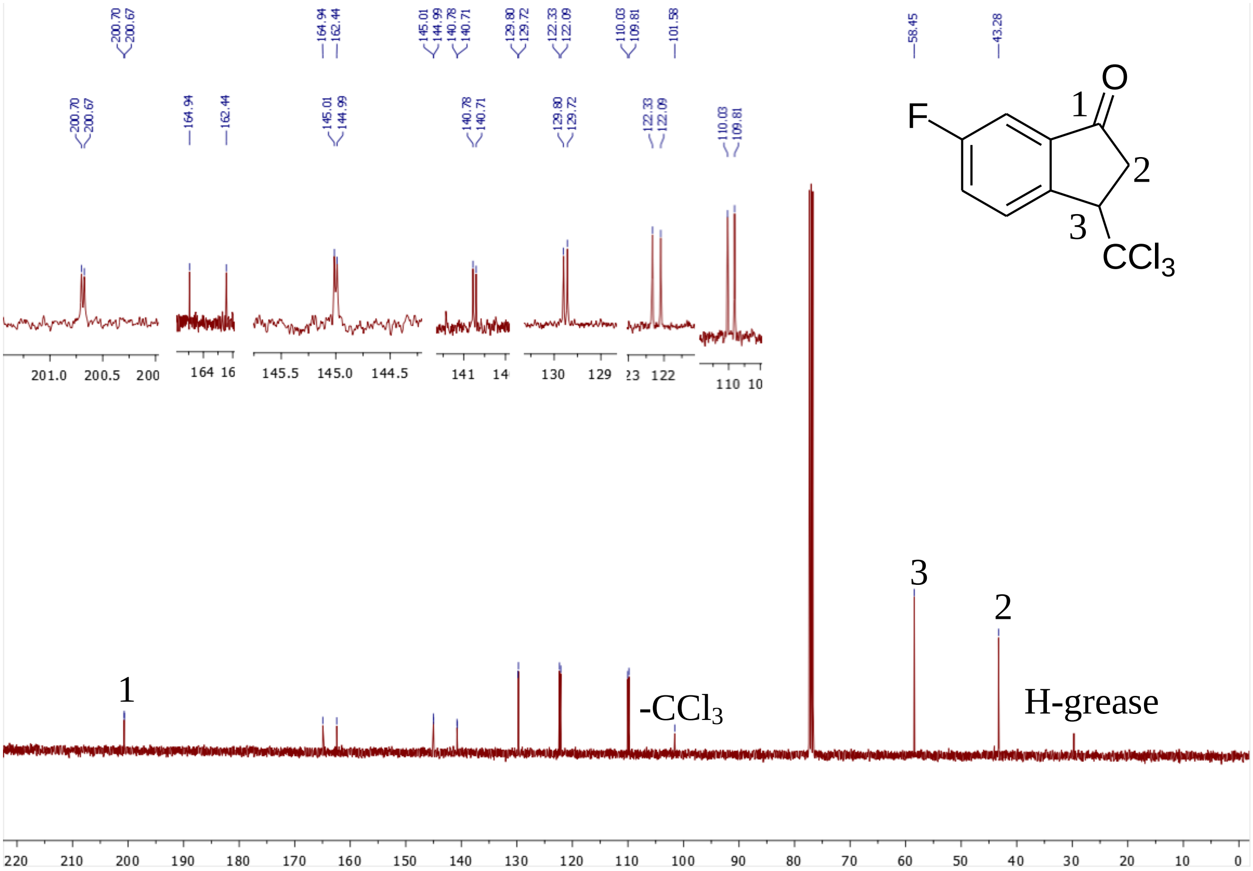

Figure S99. $^{13}C\{^{1}H\}$ NMR spectrum of the compound **3g** ($CDCl_3$, 101 MHz).

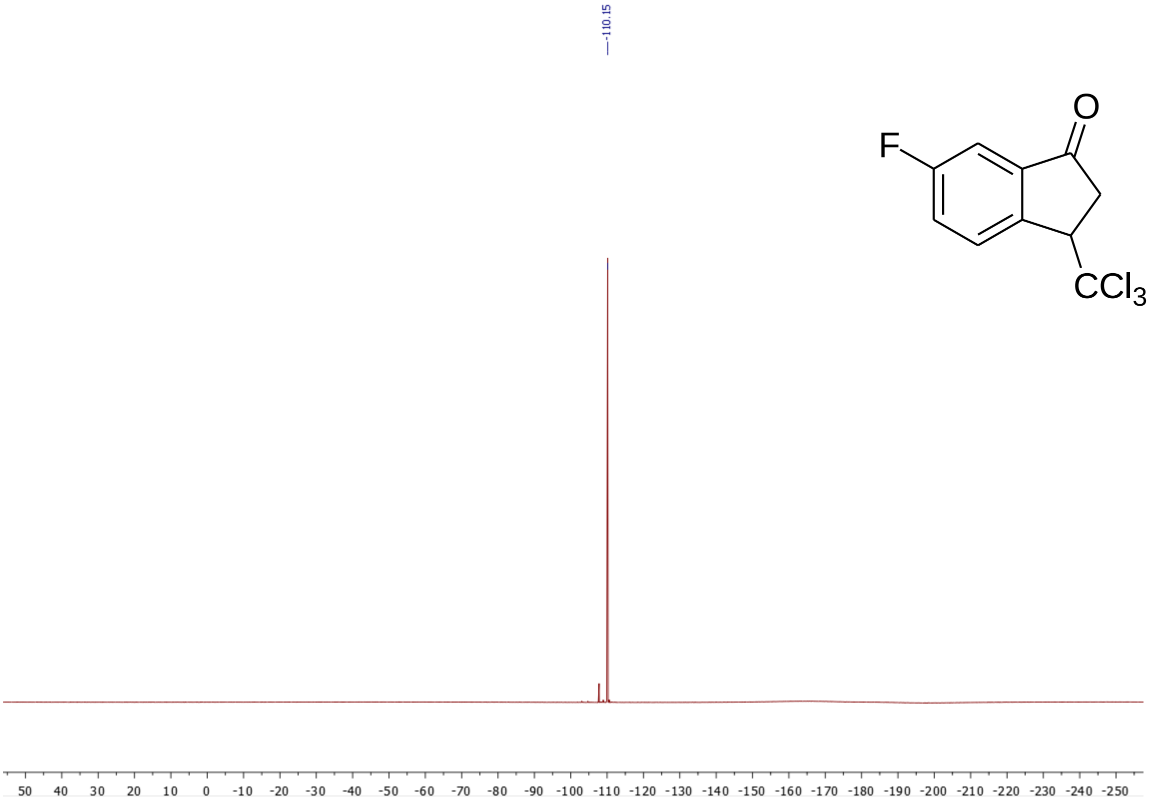

Figure S100. $^{19}F\{^{1}H\}$ NMR spectrum of the compound **3g** ($CDCl_3$, 376 MHz).

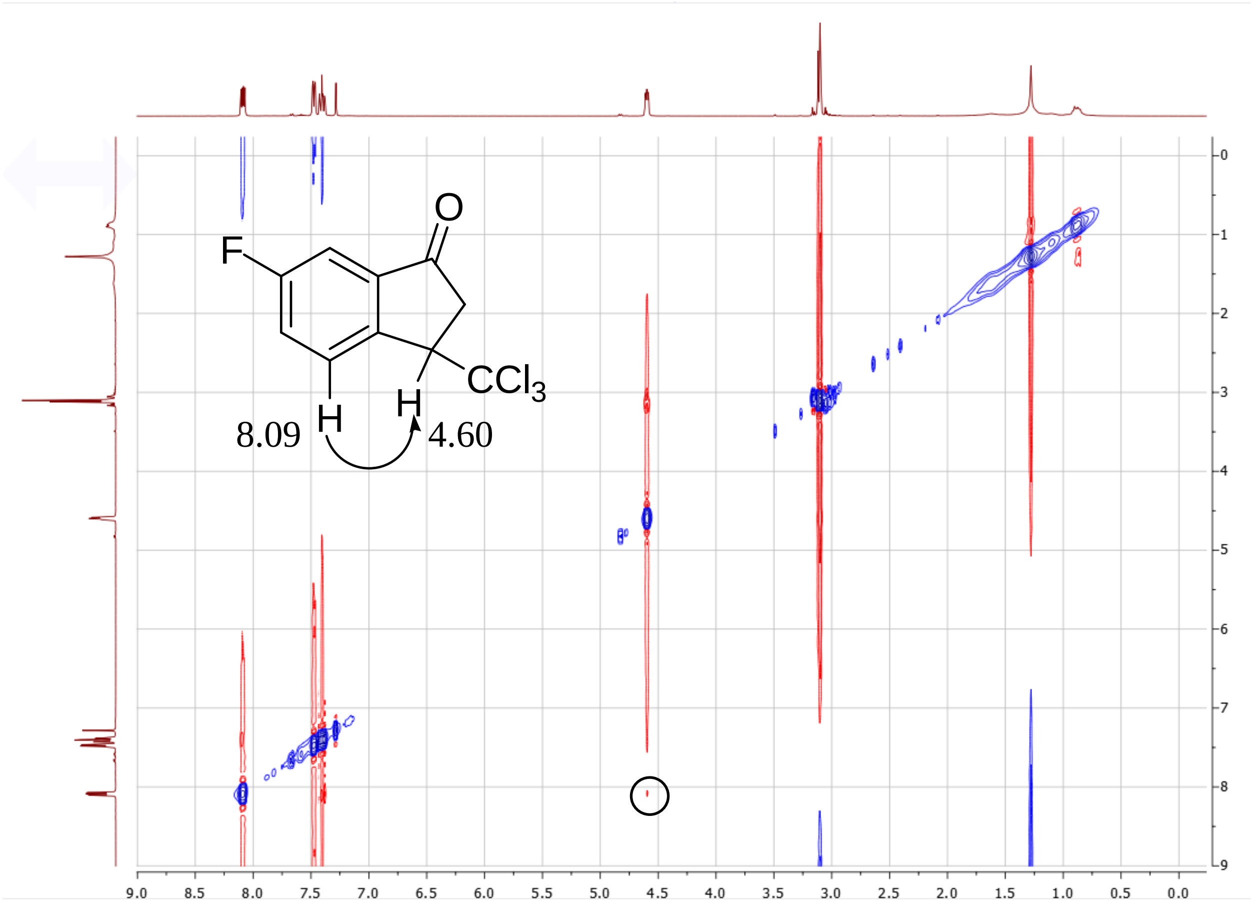

Figure S101. $^{1}H,^{1}H$ NOESY NMR spectrum of compound **3g** (400 MHz, $CDCl_3$).

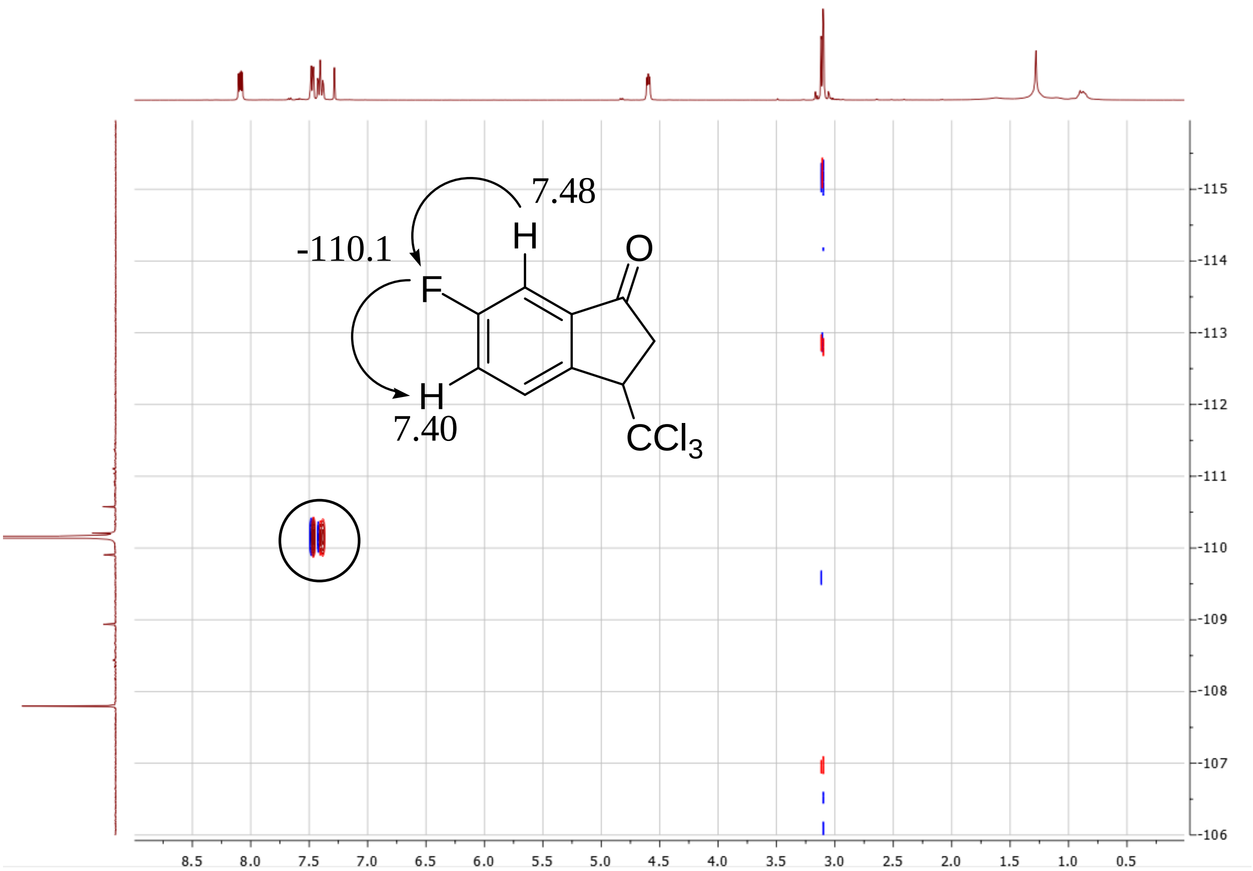

Figure S102. $^{1}H,^{19}F$ NOESY NMR spectrum of compound **3g** (400 MHz, $CDCl_3$).

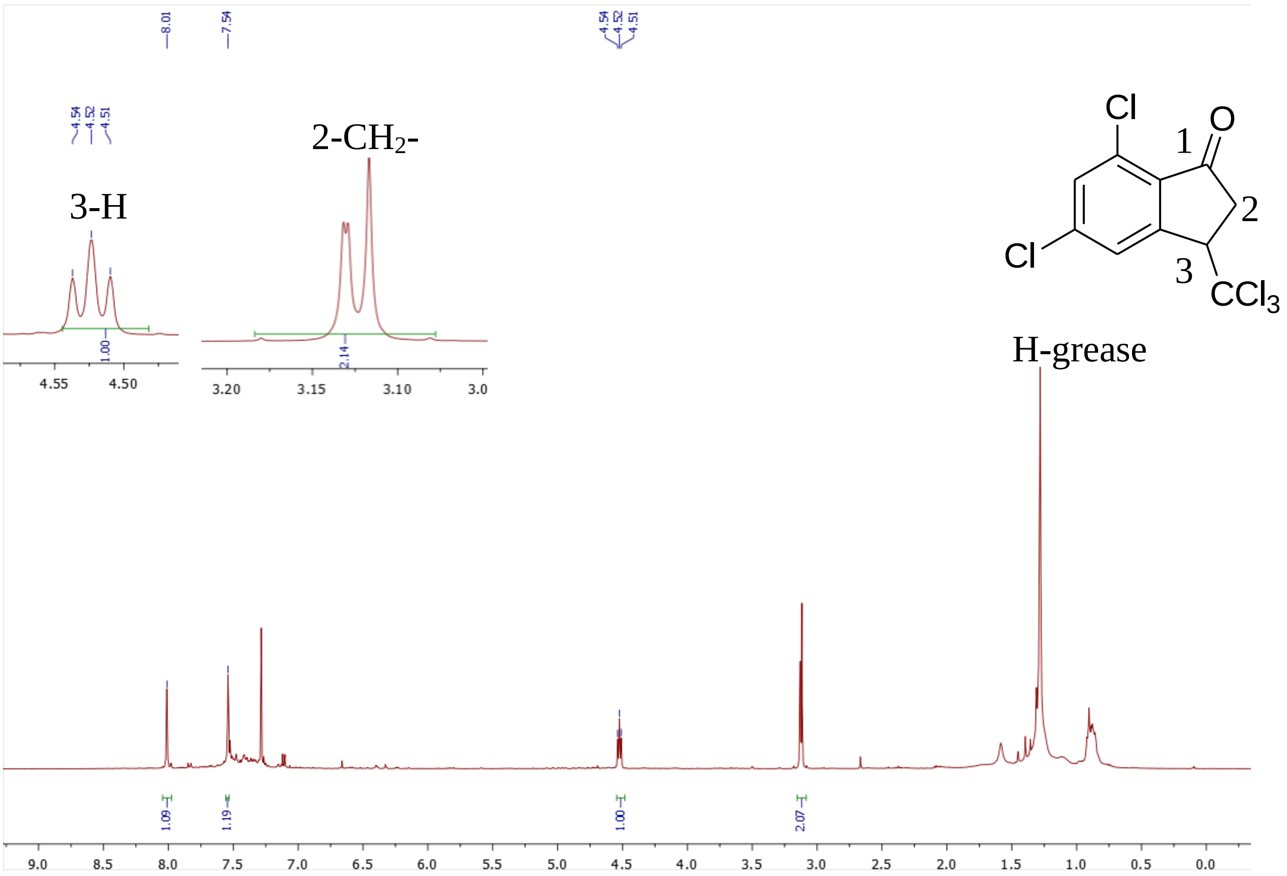

Figure S103. ^{1}H NMR spectrum of the compound **3h** ($CDCl_3$, 400 MHz).

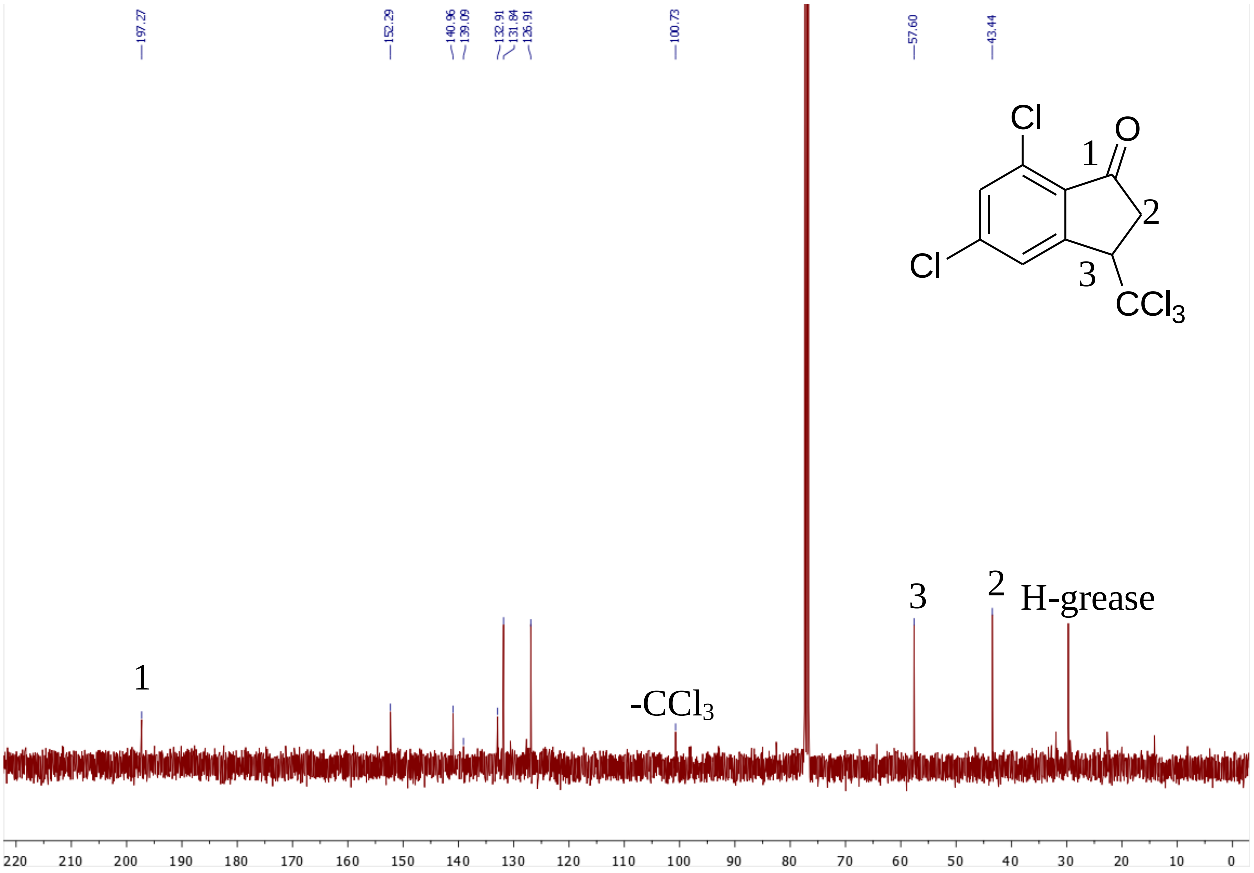

Figure S104. ^{13}C{^{1}H} NMR spectrum of the compound **3h** ($CDCl_3$, 101 MHz).

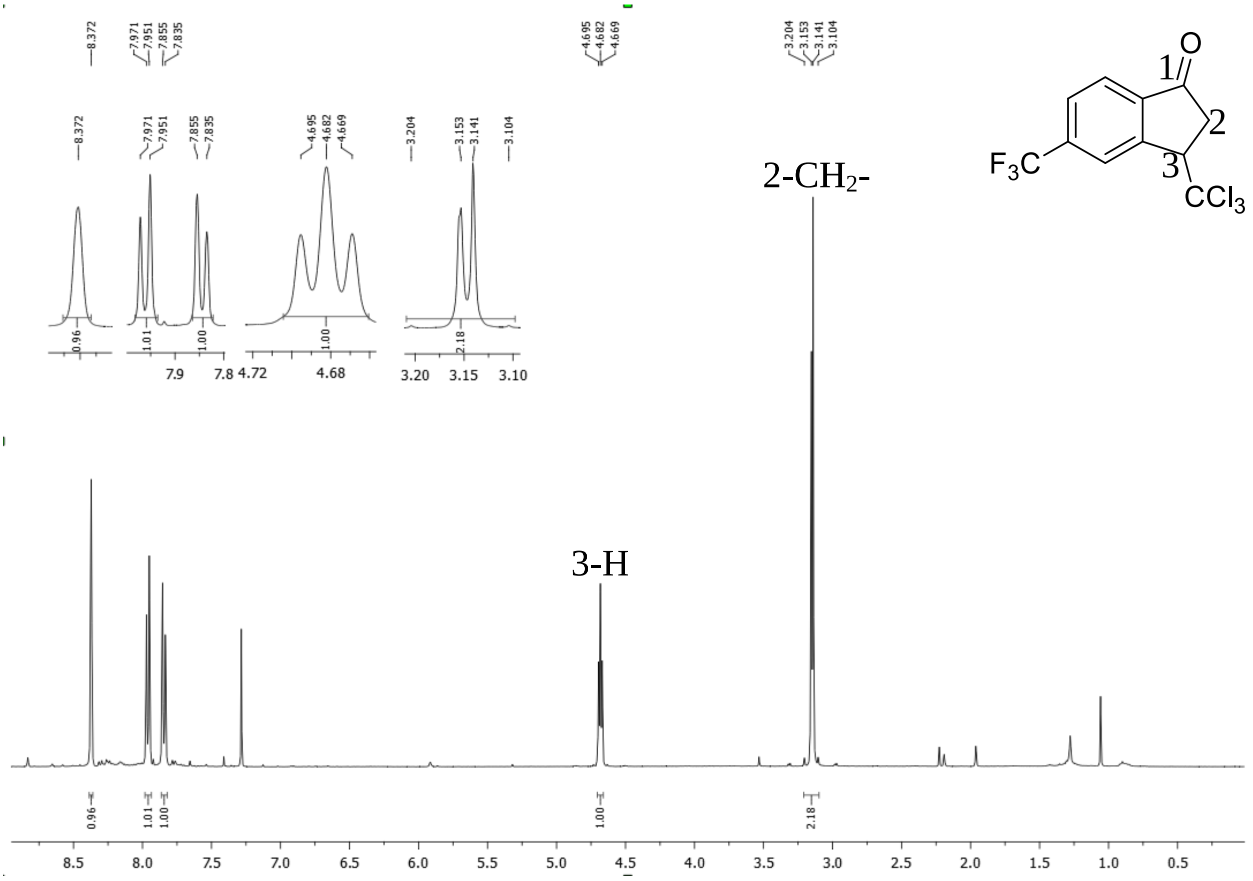

Figure S105. ^{1}H NMR spectrum of the compound **3i** ($CDCl_3$, 400 MHz).

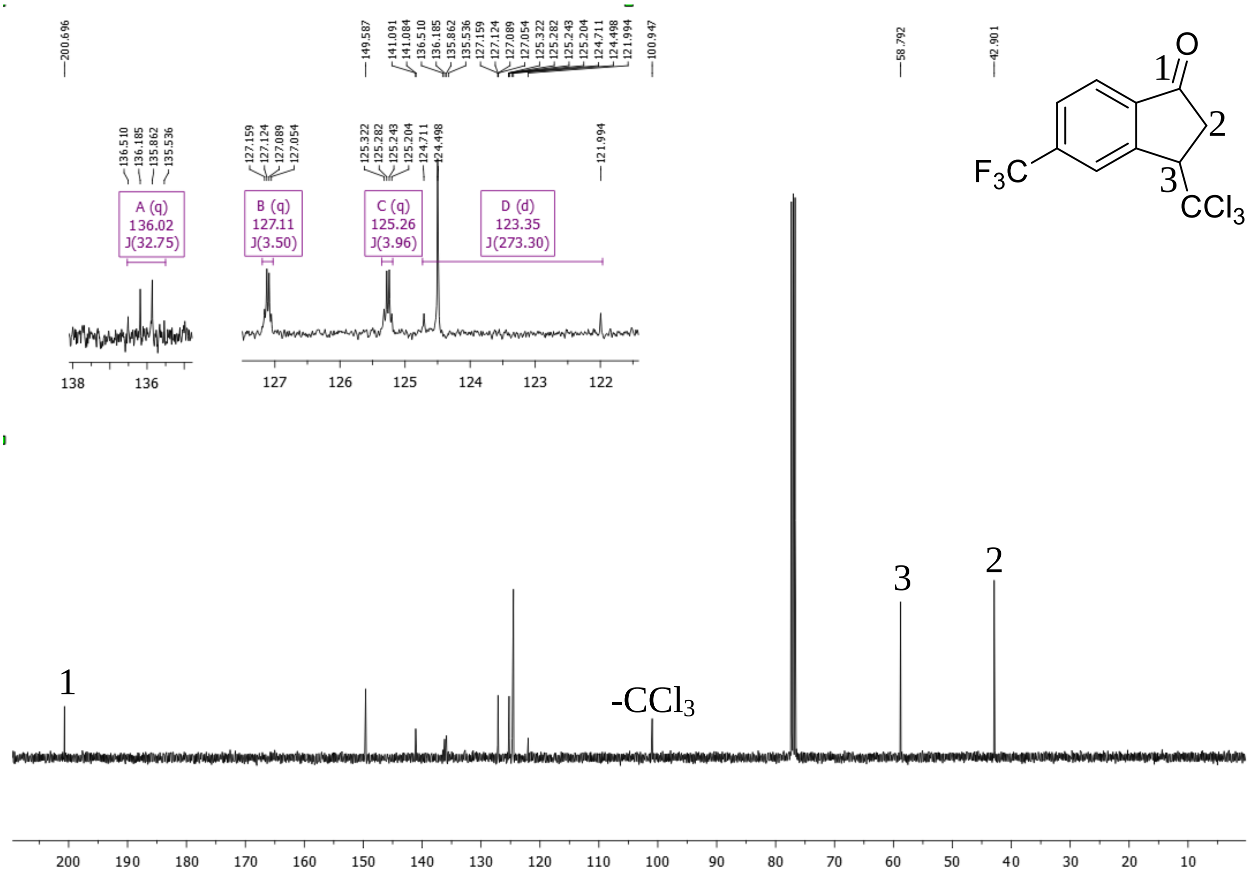

Figure S106. ^{13}C{^{1}H} NMR spectrum of the compound **3i** ($CDCl_3$, 101 MHz).

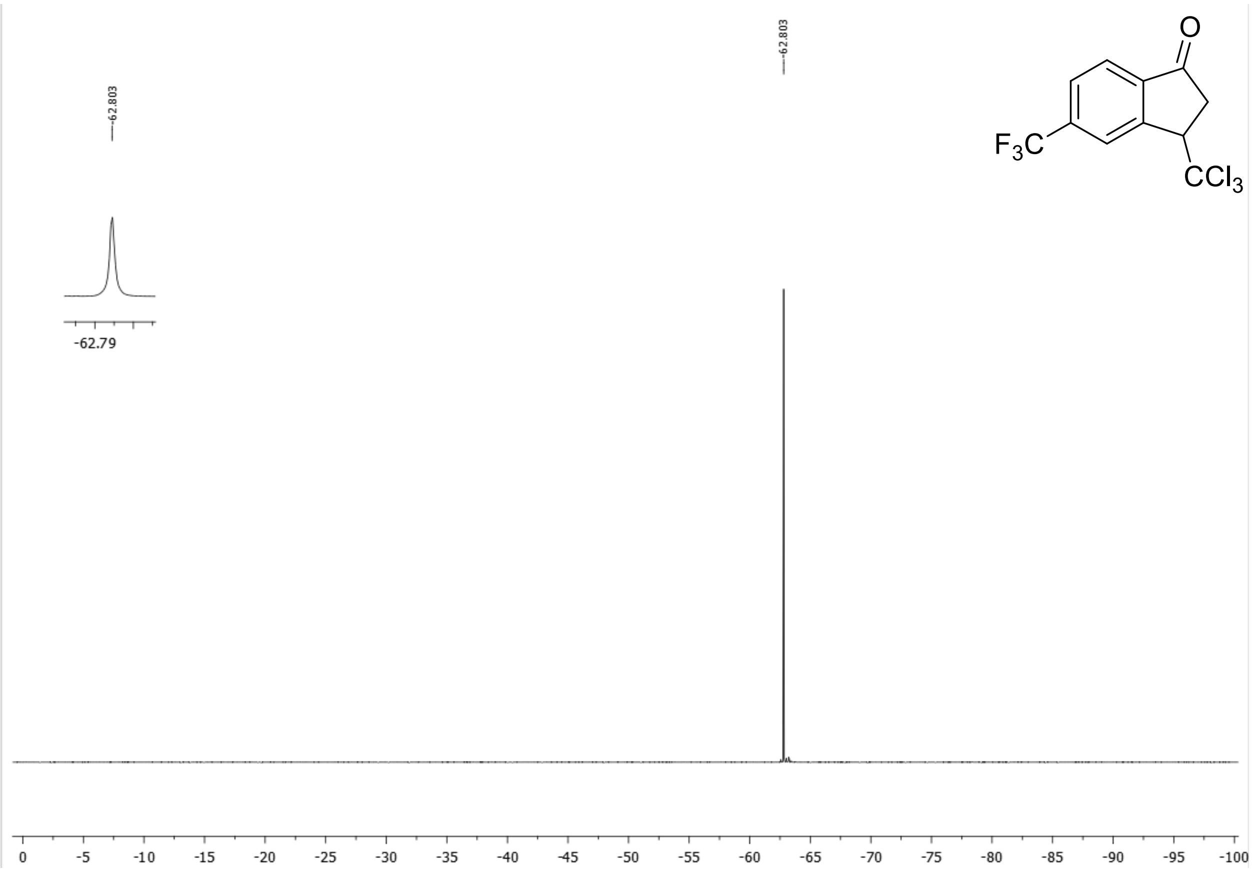

Figure S107 $^{19}F\{^{1}H\}$ NMR spectrum of the compound **3i** ($CDCl_3$, 376 MHz).

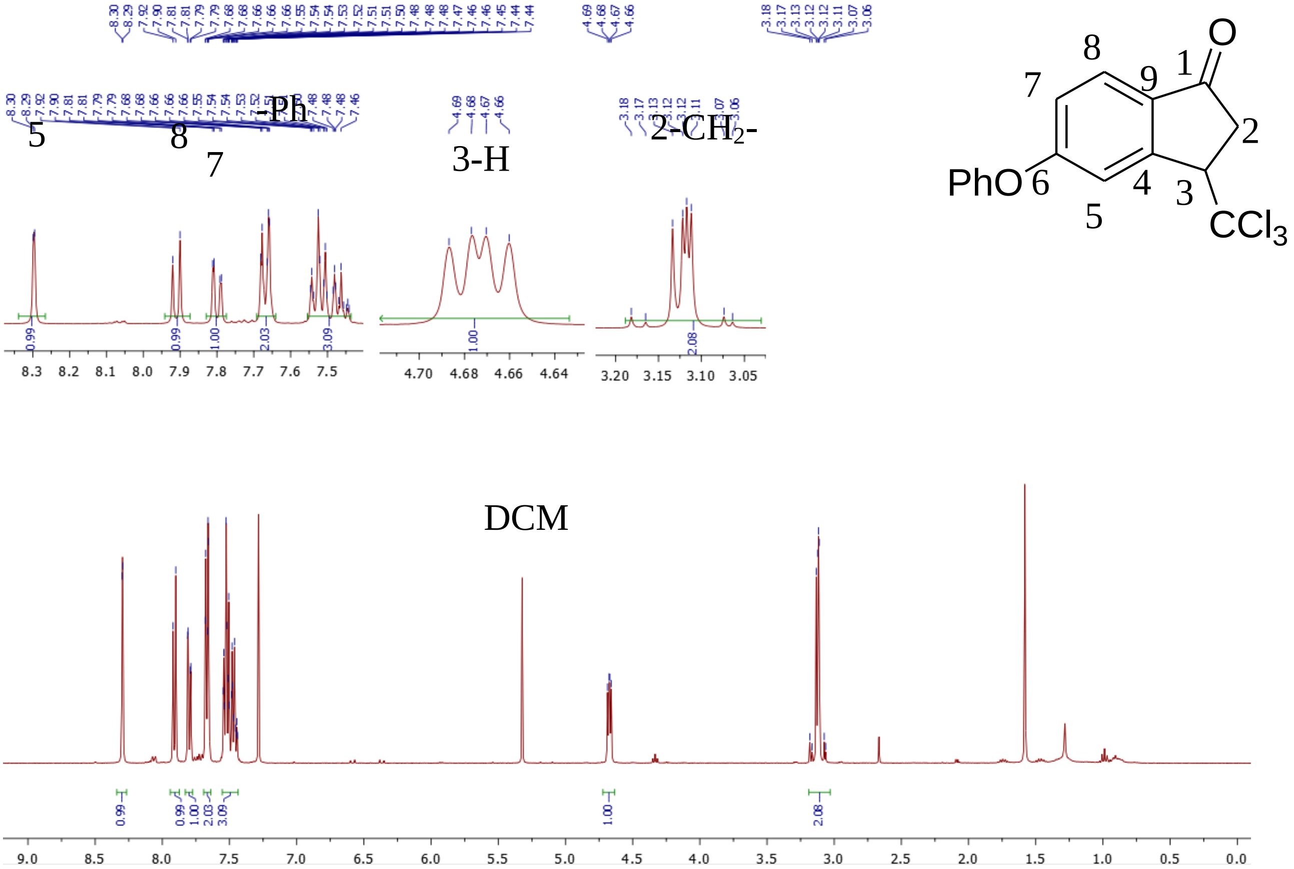

Figure S108. ^{1}H NMR spectrum of the compound **3j** ($CDCl_3$, 400 MHz).

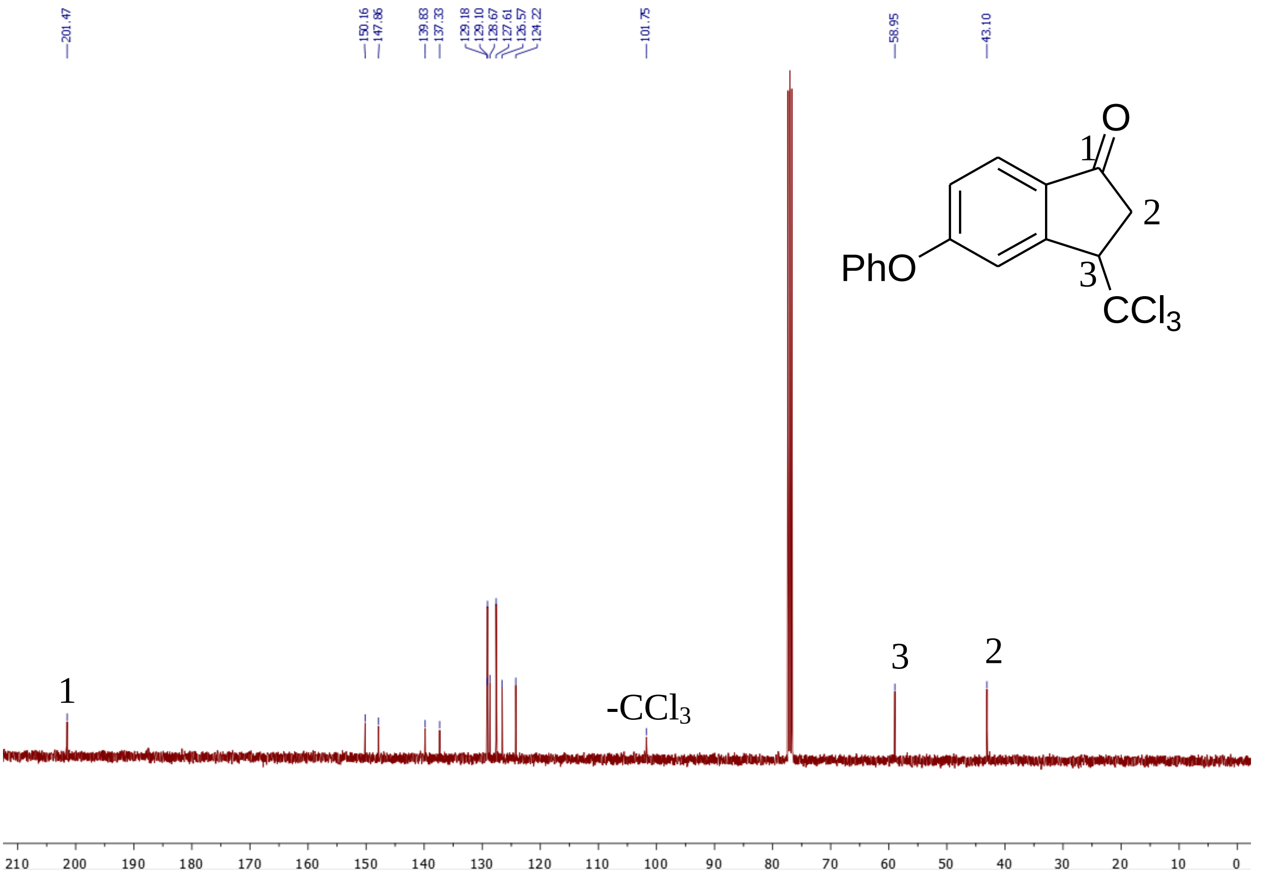

Figure S109. $^{13}C\{^1H\}$ NMR spectrum of the compound **3j** ($CDCl_3$, 101 MHz).

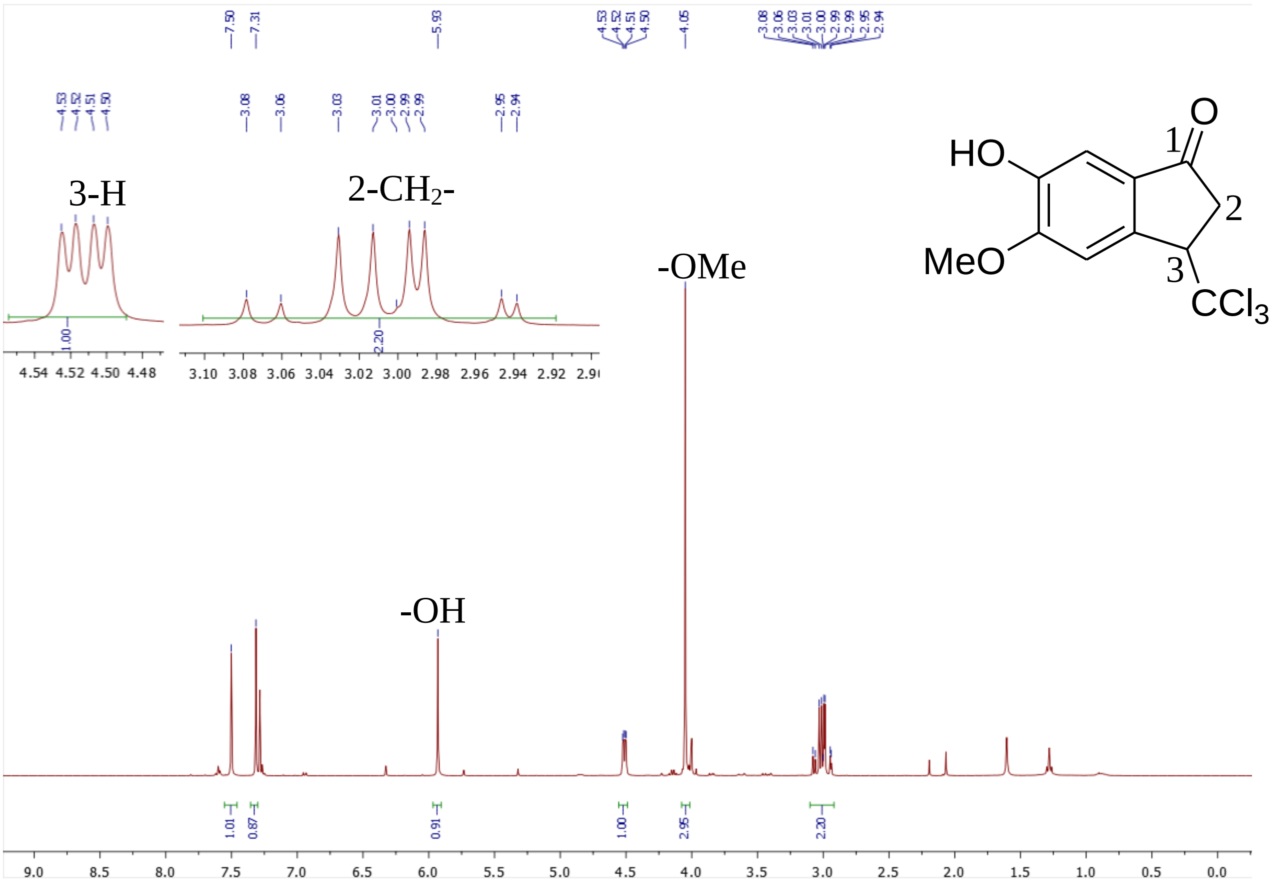

Figure S110. ^1H NMR spectrum of the compound **3k** ($CDCl_3$, 400 MHz).

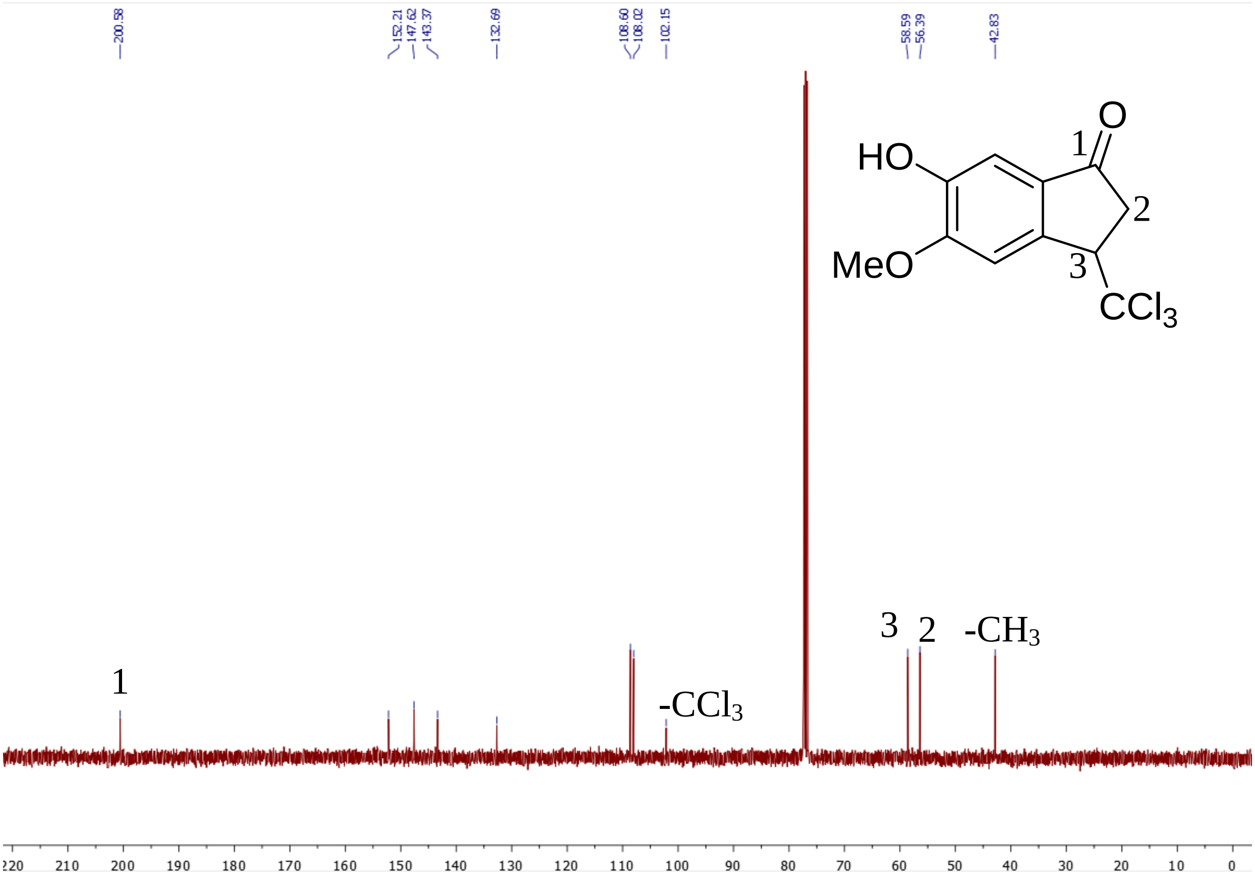

Figure S111. $^{13}C\{H\}$ NMR spectrum of compound **3k** (101 MHz, $CDCl_3$).

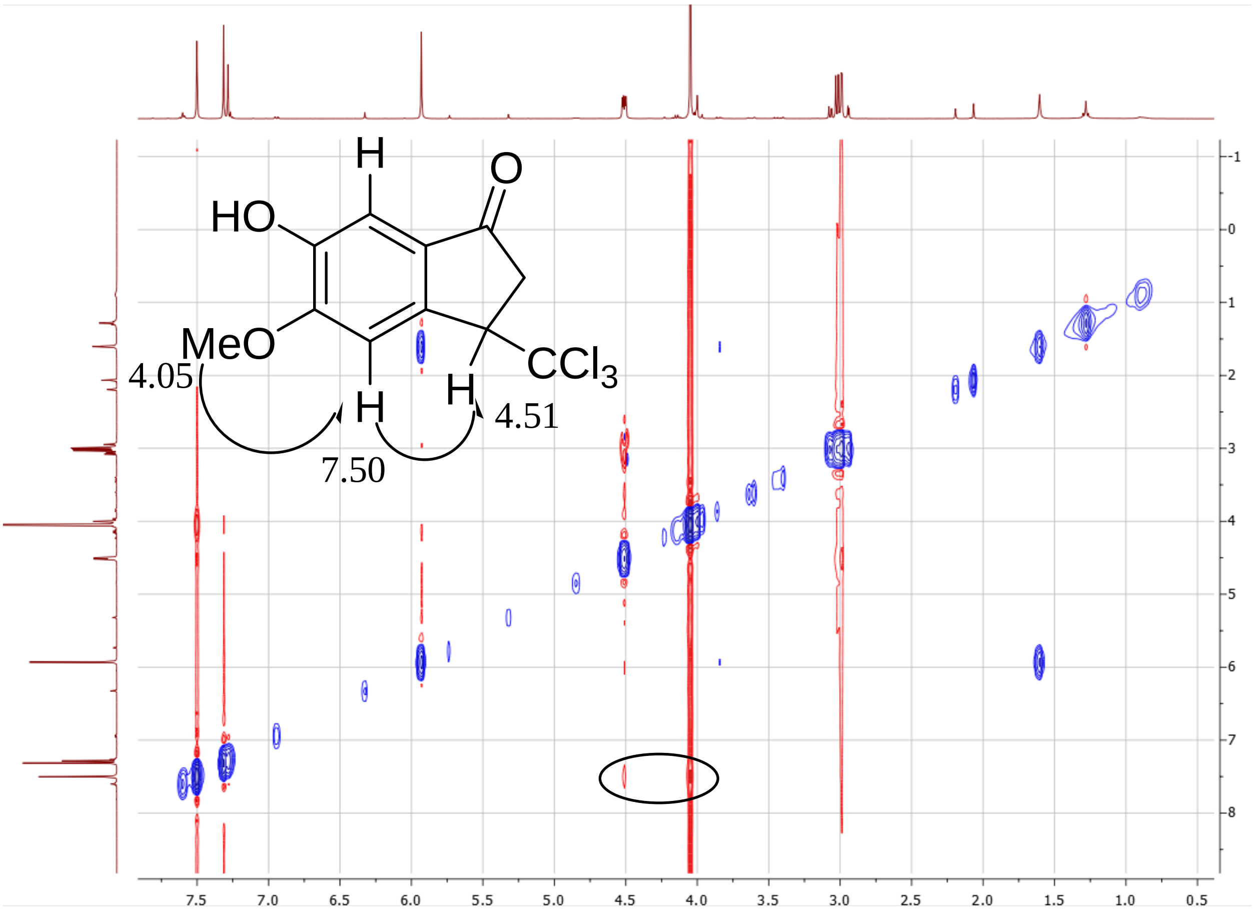

Figure S112. ^1H,^1H NOESY NMR spectrum of compound **3k** (400 MHz, $CDCl_3$).

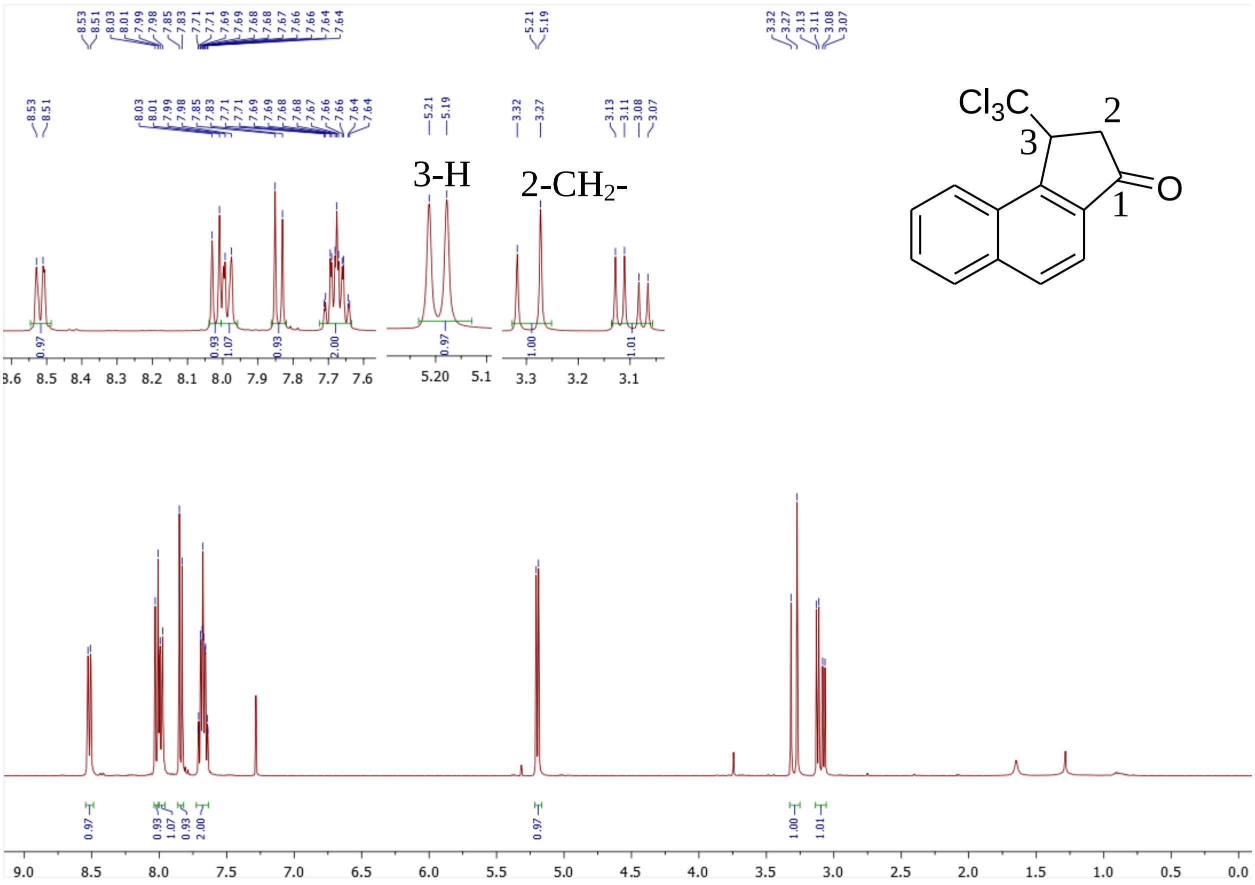

Figure S113. ^{1}H NMR spectrum of the compound **3n** ($CDCl_3$, 400 MHz).

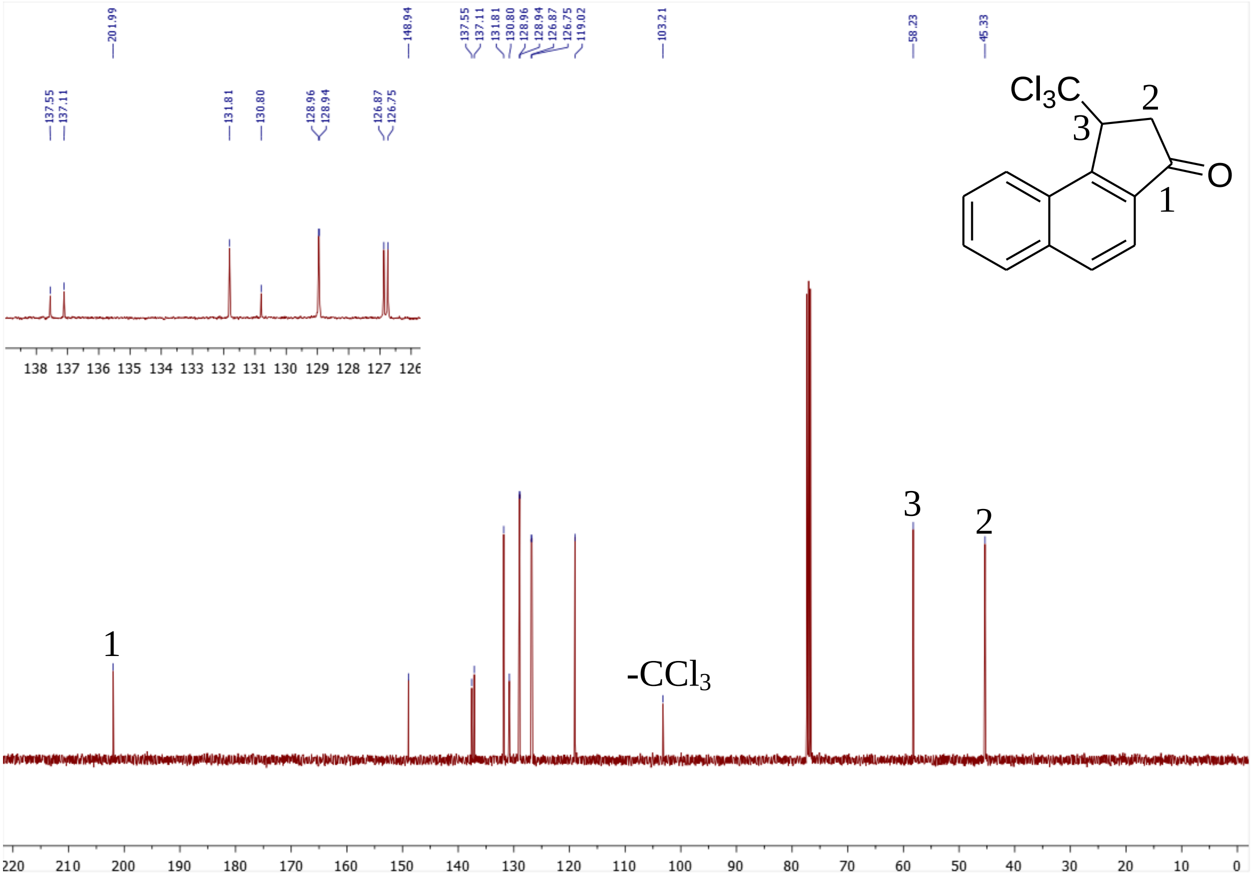

Figure S114. $^{13}C\{H\}$ NMR spectrum of compound **3n** (101 MHz, $CDCl_3$).

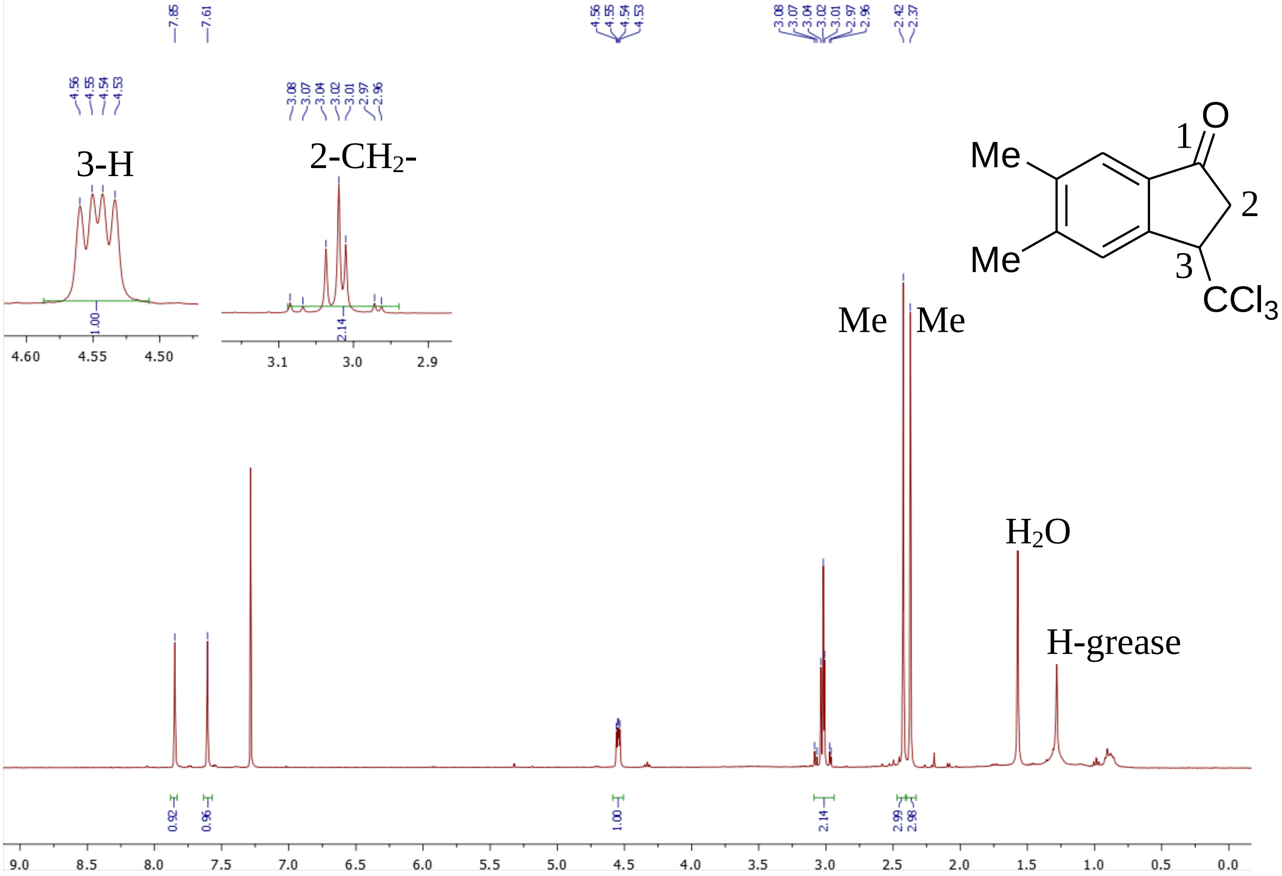

Figure S115. ^{1}H NMR spectrum of the compound **3p** ($CDCl_3$, 400 MHz).

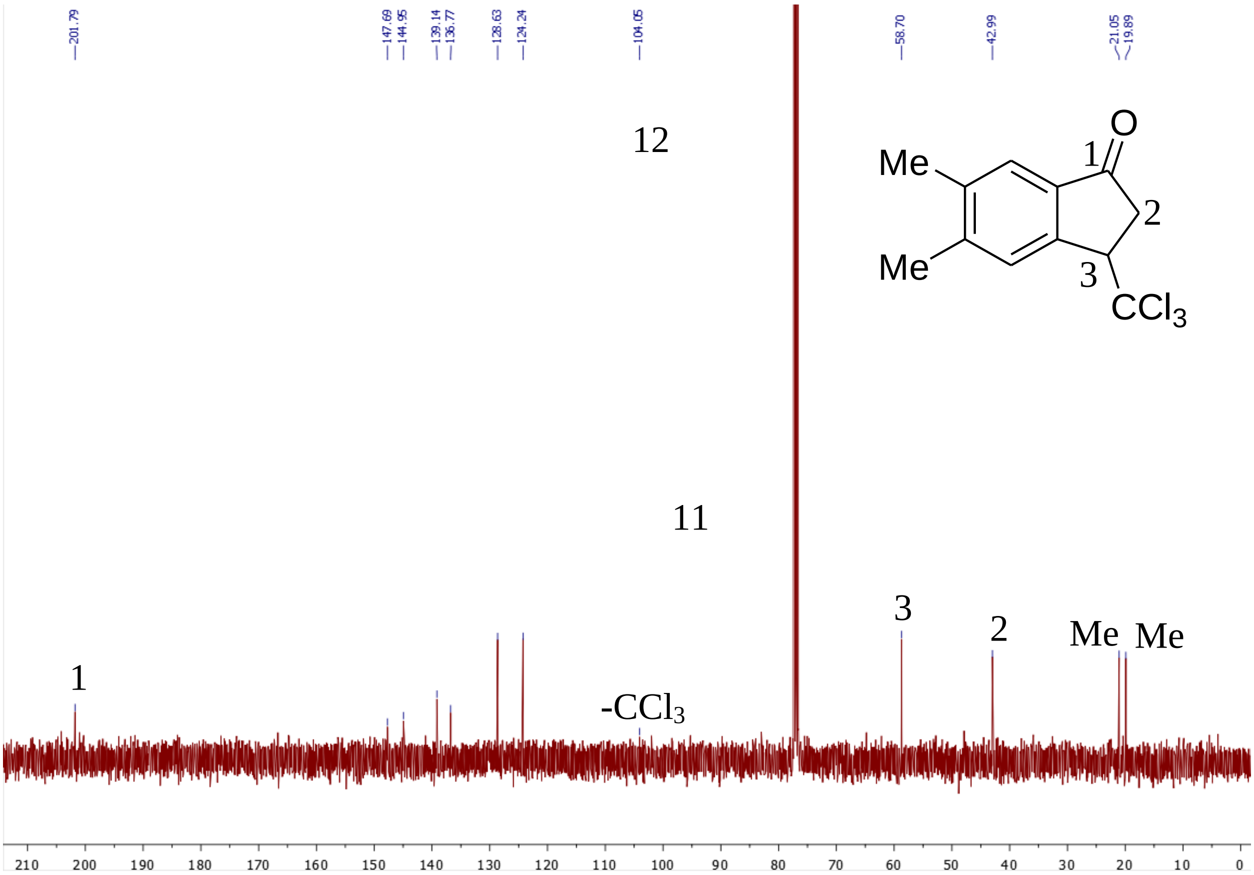

Figure S116. $^{13}C\{^1H\}$ NMR spectrum of the compound **3p** ($CDCl_3$, 101 MHz).

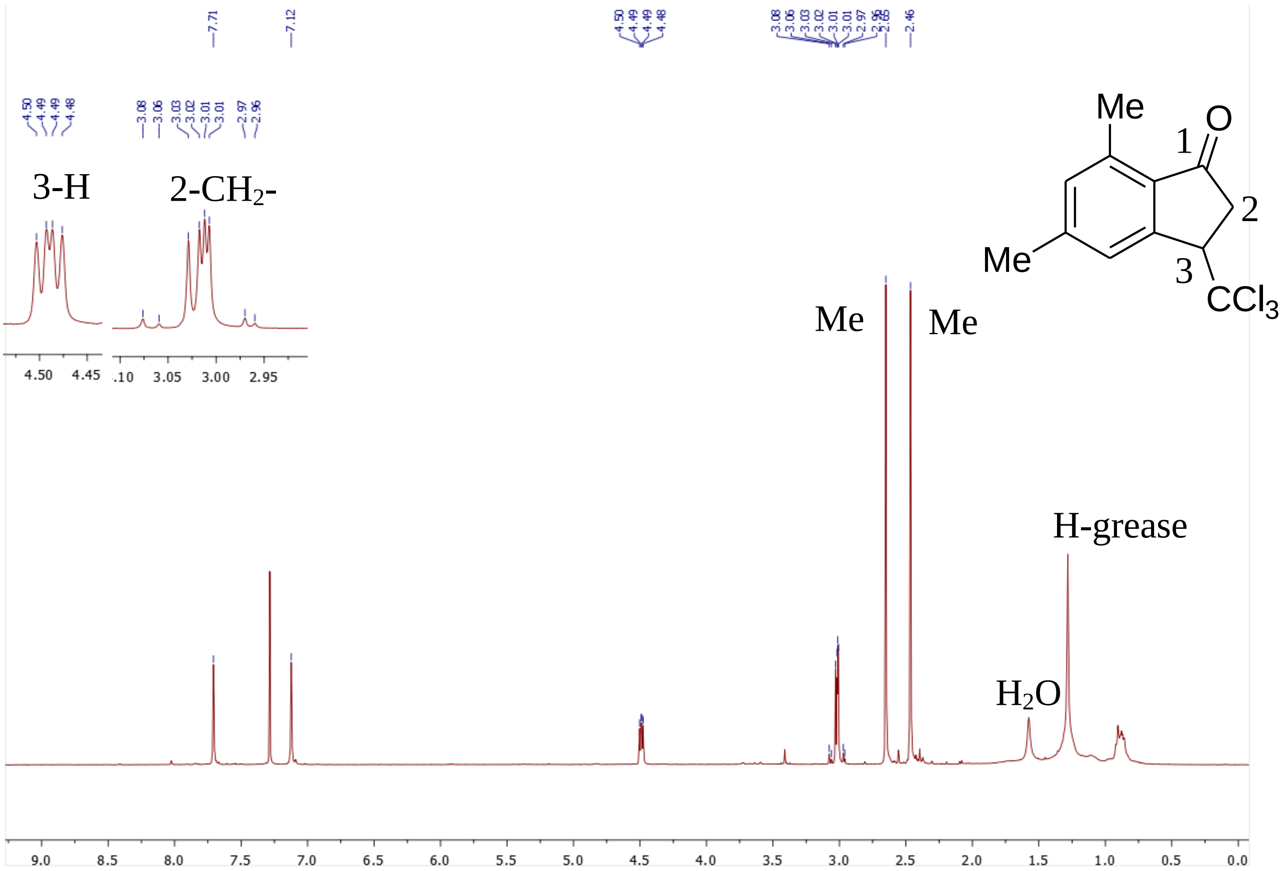

Figure S117. ^{1}H NMR spectrum of the compound **3q** ($CDCl_3$, 400 MHz).

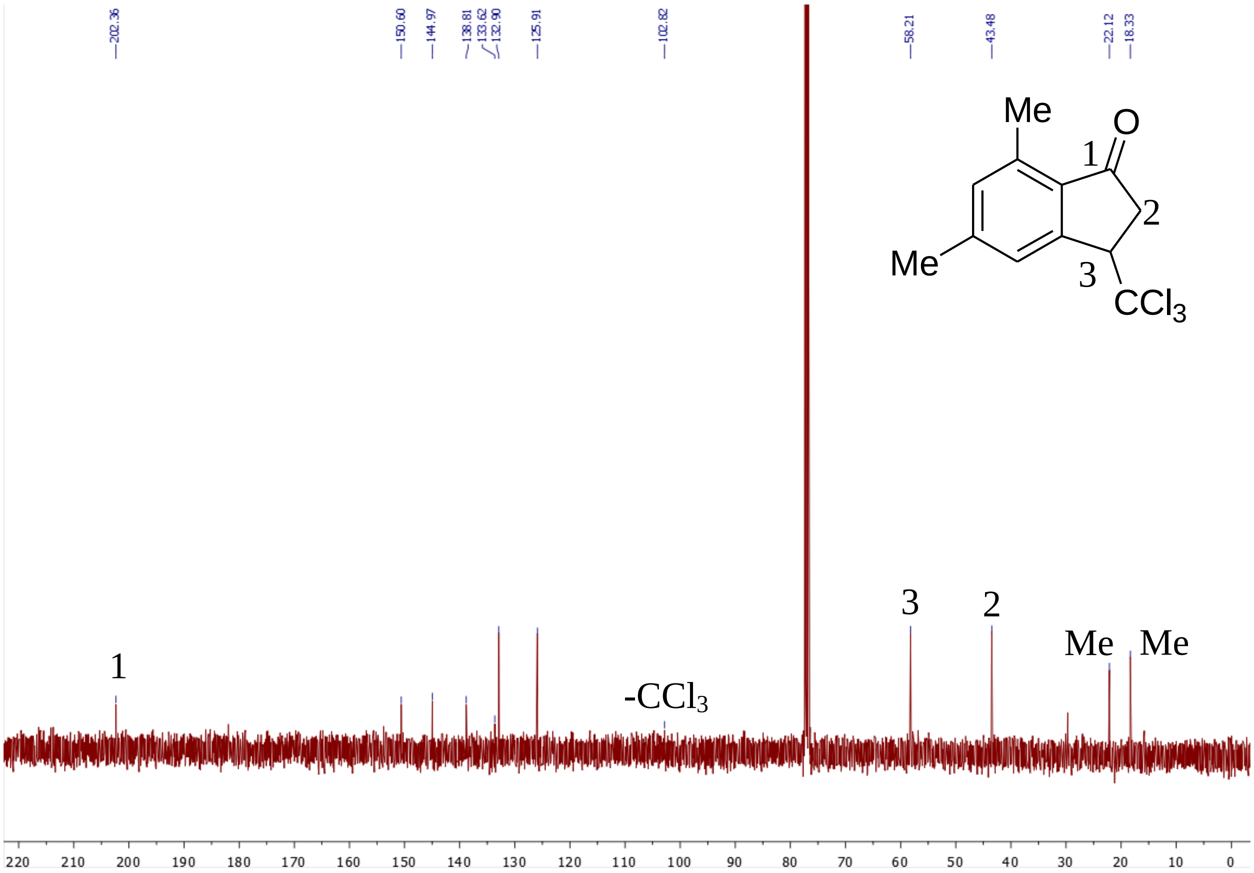

Figure S118. $^{13}C\{^{1}H\}$ NMR spectrum of the compound **3q** ($CDCl_3$, 101 MHz).

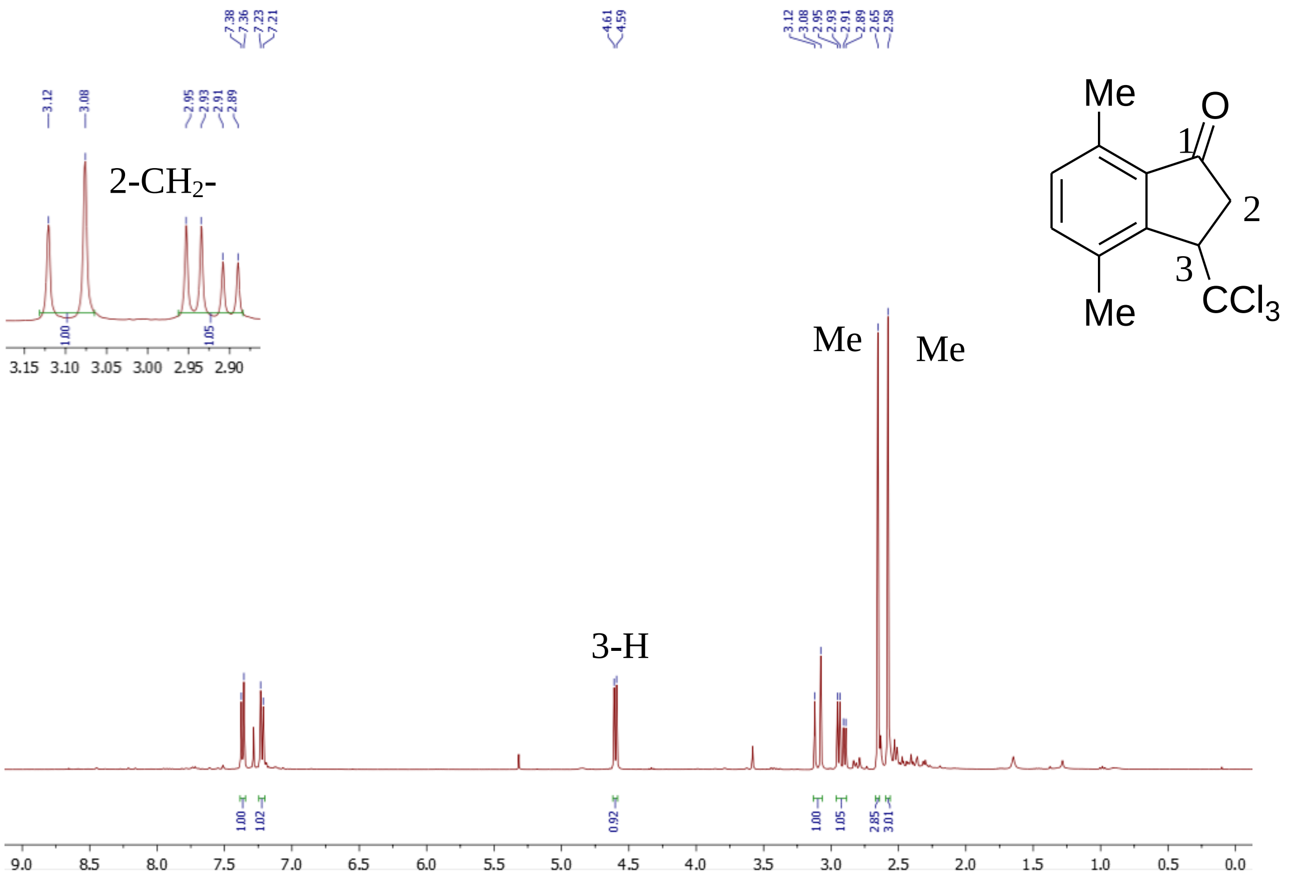

Figure S119. ^{1}H NMR spectrum of the compound **3r** ($CDCl_3$, 400 MHz).

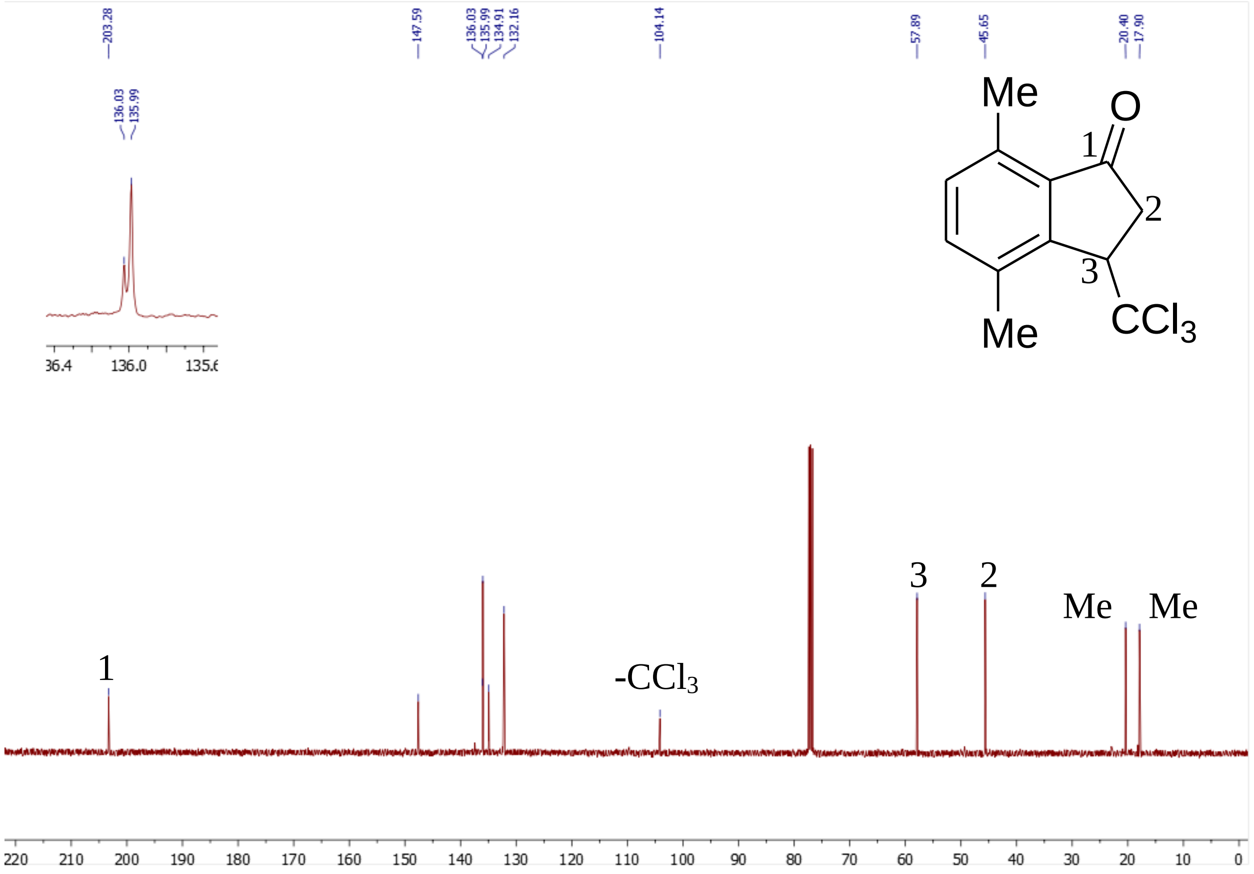

Figure S120. $^{13}C\{^{1}H\}$ NMR spectrum of the compound **3r** ($CDCl_3$, 101 MHz).

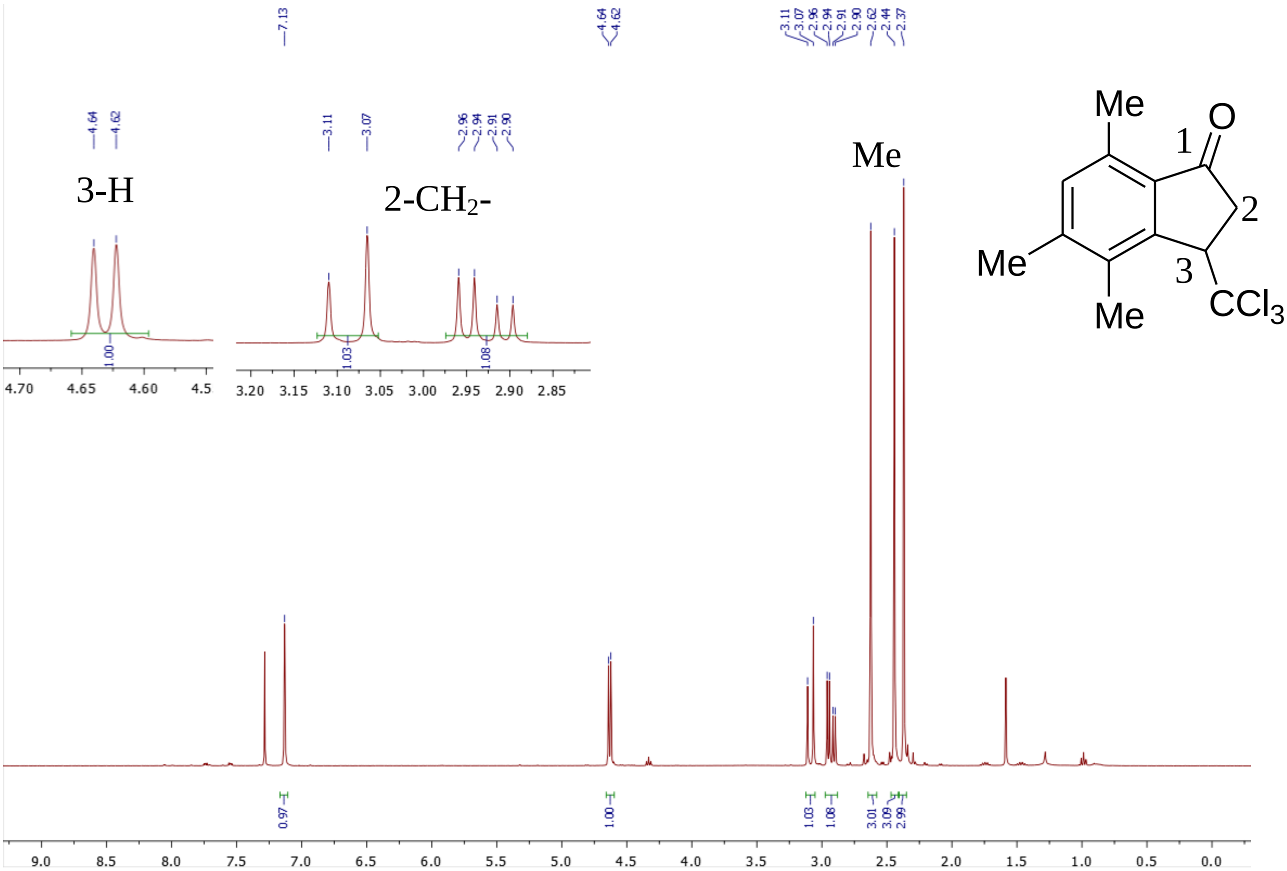

Figure S121. ^{1}H NMR spectrum of the compound **3s** ($CDCl_3$, 400 MHz).

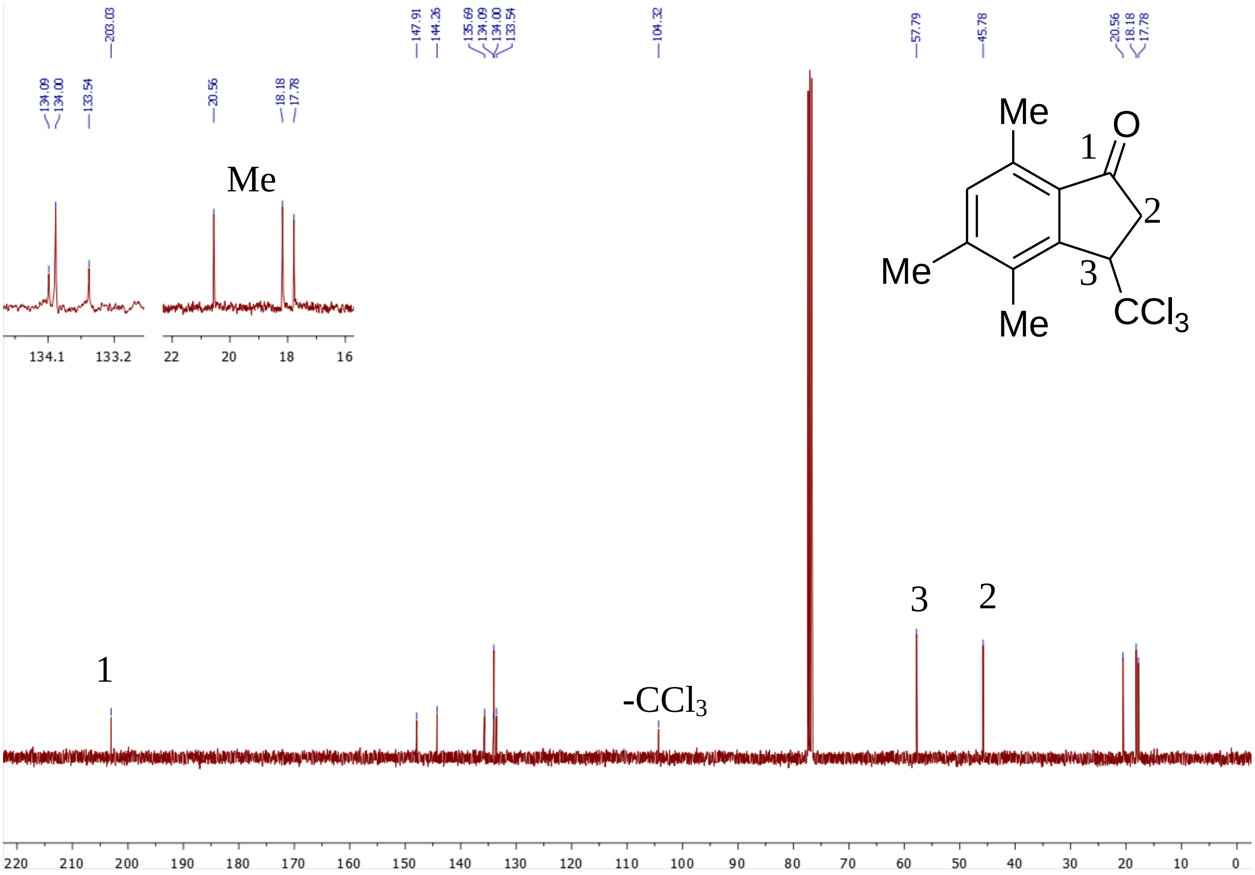

Figure S122. ^{13}C{^{1}H} NMR spectrum of the compound **3s** ($CDCl_3$, 101 MHz).

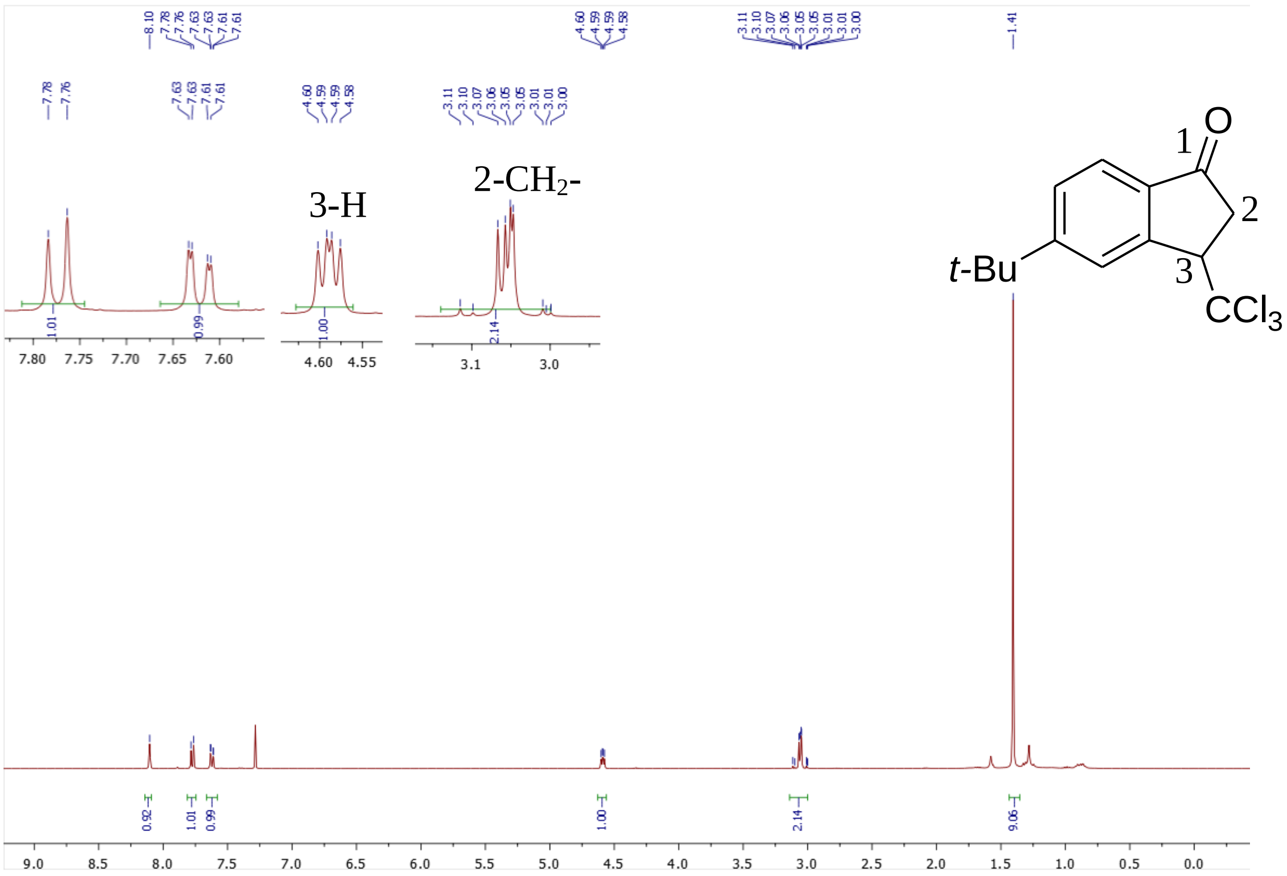

Figure S123. ^{1}H NMR spectrum of the compound **3v**($CDCl_3$, 400 MHz).

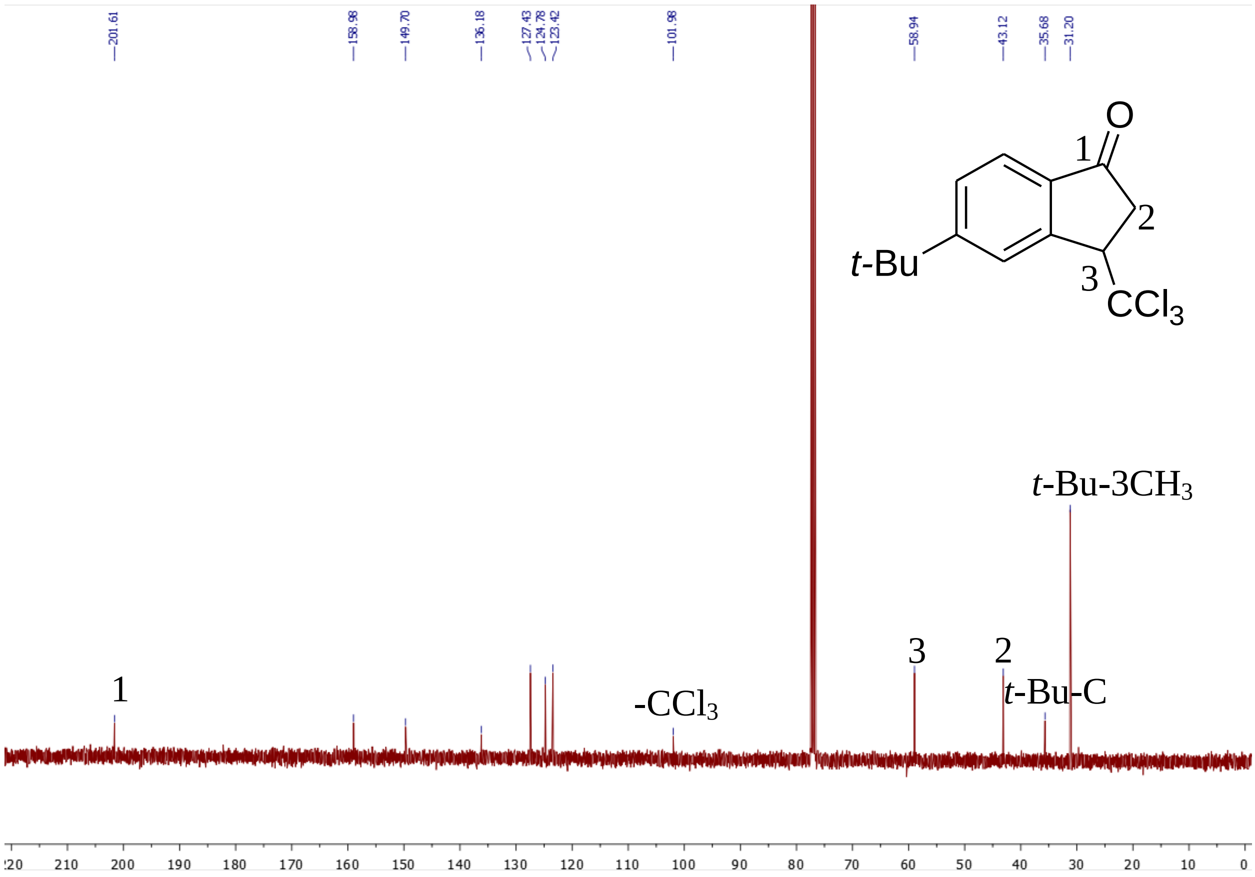

Figure S124. $^{13}C\{^{1}H\}$ NMR spectrum of the compound **3v** ($CDCl_3$, 101 MHz).

4. ^{1}H, ^{13}C{^{1}H}, ^{19}F{^{1}H} NMR Spectra of cations Aa, Ac, Ad, Ba, Bc, Bd, Bm.

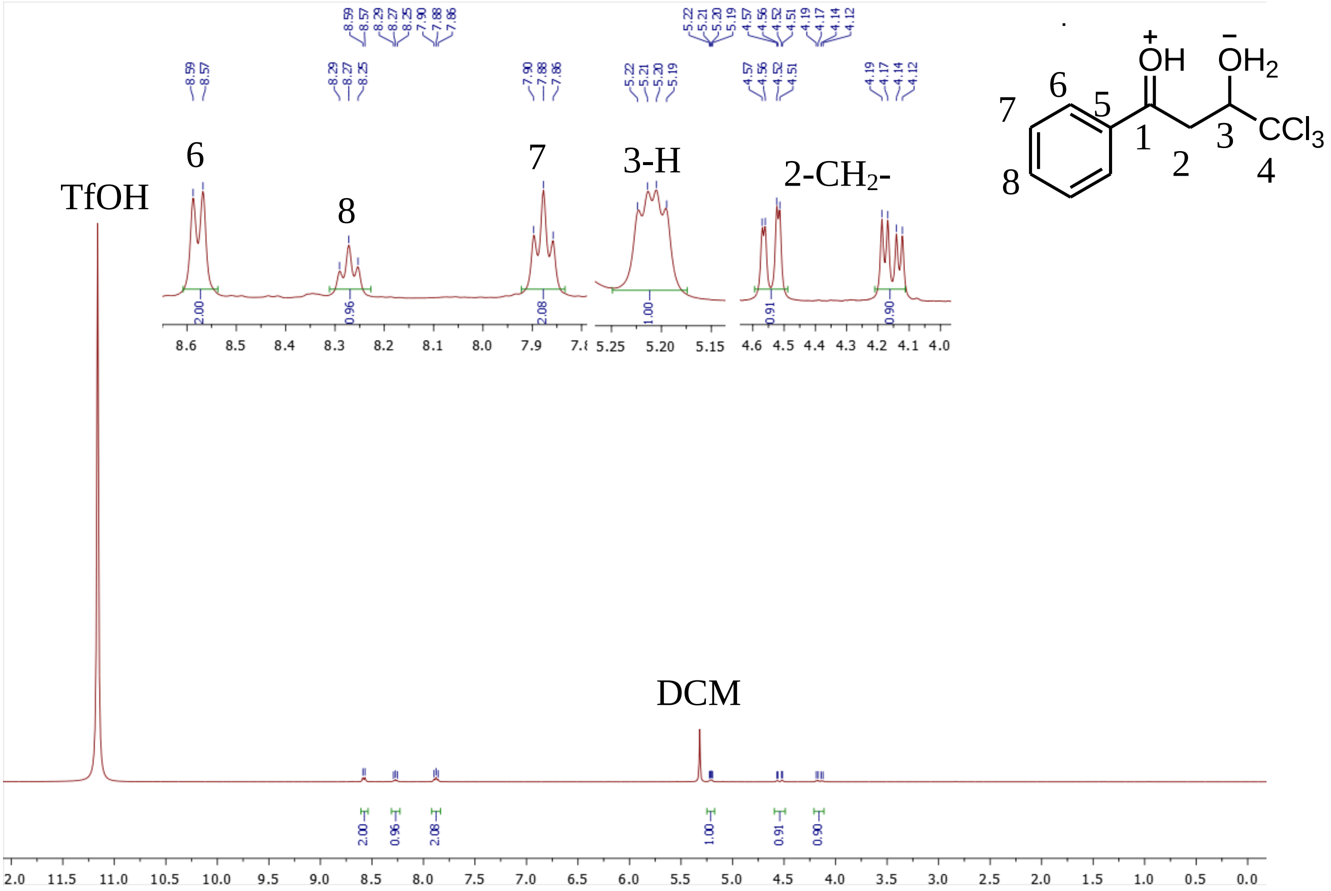

Figure S125. ^{1}H NMR spectrum of cation **Aa** (400 MHz, $CDCl_3$, DCM – internal standard).

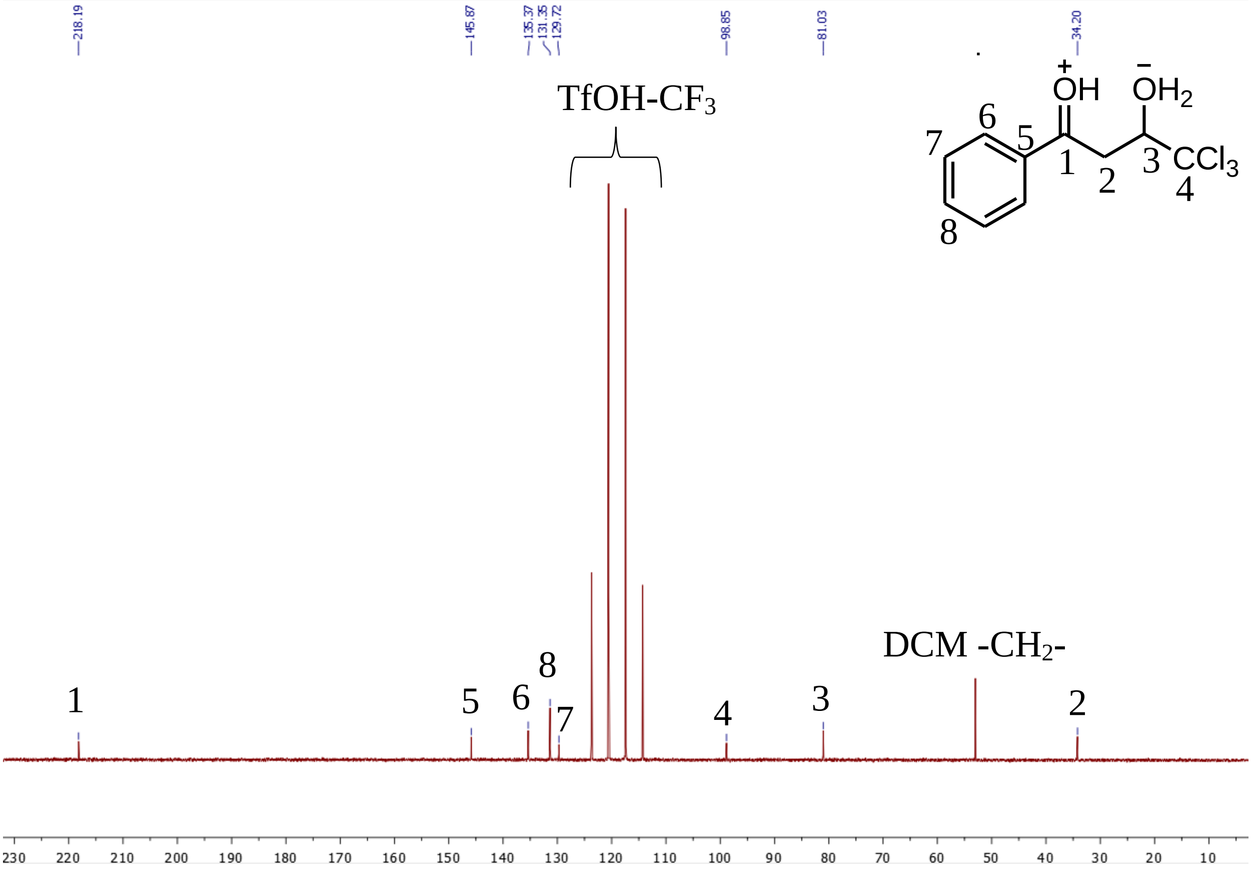

Figure S126. ^{13}C{H} NMR spectrum of cation **Aa** (101 MHz, $CDCl_3$, DCM – internal standard).

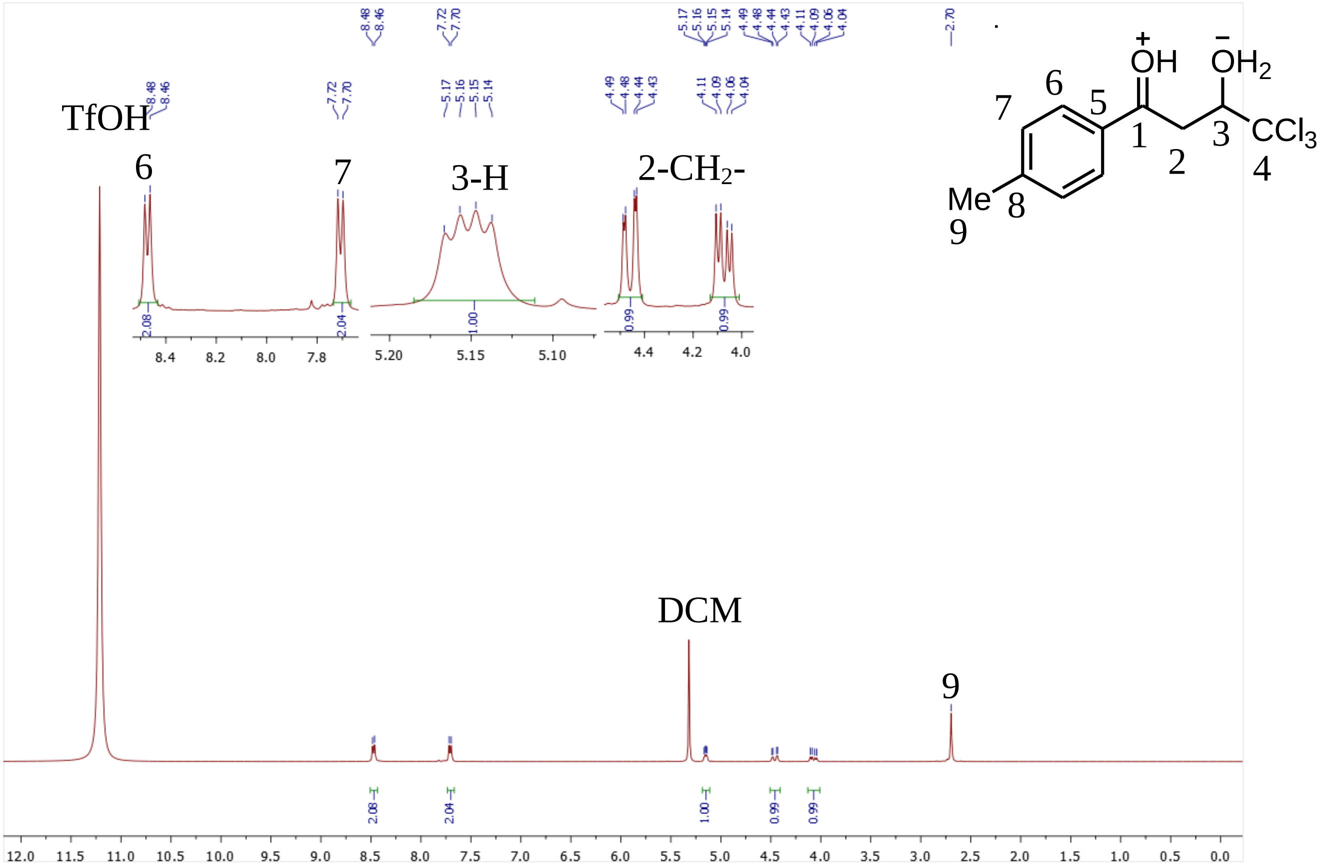

Figure S127. ^{1}H NMR spectrum of cation **Ac** (400 MHz, $CDCl_3$, DCM – internal standard).

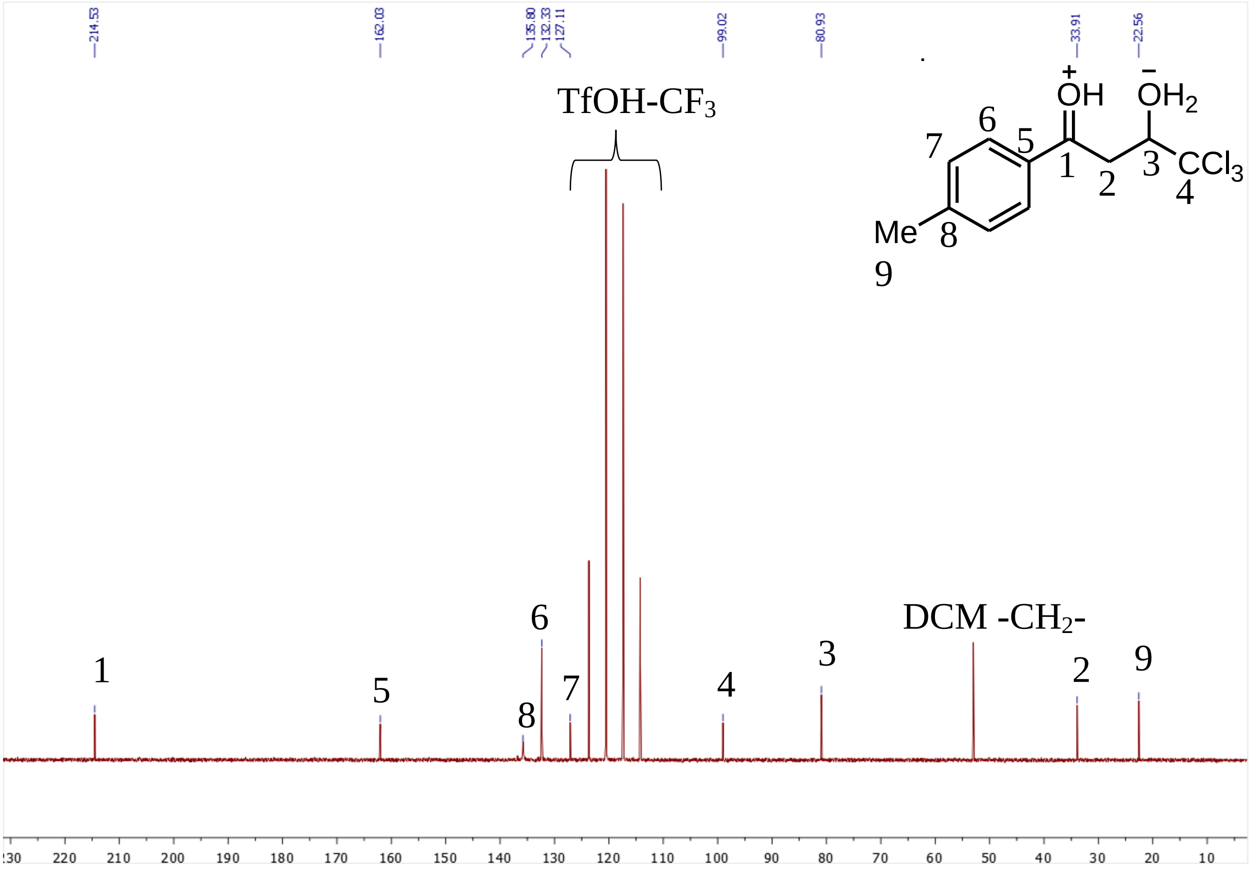

Figure S128. ^{13}C{H} NMR spectrum of cation **Ac** (101 MHz, $CDCl_3$, DCM – internal standard).

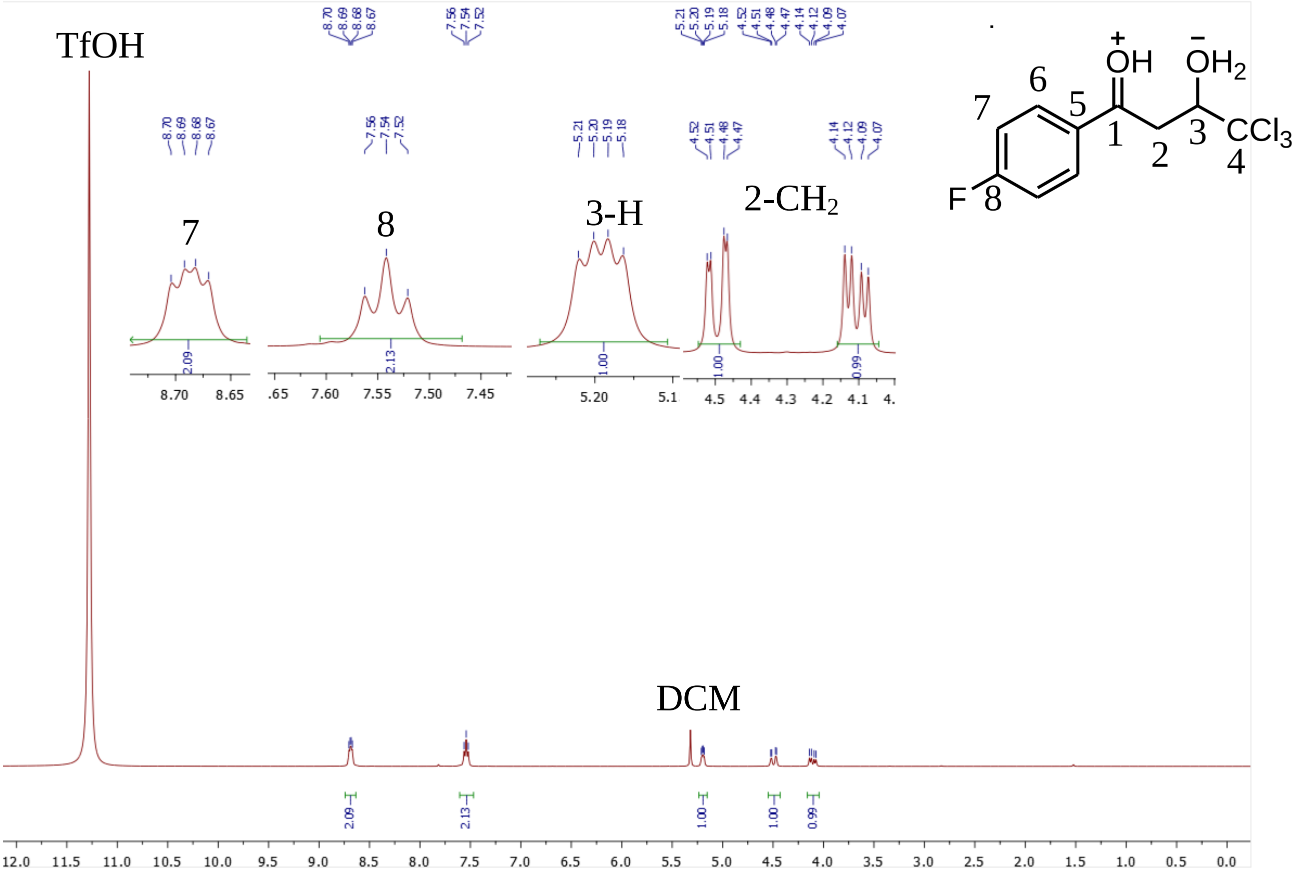

Figure S129. ^{1}H NMR spectrum of cation **Ad** (400 MHz, $CDCl_3$, DCM – internal standard).

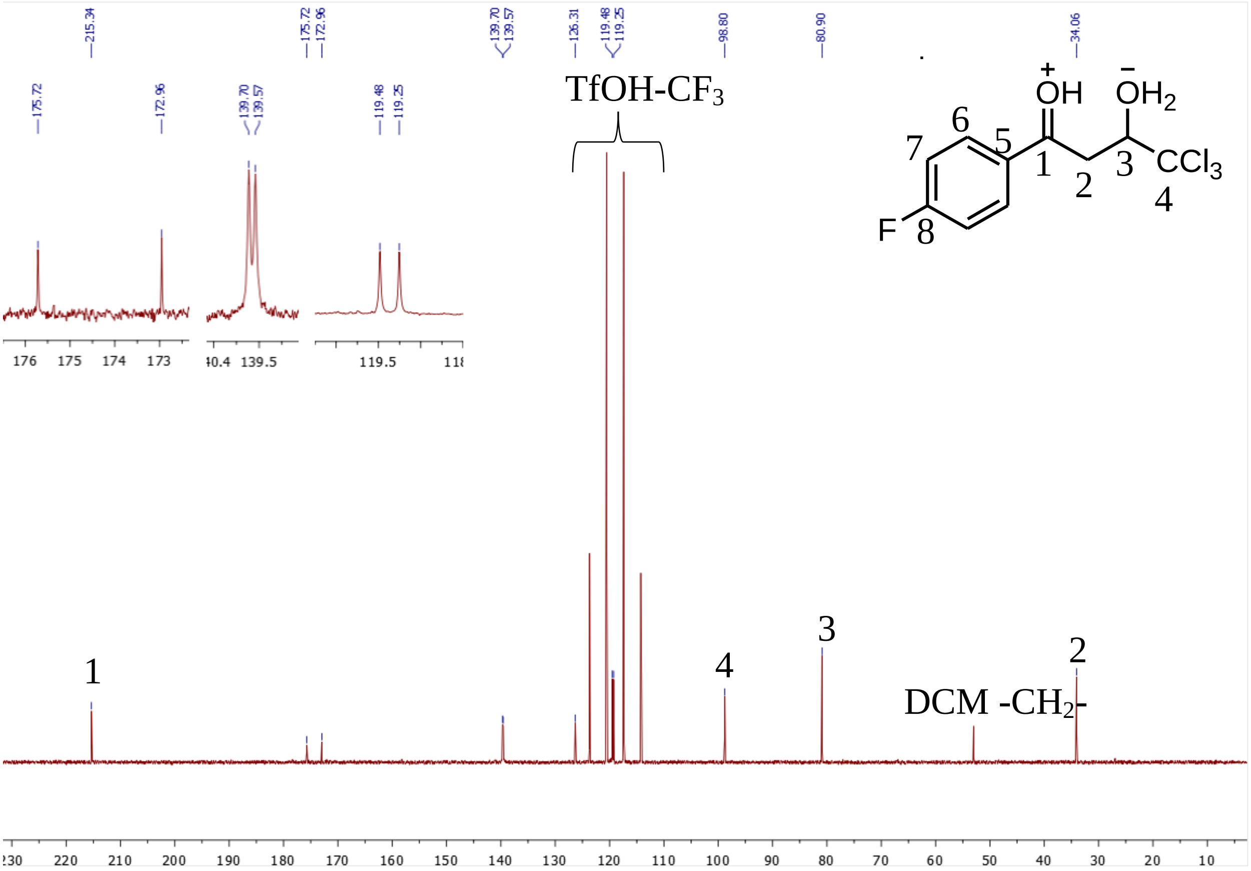

Figure S130. ^{13}C{H} NMR spectrum of c cation **Ad** (101 MHz, $CDCl_3$, DCM – internal standard).

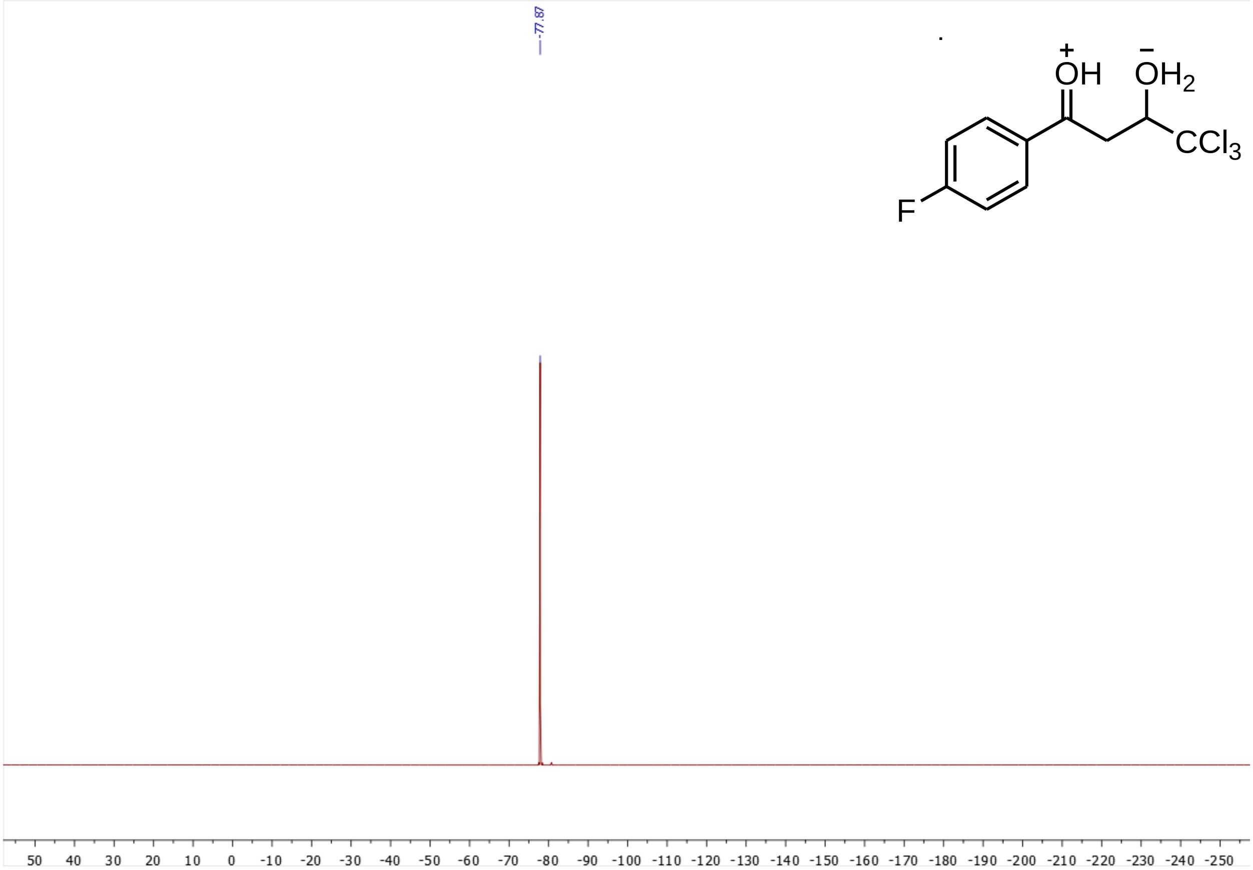

Figure S131. ^{19}F NMR spectrum of cation **Ad** ($CDCl_3$, 376 MHz, DCM – internal standard).

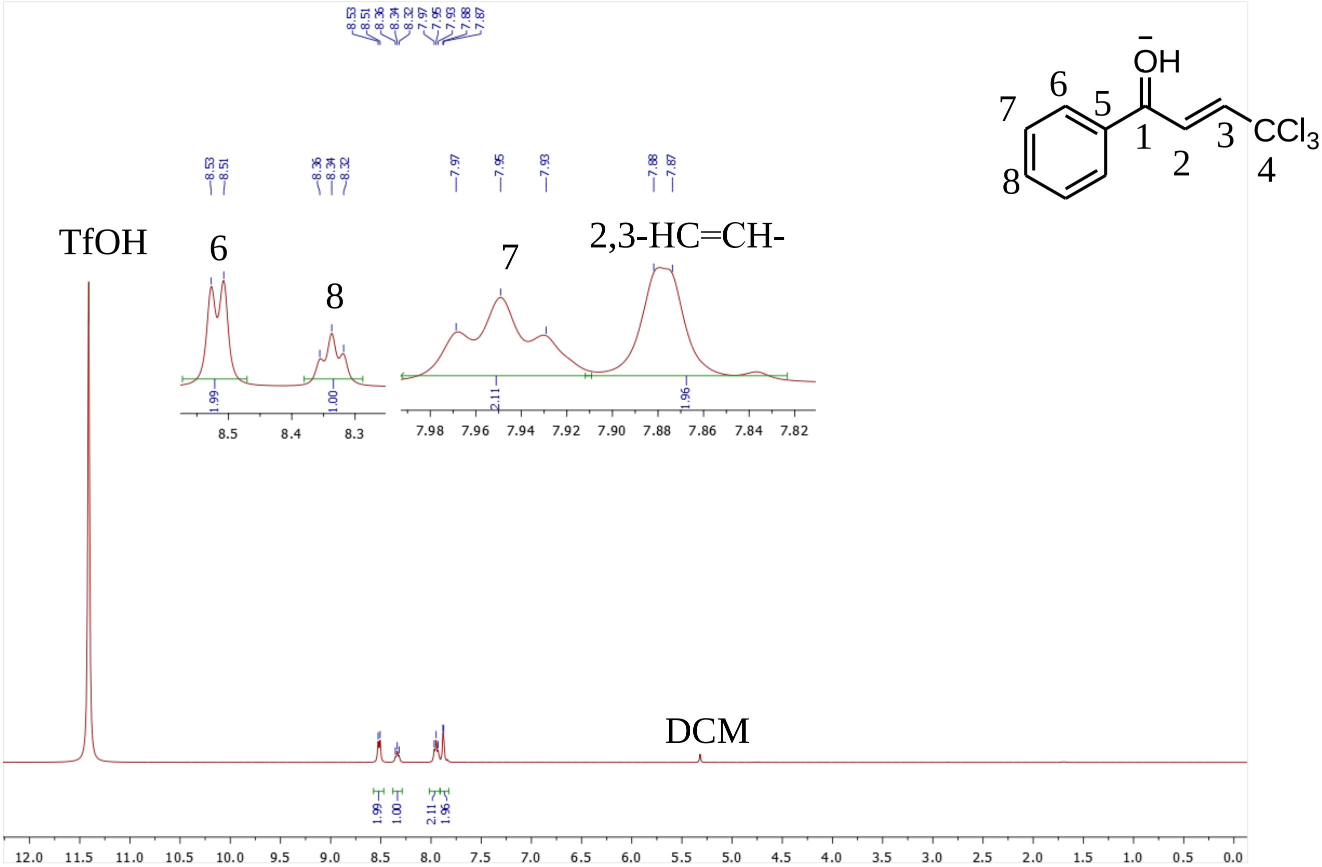

Figure S132. ^{1}H NMR spectrum of cation **Ba** (400 MHz, $CDCl_3$, DCM – internal standard).

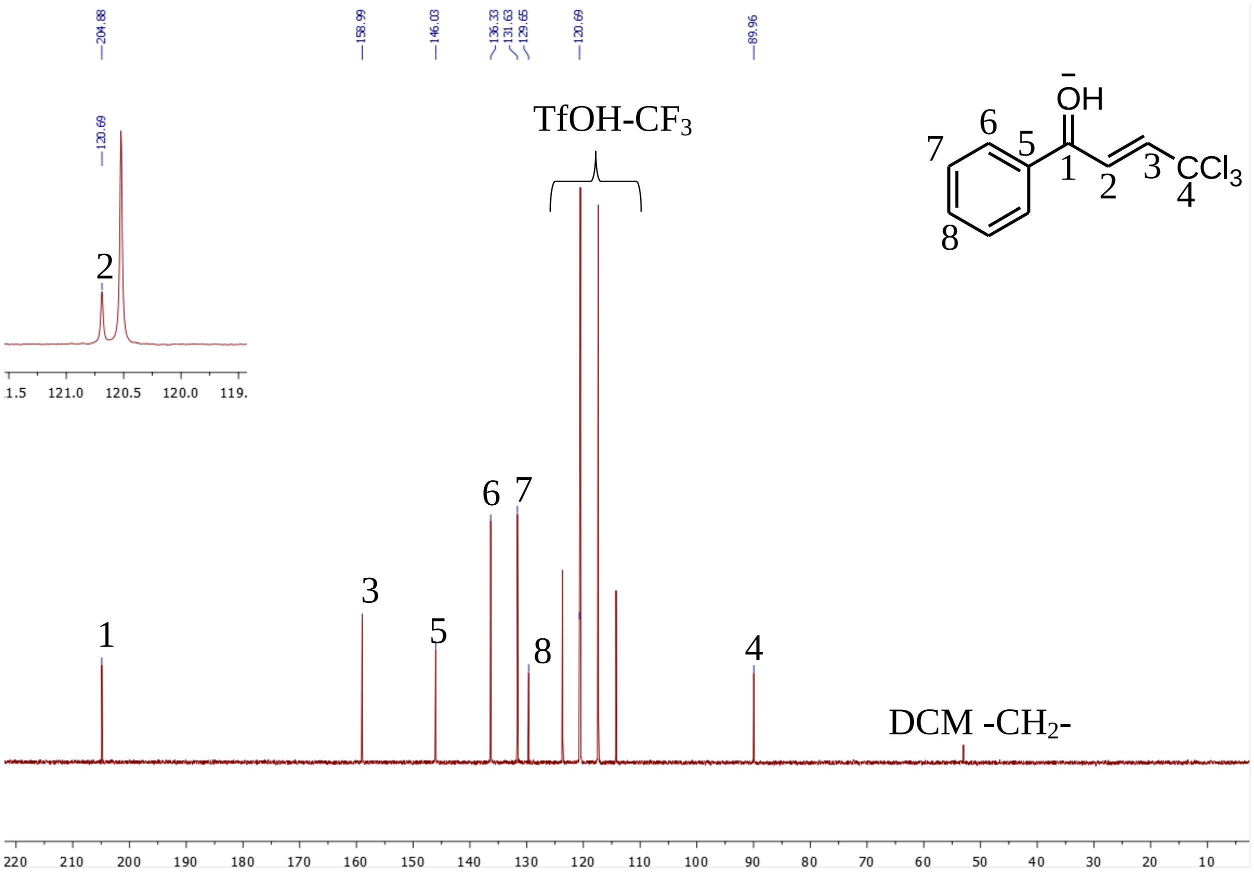

Figure S133. $^{13}C\{H\}$ NMR spectrum of cation **Ba** (101 MHz, $CDCl_3$, DCM – internal standard).

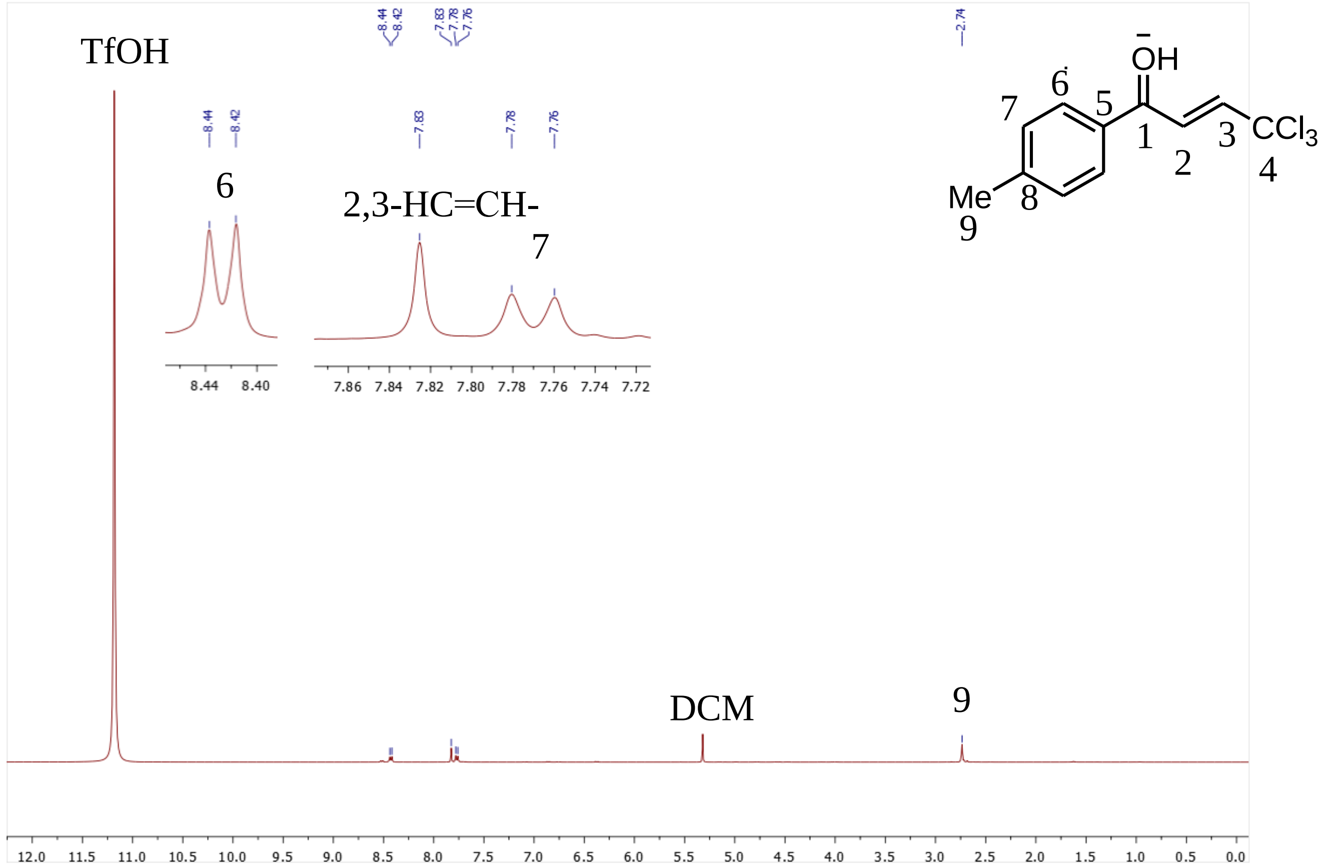

Figure S134. ^{1}H NMR spectrum of cation **Bc** (400 MHz, $CDCl_3$, DCM – internal standard).

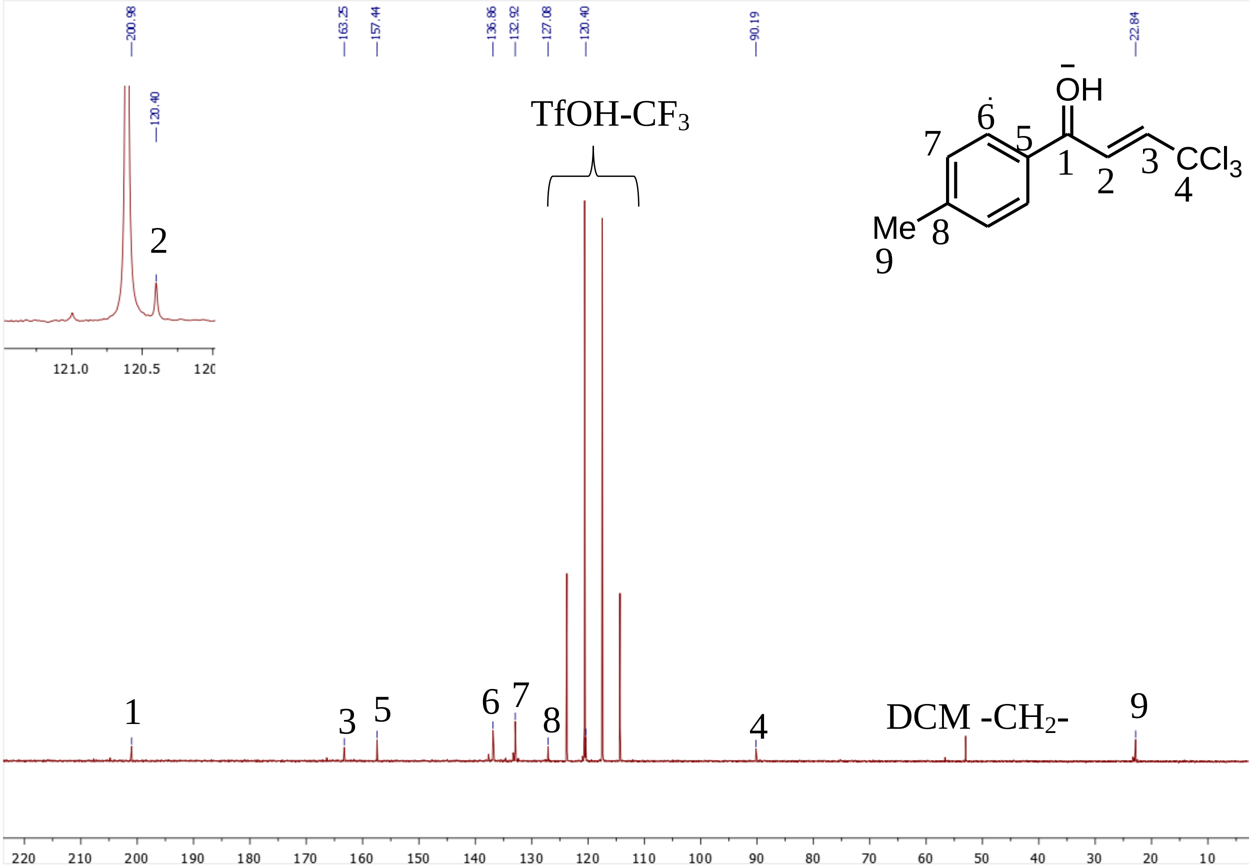

Figure S135. ^{13}C{H} NMR spectrum of cation **Bc** (101 MHz, $CDCl_3$, DCM – internal standard).

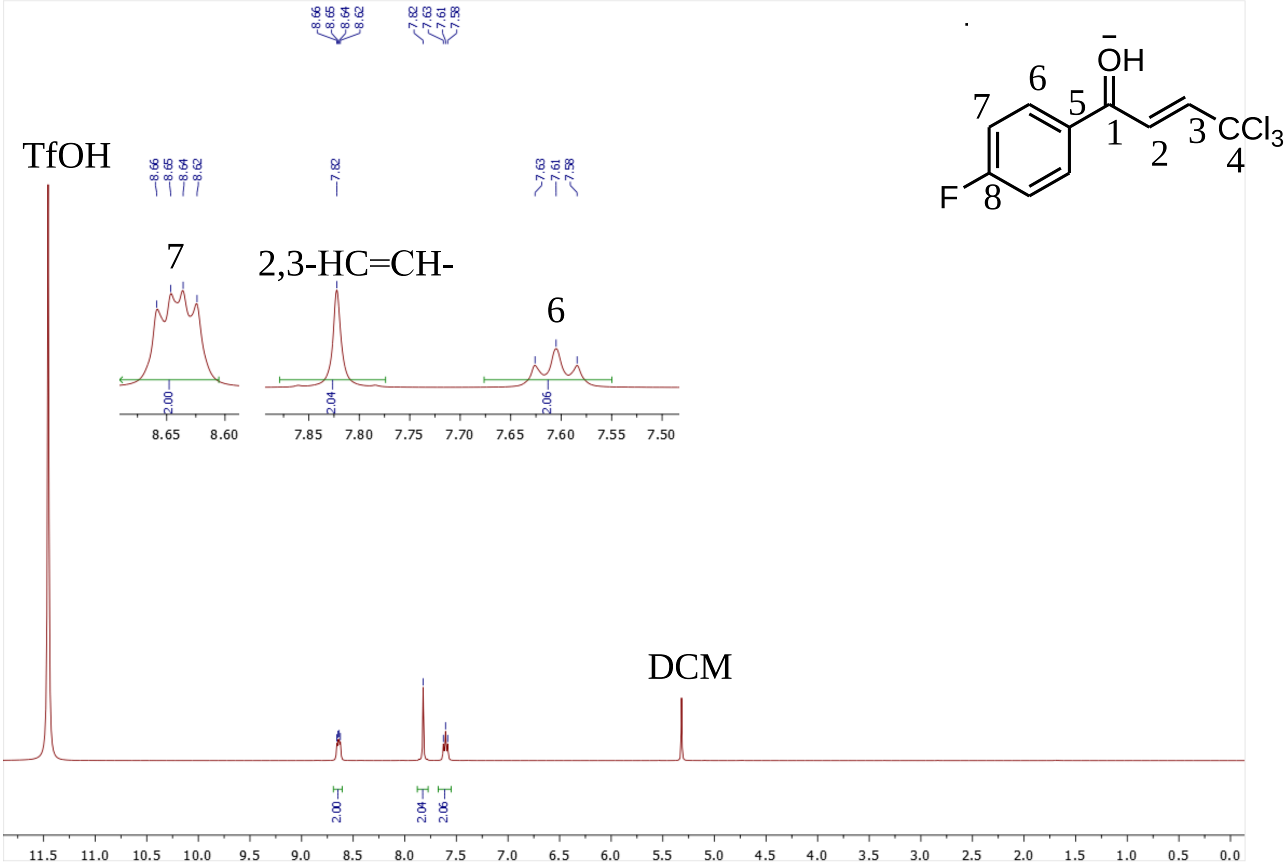

Figure S136. ^{1}H NMR spectrum of cation **Bd** (400 MHz, $CDCl_3$, DCM – internal standard).

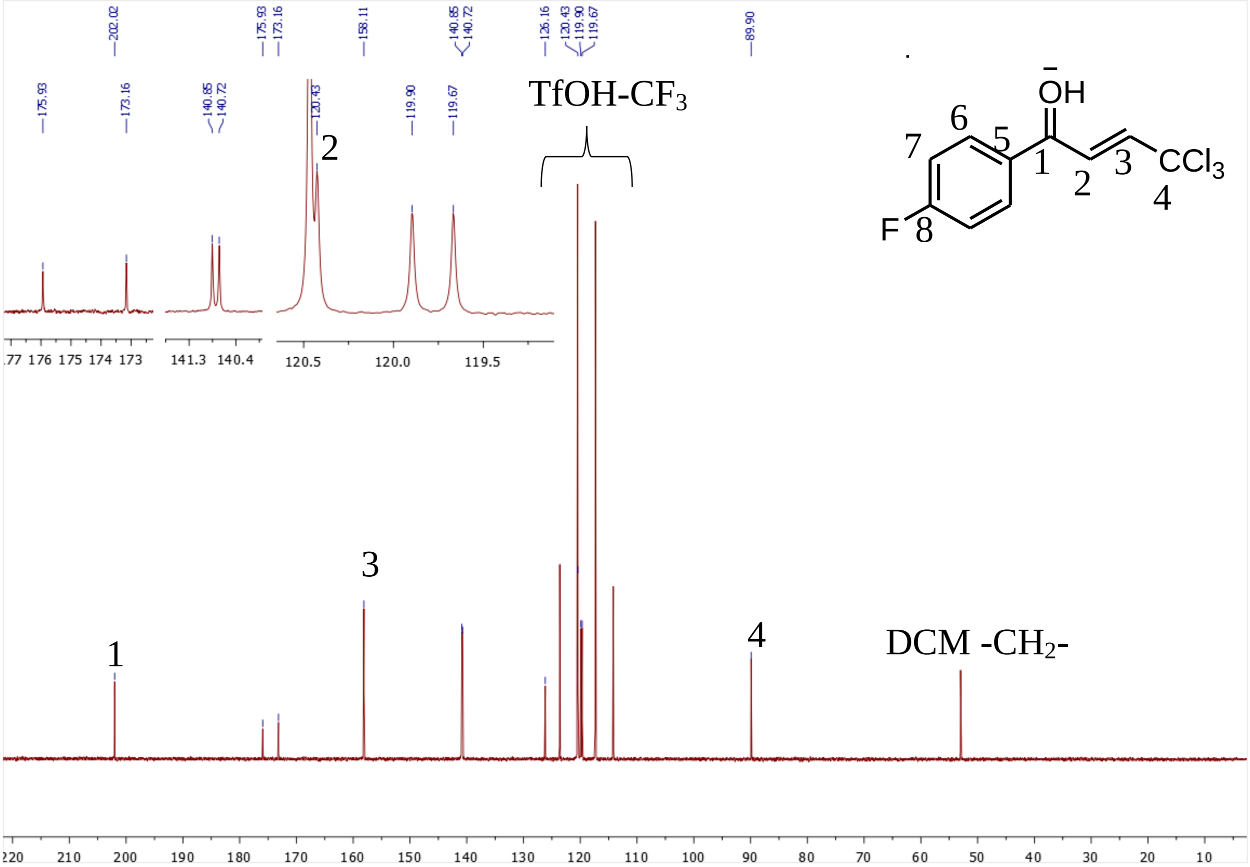

Figure S137. $^{13}C\{H\}$ NMR spectrum of cation **Bd** (101 MHz, $CDCl_3$, DCM – internal standard).

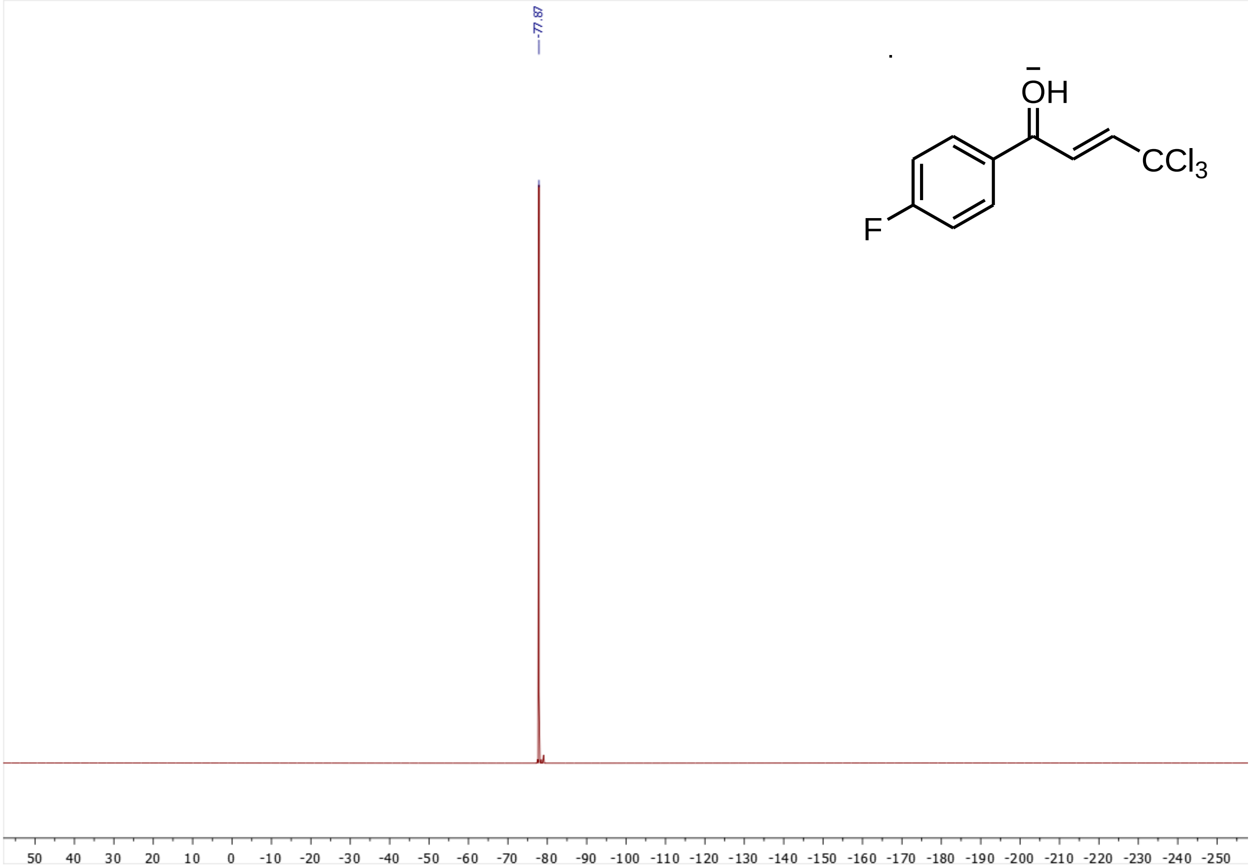

Figure S138. ^{19}F NMR spectrum of cation **Bd** ($CDCl_3$, 376 MHz, DCM – internal standard).

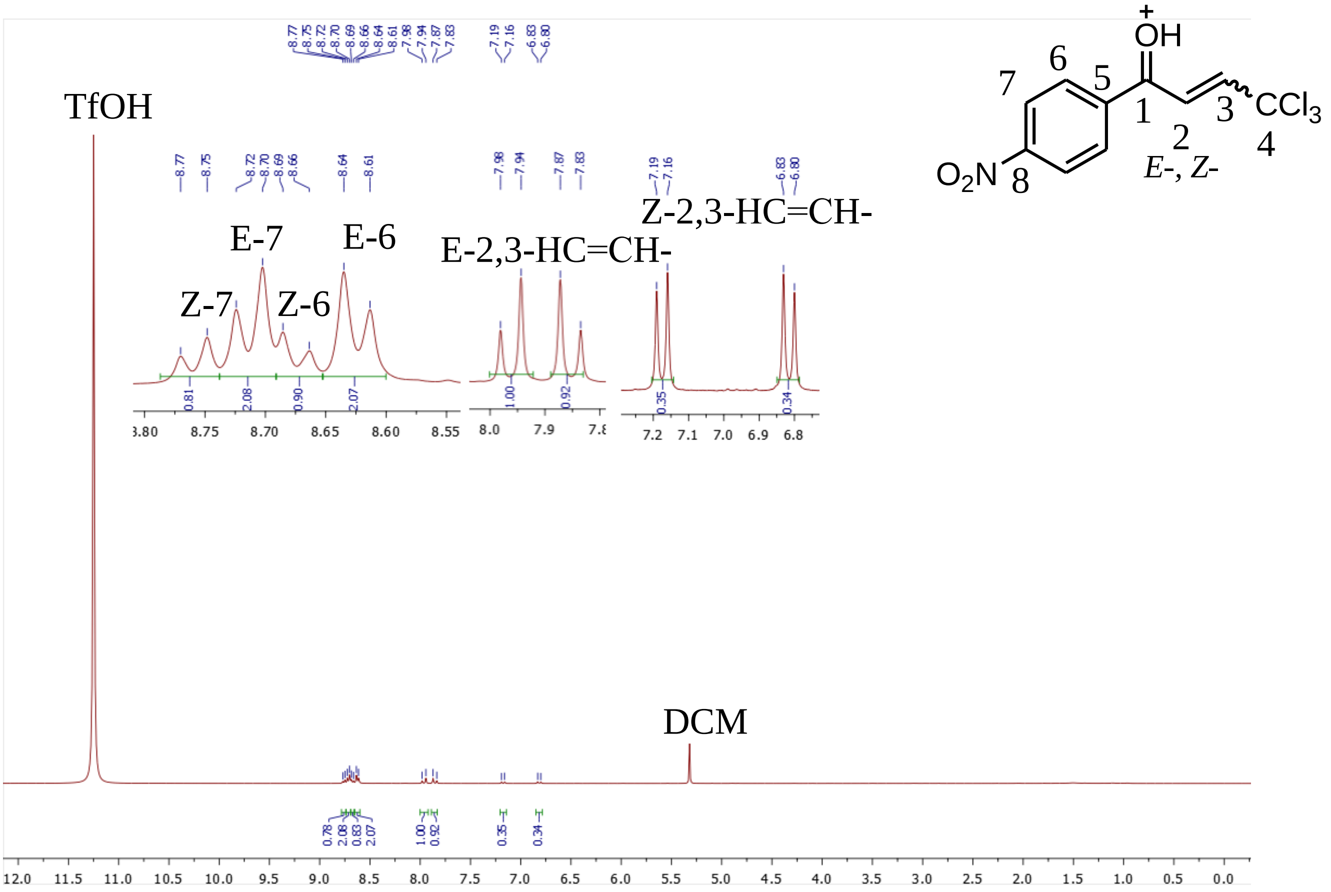

Figure S139. ^{1}H NMR spectrum of cation **Bm** (400 MHz, $CDCl_3$, DCM – internal standard).

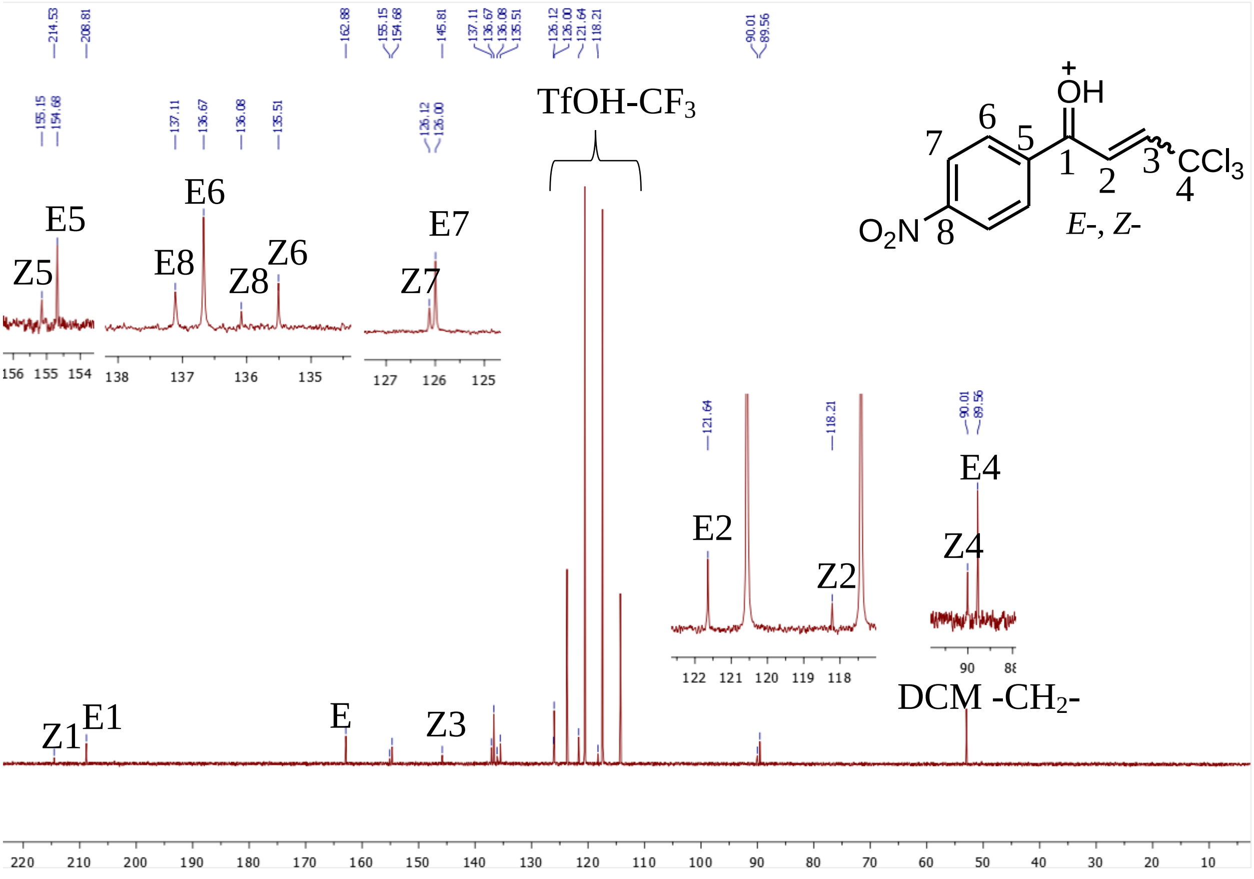

Figure S140. $^{13}C\{H\}$ NMR spectrum of cation **Bm** (101 MHz, $CDCl_3$, DCM – internal standard).

5. X-ray data of compounds 1g,h,s,t,v, and 3a

1g

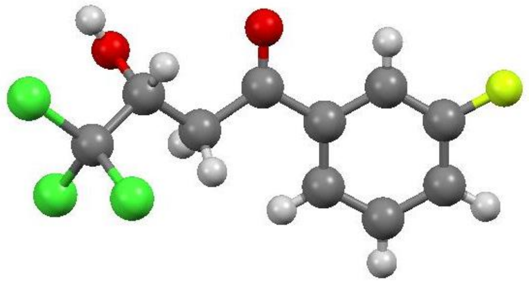

Formula	C_{10} H_8 Cl_3 F O_2
Space Group	P 2_1/n
Cell Lengths	**a** 5.4561(2) **b** 11.4662(4) **c** 17.7928(6)
Cell Angles	**α** 90 **β** 95.044(3) **γ** 90
Cell Volume	1108.82
Z, Z'	**Z**: 4 **Z'**: 0
R-Factor (%)	3

Num	Label	Charge	SybylType	Xfrac + ESD	Yfrac + ESD	Zfrac + ESD	Symm. op.
1	Cl01	0	Cl	-0.12690(8)	0.79450(4)	0.43927(2)	x,y,z
2	Cl02	0	Cl	-0.09000(8)	0.85349(4)	0.59895(3)	x,y,z
3	Cl03	0	Cl	0.33151(8)	0.87436(4)	0.51261(3)	x,y,z
4	F004	0	F	1.1753(2)	0.34565(11)	0.73532(8)	x,y,z
5	O005	0	O.2	0.4078(2)	0.45928(12)	0.57789(8)	x,y,z
6	O006	0	O.3	-0.0783(2)	0.59614(12)	0.54976(8)	x,y,z
7	H006	0	H	-0.143887	0.579732	0.506576	x,y,z
8	C007	0	C.2	0.4436(3)	0.53820(16)	0.62339(11)	x,y,z
9	C008	0	C.2	0.6418(3)	0.52776(16)	0.68713(11)	x,y,z
10	C009	0	C.2	1.0049(3)	0.43160(17)	0.73909(11)	x,y,z
11	C00A	0	C.3	0.1361(3)	0.66184(16)	0.54304(11)	x,y,z
12	H00A	0	H	0.225249	0.629648	0.500877	x,y,z

13	C00B	0	C.3	0.0657(3)	0.79039(16)	0.52559(10)	x,y,z
14	C00C	0	C.2	0.8108(3)	0.43604(17)	0.68450(11)	x,y,z
15	H00C	0	H	0.791508	0.378523	0.646003	x,y,z
16	C00D	0	C.2	0.6704(4)	0.60884(18)	0.74552(12)	x,y,z
17	H00D	0	H	0.554577	0.670306	0.747952	x,y,z
18	C00E	0	C.3	0.3000(3)	0.65160(16)	0.61677(11)	x,y,z
19	H00B	0	H	0.417225	0.717695	0.620486	x,y,z
20	H00E	0	H	0.196446	0.657522	0.659578	x,y,z
21	C00F	0	C.2	1.0396(4)	0.51114(18)	0.79724(12)	x,y,z
22	H00F	0	H	1.176386	0.505196	0.833941	x,y,z
23	C00G	0	C.2	0.8681(4)	0.60020(19)	0.80041(12)	x,y,z
24	H00G	0	H	0.885826	0.655548	0.840249	x,y,z

1h

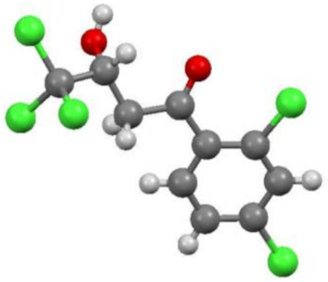

Formula	$C_{10} H_7 Cl_5 O_2$
Space Group	P -1
Cell Lengths	**a** 7.0636(4) **b** 8.0079(4) **c** 11.6593(5)
Cell Angles	**α** 92.100(4) **β** 104.995(4) **γ** 91.259(4)
Cell Volume	636.281
Z, Z’	**Z**: 2 **Z'**: 0
R-Factor (%)	5.39

Num	Label	Charge	SybylType	Xfrac + ESD	Yfrac + ESD	Zfrac + ESD	Symm. op.
1	Cl01	0	Cl	0.24637(12)	0.44565(9)	0.33740(7)	x,y,z
2	Cl02	0	Cl	0.55680(11)	0.22562(10)	0.44331(7)	x,y,z
3	Cl03	0	Cl	0.27625(12)	0.74779(9)	1.11256(7)	x,y,z

4	Cl04	0	Cl	0.22009(13)	0.09787(10)	0.26243(7)	x,y,z
5	Cl05	0	Cl	0.23964(14)	0.11461(10)	0.95590(7)	x,y,z
6	O006	0	O.3	-0.0104(3)	0.1903(3)	0.4363(2)	x,y,z
7	H006	0	H	-0.052477	0.090498	0.427942	x,y,z
8	O007	0	O.2	0.1600(4)	0.1083(3)	0.7026(2)	x,y,z
9	C008	0	C.2	0.2296(5)	0.3742(4)	0.8044(3)	x,y,z
10	C009	0	C.3	0.1947(5)	0.1953(4)	0.4832(3)	x,y,z
11	H009	0	H	0.237236	0.083167	0.513366	x,y,z
12	C00A	0	C.2	0.2057(5)	0.2536(4)	0.6990(3)	x,y,z
13	C00B	0	C.3	0.2419(5)	0.3242(4)	0.5866(3)	x,y,z
14	H00A	0	H	0.159687	0.422486	0.564729	x,y,z
15	H00B	0	H	0.380961	0.362086	0.602540	x,y,z
16	C00C	0	C.3	0.2981(5)	0.2399(4)	0.3867(3)	x,y,z
17	C00D	0	C.2	0.2420(5)	0.5458(4)	0.7900(3)	x,y,z
18	H00D	0	H	0.240200	0.584236	0.713652	x,y,z
19	C00E	0	C.3	0.2601(5)	0.6055(4)	0.9941(3)	x,y,z
20	C00F	0	C.3	0.2370(5)	0.3230(4)	0.9189(3)	x,y,z
21	C00G	0	C.2	0.2567(5)	0.6627(4)	0.8832(3)	x,y,z
22	H00G	0	H	0.264307	0.779009	0.870957	x,y,z
23	C00H	0	C.2	0.2507(5)	0.4373(4)	1.0134(3)	x,y,z
24	H00H	0	H	0.253472	0.400018	1.090172	x,y,z

1s

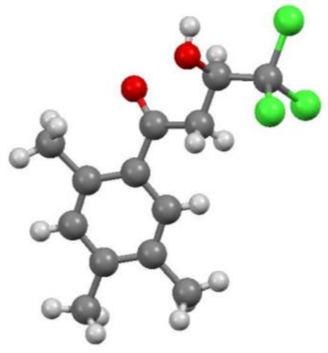

Formula	$C_{13} H_{15} Cl_3 O_2$
Space Group	P -1

Cell Lengths	**a** 5.60310(10) **b** 11.1288(2) **c** 11.2193(2)
Cell Angles	**α** 85.7130(10) **β** 84.904(2) **γ** 79.576(2)
Cell Volume	684.092
Z, Z’	**Z**: 2 **Z'**: 0
R-Factor (%)	2.99

Num	Label	Charge	SybylType	Xfrac + ESD	Yfrac + ESD	Zfrac + ESD	Symm. op.
1	Cl1	0	Cl	0.02805(6)	0.90084(4)	0.83188(3)	x,y,z
2	Cl2	0	Cl	0.52858(6)	0.78847(4)	0.79343(3)	x,y,z
3	Cl3	0	Cl	0.15147(6)	0.65544(3)	0.74756(3)	x,y,z
4	O1	0	O.3	0.0077(2)	0.86501(11)	0.56466(10)	x,y,z
5	H1	0	H	-0.0866	0.9274	0.5890	x,y,z
6	O2	0	O.2	0.3728(2)	0.94243(10)	0.37355(10)	x,y,z
7	C1	0	C.3	0.2371(3)	0.80284(14)	0.73827(14)	x,y,z
8	C2	0	C.3	0.2382(3)	0.85905(14)	0.60787(13)	x,y,z
9	H2	0	H	0.2761	0.9436	0.6064	x,y,z
10	C3	0	C.3	0.4290(3)	0.78177(15)	0.52734(14)	x,y,z
11	H3A	0	H	0.5832	0.7633	0.5673	x,y,z
12	H3B	0	H	0.3756	0.7031	0.5185	x,y,z
13	C4	0	C.2	0.4783(3)	0.84051(14)	0.40393(14)	x,y,z
14	C5	0	C.2	0.6673(3)	0.76903(14)	0.32143(14)	x,y,z
15	C6	0	C.2	0.8608(3)	0.68757(14)	0.36958(14)	x,y,z
16	H6	0	H	0.8654	0.6780	0.4542	x,y,z
17	C7	0	C.2	1.0462(3)	0.62024(14)	0.29881(14)	x,y,z
18	C8	0	C.2	1.0369(3)	0.63333(14)	0.17371(14)	x,y,z
19	C9	0	C.2	0.8421(3)	0.71280(15)	0.12620(15)	x,y,z
20	H9	0	H	0.8353	0.7201	0.0416	x,y,z
21	C10	0	C.2	0.6569(3)	0.78214(14)	0.19588(14)	x,y,z
22	C11	0	C.3	0.4538(3)	0.86476(16)	0.13470(15)	x,y,z
23	H11A	0	H	0.2969	0.8534	0.1758	x,y,z
24	H11B	0	H	0.4593	0.8443	0.0509	x,y,z
25	H11C	0	H	0.4734	0.9502	0.1377	x,y,z
26	C12	0	C.3	1.2519(3)	0.53500(15)	0.35512(15)	x,y,z

27	H12A	0	H	1.4054	0.5638	0.3313	x,y,z
28	H12B	0	H	1.2616	0.4524	0.3281	x,y,z
29	H12C	0	H	1.2224	0.5333	0.4426	x,y,z
30	C13	0	C.3	1.2350(3)	0.56433(16)	0.09208(16)	x,y,z
31	H13A	0	H	1.3911	0.5877	0.1043	x,y,z
32	H13B	0	H	1.1976	0.5843	0.0085	x,y,z
33	H13C	0	H	1.2449	0.4762	0.1104	x,y,z

1t

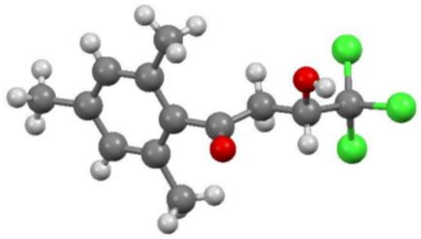

Formula	C_{13} H_{15} Cl_3 O_2
Space Group	P 2_1/c
Cell Lengths	**a** 11.8714(3) **b** 17.6308(4) **c** 7.02120(10
Cell Angles	**α** 90 **β** 97.209(2) **γ** 90
Cell Volume	1457.94
Z, Z'	**Z**: 4 **Z'**: 0
R-Factor (%)	4.18

Number	Label	Charge	SybylType	Xfrac + ESD	Yfrac + ESD	Zfrac + ESD	Symm. op.
1	Cl1	0	Cl	0.68919(5)	0.41057(3)	0.88593(8)	x,y,z
2	Cl2	0	Cl	0.55244(4)	0.27480(3)	0.82924(7)	x,y,z
3	Cl3	0	Cl	0.49524(5)	0.39136(3)	1.08779(7)	x,y,z
4	O1	0	O.3	0.52000(13)	0.40637(9)	0.5281(2)	x,y,z
5	H1	0	H	0.5747	0.4365	0.5270	x,y,z
6	O2	0	O.2	0.29156(14)	0.49506(10)	0.5037(3)	x,y,z
7	C1	0	C.3	0.54822(18)	0.37354(12)	0.8670(3)	x,y,z

8	C2	0	C.3	0.47355(17)	0.41437(11)	0.7010(3)	x,y,z
9	H2	0	H	0.4687	0.4695	0.7325	x,y,z
10	C3	0	C.3	0.35475(18)	0.38106(12)	0.6712(3)	x,y,z
11	H3A	0	H	0.3573	0.3314	0.6061	x,y,z
12	H3B	0	H	0.3290	0.3721	0.7980	x,y,z
13	C4	0	C.2	0.26938(18)	0.43152(13)	0.5532(3)	x,y,z
14	C5	0	C.2	0.15160(19)	0.39867(13)	0.5081(3)	x,y,z
15	C6	0	C.2	0.06946(19)	0.41706(14)	0.6256(3)	x,y,z
16	C7	0	C.2	-0.0390(2)	0.38679(15)	0.5826(4)	x,y,z
17	H7	0	H	-0.0957	0.3992	0.6617	x,y,z
18	C8	0	C.2	-0.0664(2)	0.33870(15)	0.4264(4)	x,y,z
19	C9	0	C.2	0.0177(2)	0.32055(14)	0.3127(4)	x,y,z
20	H9	0	H	0.0002	0.2872	0.2068	x,y,z
21	C10	0	C.2	0.1270(2)	0.35005(13)	0.3505(3)	x,y,z
22	C11	0	C.3	0.2159(2)	0.33044(18)	0.2213(4)	x,y,z
23	H11A	0	H	0.2496	0.3772	0.1786	x,y,z
24	H11B	0	H	0.1803	0.3022	0.1094	x,y,z
25	H11C	0	H	0.2753	0.2992	0.2925	x,y,z
26	C12	0	C.3	0.0940(2)	0.46956(16)	0.7963(4)	x,y,z
27	H12A	0	H	0.1761	0.4718	0.8356	x,y,z
28	H12B	0	H	0.0562	0.4504	0.9029	x,y,z
29	H12C	0	H	0.0656	0.5205	0.7608	x,y,z
30	C13	0	C.3	-0.1853(2)	0.3073(2)	0.3807(4)	x,y,z
31	H13A	0	H	-0.2306	0.3217	0.4825	x,y,z
32	H13B	0	H	-0.1819	0.2519	0.3722	x,y,z
33	H13C	0	H	-0.2204	0.3280	0.2579	x,y,z

1v

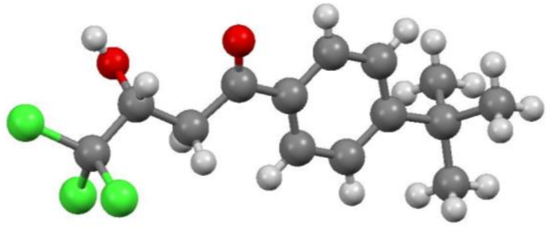

Formula	C_{14} H_{17} Cl_3 O_2
Space Group	P 2_1/n
Cell Lengths	**a** 6.4482(2) **b** 13.2384(3) **c** 17.9026(4)
Cell Angles	**α** 90 **β** 92.152(2) **γ** 90
Cell Volume	1527.16
Z, Z'	**Z**: 4 **Z'**: 0
R-Factor (%)	3.79

Num	Label	Charge	SybylType	Xfrac + ESD	Yfrac + ESD	Zfrac + ESD	Symm. op.
1	Cl1	0	Cl	0.57619(8)	0.67861(4)	0.41440(3)	x,y,z
2	Cl2	0	Cl	0.24014(10)	0.68573(4)	0.30276(3)	x,y,z
3	Cl3	0	Cl	0.18319(10)	0.76474(4)	0.45054(3)	x,y,z
4	O1	0	O.3	0.2456(2)	0.55133(11)	0.50146(8)	x,y,z
5	H1	0	H	0.1417	0.5757	0.5219	x,y,z
6	O2	0	O.2	0.0594(2)	0.36803(12)	0.41498(9)	x,y,z
7	C1	0	C.3	0.3038(3)	0.66758(15)	0.39892(11)	x,y,z
8	C2	0	C.3	0.2224(3)	0.56318(15)	0.42371(11)	x,y,z
9	H2	0	H	0.0721	0.5572	0.4085	x,y,z
10	C3	0	C.3	0.3416(3)	0.47778(14)	0.38837(11)	x,y,z
11	H3A	0	H	0.3534	0.4918	0.3344	x,y,z
12	H3B	0	H	0.4837	0.4755	0.4112	x,y,z
13	C4	0	C.2	0.2400(3)	0.37613(15)	0.39774(11)	x,y,z
14	C5	0	C.2	0.3634(3)	0.28379(15)	0.38318(10)	x,y,z
15	C6	0	C.2	0.5587(3)	0.28664(15)	0.35252(11)	x,y,z
16	H6	0	H	0.6207	0.3498	0.3416	x,y,z
17	C7	0	C.2	0.6637(3)	0.19697(15)	0.33771(11)	x,y,z
18	H7	0	H	0.7957	0.2000	0.3160	x,y,z
19	C8	0	C.2	0.5786(3)	0.10329(15)	0.35417(10)	x,y,z
20	C9	0	C.2	0.3850(3)	0.10205(16)	0.38714(11)	x,y,z
21	H9	0	H	0.3258	0.0392	0.4004	x,y,z
22	C10	0	C.2	0.2788(3)	0.18987(15)	0.40067(11)	x,y,z
23	H10	0	H	0.1465	0.1867	0.4222	x,y,z
24	C11	0	C.3	0.6882(3)	0.00320(16)	0.33916(12)	x,y,z
25	C12	0	C.3	0.8800(4)	0.01701(18)	0.29319(15)	x,y,z

26	H12A	0	H	0.8400	0.0478	0.2450	x,y,z
27	H12B	0	H	0.9442	-0.0489	0.2847	x,y,z
28	H12C	0	H	0.9793	0.0610	0.3202	x,y,z
29	C13	0	C.3	0.7524(5)	-0.04386(19)	0.41508(14)	x,y,z
30	H13A	0	H	0.8494	0.0014	0.4420	x,y,z
31	H13B	0	H	0.8196	-0.1091	0.4070	x,y,z
32	H13C	0	H	0.6290	-0.0538	0.4445	x,y,z
33	C14	0	C.3	0.5402(4)	-0.06916(17)	0.29580(14)	x,y,z
34	H14A	0	H	0.4157	-0.0802	0.3245	x,y,z
35	H14B	0	H	0.6102	-0.1338	0.2882	x,y,z
36	H14C	0	H	0.5005	-0.0394	0.2472	x,y,z

3a

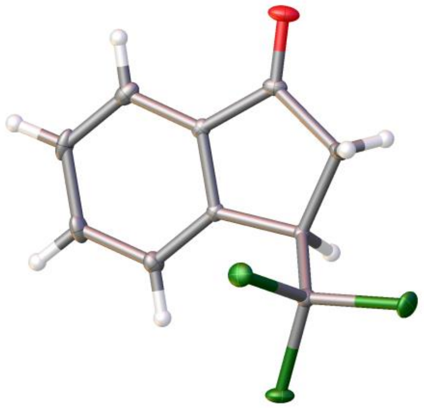

Empirical formula	$C_{10}OCl_3H_{0.25}$
Formula weight	242.70
Temperature/K	100.01(10)
Crystal system	triclinic
Space group	P-1
a/Å	6.1111(2)
b/Å	10.6200(3)
c/Å	16.0284(4)
α/°	76.109(2)
β/°	85.828(2)
γ/°	85.245(3)
Volume/Å^3	1004.84(5)
Z	4
ρ_{calc}g/cm^3	1.604
μ/mm^{-1}	7.931

F(000)	477.0
Crystal size/mm^3	? × ? × ?
Radiation	CuKα (λ = 1.54184)
2Θ range for data collection/°	5.688 to 147.78
Index ranges	-7 ≤ h ≤ 7, -13 ≤ k ≤ 13, -17 ≤ l ≤ 19
Reflections collected	8788
Independent reflections	4027 [R_{int} = 0.0351, R_{sigma} = 0.0367]
Data/restraints/parameters	4027/0/269
Goodness-of-fit on F^2	1.057
Final R indexes [I>=2σ (I)]	R_1 = 0.0391, wR_2 = 0.1021
Final R indexes [all data]	R_1 = 0.0402, wR_2 = 0.1034
Largest diff. peak/hole / e $Å^{-3}$	0.44/-0.67

**Atom**	***x***	***y***	***z***	**U(eq)**
Cl(1)	929.5(7)	5677.9(4)	7218.8(3)	11.72(12)
Cl(3)	6379.7(7)	8745.2(4)	-609.3(3)	15.27(13)
Cl(5)	10959.6(7)	8086.1(5)	-272.8(3)	15.70(13)
Cl(7)	8471.8(8)	6315.7(5)	-852.6(3)	16.25(13)
Cl(10)	4070.9(8)	7359.2(4)	7558.4(3)	16.83(13)
Cl(11)	5233.4(7)	5778.3(5)	6353.0(3)	17.03(13)
O(1)	2317(2)	5990.7(15)	1772.4(9)	17.6(3)
O(3)	-3160(2)	9639.4(15)	5815.5(9)	19.1(3)
C(5)	8329(3)	7447.9(18)	-186.3(12)	10.4(3)
C(8)	3029(3)	6721.9(18)	6740.1(12)	10.0(3)
C(1)	-1000(3)	8072.6(18)	5179.5(11)	9.7(3)
C(4)	7131(3)	7701.4(17)	1337.0(11)	8.9(3)
C(2)	-1521(3)	8915.3(18)	5786.1(12)	11.5(4)
C(7)	581(3)	6459(2)	4144.8(13)	16.2(4)
C(9)	380(3)	8738.5(18)	6372.1(12)	12.3(4)
C(11)	-2325(3)	7910.9(18)	4543.3(12)	12.0(4)
C(13)	5060(3)	7499.0(17)	1757.5(12)	9.5(3)
C(16)	-1514(3)	7078.9(19)	4030.0(12)	13.5(4)
C(18)	7696(3)	6772.6(17)	750.2(11)	8.2(3)
C(20)	2173(3)	7852.3(18)	6023.4(12)	10.3(4)
C(21)	1077(3)	7444.1(17)	5307.5(11)	9.4(4)
C(23)	4081(3)	6437.8(18)	1501.3(12)	11.6(4)
C(25)	5634(3)	5999.5(18)	828.5(12)	11.2(4)
C(28)	8367(3)	8646.8(19)	1504.9(12)	12.9(4)
C(30)	5387(4)	9189.3(19)	2479.0(12)	16.4(4)
C(31)	7473(4)	9387.6(19)	2068.3(12)	16.2(4)
C(33)	1907(3)	6643.5(19)	4771.5(13)	14.5(4)
C(36)	4165(3)	8230.2(19)	2329.3(12)	14.5(4)

6. DFT-calculations data for compounds 1a, 2a, 3a and cations Aa, Ba, Ca, Ea

1a

Energy E(B3LYP) = -1917.78454462 h, G^{298} = -1917.660907 h, μ=6.86 D

Cartesian coordinates, Å

N	atom	x	y	z
1	C	5.293077	0.785122	-0.069951
2	C	4.195812	1.636145	-0.165850
3	C	2.905539	1.120813	-0.103682
4	C	2.698612	-0.255143	0.060034
5	C	3.812163	-1.101730	0.159527
6	C	5.098218	-0.586538	0.092338
7	C	1.335634	-0.856565	0.151340
8	O	1.188446	-2.051416	0.352131
9	C	0.124850	0.064955	0.044417
10	C	-1.165400	-0.702228	-0.234485
11	O	-1.214164	-1.192092	-1.551784
12	C	-2.441942	0.126924	0.083902
13	Cl	-2.507786	1.641621	-0.895055
14	Cl	-3.910865	-0.850765	-0.285255
15	Cl	-2.487131	0.556594	1.834716
16	H	2.071207	1.805892	-0.178720
17	H	3.657003	-2.165583	0.287484
18	H	4.342755	2.703204	-0.289342
19	H	5.951272	-1.251706	0.167532
20	H	6.298761	1.188329	-0.120730
21	H	-0.976023	-0.479649	-2.183587
22	H	0.280818	0.816185	-0.730338
23	H	0.062710	0.606892	0.991801
24	H	-1.223868	-1.572684	0.422693

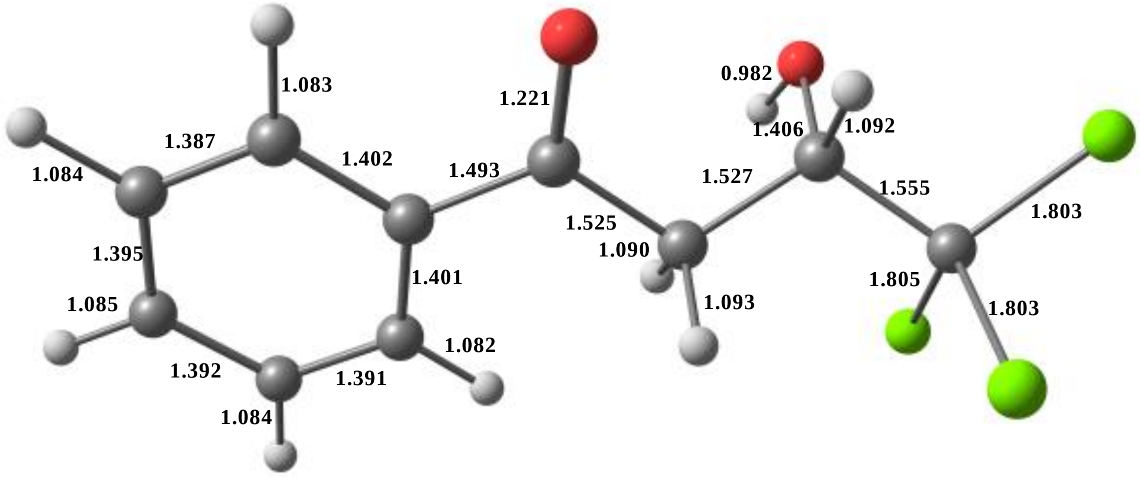
1.083
1.221
0.982
1.387
1.402
1.406
1.092
1.084
1.493
1.527
1.555
1.525
1.803
1.395
1.090
1.401
1.805
1.085
1.093
1.803
1.082
1.392
1.391
1.084

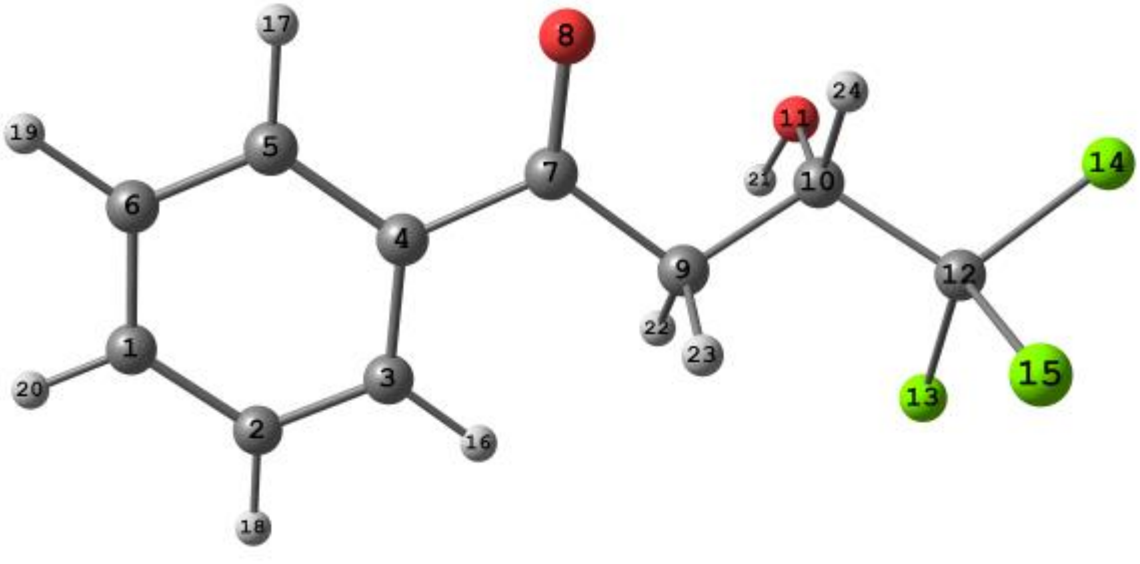
17
8
24
19
11
5
7
14
6
21
10
4
9
12
1
22
23
20
3
15
13
2
16
18

Summary of Natural Population Analysis:

Atom	No	Natural Charge	Natural Population: Core	Valence	Rydberg	Total
C	1	-0.17715	1.99915	4.15766	0.02034	6.17715
C	2	-0.21404	1.99915	4.19430	0.02059	6.21404
C	3	-0.17111	1.99908	4.15419	0.01784	6.17111
C	4	-0.16505	1.99892	4.14595	0.02018	6.16505
C	5	-0.15819	1.99907	4.13876	0.02036	6.15819
C	6	-0.21342	1.99915	4.19419	0.02008	6.21342
C	7	0.60045	1.99924	3.36531	0.03500	5.39955
O	8	-0.61737	1.99976	6.59247	0.02514	8.61737
C	9	-0.51603	1.99912	4.49690	0.02001	6.51603
C	10	0.07652	1.99879	3.88793	0.03676	5.92348
O	11	-0.75940	1.99978	6.73823	0.02140	8.75940
C	12	-0.15939	1.99911	4.09536	0.06491	6.15939
Cl	13	0.01941	9.99947	6.96013	0.02099	16.98059
Cl	14	0.03278	9.99947	6.94716	0.02059	16.96722
Cl	15	0.03008	9.99947	6.95026	0.02018	16.96992
H	16	0.22542	0.00000	0.77270	0.00188	0.77458
H	17	0.23359	0.00000	0.76407	0.00233	0.76641
H	18	0.22766	0.00000	0.77073	0.00161	0.77234
H	19	0.22679	0.00000	0.77157	0.00164	0.77321
H	20	0.22637	0.00000	0.77214	0.00149	0.77363
H	21	0.51506	0.00000	0.48218	0.00276	0.48494
H	22	0.24057	0.00000	0.75676	0.00266	0.75943
H	23	0.26084	0.00000	0.73694	0.00222	0.73916
H	24	0.23561	0.00000	0.75988	0.00450	0.76439
* Total *		0.00000	53.98875	81.60577	0.40548	136.00000

2a

Energy E(B3LYP) = -1841.29969838 h, G^{298} = -1841.199818-1725.917608 h, μ=5.15 D

Cartesian coordinates, Å

N	atom	x	y	z
1	C	-5.010502	-0.587625	0.005795
2	C	-5.184905	0.797068	0.015025
3	C	-4.075328	1.636338	0.013636
4	C	-2.792625	1.098531	0.006173
5	C	-2.604839	-0.290469	-0.004873
6	C	-3.733377	-1.125390	-0.006587
7	C	-1.259950	-0.932906	-0.016785
8	C	-0.037023	-0.073081	-0.070987
9	C	1.164674	-0.634549	0.032474
10	C	2.479516	0.067308	0.005249
11	Cl	2.379460	1.842016	-0.198994
12	O	-1.148831	-2.153439	0.019666
13	Cl	3.349047	-0.287981	1.557588
14	Cl	3.460025	-0.616131	-1.360715
15	H	-1.951629	1.779273	0.003820
16	H	-3.591806	-2.198476	-0.014611
17	H	-4.205797	2.712592	0.019544
18	H	-5.873574	-1.244023	0.007300
19	H	-6.184416	1.218372	0.023201
20	H	-0.138550	0.994441	-0.188827
21	H	1.257529	-1.710377	0.153592

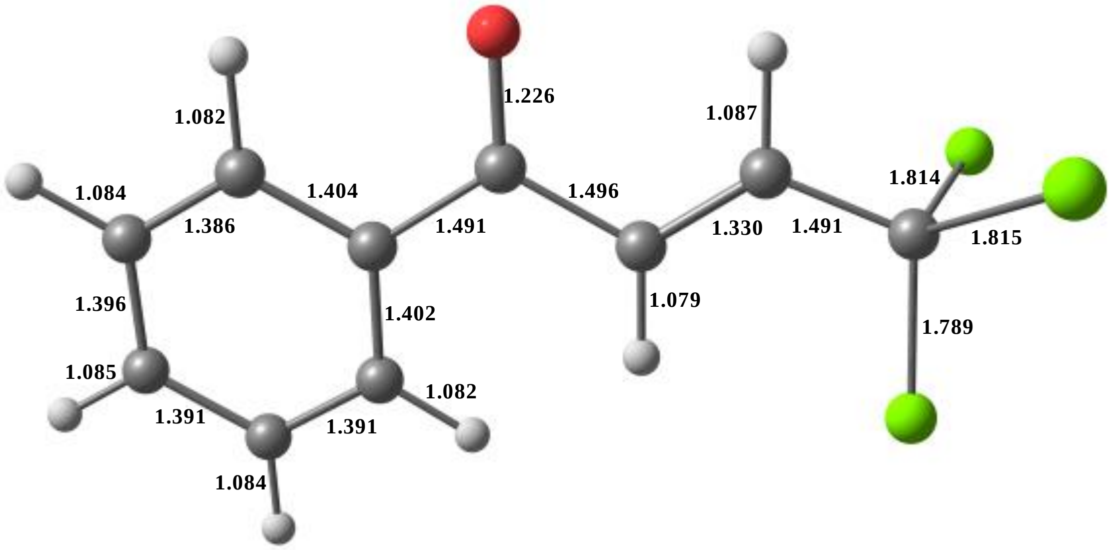

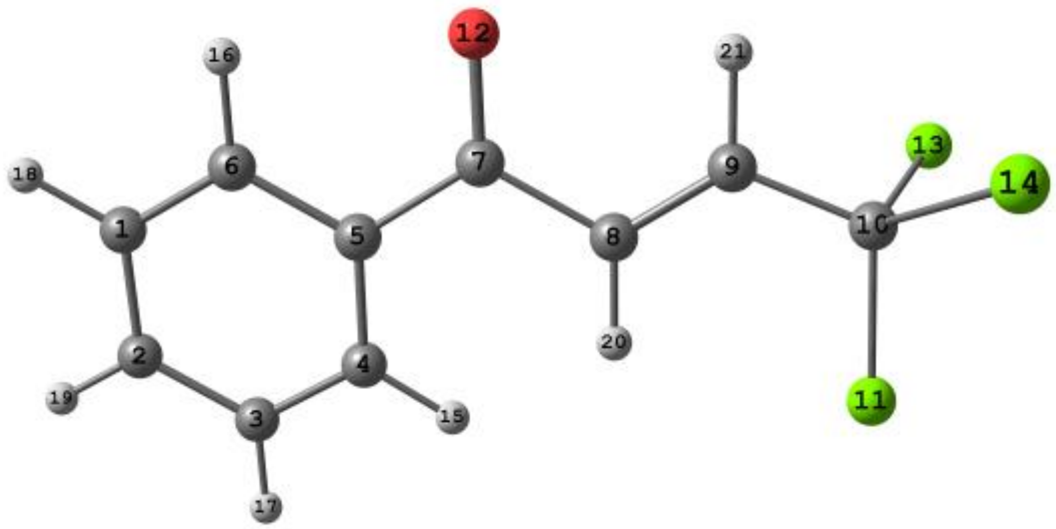

Summary of Natural Population Analysis:

Atom	No	Natural Charge	Natural Population Core	Valence	Rydberg	Total
C	1	-0.21213	1.99915	4.19287	0.02011	6.21213
C	2	-0.17255	1.99915	4.15305	0.02035	6.17255
C	3	-0.21477	1.99915	4.19511	0.02051	6.21477
C	4	-0.16748	1.99908	4.15034	0.01806	6.16748
C	5	-0.15400	1.99893	4.13542	0.01964	6.15400
C	6	-0.15781	1.99907	4.13831	0.02043	6.15781
C	7	0.54227	1.99920	3.42484	0.03369	5.45773
C	8	-0.25140	1.99890	4.23146	0.02104	6.25140
C	9	-0.18316	1.99881	4.15621	0.02814	6.18316
C	10	-0.16206	1.99902	4.09970	0.06334	6.16206
Cl	11	0.04941	9.99948	6.93093	0.02017	16.95059
O	12	-0.61945	1.99977	6.59542	0.02427	8.61945
Cl	13	0.03352	9.99952	6.94806	0.01890	16.96648
Cl	14	0.03295	9.99952	6.94866	0.01887	16.96705
H	15	0.22540	0.00000	0.77280	0.00180	0.77460
H	16	0.23451	0.00000	0.76318	0.00231	0.76549
H	17	0.22845	0.00000	0.76994	0.00161	0.77155
H	18	0.22752	0.00000	0.77085	0.00164	0.77248
H	19	0.22702	0.00000	0.77150	0.00149	0.77298
H	20	0.23379	0.00000	0.76364	0.00257	0.76621
H	21	0.25998	0.00000	0.73674	0.00328	0.74002
* Total *		0.00000	51.98876	73.64901	0.36222	126.00000

3a

Energy E(B3LYP) = -1841.3259343 h, G^{298} = -1841.222557 h, μ=5.19 D

Cartesian coordinates, Å

N	atom	x	y	z
1	C	1.357290	-1.750530	-0.551988
2	C	0.988762	-0.409925	-0.486816
3	C	1.930343	0.541842	-0.087480
4	C	3.223984	0.186990	0.293541
5	C	3.577031	-1.153386	0.254672
6	C	2.649899	-2.109312	-0.175700
7	C	-0.337073	0.247694	-0.863290
8	C	-0.035484	1.765854	-0.780180
9	H	0.670587	-2.511961	-0.894561
10	H	3.933943	0.948715	0.597112
11	H	4.575956	-1.463519	0.540168
12	H	2.944468	-3.152220	-0.224287
13	H	-0.045794	2.215259	-1.775055
14	C	1.366104	1.894805	-0.203380
15	O	1.912990	2.947343	0.076628
16	C	-1.538750	-0.157564	0.014122
17	Cl	-3.009382	0.766269	-0.504663
18	Cl	-1.933555	-1.909034	-0.156152
19	Cl	-1.240040	0.187134	1.761604
20	H	-0.740033	2.328839	-0.171072
21	H	-0.625079	-0.036937	-1.879732

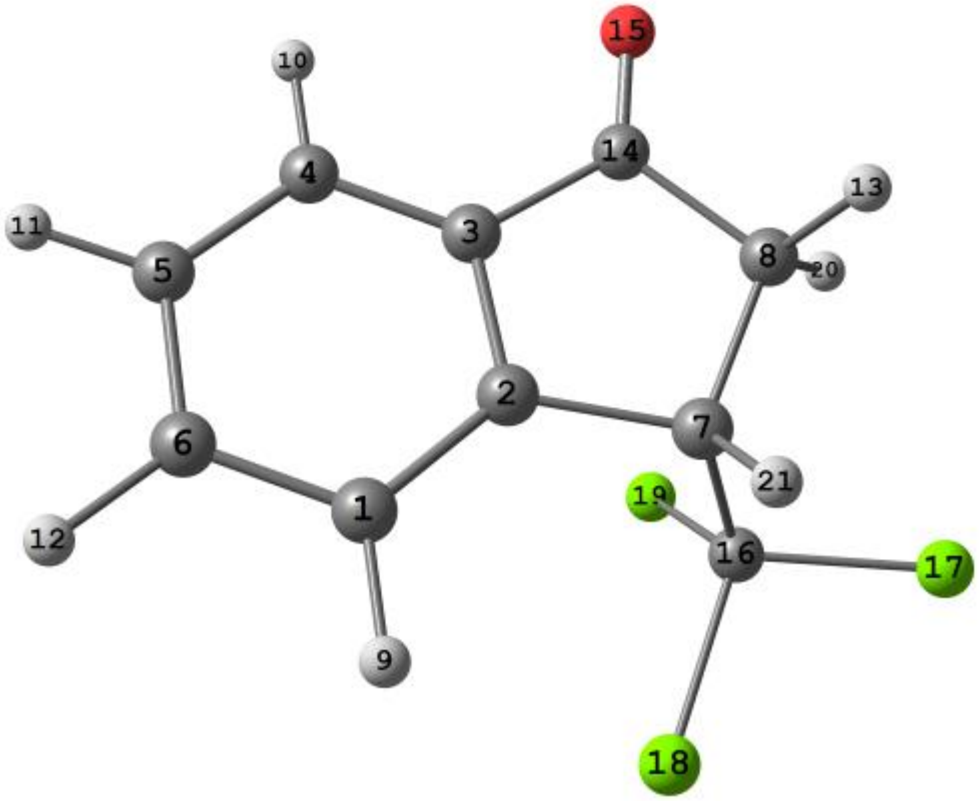

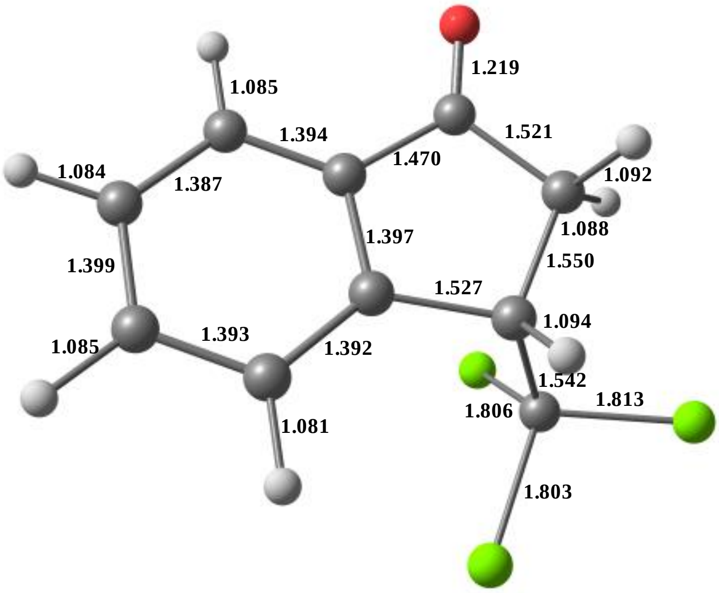
1.219
1.085
1.394
1.521
1.470
1.084
1.387
1.092
1.088
1.397
1.399
1.550
1.527
1.094
1.393
1.085
1.392
1.542
1.813
1.806
1.081
1.803

Aa

Energy E(B3LYP) = -1918.5 8095273 h, G^{298} = -1918.435599 h, μ=10.3 D

Cartesian coordinates, Å

N	atom	x	y	z
1	C	-5.191665	-0.877797	-0.029473
2	C	-4.077255	-1.665119	-0.323093
3	C	-2.813520	-1.108196	-0.304220
4	C	-2.650472	0.259528	0.012929
5	C	-3.790599	1.045400	0.309072
6	C	-5.046156	0.475239	0.286467
7	C	-1.355066	0.865263	0.040342
8	O	-1.330656	2.120488	0.307331
9	C	-0.123916	0.052599	-0.254600
10	C	1.233424	0.734396	-0.193200
11	O	1.440422	1.376832	-1.519957
12	C	2.406636	-0.210811	0.152468
13	Cl	2.459478	-1.592603	-0.985741
14	Cl	3.947148	0.694655	0.066322
15	Cl	2.145505	-0.789544	1.821781
16	H	-1.965995	-1.741895	-0.533975
17	H	-3.677165	2.092913	0.557133
18	H	-4.200173	-2.714340	-0.565552
19	H	-5.917630	1.077835	0.515765
20	H	-6.181842	-1.322117	-0.045220
21	H	1.590230	0.765858	-2.306253
22	H	-0.264860	-0.371799	-1.256157
23	H	-0.133040	-0.805204	0.421629
24	H	1.291100	1.579461	0.504970
25	H	-0.462760	2.598227	0.427010
26	H	2.109303	2.126865	-1.538650

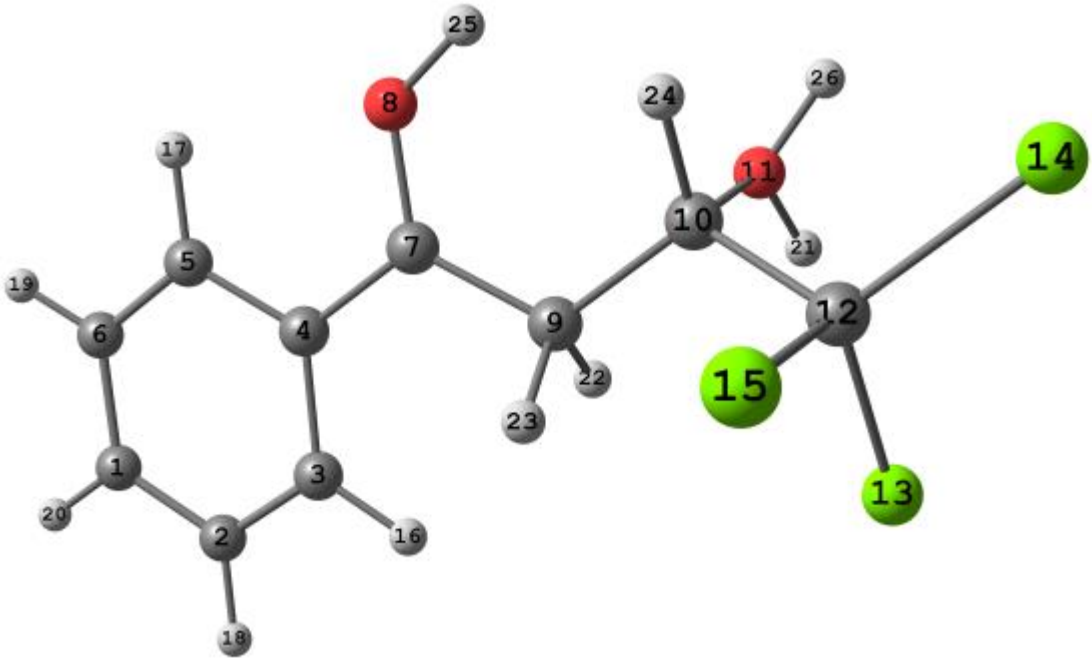

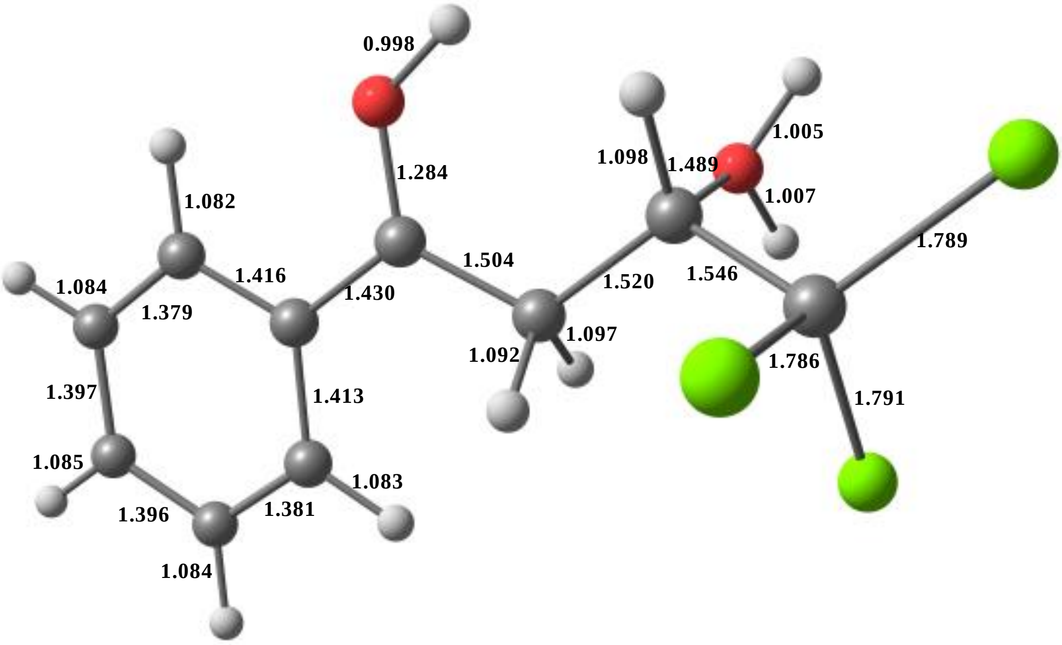
0.998
1.284
1.082
1.416
1.084
1.379
1.430
1.504
1.520
1.098
1.489
1.005
1.007
1.789
1.546
1.097
1.092
1.786
1.791
1.413
1.397
1.085
1.083
1.396
1.381
1.084

Summary of Natural Population Analysis:

Atom	No	Natural Charge	Core	Valence	Rydberg	Total
			Natural Population			
C	1	-0.09676	1.99917	4.07804	0.01955	6.09676
C	2	-0.21022	1.99915	4.19071	0.02036	6.21022
C	3	-0.11036	1.99909	4.09438	0.01689	6.11036
C	4	-0.18806	1.99892	4.16979	0.01935	6.18806
C	5	-0.09913	1.99908	4.08059	0.01947	6.09913
C	6	-0.21023	1.99915	4.19112	0.01996	6.21023
C	7	0.66296	1.99902	3.31272	0.02530	5.33704
O	8	-0.58050	1.99966	6.55666	0.02418	8.58050
C	9	-0.54086	1.99905	4.52003	0.02178	6.54086
C	10	0.07354	1.99873	3.89397	0.03375	5.92646
O	11	-0.67343	1.99973	6.65619	0.01751	8.67343
C	12	-0.20317	1.99909	4.13235	0.07173	6.20317
Cl	13	0.07313	9.99948	6.90660	0.02079	16.92687
Cl	14	0.08230	9.99948	6.89701	0.02121	16.91770
Cl	15	0.09575	9.99946	6.88413	0.02066	16.90425
H	16	0.23897	0.00000	0.75907	0.00196	0.76103
H	17	0.24844	0.00000	0.74949	0.00207	0.75156
H	18	0.24409	0.00000	0.75438	0.00153	0.75591
H	19	0.24331	0.00000	0.75512	0.00157	0.75669
H	20	0.23976	0.00000	0.75889	0.00135	0.76024
H	21	0.61280	0.00000	0.38496	0.00225	0.38720
H	22	0.30444	0.00000	0.69354	0.00202	0.69556
H	23	0.30940	0.00000	0.68827	0.00233	0.69060
H	24	0.28912	0.00000	0.70755	0.00333	0.71088
H	25	0.57484	0.00000	0.42141	0.00375	0.42516
H	26	0.61989	0.00000	0.37816	0.00195	0.38011
* Total *		2.00000	53.98826	81.61514	0.39659	136.00000

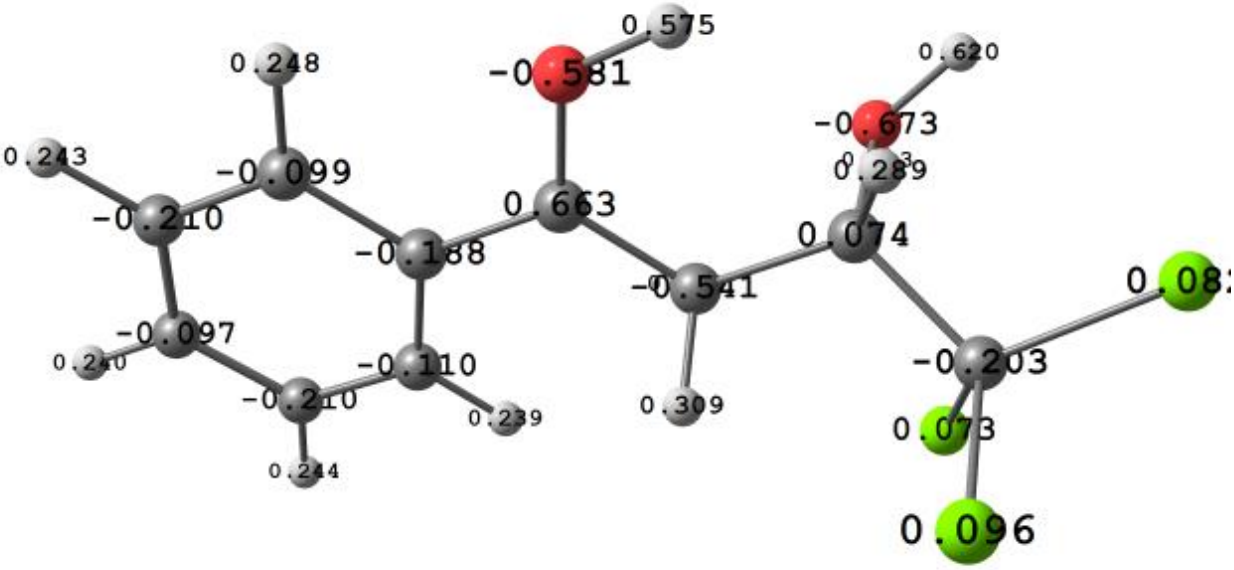

Ba

Energy E(B3LYP) = -1841.71903985 h, G^{298} = -1841.609241 h, μ=8.96 D

Cartesian coordinates, Å

N	atom	x	y	z
1	C	-4.945625	0.465678	0.108246
2	C	-5.027481	-0.929699	0.108320
3	C	-3.874720	-1.710724	0.022652
4	C	-2.635804	-1.104240	-0.066247
5	C	-2.538147	0.304079	-0.064533
6	C	-3.714533	1.083839	0.024927
7	C	-1.259305	0.949250	-0.124944
8	C	-0.024740	0.217583	-0.367178
9	C	1.130714	0.599686	0.183349
10	C	2.446448	-0.097780	0.033982
11	Cl	2.958419	-0.654208	1.672929
12	O	-1.256921	2.230200	0.064973
13	Cl	2.423867	-1.489444	-1.080999
14	Cl	3.630054	1.125878	-0.572149
15	H	-1.749346	-1.724127	-0.122327
16	H	-3.648768	2.164395	0.016215
17	H	-3.947390	-2.792134	0.026102
18	H	-5.848219	1.062751	0.169895
19	H	-5.998245	-1.410604	0.173344
20	H	-0.094014	-0.680635	-0.967494
21	H	-0.403464	2.701617	-0.135334
22	H	1.194190	1.473269	0.832096

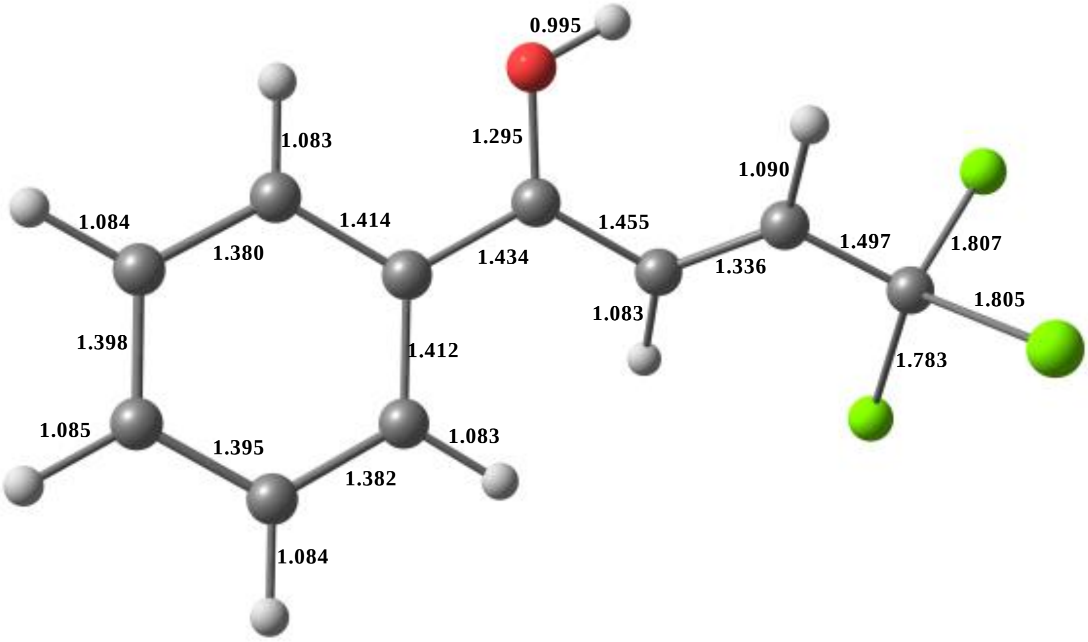
0.995
1.083
1.295
1.090
1.084
1.414
1.455
1.497
1.807
1.380
1.434
1.336
1.805
1.083
1.398
1.412
1.783
1.085
1.083
1.395
1.382
1.084

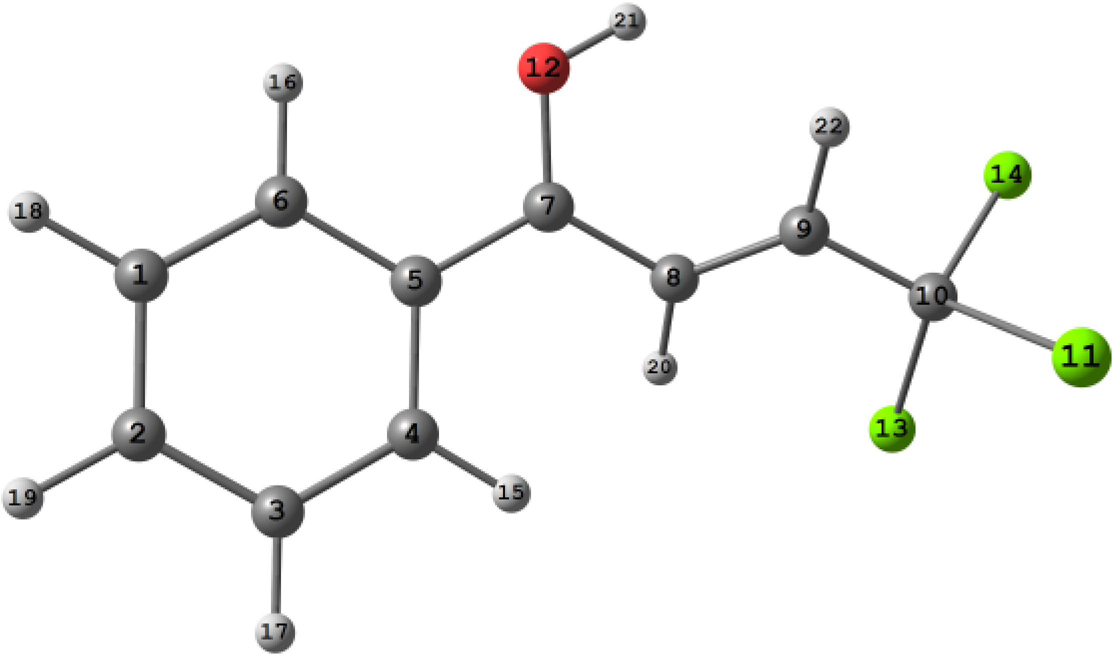
21
12
16
22
14
18
6
7
9
1
5
8
10
11
20
2
4
13
19
15
3
17

Summary of Natural Population Analysis:

Atom	No	Natural Charge	Natural Population Core	Valence	Rydberg	Total
C	1	-0.20963	1.99915	4.19049	0.02000	6.20963
C	2	-0.10624	1.99917	4.08746	0.01961	6.10624
C	3	-0.21357	1.99915	4.19408	0.02034	6.21357
C	4	-0.11186	1.99909	4.09568	0.01708	6.11186
C	5	-0.16844	1.99893	4.15020	0.01932	6.16844
C	6	-0.11087	1.99908	4.09245	0.01933	6.11087
C	7	0.59719	1.99898	3.37754	0.02629	5.40281
C	8	-0.27840	1.99890	4.25824	0.02126	6.27840
C	9	-0.12794	1.99882	4.10214	0.02698	6.12794
C	10	-0.18238	1.99905	4.11821	0.06513	6.18238
Cl	11	0.06016	9.99951	6.92118	0.01915	16.93984
O	12	-0.59353	1.99969	6.57038	0.02347	8.59353
Cl	13	0.06825	9.99948	6.91209	0.02018	16.93175
Cl	14	0.05982	9.99952	6.92162	0.01905	16.94018
H	15	0.24023	0.00000	0.75794	0.00184	0.75977
H	16	0.24641	0.00000	0.75158	0.00201	0.75359
H	17	0.24175	0.00000	0.75670	0.00155	0.75825
H	18	0.24089	0.00000	0.75754	0.00157	0.75911
H	19	0.23766	0.00000	0.76097	0.00137	0.76234
H	20	0.27334	0.00000	0.72426	0.00240	0.72666
H	21	0.57005	0.00000	0.42747	0.00248	0.42995
H	22	0.26712	0.00000	0.73009	0.00278	0.73288
* Total *		1.00000	51.98852	73.65829	0.35320	126.00000

Ca

Energy E(B3LYP) = -1918.20280752 h, G^{298} = -1918.065582 h, μ=9.01 D

Cartesian coordinates, Å

N	atom	x	y	z
1	C	-5.065015	-0.547258	-0.188949
2	C	-5.261346	0.828773	-0.045269
3	C	-4.175792	1.688702	0.118925
4	C	-2.889117	1.181455	0.136710
5	C	-2.676391	-0.206208	-0.005509
6	C	-3.786478	-1.066746	-0.168396
7	C	-1.356539	-0.763341	0.028180
8	C	-0.118019	0.046208	0.283089
9	C	1.146469	-0.593867	-0.312065
10	C	2.433761	0.230961	-0.073184
11	Cl	2.252073	1.860107	-0.809172
12	O	-1.244682	-2.033518	-0.131985
13	O	1.300099	-1.933125	0.141746
14	Cl	2.767520	0.399335	1.687223
15	Cl	3.822937	-0.600783	-0.848939
16	H	-2.060896	1.867820	0.258338
17	H	-3.632936	-2.133382	-0.272883
18	H	-4.336249	2.755027	0.227891
19	H	-5.915308	-1.207740	-0.314477
20	H	-6.268708	1.232214	-0.062529
21	H	1.452900	-1.971975	1.119917
22	H	-0.024489	0.129554	1.375293
23	H	-0.250391	1.053780	-0.099857
24	H	1.044016	-0.663201	-1.400438
25	H	-0.283476	-2.318223	-0.045449

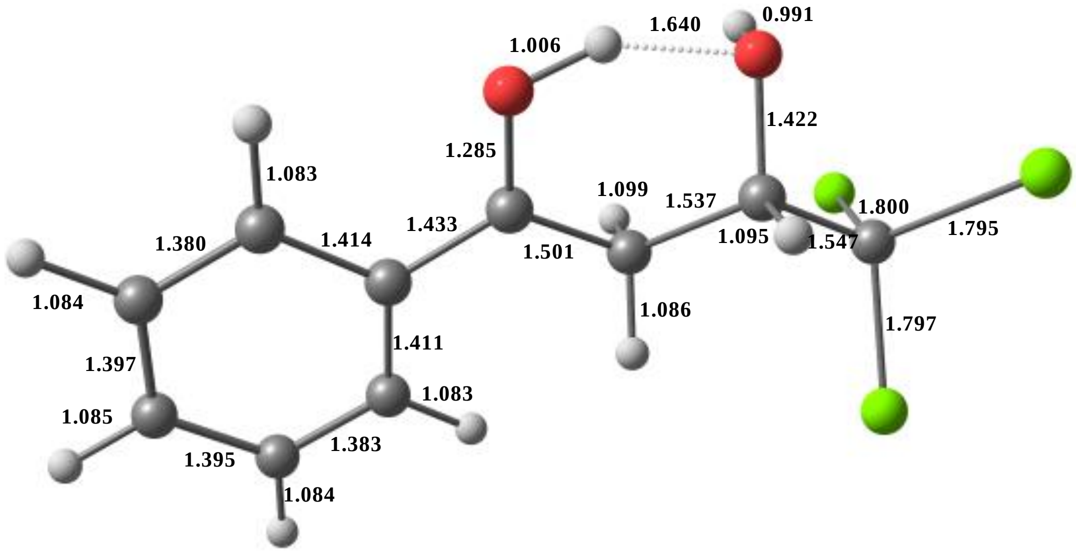
1.640
0.991
1.006
1.422
1.285
1.083
1.099
1.537
1.800
1.433
1.380
1.414
1.501
1.095
1.547
1.795
1.084
1.086
1.411
1.797
1.397
1.083
1.085
1.383
1.395
1.084

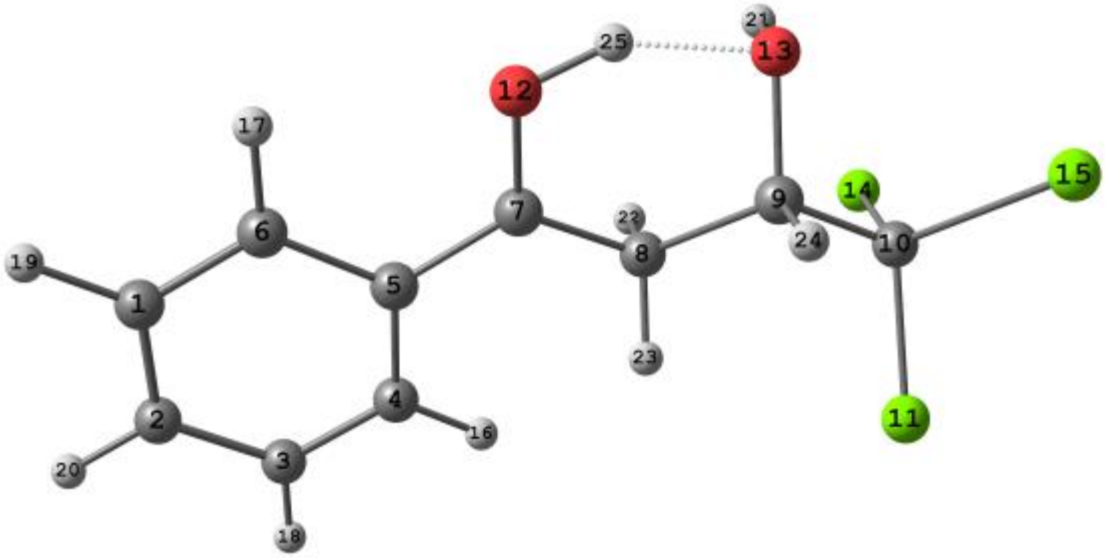
21
25
13
12
17
15
14
9
6
7
22
24
10
19
8
5
1
23
4
2
16
11
20
3
18

Summary of Natural Population Analysis:

Atom	No	Natural Charge	Natural Population Core	Valence	Rydberg	Total
C	1	-0.21021	1.99915	4.19115	0.01991	6.21021
C	2	-0.10840	1.99916	4.08966	0.01957	6.10840
C	3	-0.21292	1.99915	4.19342	0.02035	6.21292
C	4	-0.10973	1.99909	4.09365	0.01699	6.10973
C	5	-0.18977	1.99892	4.17133	0.01951	6.18977
C	6	-0.10655	1.99908	4.08786	0.01960	6.10655
C	7	0.65970	1.99906	3.31452	0.02672	5.34030
C	8	-0.52133	1.99909	4.50186	0.02038	6.52133
C	9	0.06539	1.99880	3.90077	0.03504	5.93461
C	10	-0.17254	1.99909	4.10586	0.06759	6.17254
Cl	11	0.05436	9.99948	6.92576	0.02041	16.94564
O	12	-0.58092	1.99968	6.55460	0.02663	8.58092
O	13	-0.76149	1.99978	6.74156	0.02015	8.76149
Cl	14	0.04005	9.99948	6.93947	0.02100	16.95995
Cl	15	0.05666	9.99948	6.92305	0.02081	16.94334
H	16	0.23717	0.00000	0.76102	0.00181	0.76283
H	17	0.24578	0.00000	0.75196	0.00226	0.75422
H	18	0.24149	0.00000	0.75696	0.00155	0.75851
H	19	0.24068	0.00000	0.75773	0.00158	0.75932
H	20	0.23765	0.00000	0.76098	0.00137	0.76235
H	21	0.54406	0.00000	0.45338	0.00256	0.45594
H	22	0.28865	0.00000	0.70905	0.00231	0.71135
H	23	0.27563	0.00000	0.72217	0.00220	0.72437
H	24	0.25636	0.00000	0.74092	0.00271	0.74364
H	25	0.53023	0.00000	0.46427	0.00549	0.46977
* Total *		1.00000	53.98849	81.61300	0.39851	136.00000

Ea

Energy E(B3LYP) = -1842.08585995 h, G^{298} = -1841.963847 h, μ=7.44 D

Cartesian coordinates, Å

N	atom	x	y	z
1	C	-4.587673	0.414557	0.286227
2	C	-4.613254	-0.983834	0.242338
3	C	-3.464899	-1.710617	-0.072268
4	C	-2.283532	-1.049224	-0.345037
5	C	-2.238208	0.364832	-0.298967
6	C	-3.417124	1.088719	0.019136
7	C	-1.035578	1.076656	-0.562261
8	C	0.273381	0.434110	-0.931947
9	C	1.274481	0.452540	0.229114
10	C	2.657693	-0.013099	-0.128401
11	Cl	0.841656	-0.718259	1.551784
12	O	-1.085103	2.360770	-0.478283
13	Cl	2.897767	-1.182485	-1.265376
14	Cl	3.947706	0.649063	0.650732
15	H	-1.409057	-1.635328	-0.596654
16	H	-3.394409	2.170640	0.051459
17	H	-3.497404	-2.793381	-0.107558
18	H	-5.488266	0.966932	0.528907
19	H	-5.539663	-1.509533	0.451811
20	H	0.704398	1.030466	-1.746837
21	H	0.126382	-0.576729	-1.300498
22	H	1.352244	1.434672	0.705854
23	H	-0.246318	2.856826	-0.719207

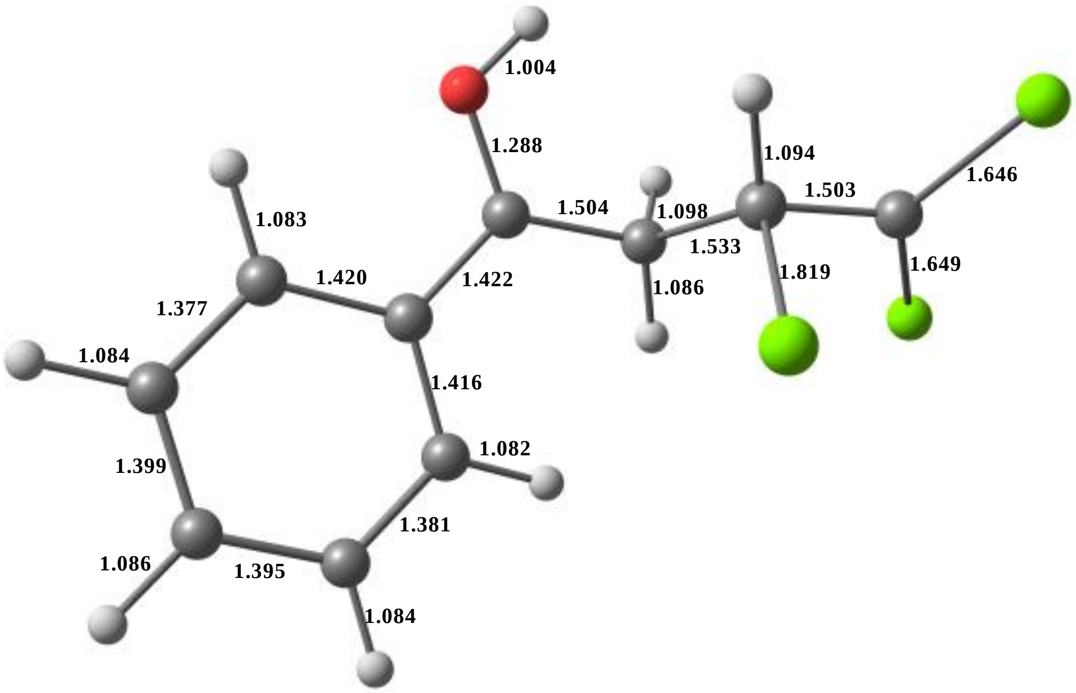

1.004
1.288
1.083
1.504
1.098
1.094
1.503
1.646
1.420
1.422
1.533
1.819
1.649
1.377
1.086
1.084
1.416
1.399
1.082
1.381
1.086
1.395
1.084

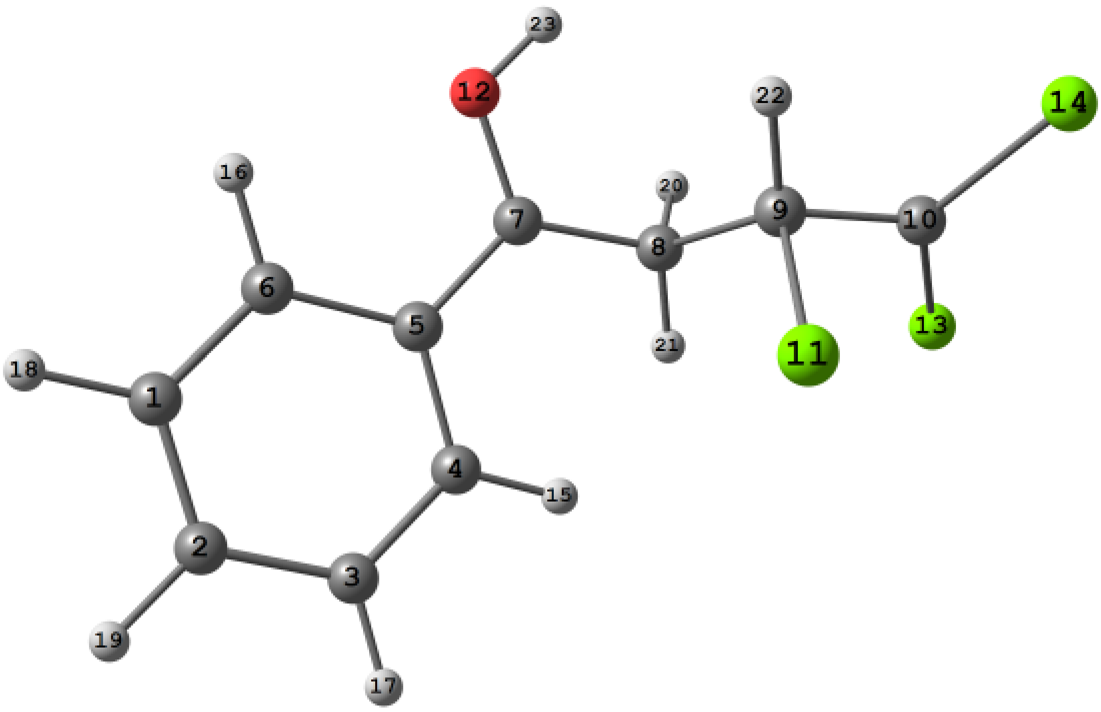

23
12
22
14
16
20
9
10
7
6
8
5
13
21
11
18
1
4
15
2
3
19
17

Summary of Natural Population Analysis:

Atom	No	Natural Charge	Natural Population Core	Valence	Rydberg	Total
C	1	-0.20863	1.99915	4.18961	0.01987	6.20863
C	2	-0.08520	1.99917	4.06664	0.01939	6.08520
C	3	-0.21482	1.99915	4.19521	0.02046	6.21482
C	4	-0.09770	1.99909	4.08139	0.01722	6.09770
C	5	-0.18763	1.99890	4.16865	0.02007	6.18763
C	6	-0.09511	1.99908	4.07648	0.01955	6.09511
C	7	0.64568	1.99902	3.32814	0.02716	5.35432
C	8	-0.50182	1.99907	4.47798	0.02476	6.50182
C	9	-0.27687	1.99873	4.24237	0.03578	6.27687
C	10	0.05554	1.99905	3.89145	0.05395	5.94446
Cl	11	0.08788	9.99968	6.89621	0.01623	16.91212
O	12	-0.58634	1.99967	6.56164	0.02502	8.58634
Cl	13	0.38952	9.99917	6.57880	0.03252	16.61048
Cl	14	0.40455	9.99917	6.56369	0.03260	16.59545
H	15	0.23910	0.00000	0.75889	0.00201	0.76090
H	16	0.24950	0.00000	0.74842	0.00208	0.75050
H	17	0.24583	0.00000	0.75263	0.00154	0.75417
H	18	0.24484	0.00000	0.75360	0.00156	0.75516
H	19	0.24110	0.00000	0.75756	0.00134	0.75890
H	20	0.29094	0.00000	0.70682	0.00224	0.70906
H	21	0.27456	0.00000	0.72324	0.00220	0.72544
H	22	0.30448	0.00000	0.69224	0.00327	0.69552
H	23	0.58057	0.00000	0.41700	0.00243	0.41943
* Total *		2.00000	51.98811	73.62867	0.38322	126.00000

		HOMO		LUMO
**1a**		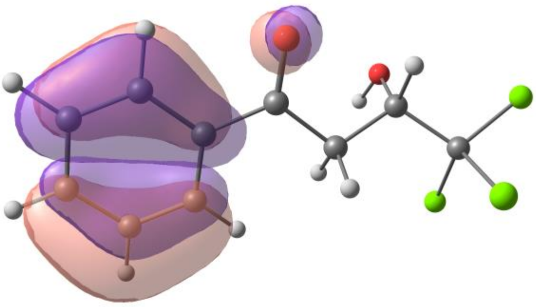		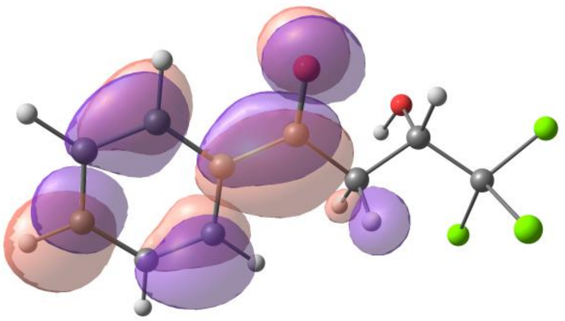
**Ca**		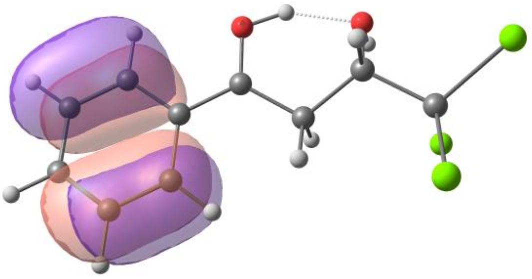		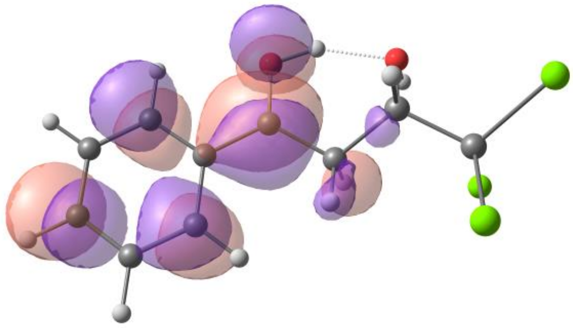
**Aa**		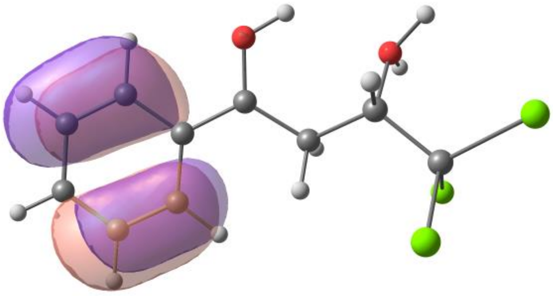		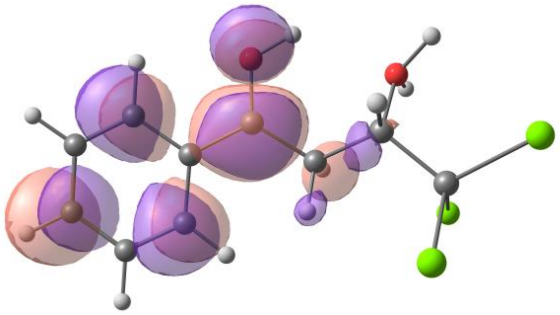
**2a**		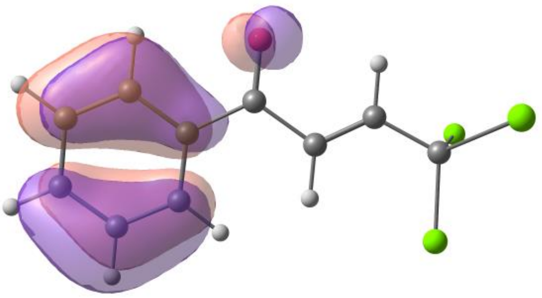		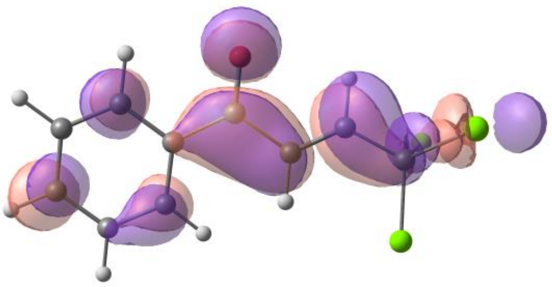
**Ba**		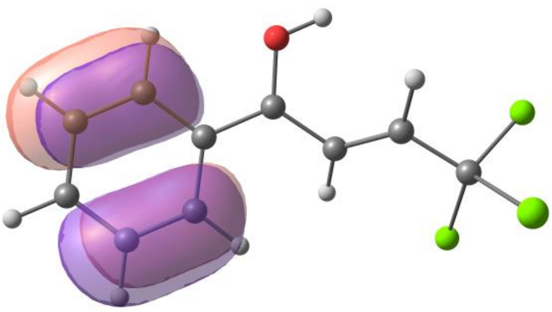		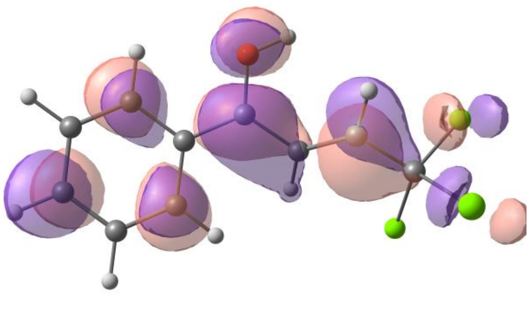

**Ea**		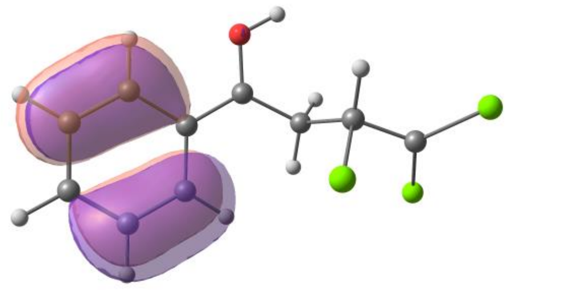		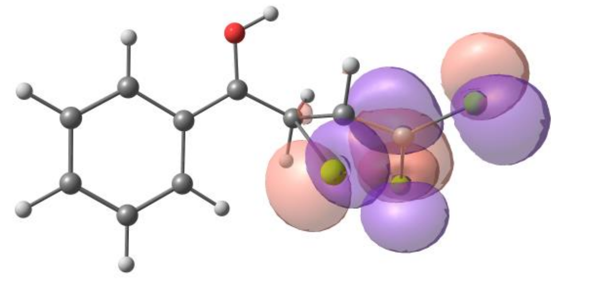
